# Best step-up treatments for children with uncontrolled asthma: a systematic review and network meta-analysis of individual participant data

**DOI:** 10.1183/13993003.01011-2023

**Published:** 2023-12-21

**Authors:** Sofia Cividini, Ian Sinha, Sarah Donegan, Michelle Maden, Katie Rose, Olivia Fulton, Giovanna Culeddu, Dyfrig A. Hughes, Stephen Turner, Catrin Tudur Smith

**Affiliations:** 1 Department of Health Data Science, Institute of Population Health, University of Liverpool, Liverpool, UK; 2 Alder Hey Children's Foundation NHS Trust, Liverpool, UK; 3 Liverpool Reviews and Implementation Group, Institute of Population Health, University of Liverpool, Liverpool, UK; 4 Patient Representative, Liverpool, UK; 5 Centre for Health Economics and Medicines Evaluation, Bangor University, Bangor, UK; 6 Women and Children Division, NHS Grampian, Aberdeen, UK; 7 Institute of Applied Health Sciences, University of Aberdeen, Aberdeen, UK

## Abstract

**Background:**

There is uncertainty about the best treatment option for children/adolescents with uncontrolled asthma despite inhaled corticosteroids (ICS) and international guidelines make different recommendations. We evaluated the pharmacological treatments to reduce asthma exacerbations and symptoms in uncontrolled patients age <18 years on ICS.

**Methods:**

We searched MEDLINE, Cochrane Database of Systematic Reviews, Cochrane Central Register of Controlled Trials, Embase, Web of Science, National Institute for Health and Care Excellence Technology Appraisals, National Institute for Health and Care Research Health Technology Assessment series, World Health Organization International Clinical Trials Registry, conference abstracts and internal clinical trial registers (1 July 2014 to 5 May 2023) for randomised controlled trials of participants age <18 years with uncontrolled asthma on any ICS dose alone at screening. Studies before July 2014 were retrieved from previous systematic reviews/contact with authors. Patients had to be randomised to any dose of ICS alone or combined with long-acting β_2_-agonists (LABA) or combined with leukotriene receptor antagonists (LTRA), LTRA alone, theophylline or placebo. Primary outcomes were exacerbation and asthma control. The interventions evaluated were ICS (low/medium/high dose), ICS+LABA, ICS+LTRA, LTRA alone, theophylline and placebo.

**Results:**

Of the 4708 publications identified, 144 trials were eligible. Individual participant data were obtained from 29 trials and aggregate data were obtained from 19 trials. Compared with ICS Low, ICS Medium+LABA was associated with the lowest odds of exacerbation (OR 0.44, 95% credibility interval (95% CrI) 0.19–0.90) and with an increased forced expiratory volume in 1 s (mean difference 0.71, 95% CrI 0.35–1.06). Treatment with LTRA was the least preferred. No apparent differences were found for asthma control.

**Conclusions:**

Uncontrolled children/adolescents on low-dose ICS should be recommended a change to medium-dose ICS+LABA to reduce the risk for exacerbation and improve lung function.

## Tweetable abstract

**Using medium-dose inhaled corticosteroids (ICS) with long-acting β_2_-agonists reduces the odds of exacerbation and increases FEV_1_ in patients age 6–17 years whose asthma is uncontrolled on a low dose of ICS alone.** https://bit.ly/47buW6o

## Graphical abstract

**Figure:**
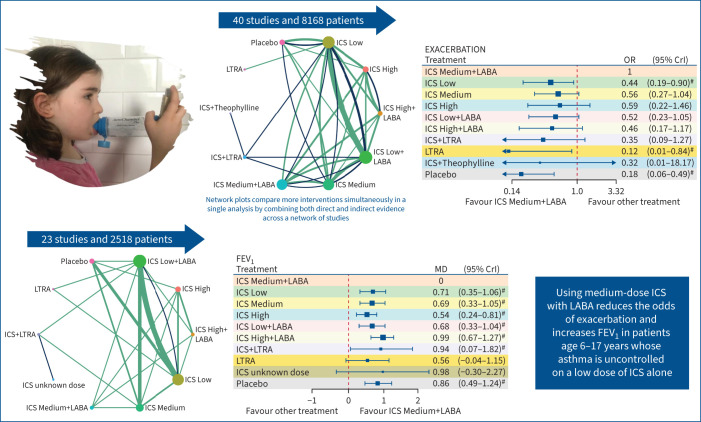
The EstablishINg the best STEp-up treatments for children with uncontrolled asthma despite INhaled corticosteroids (EINSTEIN) study. Photograph provided by the International Primary Care Respiratory Group (IPCRG) under under Creative Commons licence CC BY-NC-SA. ICS: inhaled corticosteroid; LTRA: leukotriene receptor antagonist; LABA: long-acting β_2_-agonist; OR: odds ratio; 95% CrI: 95% credibility interval; MD: mean difference; FEV_1_: forced expiratory volume in 1 s. ^#^: 95% CrIs that exclude the null value (1 or 0).

## Introduction

Asthma is the most common long-term medical condition in young people [1], and is characterised by regular wheeze, breathlessness, chest tightness and cough, with periods of relapse and remission. Asthma affects over 1 million children in the UK and 6 million in the USA. The UK National Health Service (NHS) spends around GBP 1 billion a year (2010/11 prices) treating and caring for people with asthma [2]. Asthma affects a child's quality of life by limiting daily activities such as sleep, attending school and playing sports [3, 4], and also by causing asthma exacerbations. Asthma cannot be cured, but preventer treatment is available to control symptoms and reduce risk for exacerbations in accordance with a number of guidelines [5–7]. The two British guidelines on asthma management recommend that the preferred initial preventer for children is low-dose inhaled corticosteroid (ICS) [5, 6]. In 10–15% of children, low-dose ICS does not control asthma [8] and additional treatment options include increasing the dose of ICS or adding either a long-acting β_2_-adrenoceptor agonist (LABA) or leukotriene receptor antagonist (LTRA) [5–7]. At present, guidelines recommend different options. Part of the uncertainty depends on the heterogeneity in treatment response within the population of children with asthma [9, 10].

Systematic reviews and network meta-analyses have tried to identify what the best treatment option is for children with poorly controlled asthma despite low-dose ICS treatment. A Cochrane review with 6381 children from 33 trials demonstrated that adding LABA to ICS was not associated with a significant decrease in exacerbations requiring systemic steroids [11]. In children and adolescents with mild to moderate asthma, a second Cochrane review found that combining LTRA with ICS was not associated with reducing rescue oral corticosteroids (OCS) or hospital admission compared with the same or a higher dose of ICS [12]. Two previous network meta-analyses used aggregated data from randomised clinical trials (RCTs) whose participants were children with uncontrolled asthma [13, 14]. In 2012, Van der Mark *et al.* [13] published a review with 23 trials and 4129 patients but could not present a formal network meta-analysis since outcome measures were too heterogeneous and not wholly reported. In 2015, Zhao *et al.* [14] conducted a formal network meta-analysis using data from 35 RCTs with 12 010 children, concluding that combined ICS and LABA treatments were most effective in preventing exacerbations and that medium-dose or high-dose ICS, combined ICS and LTRA, and low-dose ICS treatments seem to be equally effective [14]. Notably, the authors excluded 70 relevant RCTs because data about exacerbations or symptom-free days were not provided in trial publications, suggesting potential for outcome reporting bias if those excluded trials had selectively reported results based on the statistical significance of their findings [15].

The EstablishINg the best STEp-up treatments for children with uncontrolled asthma despite INhaled corticosteroids (EINSTEIN) study addressed the ongoing need to identify what the best treatment option is for children and adolescents with asthma whose symptoms are uncontrolled despite low-dose ICS by seeking to include published and unpublished data, using robust and unbiased methods.

## Material and methods

We conducted a systematic review and network meta-analysis using individual participant data (IPD) from RCTs supplemented with aggregate data (AgD). We also carried out pairwise meta-analyses and a network meta-regression analysis to explore potential effect modifiers. The protocol was registered PROSPERO with identifier number CRD42019127599 and has been published [16].

### Search strategy

We retrieved all trials identified (up to June 2014) in previous AgD network meta-analyses [13, 14] and Cochrane reviews [11, 12, 17–19]. We then created and applied a new search strategy, based on the previously published search strategies [11–14, 17–19] (supplementary material), to identify published and unpublished trials. An initial search was conducted covering the period between 1 July 2014 to 11 September 2019. The search was subsequently updated to 5 May 2023. The search was conducted across seven databases, one trial registry, internal pharmaceutical company trial registries and guidelines. Additional details are provided in the supplementary material. The search focused on identifying articles in the English language that included participants age <18 years. Two searches were conducted in MEDLINE to identify potential modifiers for the network meta-regression analysis (supplementary material).

### Eligibility criteria

A detailed description of trial designs, participants, and interventions and comparators is provided in the supplementary material. In brief, we included parallel and crossover RCTs of any duration and with any level of blinding, which compared at least two of the interventions of interest. RCTs had to include participants age <18 years with “uncontrolled asthma” on ICS alone, defined as such by a validated diagnostic test or the trialists.

### Outcomes and effect modifiers

The primary outcomes were 1) exacerbation (yes/no) and 2) asthma control (yes/no) (supplementary material). We defined exacerbations as “events characterised by a change from the patient's previous status” [20], mainly requiring 1) the use of OCS, 2) the need for unscheduled visits with general practitioners or at the emergency department, 3) hospitalisation or 4) when classified as exacerbation by the trial authors. We defined asthma control as “the extent to which the various manifestations of asthma have been reduced or removed by treatment” [20]. Asthma control had to be measured by a validated test, *e.g.* the Asthma Control Test (ACT) [21] or Asthma Control Questionnaire (ACQ) [22]. Secondary outcomes were forced expiratory volume in 1 s (FEV_1_), symptoms, quality of life (QoL), mortality, adverse events and hospital admissions. We evaluated a set of potential treatment effect modifiers that were informed by clinical opinion and the literature review for both the primary and secondary outcomes: age (years), sex (females *versus* males), ethnicity (not Hispanic or Latino *versus* Hispanic or Latino), eczema (present *versus* absent), eosinophilia (eosinophilic *versus* non-eosinophilic inflammatory type) and baseline asthma severity (mild, moderate or severe).

### Trial selection

Two reviewers (S. Cividini and K. Rose) independently screened and appraised all titles and abstracts, followed by full-text screening (excluded studies are listed in the supplementary material) to identify trials for inclusion by resolving disagreements by consensus or discussion with a third reviewer (S. Turner, I. Sinha or C. Tudur Smith). The inclusion of trials was not determined by the outcomes reported in publications to minimise the impact of selective outcome reporting.

### Processing IPD and data extraction

A detailed description is provided in the supplementary material.

### Risk of bias assessment

One reviewer (S. Cividini) used the Cochrane Risk of Bias tool [23] to record the risk of bias concerning: 1) randomisation method, 2) allocation concealment, 3) blinding, 4) incomplete outcome data and 5) selective reporting. The assessment was done at the trial level. Concerns were resolved through discussion with a second reviewer (C. Tudur Smith).

### Data analysis

We used fixed effect and random effects pairwise meta-analysis, network meta-analysis and network meta-regression supplemented, wherever possible, with AgD when IPD were unavailable. Pairwise and network meta-analyses were performed using both the frequentist approach and the Bayesian approach. We used odds ratio (OR) as the measure of treatment effect for binary outcomes (exacerbation, asthma control and adverse events) and mean difference (MD) as the measure of treatment effect for continuous outcomes (FEV_1_ and QoL). We used the R package multinma based on Stan to construct all plots and fit models [24]. Additional technical details of the applied methodology are available in the supplementary material and supplementary table S2. We conducted sensitivity analyses to explore the impact of the exacerbation data collection approach by excluding trials that had recorded exacerbation data only through adverse event data collection and may not have captured all events systematically. Data availability bias could impact the IPD network meta-analysis results if the availability of IPD from included trials is related to the trial results. We attempted to overcome this by including AgD wherever possible in primary analyses and explored whether results and conclusions were different in sensitivity analyses that excluded AgD. We also compared the risk of bias and the participant and trial characteristics between IPD trials and trials with no IPD, wherever possible.

### Patient and public involvement

See the supplementary material.

## Results

**FIGURE 1:**
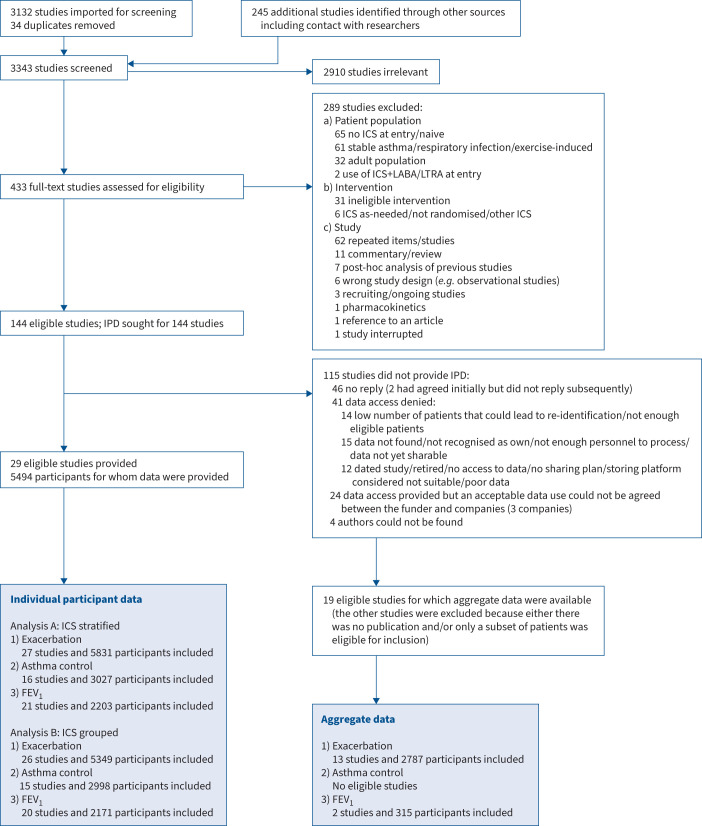
Study selection. Study search from 1 July 2014 to 11 September 2019. The flowchart also comprises the studies retrieved before July 2014 from other sources/contacts with authors. These data were used in the analysis. The update from 10 September 2019 to 5 May 2023 did not provide studies eligible for inclusion (supplementary figure S1). For the studies by Scott *et al.* [43] and Thomas [47] we used unpublished data provided by GSK and the author, respectively. The references are for conference abstracts with a no longer active link or no suitable data for inclusion. ICS: inhaled corticosteroid; LABA: long-acting β_2_-agonist; LTRA: leukotriene receptor antagonist; FEV_1_: forced expiratory volume in 1 s.

The flow diagram of the identification and inclusion of studies is shown in figure 1. In the primary search, we screened 3343 trials overall: 2910 were excluded as irrelevant and the full text was retrieved for the remaining 433 trials. We identified 144 trials as eligible for inclusion. The characteristics of included trials can be found in supplementary tables S3 and S4. 29 trials [9, 25–52] provided IPD for a total of 5494 participants. We could not retrieve the IPD for 115 trials: 24 because of issues with the data sharing agreement, 46 did not reply (two of which had initially agreed to provide data but did not reply to our following contact), 41 did not want to share data and four did not have contact details. Of the 115 eligible trials without IPD, we were able to extract AgD for at least one outcome in 19 studies [53–71]. Full details of the 96 potentially eligible trials without IPD and AgD are summarised in supplementary table S5. Of the 48 trials with IPD or AgD, 40 [25–43, 45, 46, 48–55, 58–65, 68, 71] could be included in the analysis of exacerbation outcome (39 in the ICS grouped analysis), 16 [9, 25, 26, 28, 35, 36, 39–41, 44–47, 50–52] in the analysis of asthma control outcome (15 in the ICS grouped analysis) and 23 [9, 25–30, 32, 34–37, 39–41, 43, 44, 49, 51, 52, 68, 70] in the analysis of FEV_1_ outcome (22 in the ICS grouped analysis). For the exacerbation and FEV_1_ analyses, the trial by Lötvall *et al.* [34] was split according to Global Initiative for Asthma Strategy 2019 [7] age groups to avoid the trial artificially contributing to a head-to-head comparison of ICS Low *versus* ICS Medium. One trial [51] was excluded from the analyses with grouped ICS doses as all treatments randomised were within the same treatment class and could not contribute comparative data. A stratification of the ICS+LTRA combination on ICS was not possible because of insufficient data. A repeated search strategy with a date range between 10 September 2019 and 5 May 2023 (supplementary figure S1) did not identify any new eligible studies that could impact the results. We assessed the risk of bias for 29 trials with IPD and 19 trials with AgD (supplementary table S6 and supplementary figure S2a–c). Most trials (32 trials corresponding to 67% of all studies) were considered as low risk of bias across all domains; 12 (25%) trials had one domain classed as high risk, two (4%) trials had two domains classed as high risk and two (4%) trials had three domains classed as high risk (supplementary table S6).

### Network meta-analysis

#### Exacerbation

##### ICS stratified by dose when combined with LABA (Analysis A1)

40 trials (27 IPD; 13 AgD) that randomised 8168 patients (5381 (328 events) IPD; 2787 (321 events) AgD) provided evidence for 10 treatment classes included in the random effects network meta-analysis (figure 2a and supplementary table S7). There is evidence in favour of ICS Low (OR 0.42, 95% credibility interval (95% CrI) 0.18–0.91), ICS Medium (OR 0.33, 95% CrI 0.13–0.82), ICS High (OR 0.31, 95% CrI 0.09–0.98), ICS Low+LABA (OR 0.35, 95% CrI 0.14–0.84) and ICS Medium+LABA (OR 0.18, 95% CrI 0.06–0.49) for reducing exacerbations compared with placebo (figure 3 and supplementary table S7). There is also evidence in favour of ICS Medium+LABA compared with both ICS Low (OR 0.44, 95% CrI 0.19–0.90) and LTRA (OR 0.12, 95% CrI 0.01–0.84), and to a lesser extent compared with ICS Medium (OR 0.56, 95% CrI 0.27–1.04) or ICS Low+LABA (OR 0.52, 95% CrI 0.23–1.05) (figure 3 and supplementary table S7). In support of these results the posterior ranking suggests that ICS Medium+LABA (rank median (interquartile range (IQR)) 1 (1–2)) is the most likely treatment to be best while LTRA (rank median (IQR) 9 (8–10)) and placebo (rank median (IQR) 9 (8–9)) would be least preferred (supplementary figure S3). However, there is uncertainty about the ranking of every treatment in the network as shown by the wide and overlapping intervals (supplementary figure S3). A comparison of deviance information criteria (DIC) between the network meta-analysis consistency model and the unrelated mean effects model did not suggest inconsistency in the network. Similar results and conclusions are drawn from the corresponding frequentist analyses presented in supplementary figure S4.

**FIGURE 2:**
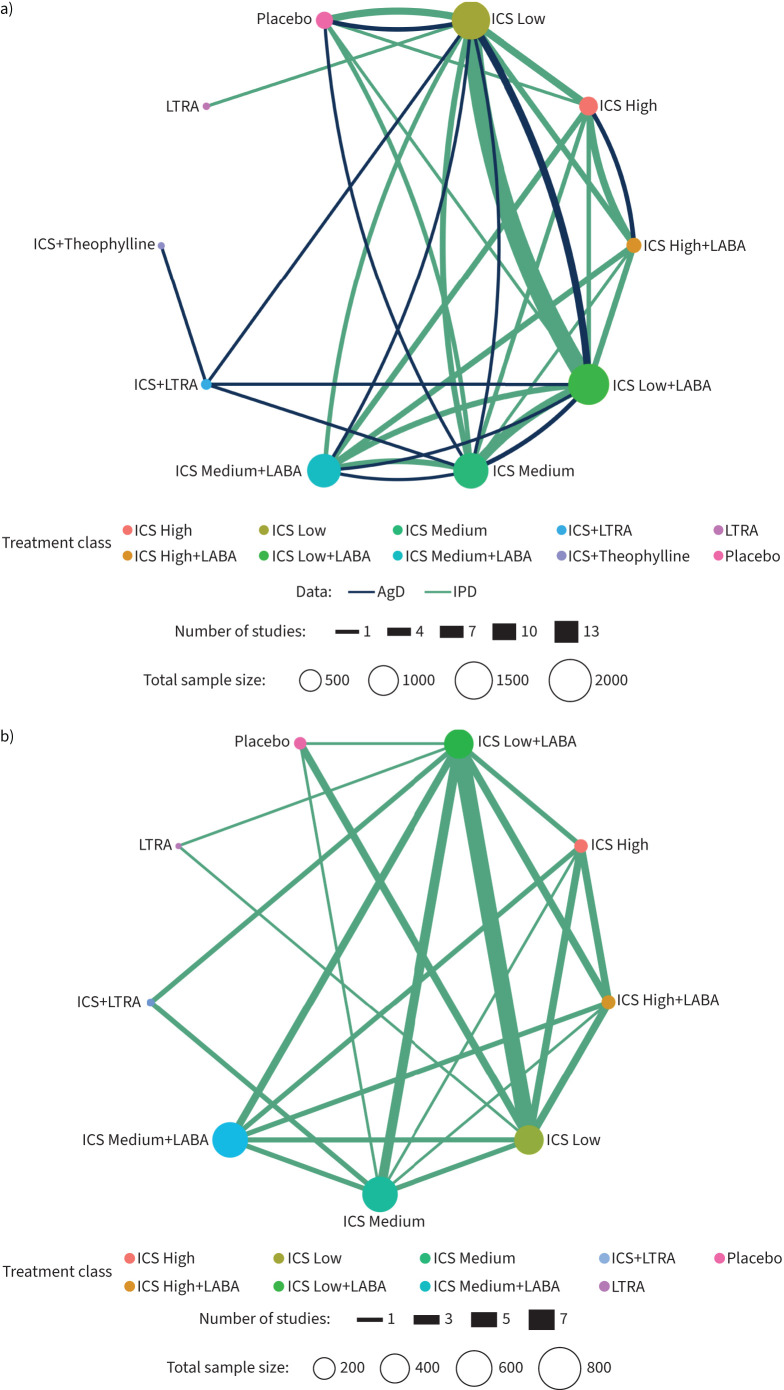


**FIGURE 2:**
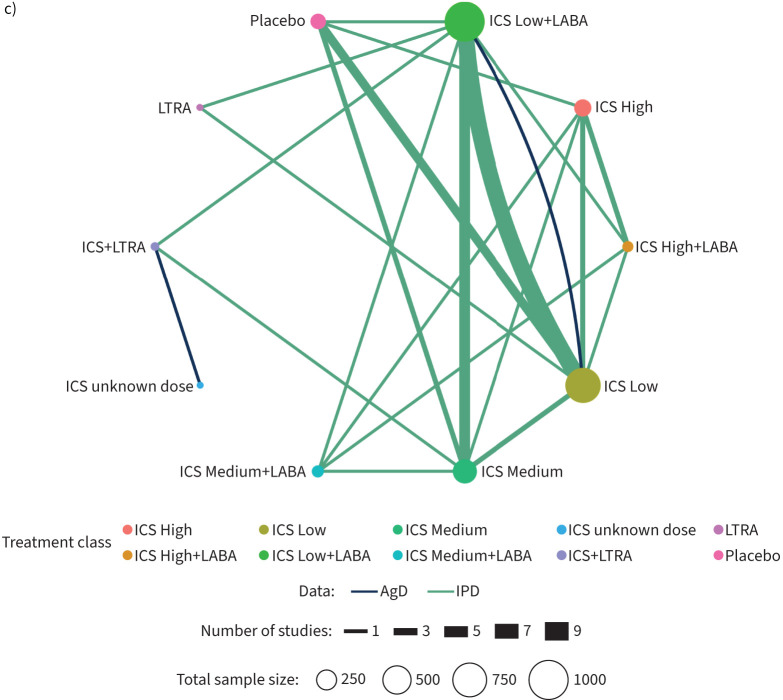
Network diagrams. a) Network plot for the random effects network meta-analysis with inhaled corticosteroid (ICS) stratified by dose when combined with long-acting β_2_-agonist (LABA) for exacerbation (Analysis A1). b) Network plot for the fixed effect network meta-analysis with ICS stratified when combined with LABA for asthma control (Analysis A2). c) Network plot for the fixed effect network meta-analysis with ICS stratified when combined with LABA for forced expiratory volume in 1 s (Analysis A3). Network plots compare more interventions simultaneously in a single analysis by combining both direct and indirect evidence across a network of studies. LTRA: leukotriene receptor antagonist; IPD: individual participant data; AgD: aggregate data.

**FIGURE 3:**
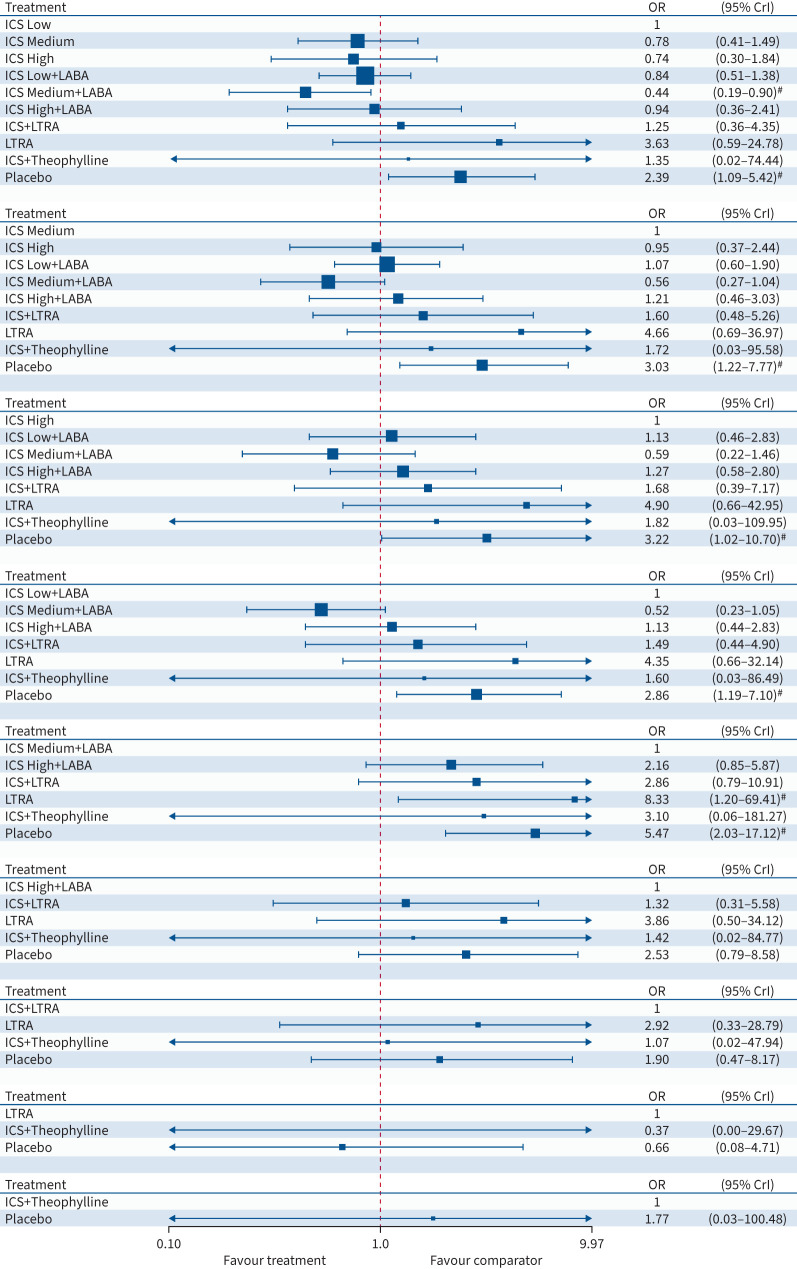
Forest plot for exacerbation. The results are from a Bayesian network meta-analysis. Squares are proportional to the weight of studies. OR: odds ratio; 95% CrI: 95% credibility interval; ICS: inhaled corticosteroid; LABA: long-acting β_2_-agonist; LTRA: leukotriene receptor antagonist. ^#^: 95% CrIs that exclude 1.

##### Additional analyses

Results for ICS grouped when combined with LABA (Analysis B1) are shown in supplementary figure S5 and supplementary table S8. Reliable estimates could not be obtained from a network meta-analysis of individual compounds due to the sparse nature of the network, with few trials and exacerbation events contributing data to particular nodes in the network. Sensitivity analyses (supplementary tables S9 and S10) were generally similar to the main analyses and further supported the conclusion that ICS Medium+LABA is the most promising of the included treatments.

##### Data availability bias

We explored the potential for data availability bias by comparing odds ratios (95% CrI) from the principal analyses, which include all available IPD and AgD (supplementary tables S7 and S8), against the corresponding sensitivity analysis excluding 13 trials (2787 participants and 321 events) with only AgD (supplementary tables S11 and S12). Where a comparison can be made, the conclusions are consistent. However, the odds ratios for comparisons against placebo are more extreme from the “IPD only” analyses (supplementary tables S11 and S12), a trend which might be expected if IPD was more likely to be provided when results were more strongly in favour of the active treatment compared with placebo. Comparing the risk of bias and trial and patient characteristics between trials that provided IPD and trials with only AgD did not ascertain any apparent differences. Assessment of risk of bias in the trials with only AgD were more often “unclear” than in the IPD trials (supplementary table S6); however, this is to be somewhat expected as additional information (*e.g.* detailed protocol) was often provided with IPD, which allowed further clarification during the assessment procedure. While we cannot rule out the possibility of data availability bias, we have tried to mitigate this risk by including both IPD and AgD in the primary analysis.

#### Asthma control

##### ICS stratified by dose when combined with LABA (Analysis A2)

**FIGURE 4:**
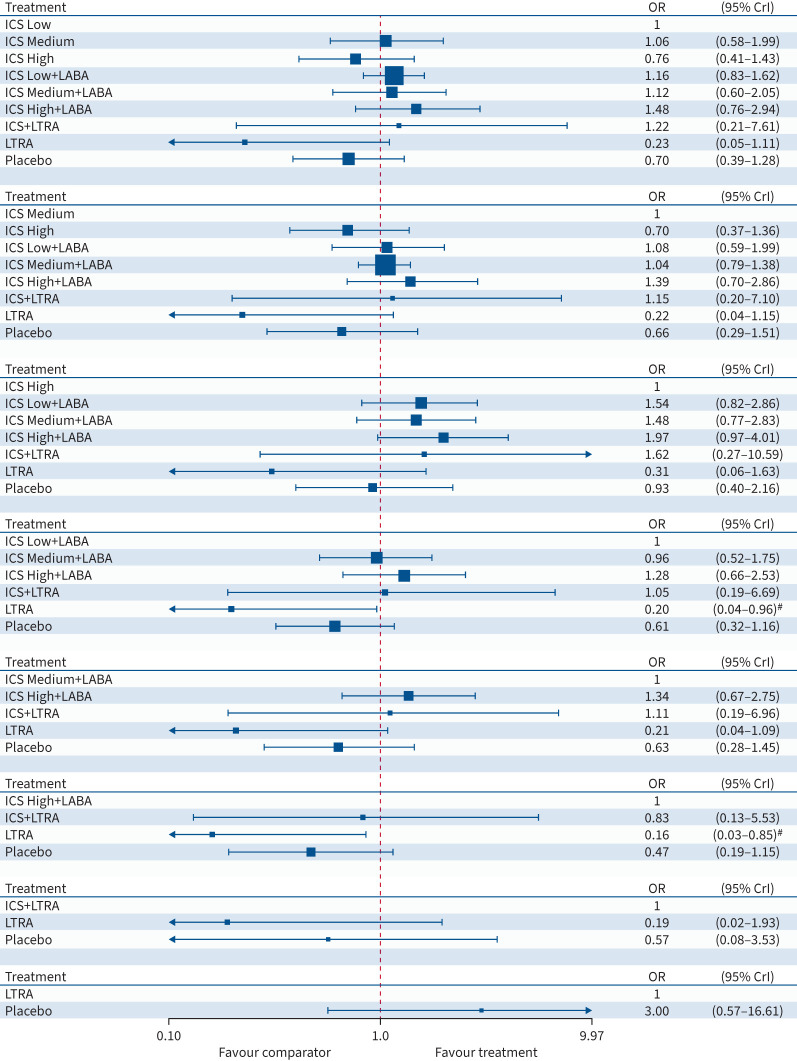
Forest plot for asthma control. The results are from a Bayesian network meta-analysis. Squares are proportional to the weight of studies. OR: odds ratio; 95% CrI: 95% credibility interval; ICS: inhaled corticosteroid; LABA: long-acting β_2_-agonist; LTRA: leukotriene receptor antagonist. ^#^: 95% CrIs that exclude 1.

16 trials provided data for nine treatment classes in the network meta-analysis (figure 2b). There were 2453 participants out of 3027 that experienced good/total asthma control at their last follow-up visit according to the ACT/ACQ tests. The fixed effect network meta-analysis (figure 4 and supplementary table S13) suggests an advantage for both ICS Low+LABA (OR 5.00, 95% CrI 1.04–25.53) and ICS High+LABA (OR 6.36, 95% CrI 1.17–35.87) when compared with LTRA. However, for all other pairwise comparisons, the 95% CrI includes values for the odds ratios that could indicate benefit for either treatment being compared, as well as both being identical. There is too much uncertainty to make any firm conclusions about preferred treatment for asthma control and this is supported by the overlapping intervals for the rank probabilities (supplementary figure S6). A comparison of DIC between the network meta-analysis consistency model and the unrelated mean effects model did not suggest inconsistency in the network. Similar results and conclusions are drawn from the corresponding frequentist analyses presented in supplementary figure S7.

##### Additional analyses

Results for ICS grouped when combined with LABA (Analysis B2) and individual compounds (Analysis C2) are shown in supplementary tables S14 and S15 and supplementary figures S8 and S9.

#### Forced expiratory volume in 1 s

##### ICS stratified by dose when combined with LABA (Analysis A3)

**FIGURE 5:**
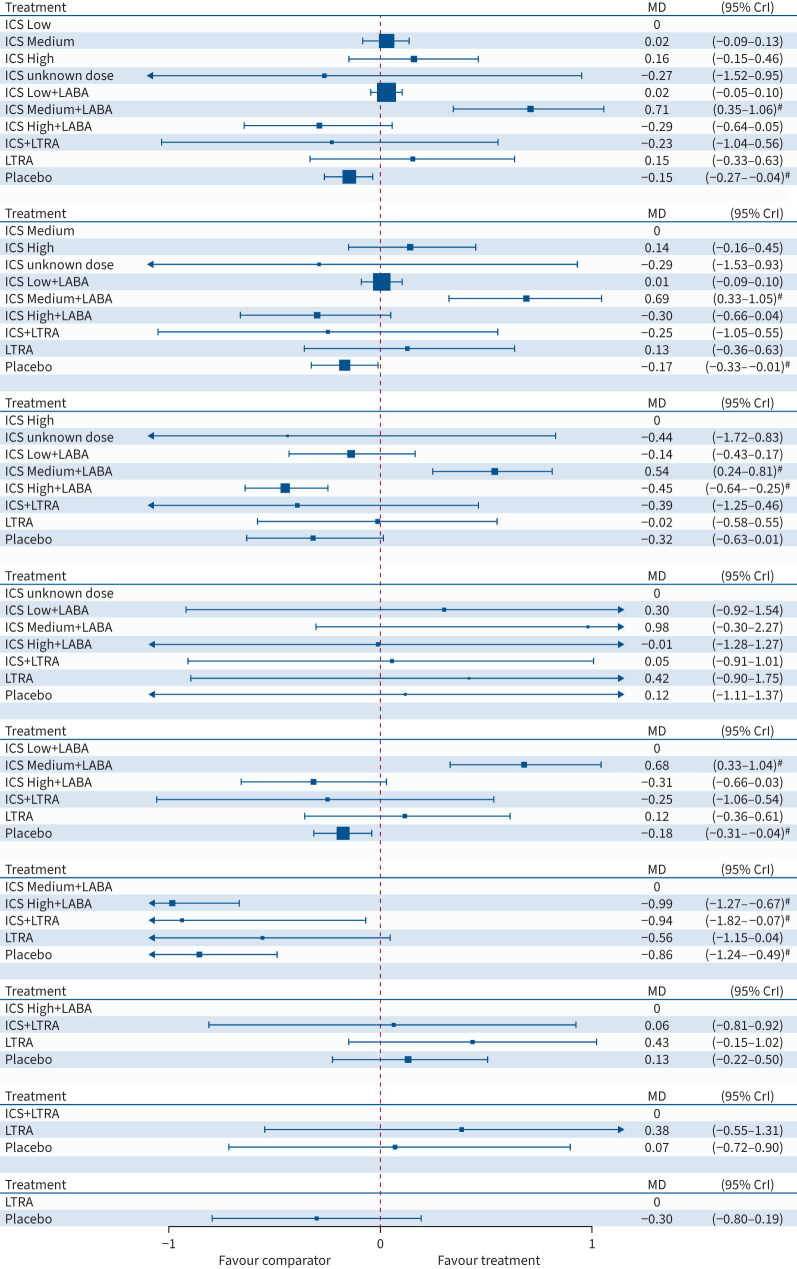
Forest plot for forced expiratory volume in 1 s. The results are from a Bayesian network meta-analysis. Squares are proportional to the weight of studies. MD: mean difference; 95% CrI: 95% credibility interval; ICS: inhaled corticosteroid; LABA: long-acting β_2_-agonist; LTRA: leukotriene receptor antagonist. ^#^: 95% CrIs that exclude 0.

23 trials (21 IPD; 2 AgD) with 2518 participants (2203 IPD; 315 AgD) provided data for 10 treatment classes included in this network (figure 2c). The mean difference (MD) from the fixed effect network meta-analysis (figure 5 and supplementary table S16) suggests that ICS Low (MD 0.15, 95% CrI 0.04–0.27), ICS Medium (MD 0.17, 95% CrI 0.01–0.33), ICS Low+LABA (MD 0.18, 95% CrI 0.04–0.31) and ICS Medium+LABA (MD 0.86, 95% CrI 0.49–1.24) are more effective than placebo. There is evidence that ICS Medium+LABA is more effective than ICS Low (MD 0.71, 95% CrI 0.35–1.06), ICS Medium (MD 0.69, 95% CrI 0.33–1.05), ICS High (MD 0.54, 95% CrI 0.24–0.81), ICS Low+LABA (MD 0.68, 95% CrI 0.33–1.04), ICS High+LABA (MD 0.99, 95% CrI 0.67–1.27) and ICS+LTRA (MD 0.94, 95% CrI 0.07–1.82) (figure 5 and supplementary table S16). There is also some evidence to suggest that ICS High is better than ICS High+LABA (MD 0.45, 95% CrI 0.25–0.64) (figure 5 and supplementary table S16). The rank probability plots (supplementary figure S10) show that ICS Medium+LABA is likely the best treatment in this network, but there is considerable uncertainty around the rank probability of other treatments. A comparison of DIC between the network meta-analysis consistency model and the unrelated mean effects model did not suggest inconsistency in the network. Similar results and conclusions are drawn from the corresponding frequentist analyses presented in supplementary figure S11.

##### Additional analyses

Results for ICS grouped when combined with LABA (Analysis B3) and individual compounds (Analysis C3) are shown in supplementary tables S17 and S18 and supplementary figures S12 and S13.

### Further secondary outcomes

There were no deaths recorded in any of the included trials. The “symptoms” outcome was not analysed as it can be challenging to interpret isolated symptoms, *e.g.* coughing at night without needing reliever medication, missing school and not wheezing when running around. The decision to abandon the analysis of this outcome was not influenced by any results or other investigations completed. 11 trials measured the “QoL” outcome using two questionnaires: 1) Asthma Quality of Life Questionnaire (32 items; developed for use in adults age 17–70 years) [21] and 2) Paediatric Asthma Quality of Life Questionnaire (23 items; developed for use in children age 7–17 years) [22]. There was insufficient data for a reliable network meta-analysis and limited pairwise meta-analyses (supplementary table S19) did not suggest clinically important differences in QoL. Data for “hospital admissions” caused by an asthma exacerbation were only available from five trials with IPD, with percentage admission ranging from 0.5% to 2.7% of participants (supplementary table S20). There was considerable heterogeneity in the recording and coding of adverse events data across trials. We summarised the numerical results and conducted frequentist pairwise meta-analyses using IPD and AgD, where more than one trial recorded the same adverse event: infections/infestations, neurological disorders, oral candidiasis, pneumonia, cardiac disorders, clinically significant ECG changes (favourable and unfavourable) and heart rate (MD at the last visit *versus* baseline) (supplementary figures S14–S22). There is insufficient evidence to conclude that the odds of any of these adverse events differ between the treatment classes that could be compared except for neurological disorders, suggesting lower odds of neurological disorders (graded as mild or moderate) on ICS+LABA compared with ICS+LTRA (OR 0.09, 95% confidence interval (CI) 0.01–0.82; one trial) and greater odds for ICS Medium compared with placebo (OR 4.8, 95% CI 1.12–20.60; three trials).

### Effect modification

We compared the DIC between network meta-regression models with and without interaction terms. We found no overall evidence of interactions in any models for exacerbation, asthma control and FEV_1_ (supplementary tables S21, S25 and S27). However, some models had non-zero interaction regression coefficients (supplementary tables S22 and S28) for exacerbation and FEV_1_. Still, these results should be viewed cautiously due to the few patients included. Furthermore, as recommendations regarding the treatment and care of patients do not differ according to any of the studied covariates (supplementary tables S23, S24, S29 and S30), and the interactions were not consistently identified as non-zero across all outcomes, we conclude there is insufficient evidence for effect modification based on this data.

## Discussion

### Principal findings

The network meta-analysis results suggest that for a child with uncontrolled asthma despite ICS treatment, the odds of an exacerbation are reduced by stepping up to medium-dose ICS in combination with LABA compared with low-dose ICS. Objective testing with lung function demonstrated that medium-dose ICS plus LABA was superior compared with any dose of ICS without LABA and low-dose ICS plus LABA. Low or high doses of ICS combined with LABA were associated with increased odds of good asthma control but only *versus* LTRA monotherapy. Across the trials there were no deaths, relatively few hospitalisation admissions due to asthma and adverse events were uncommon.

### Strengths and limitations of the study

To the best of our knowledge, this is the first network meta-analysis of studies in children and adolescents with asthma using IPD. The network meta-analysis approach with IPD enabled us to include direct and indirect evidence comparing different treatments and dose levels, which have not been compared against each other in previous RCTs or network meta-analyses. We did not manage to retrieve and include data from 96 potentially eligible trials (67% of the eligible trials on this question); this may have introduced bias. Due to a scarcity of RCTs conducted on theophylline, we had minimal data for ICS+Theophylline and insufficient data to stratify ICS dose when combined with LTRA; therefore, uncertainty remains about these treatments. Furthermore, several of the credible intervals from the network meta-analyses are wide and include clinically important values indicating that further differences or robust conclusions about the equivalence between treatments may be identified with additional data. Due to sparse data, we could not carry out time-to-event analyses. Diagnosing asthma can be more uncertain in younger children since they can comply less with lung function testing. However, few children under age 6 years were included in our analysis, meaning that imprecision in asthma diagnosis between studies was not substantially affected by the inclusion of younger children. There are two aspects of childhood asthma management that we could not consider in this review: 1) the role of maintenance and reliever therapy (MART) (there is only one publication) and symptom-driven approaches to using ICS, and 2) long-term or rare side-effects of treatments. We were not able to explore the impact of inhaler technique or adherence.

### Comparison with other studies

In 2012, Van der Mark *et al*. [13] attempted a similar approach but could not synthesise results due to variations in the measurement and reporting of outcomes; they concluded that ranking of effectiveness was not possible. In 2015, the network meta-analysis by Zhao *et al.* [14] suggested that combining ICS (dose not specified) and LABA treatments was most effective in preventing exacerbations. They also reported that there was a little difference between continuing low-dose ICS, increasing the ICS dose to the medium-dose or high-dose range or combining ICS with LTRA. However, they could not make recommendations about the dose of ICS when combined with LABA. Using IPD where available, our approach enabled us to analyse the data more robustly, identify more relevant dose-specific differences between treatments that were previously not evident and explore the potential for treatment effect modification.

### Implications for clinicians and policymakers and future research

The current recommendation for treating children and adolescents with asthma who are not well-controlled on ICS is to check adherence, inhaler technique and comorbidities first, then consider a “step-up” to their treatment by increasing the dose of ICS or adding another therapy. The 2019 GINA guideline [7] recommends the preferred controller for children age 6–11 years is “medium-dose ICS” or “low-dose ICS with LABA”, which have similar benefits. However, the EINSTEIN analysis suggests that the preferred first “step-up” option should be to increase the dose of ICS to a medium dose in combination with LABA, as this has the most beneficial effect on exacerbation prevention and improves asthma control and lung function. The parents we consulted supported the recommendation of medium-dose ICS with LABA, preferring to avoid trying alternative “small-step” treatment adjustments, which could put children at an increased risk of exacerbation and hospital admission for a more extended period. A future update of the review is needed to incorporate additional IPD, ensure maximum representation of treatments within the network meta-analysis and make a reliable recommendation regarding specific formulations.

### Conclusions

Although more included patients would have led to more precise estimates, we can reasonably conclude that medium-dose ICS with LABA would be recommended for children and adolescents with asthma who are uncontrolled on a low dose of ICS. Although there was insufficient data to infer whether LTRA monotherapy was superior to ICS monotherapy, no guideline currently recommends LTRA monotherapy over ICS monotherapy.

Results from the EINSTEIN study will provide clinicians and patients with accessible, high-quality, patient-relevant information to help make evidence-informed treatment choices. Earlier identification of the best step-up treatment for a particular child could significantly impact children's lives with more extensive benefits to society and the NHS.

## Supplementary material

10.1183/13993003.01011-2023.Supp1 **Please note:** supplementary material is not edited by the Editorial Office, and is uploaded as it has been supplied by the author.
Supplementary methods, tables and figures ERJ-01011-2023.SUPPLEMENT
Supplementary material: excluded studies ERJ-01011-2023.SUPPLEMENT2
Supplementary material: excluded studies ERJ-01011-2023.SUPPLEMENT3

## Shareable PDF

10.1183/13993003.01011-2023.Shareable1 This one-page PDF can be shared freely online.
Shareable PDF ERJ-01011-2023.Shareable
