# Supplementary material for: Best step-up treatments for children with uncontrolled asthma: a systematic review and network meta-analysis of individual participant data
Source: Eur Respir J. 2023 Dec 21;62(6):2301011. doi: 10.1183/13993003.01011-2023 (PMC10752294; doi:10.1183/13993003.01011-2023)
Supplement: Supplementary file 1 [file ERJ-01011-2023.SUPPLEMENT.pdf]

Supplement 1

Best step-up treatments for children with uncontrolled asthma: A systematic review and network meta-analysis of individual participant data

Sofia Cividini, MSc; Ian Sinha, PhD; Sarah Donegan, PhD; Michelle Maden, PhD; Katie Rose, MBChB; Olivia Fulton; Giovanna Culeddu, MSc; Dyfrig A. Hughes, PhD; Stephen Turner, MD; Catrin Tudur Smith, PhD on behalf of the EINSTEIN collaborative group

Methods S1. Search strategy; for example, MEDLINE (OVID) search
Methods S2. Modifiers searches 1 − Database: Ovid MEDLINE(R) ALL <1946 to July 02, 2019>
Methods S3. Modifiers searches 2 − Database: Ovid MEDLINE(R) ALL <1946 to July 02, 2019>
Methods S4. Eligibility criteria
Methods S5. Outcomes
Methods S6. Processing individual participant data and data extraction
Methods S7. Data analysis
Methods S8. Patient and public involvement
Table S1. Estimated clinical comparability daily doses (µg) of Inhaled Corticosteroids
Table S2. Prior distributions used in Bayesian NMA and ML-NMR models
Table S3. Characteristics of the included studies with individual participant data (parts 1 to 6)
Table S4. Characteristics of the included studies with aggregate data (parts 1 to 4)
Table S5. Eligible studies without individual participant data or aggregate data (parts 1 to 18)
Table S6. Risk of bias for included studies with individual participant data or aggregate data (parts 1 to 5)
Table S7. Exacerbation Bayesian random-effects network meta-analysis (ORa, 95% CrI) with IPD and AgD (Analysis A1: 40 trials, 8168 participants, 649 events)
Table S8. Bayesian fixed effect network meta-analysis results (IPD And AgD) for exacerbations. ICS grouped with LABA – Analysis B1
Table S9. Sensitivity analysis excluding exacerbation events identified from adverse event data: Bayesian random-effects network meta-analysis results (IPD and AgD) for exacerbations. ICS stratified by dose when combined with LABA – Analysis A1
Table S10. Sensitivity analysis excluding exacerbation events identified from adverse event data: Bayesian fixed effect network meta-analysis results (IPD and AgD) for the exacerbation outcome. ICS grouped when combined with LABA – Analysis B1
Table S11. Sensitivity analysis to explore data availability bias: Bayesian fixed effect network meta-analysis results for exacerbations. ICS stratified by dose when combined with LABA (IPD trials only, i.e., excluding trials with AgD only) – Analysis A1
Table S12. Sensitivity analysis to explore data availability bias: Bayesian fixed effect network meta-analysis results for the exacerbation outcome (including ICS grouped when combined with LABA). IPD trials only (i.e., excluding trials with AgD only) – Analysis B1
Table S13. Asthma Control Bayesian fixed effect network meta-analysis (ORa, 95% CrI) with IPD (Analysis A2: 16 trials, 3027 participants, 2453 events)
Table S14. Bayesian fixed effect network meta-analysis (IPD only) for asthma control. ICS grouped when combined with LABA – Analysis B2
Table S15. Bayesian random-effects network meta-analysis (IPD only) for asthma control (individual compounds) – Analysis C2
Table S16. FEV1 Bayesian fixed effect network meta-analysis (MDa, 95% CrI) with IPD and AgD (Analysis A3: 23 trials, 2518 participants)
Table S17. Bayesian random-effects network meta-analysis (IPD and AgD) for FEV1. ICS grouped when combined with LABA – Analysis B3
Table S18. Bayesian fixed effect network meta-analysis (IPD only) for FEV1 (individual compounds) – Analysis C3
Table S19. Direct pairwise comparisons of treatment classes (IPD and AgD) for quality of life outcome
Table S20. Hospital admissions
Table S21. Model comparison assessments from network meta-analysis models including interactions for the outcome exacerbation
Table S22. Parameter estimates (Posterior mean [95% CrI]) from NMR models including interactions for the outcome exacerbation

Table S23. Odds ratios (95% CrI) from fixed effect NMR with "treatment by ethnicity" interactions for the outcome exacerbation
Table S24. Odds ratios (95% CrI) from fixed effect NMR with "treatment by baseline severity" interactions for the outcome exacerbation
Table S25. Model comparison assessments from network meta-analysis models including interactions for the outcome asthma control
Table S26. Parameter estimates (Posterior mean [95% CrI]) from NMR models including interactions for the outcome asthma control
Table S27. Model comparison assessments from network meta-analysis models including interactions for the outcome FEV_1
Table S28. Parameter estimates (Posterior mean [95% CrI]) from NMR models including interactions for the outcome FEV_1
Table S29. Mean difference (95% CrI) from random- effects NMR with "treatment by sex" interactions for the outcome FEV_1
Table S30. Mean difference (95% CrI) from fixed effect NMR with "treatment by eosinophilia" interactions for the outcome FEV_1
Figure S1. Secondary flowchart
Figure S2A. Comparison-adjusted funnel plots (exacerbation frequentist random-effects network meta-analysis)
Figure S2B. Comparison-adjusted funnel plots (asthma control frequentist fixed-effect network meta-analysis)
Figure S2C. Comparison-adjusted funnel plots (FEV_1 frequentist fixed-effect network meta-analysis)
Figure S3. Rankings for the random-effects network meta-analysis (ICS stratified by dose when combined with LABA) for exacerbations – Analysis A1
Figure S4 (parts 1 to 3). Exacerbation frequentist random-effects network meta-analysis (OR, 95% Cr) with IPD and AgD (Analysis A1: 40 trials, 8168 participants, 649 events)
Figure S5. Network plot and rankings for the fixed effect network meta-analysis (ICS grouped when combined with LABA) for exacerbations – Analysis B1
Figure S6. Network plot and rankings for the fixed effect network meta-analysis (ICS stratified when combined with LABA) for asthma control – Analysis A2
Figure S7 (parts 1 to 3). Asthma Control frequentist fixed effect network meta-analysis (OR, 95% Cr) with IPD (Analysis A2: 16 trials, 3027 participants, 2453 events)
Figure S8. Network plot and rankings for the fixed effect network meta-analysis (ICS grouped when combined with LABA) for asthma control – Analysis B2
Figure S9. Network plot and rankings for the random-effects network meta-analysis (individual compounds) for asthma control – Analysis C2
Figure S10. Network plot and rankings for the fixed effect network meta-analysis (ICS stratified when combined with LABA) for FEV_1 – Analysis A3
Figure S11 (parts 1 to 3). FEV_1 frequentist fixed effect network meta-analysis (MD, 95% CI) with IPD and AgD (Analysis A3: 23 trials, 2518 participants)
Figure S12. Network plot and rankings for the random-effects network-meta-analysis (ICS grouped when combined with LABA) for FEV_1 – Analysis B3
Figure S13. Network plot and rankings for the fixed effect network meta-analysis (individual compounds) for FEV_1 – Analysis C3
Figure S14. Oral candidiasis (ICS dose stratified)
Figure S15. Oral candidiasis (any ICS dose combined with LABA)
Figure S16. Cardiac disorders (ICS dose grouped)
Figure S17. Clinically significant electrocardiogram (ECG) favorable changes (ICS dose grouped)
Figure S18. Clinically significant electrocardiogram (ECG) unfavorable changes (ICS dose grouped)
Figure S19. Heart rate (HR) change (last visit vs baseline) (ICS dose grouped)
Figure S20. (part 1). Infections and infestations (ICS dose grouped)
Figure S20. (part 2). Infections and infestations (ICS dose grouped)
Figure S20. (part 3). Infections and infestations (ICS dose grouped)
Figure S21. (part 1). Neurological disorders (ICS dose grouped)
Figure S21. (part 2). Neurological disorders (ICS dose grouped)
Figure S22. Pneumonia (ICS dose grouped)

Methods S1. Search strategy; for example, MEDLINE (OVID) search

We searched MEDLINE, the Cochrane Database of Systematic Reviews (CDSR), the Cochrane Central Register of Controlled Trials (CENTRAL), Embase, Web of Science (all databases), National Institute for Health and Care Excellence (NICE) Technology Appraisals, and the National Institute for Health Research (NIHR) Health Technology Assessment (HTA) series using relevant search terms. The reference list of included trials and relevant reviews, along with the reference lists of existing clinical guidelines such as the British Thoracic Society (BTS) Guideline [1, 2] and Global Initiative for Asthma (GINA), [3] were also scanned. Unpublished trials were located by searching across a range of clinical trial registries included within the World Health Organization (WHO) International Clinical Trials Registry Platform search portal (including clinicaltrials.gov and the International Traditional Medicine Clinical Trial Registry) and conference abstracts (e.g., European Respiratory Society; American Thoracic Society). We also searched internal clinical trial registers for pharmaceutical companies that manufacture health technologies of interest (e.g., GSK, AstraZeneca, Novartis, Merck). Selection and screening of studies were carried out using Covidence and Rayyan.

1 exp Asthma/

2 asthma.ti,ab.

3 1 or 2

4 exp Infant/

5 infant*.ti,ab.

6 infancy.ti,ab.

7 newborn*.ti,ab.

8 baby*.ti,ab.

9 babies.ti,ab.

10 neonat*.ti,ab.

11 preterm*.ti,ab.

12 prematur*.ti,ab.

13 postmatur*.ti,ab.

14 exp child/

15 child*.ti,ab.

16 schoolchild*.ti,ab.

17 "school age*".ti,ab.

18 preschool*.ti,ab.

19 kid.ti,ab.

20 kids.ti,ab.

21 toddler*.ti,ab.

22 exp Adolescent/

23 adoles*.ti,ab.

24 teen*.ti,ab.

25 boy*.ti,ab.

26 girl*.ti,ab.

27 exp Minors/

28 minor*.ti,ab.

29 exp Puberty/

30 pubert*.ti,ab.

31 pubescen*.ti,ab.

32 prepubescen*.ti,ab.

33 exp Pediatrics/

34 paediatric*.ti,ab.

35 pediatric*.ti,ab.

36 exp Schools/

37 "nursery school*".ti,ab.

38 kindergar*.ti,ab.

39 "primary school*".ti,ab.

40 "secondary school*".ti,ab.

41 "elementary school*".ti,ab.

42 "high school*".ti,ab.

43 highschool*.ti,ab.

44 or/4-43

45 "inhaled corticosteroid*".mp.

46 ICS.mp.

47 exp Beclomethasone/

48 beclomethasone.mp.

49 "beclomethasone dipropionate".mp.

50 becotide.mp.

51 clenil.mp.

52 ciclesonide.mp.

53 "clenil modulite".mp.

54 exp Fluticasone/

55 "fluticasone propionate".mp.

56 fluticasone.mp.

57 flixotide.mp.

58 exp Budesonide/

59 budesonide.mp.

60 Mometasone Furoate/

61 mometasone.mp.

62 exp Adrenergic beta-Agonists/

63 "long acting beta-2 agonist*".mp.

64 "long acting beta2 agonist*".mp.

65 LABA.mp.

66 exp Formoterol Fumarate/

67 formoterol.mp.

68 Oxis.mp.

69 "fluticasone furoate".mp.

70 exp Salmeterol Xinafoate/

71 salmeterol.mp.

72 serevent.mp.

73 vilanterol.mp.

74 exp Leukotriene Antagonists/

75 "leukotriene receptor antagonist*".mp.

76 LTRA.mp.

77 zafirlukast.mp.

78 montelukast.mp.

79 exp Theophylline/

80 theophylline.mp.

81 Tiotropium.mp.

82 spiriva.mp.

83 Symbicort.mp.

84 Seretide.mp.

85 flutiform.mp.

86 relvar.mp.

87 or/45-86

88 Clinical Trial.pt.

89 Randomized Controlled Trial.pt.

90 exp Random Allocation/

91 exp Single-Blind Method/

92 exp Double-Blind Method/

93 exp Cross-Over Studies/

94 exp Placebos/

95 RCT.ti,ab.

96 Random*.ti,ab.

97 "Single blind*".ti,ab.

98 "Double blind*".ti,ab.

99 "triple blind*".ti,ab.

100 placebo*.ti,ab.

101 or/88-100

102 3 and 44 and 87 and 101

103 limit 102 to ed=20140701-20190911

104 limit 103 to english language

105 (case reports or editorial or letter).pt.

106 4 not 105

Methods S2. Modifiers searches 1 − Database: Ovid MEDLINE(R) ALL <1946 to July 02, 2019>

To identify potential modifiers for the network meta-regression analysis, a search was first conducted in MEDLINE combining four concepts; asthma terms AND child terms AND ICS terms AND modifier terms.

1 exp Asthma/

2 asthma.ti,ab.

3 1 or 2

4 exp Infant/

5 infant*.ti,ab.

6 infancy.ti,ab.

7 newborn*.ti,ab.

8 baby*.ti,ab.

9 babies.ti,ab.

10 neonat*.ti,ab.

11 preterm*.ti,ab.

12 prematur*.ti,ab.

13 postmatur*.ti,ab.

14 exp child/

15 child*.ti,ab.

16 schoolchild*.ti,ab.

17 "school age*".ti,ab.

18 preschool*.ti,ab.

19 kid.ti,ab.

20 kids.ti,ab.

21 toddler*.ti,ab.

22 exp Adolescent/

23 adolescen*.ti,ab.

24 teen*.ti,ab.

25 boy*.ti,ab.

26 girl*.ti,ab.

27 exp Minors/

28 minor*.ti,ab.

29 exp Puberty/

30 pubert*.ti,ab.

31 pubescen*.ti,ab.

32 prepubescen*.ti,ab.

33 exp Pediatrics/

34 paediatric*.ti,ab.

35 pediatric*.ti,ab.

36 exp Schools/

37 "nursery school*".ti,ab.

38 kindergar*.ti,ab.

39 "primary school*".ti,ab.

40 "secondary school*".ti,ab.

41 "elementary school*".ti,ab.

42 "high school*".ti,ab.

43 highschool*.ti,ab.

44 4 or 5 or 6 or 7 or 8 or 9 or 10 or 11 or 12 or 13 or 14 or 15 or 16 or 17 or 18 or 19 or 20 or 21 or 22 or 23 or 24 or 25 or 26 or 27 or 28 or 29 or 30 or 31 or 32 or 33 or 34 or 35 or 36 or 37 or 38 or 39 or 40 or 41 or 42 or 43

45 3 and 44

46 "inhaled corticosteroid*".ti,ab,kw.

47 exp Beclomethasone/

48 "beclomethasone dipropionate".ti,ab,kw.

49 ciclesonide.ti,ab,kw.

50 exp Fluticasone/

51 "fluticasone propionate".ti,ab,kw.

52 exp Budesonide/

53 budesonide.ti,ab,kw.

54 Mometasone Furoate/

55 mometasone.ti,ab,kw.

56 exp Adrenal Cortex Hormones/ or exp Adrenergic beta-Agonists/

57 "long acting beta-2 agonist*".ti,ab,kw.

58 "long acting beta2 agonist*".ti,ab,kw.

59 exp Formoterol Fumarate/

60 formoterol.ti,ab,kw.

61 exp Salmeterol Xinafoate/

62 salmeterol.ti,ab,kw.

63 vilanterol.ti,ab,kw.

64 exp Leukotriene Antagonists/

65 "leukotriene receptor antagonist*".ti,ab,kw.

66 zafirlukast.ti,ab,kw.

67 montelukast.ti,ab,kw.

68 exp Theophylline/

69 theophylline.ti,ab,kw.

70 Tiotropium.ti,ab,kw.

71 46 or 47 or 48 or 49 or 50 or 51 or 52 or 53 or 54 or 55 or 56 or 57 or 58 or 59 or 60 or 61 or 62 or 63 or 64 or 65 or 66 or 67 or 68 or 69 or 70

72 45 and 71

73 modifi*.ti,ab,kw.

74 72 and 73

75 ((age or gender or ethnicity or eczema or asthma severity) adj3 (outcome* or effect* or modif* or success* or response or differen*)).mp.

76 72 and 75

77 ((age or gender or ethnic* or racial or eczema or asthma severity) and (effect* or differen* or modif* or success* or response or outcome*)).ti.

78 72 and 77

79 74 or 76 or 78

80 limit 79 to english language

Methods S3. Modifiers searches 2 − Database: Ovid MEDLINE(R) ALL <1946 to July 02, 2019>

As modifier details may not be identified from titles and abstracts, a second MEDLINE search was then conducted on the following concepts; asthma terms AND child terms AND ICS terms AND limit to RCTs. All results from this search were then imported into an Endnote Library and the full text for all RCTs were obtained. A full text search of the PDF files was then undertaken on the following terms; modifier*, modified, differential effect, predictor*, stratified, subgroup analysis.

1 exp Asthma/

2 asthma.ti,ab.

3 1 or 2

4 exp Infant/

5 infant*.ti,ab.

6 infancy.ti,ab.

7 newborn*.ti,ab.

8 baby*.ti,ab.

9 babies.ti,ab.

10 neonat*.ti,ab.

11 preterm*.ti,ab.

12 prematur*.ti,ab.

13 postmatur*.ti,ab.

14 exp child/

15 child*.ti,ab.

16 schoolchild*.ti,ab.

17 "school age*".ti,ab.

18 preschool*.ti,ab.

19 kid.ti,ab.

20 kids.ti,ab.

21 toddler*.ti,ab.

22 exp Adolescent/

23 adolescen*.ti,ab.

24 teen*.ti,ab.

25 boy*.ti,ab.

26 girl*.ti,ab.

27 exp Minors/

28 minor*.ti,ab.

29 exp Puberty/

30 pubert*.ti,ab.

31 pubescen*.ti,ab.

32 prepubescen*.ti,ab.

33 exp Pediatrics/

34 paediatric*.ti,ab.

35 pediatric*.ti,ab.

36 exp Schools/

37 "nursery school*".ti,ab.

38 kindergar*.ti,ab.

39 "primary school*".ti,ab.

40 "secondary school*".ti,ab.

41 "elementary school*".ti,ab.

42 "high school*".ti,ab.

43 highschool*.ti,ab.

44 4 or 5 or 6 or 7 or 8 or 9 or 10 or 11 or 12 or 13 or 14 or 15 or 16 or 17 or 18 or 19 or 20 or 21 or 22 or 23 or 24 or 25 or 26 or 27 or 28 or 29 or 30 or 31 or 32 or 33 or 34 or 35 or 36 or 37 or 38 or 39 or 40 or 41 or 42 or 43

45 3 and 44

46 "inhaled corticosteroid*".ti,ab,kw.

47 exp Beclomethasone/

48 "beclomethasone dipropionate".ti,ab,kw.

49 ciclesonide.ti,ab,kw.

50 exp Fluticasone/

51 "fluticasone propionate".ti,ab,kw.

52 exp Budesonide/

53 budesonide.ti,ab,kw.

54 Mometasone Furoate/

55 mometasone.ti,ab,kw.

56 exp Adrenal Cortex Hormones/ or exp Adrenergic beta-Agonists/

57 "long acting beta-2 agonist*".ti,ab,kw.

58 "long acting beta2 agonist*".ti,ab,kw.

59 exp Formoterol Fumarate/

60 formoterol.ti,ab,kw.

61 exp Salmeterol Xinafoate/

62 salmeterol.ti,ab,kw.

63 vilanterol.ti,ab,kw.

64 exp Leukotriene Antagonists/

65 "leukotriene receptor antagonist*".ti,ab,kw.

66 zafirlukast.ti,ab,kw.

67 montelukast.ti,ab,kw.

68 exp Theophylline/

69 theophylline.ti,ab,kw.

70 Tiotropium.ti,ab,kw.

71 46 or 47 or 48 or 49 or 50 or 51 or 52 or 53 or 54 or 55 or 56 or 57 or 58 or 59 or 60 or 61 or 62 or 63 or 64 or 65 or 66 or 67 or 68 or 69 or 70

72 45 and 71

73 limit 72 to english language and randomized controlled trials.pt

Methods S4. Eligibility criteria

Trial design

We included parallel and crossover RCTs of any duration and with any level of blinding, which compared at least one of the health technologies of interest. All trials meeting our inclusion criteria were included irrespective of the outcomes reported in the publications to reduce the potential for outcome reporting bias.

Participants

We aimed to include children/adolescents (<18 years) with poor asthma control of any ethnicity and on any dose of ICS alone at the screening visit as defined by the trial protocol.

Interventions and comparators

Trials had to include a direct head-to-head comparison of at least two of the following interventions, alone or in combination with each other (where applicable), compared against each other or against a placebo:

▪ Inhaled Corticosteroids (ICSs) – beclomethasone dipropionate (BDP); ciclesonide (CIC); fluticasone propionate (FP); fluticasone furoate (FF); budesonide (BUD); mometasone furoate (MF).

▪ Long-acting β_2-agonists (LABAs) – formoterol (FORM); salmeterol (SAL); vilanterol (VI).

▪ Leukotriene receptor antagonists (LTRAs) – zafirlukast; montelukast.

▪ Theophylline.

We considered any dose of preventer treatment – inhaled or oral – and any inhaler devices used for administration. We compared patient outcomes at the level of the following treatment classes: a) ICS , b) LABA (combined with ICS), c) LTRA (as monotherapy or with ICS), d) theophylline, and e) placebo. We distinguished among low, medium, and high doses (Table S1) for the ICS class according to the GINA 2019 definitions. [3] We applied the dosage of the age class ‘6-11 years’ for the age class ‘≤5 years’, which was undefined in the GINA guideline. We performed three different levels of analysis by considering (A) ICS stratified as low, medium, and high doses when in combination with LABA, (B) all ICS doses combined, and (C) with different ICS, LABA, and LTRA molecules regardless of doses.

Methods S5. Outcomes

Categorisation of the primary outcome "asthma control".

Test	Total score	Asthma control
**ACT 4-11 (years)**	score ≤19<br>score = 20–27	0 = poor control<br>1 = good/total control
**ACT 12+ (years)**	score ≤19<br>score = 20–25	0 = poor control<br>1 = good/total control
**ACQ**	score >1<br>score ≤1	0 = poor control<br>1 = good/total control
**Others**	to be evaluated on an individual case by case basis	0 = poor control<br>1= good/total control

Methods S6. Processing individual participant data and data extraction

We approached the sponsor or the corresponding author of each eligible trial via email or a dedicated portal for data sharing (e.g., Clinical Study Data Request - CSDR), requesting anonymized individual participant data, metadata, and relevant documentation. [4] We conducted a range of standard quality and consistency checks of the data, cross-checking the re-analysed IPD against previously published results to highlight inconsistencies or possible errors. We created a new dataset for every included trial using a pre-specified variable dictionary to ensure a standardised approach across all trials. One reviewer (SC) extracted trial-level data, and a second reviewer (CTS) checked for consistency. For eligible trials without IPD, we abstracted suitable aggregate outcome and treatment effect modifier data to allow inclusion in analyses wherever possible. Discrepancies were resolved through a consensus procedure.

Methods S7. Data analysis

A logit link function was used for binary outcomes, and an identity link function for normally distributed continuous outcomes. All network meta-regression models used independent interactions between treatment and covariate, and all NMR models for FEV_1 were adjusted for baseline FEV_1 value (except for "baseline severity" based on the baseline per cent predicted normal FEV_1). Models accounted for correlation between treatment effects from multi-arm trials. The between trial variance was assumed to be constant across all comparisons in the network. The Markov Chain Monte Carlo (MCMC) algorithm with four chains was run for each model until convergence was achieved, and 50% of iterations were discarded during the warmup period. Convergence was assessed using the Gelman-Rubin R hat statistic. We used Normal prior distributions for model parameters (i.e., trial-specific event rate or mean, log odds ratio or mean difference, and regression coefficients for covariate terms), except for the between-trial standard deviation, for which we used a half-Normal prior distribution (Table S2). Divergent transitions were handled by choosing appropriate priors (weakly informative or informative) and/or increasing the target average proposal acceptance probability during Stan's adaptation period. Models were fitted using a tree depth of 15. We used the deviance information criteria (DIC) to compare the model fit and complexity of models (e.g., fixed effect and random-effects models; or models with and without interaction terms). If the difference in DIC was greater than five, we focussed interpretation on the model with the lowest DIC; otherwise, we focussed on the simplest model. We also ran models of inconsistency based on unrelated mean effects (UMEs) [5] to assess the consistency assumption based on the agreement of direct and indirect evidence. We evaluated the plausibility of the underlying transitivity assumption by examining covariate distributions across comparisons from an evaluation of treatment-covariate interactions. Treatment rankings were calculated for every outcome. For every outcome variable and fitted model of network meta-analysis or network meta-regression, we assessed the geometry of the treatment network.

Methods S8. Patient and public involvement

We developed the EINSTEIN protocol in consultation with children with asthma and their parents and with National Health Service (NHS) clinicians routinely caring for children with uncontrolled asthma in NHS

settings. We also included a patient with lived experience (OF) as part of the research team. We sought advice on our proposal and the lay summary from five families, including two children, who attended our asthma clinic at Alder Hey. We selected the outcomes in our review from the core outcomes set that clinicians and patients agreed were crucial. [6] Finally, we consulted an Alder Hey patient advisory group comprising children with asthma and their parents.

References

1. British Thoracic Society SIGN. British guideline on the management of asthma. https://www.brit-thoracic.org.uk/quality-improvement/guidelines/asthma/

2. Asthma: diagnosis, monitoring and chronic asthma management. https://www.nice.org.uk/guidance/ng80

3. Reddel HK, Bacharier LB, Bateman ED, et al. Global Initiative for Asthma Strategy 2021: executive summary and rationale for key changes. Eur Respir J. 2021;59(1):2102730. Published 2021 Dec 31. doi:10.1183/13993003.02730-2021

4. Tudur Smith C, Hopkins C, Sydes MR, et al. How should individual participant data (IPD) from publicly funded clinical trials be shared? BMC Med. 2015;13:298. Published 2015 Dec 17. doi:10.1186/s12916-015-0532-z

5. Dias S, Welton NJ, Sutton AJ, et al. NICE DSU Technical Support Document 4: Inconsistency in Networks of Evidence Based on Randomised Controlled Trials. London: National Institute for Health and Care Excellence (NICE); April 2014

6. Sinha IP, Gallagher R, Williamson PR, et al. Development of a core outcome set for clinical trials in childhood asthma: a survey of clinicians, parents, and young people. Trials. 2012;13:103. Published 2012 Jul 2. doi:10.1186/1745-6215-13-103

LIST OF ABBREVIATIONS

ACQ	Asthma Control Questionnaire
ACT	Asthma Control Test
AEs	Adverse Events
AgD	Aggregate Data
AQLQ	Asthma Quality of Life Questionnaire
BDP	Beclomethasone dipropionate
BUD	Budesonide
CIC	Ciclesonide
CI	Confidence Interval
CrI	Credibility Interval
DIC	Deviance Information Criterion
ECG	Electrocardiogram
ED	Emergency Department
FE	Fixed Effect
FEV_1	Forced Expiratory Volume in one second
FF	Fluticasone furoate
FP	Fluticasone propionate
GP	General Practitioner
ICS	Inhaled Corticosteroid
IPD	Individual Participant Data
IQR	Interquartile Range
LABA	Long-Acting β_2-Agonist
LTRA	Leukotriene Receptor Antagonist
MA	Meta-Analysis
MCMC	Markov Chain Monte Carlo
MD	Mean difference
MF	Mometasone furoate
NMA	Network Meta-analysis
NMR	Network Meta-regression
OCS	Oral Corticosteroids
OR	Odds Ratio
PAQLQ	Paediatric Asthma Quality of Life Questionnaire
QoL	Quality of Life
RCT	Randomised Controlled Trial
RE	Random Effects
RR	Relative Risk
SAL	Salmeterol
UME	Unrelated Mean Effects
VI	Vilanterol

Table S1. Estimated clinical comparability daily doses (µg) of Inhaled Corticosteroids

≤ 5-year-old (Children)			
**Drug**	**Low Dose**	**Medium Dose**	**High Dose**
Beclomethasone dipropionate (HFA)	100 (≥5 years)	N.A.	N.A.
Budesonide nebulised	500 (≥1 year)	N.A.	N.A.
Budesonide pMDI + spacer	N.A.	N.A.	N.A.
Fluticasone propionate (HFA)	50 (≥4 years)	N.A.	N.A.
Mometasone furoate	110 (≥4 years)	N.A.	N.A.
Ciclesonide	N.A.	N.A.	N.A.
**6-11-year-old (Children)**			
**Drug**	**Low Dose**	**Medium Dose**	**High Dose**
Beclomethasone dipropionate (CFC)	100-200	>200-400	>400
Beclomethasone dipropionate (HFA)	50-100	>100-200	>200
Budesonide (DPI)	100-200	>200-400	>400
Budesonide (nebules)	250-500	>500-1000	>1000
Ciclesonide	80	>80-160	>160
Fluticasone furoate (DPI)	N.A.	N.A.	N.A.
Fluticasone propionate (DPI)	100-200	>200-400	>400
Fluticasone propionate (HFA)	100-200	>200-500	>500
Mometasone furoate	110	≥220-<440	≥440
**≥ 12-year-old (Adults and adolescents)**			
**Drug**	**Low Dose**	**Medium Dose**	**High Dose**
Beclomethasone dipropionate (CFC)	200-500	>500-1000	>1000
Beclomethasone dipropionate (HFA)	100-200	>200-400	>400
Budesonide (DPI)	200-400	>400-800	>800
Ciclesonide (HFA)	80-160	>160-320	>320
Fluticasone furoate (DPI)	100	N.A.	200
Fluticasone propionate (DPI)	100-250	>250-500	>500
Fluticasone propionate (HFA)	100-250	>250-500	>500
Mometasone furoate	110-220	>220-440	>440

CFC = chlorofluorocarbon propellant (no longer used; included for comparison with older literature); DPI = dry powder inhaler; HFA = hydrofluoroalkane propellant; N.A. = not applicable; pMDI = pressurized metered dose inhaler

Table S2. Prior distributions used in Bayesian NMA and ML-NMR models

**Outcome**	**Model**	**Prior distribution**	
		**Fixed-effect model**	**Random-effects model**
**EXACERBATION**	NMA 1<br>NMA 2	Intercept, trt ~ Normal(0,100^2)	Intercept,<br>trt ~ Normal(0,100^2)<br>het ~ half-Normal(2.5^2)
	ML-NMR<br>All covariates	Intercept, trt, reg ~ Normal(0,100^2)	Intercept, trt, reg ~ Normal(0,100^2)<br>het ~ half-Normal(2.5^2)
**ASTHMA CONTROL**	NMA 1<br>NMA2<br>NMA 3	Intercept, trt ~ Normal(0,10^2)	Intercept, trt ~ Normal(0,100^2)<br>het ~ half-Normal(2.5^2)
	ML-NMR:<br>Age<br>Sex<br>Ethnicity<br>Baseline severity	Intercept, trt, reg ~ Normal(0,100^2)	Intercept, trt, reg ~ Normal(0,100^2)<br>het ~ half-Normal(2.5^2)
	Eczema	Intercept, trt, reg ~ Normal(0,100^2)	Intercept ~ Normal(0,5^2)<br>trt, reg ~ Normal(0,3^2)<br>het ~ half-Normal(0.5^2)
	Eosinophilia	Intercept, trt, reg ~ Normal(0,100^2)	Intercept, trt, reg ~ Normal(0,100^2)<br>het ~ half-Normal(1.5^2)
**FEV_1 (L)**	NMA 1	intercept ~ Normal(0,10^2)<br>trt, aux ~ Normal(0, 5^2)	intercept ~ Normal(scale ~ 100)<br>trt ~ Normal(scale ~ 10)<br>het ~ half-Normal(scale ~ 1.5)<br>aux ~ Normal(scale ~ 10)
	NMA 2	intercept ~ Normal(0,10^2)<br>trt, aux ~ normal(0, 5^2)	intercept ~ Normal(scale ~ 100)<br>trt ~ Normal(scale ~ 10)<br>het ~ half-Normal(scale ~ 1)<br>aux ~ Normal(scale ~ 10)
	NMA 3	intercept ~ Normal(0,100^2)<br>trt, aux ~ Normal(0,10^2)	intercept ~ Normal(scale ~ 100)<br>trt ~ Normal(scale ~ 10)<br>het ~ half-Normal(scale ~ 1.5)<br>aux ~ Normal(scale ~ 10)
	NMR 1*<br>NMR 2*	Intercept,<br>reg ~ Normal(0,10^2)<br>trt, aux ~ Normal(0,5^2)	intercept ~ Normal(scale ~ 10)<br>trt ~ Normal(scale ~ 3)<br>reg ~ Normal(scale ~ 3)<br>het ~ half-Normal(scale ~ 1)<br>aux ~ Normal(scale ~ 3)
	NMR 3*	Intercept, trt ~ Normal(0, 10^2)<br>trt, aux ~ Normal(0, 5^2)	intercept ~ Normal(scale ~ 10)<br>trt ~ Normal(scale ~ 2)<br>reg ~ Normal(scale ~ 2)<br>het ~ half-Normal(scale ~ 1)<br>aux ~ Normal(scale ~ 2)
	ML-NMR:<br>Age<br>Ethnicity	Intercept, aux ~ Normal(0,10^2)<br>trt, reg ~ Normal(0,5^2)	Intercept ~ Normal(0,100^2)<br>trt, reg, aux ~ Normal(0,3^2)<br>het ~ half-Normal(1^2)
	Sex		Intercept ~ Normal(0,100^2)<br>trt, reg, ~ Normal(0,5^2)<br>aux ~ Normal(0,10^2)<br>het ~ half-Normal(1.5^2)
	Eczema	intercept ~ Normal(0,100^2)<br>trt, reg, aux ~ Normal(0,10^2)	intercept ~ Normal(0,10^2)<br>trt, reg, aux ~ Normal(0,2^2)<br>het ~ half-Normal(0.1^2)
	Eosinophilia	intercept ~ Normal(0,100^2)<br>trt, reg, aux ~ Normal(0,5^2)	intercept ~ Normal(0,5^2)<br>trt, reg, aux ~ Normal(0,2^2)<br>het ~ half-Normal(0.5^2)

* the same models as NMA but adjusted for FEV_1 at baseline

NMA 1 = analysis with grouped ICS + LABA; NMA 2 = analysis with stratified ICS dose + LABA; NMA 3 = analysis of individual compounds. The ‘intercept’ represents the log odds of an event in the baseline group, ‘trt’ represents the treatment effects, ‘reg’ represents the regression coefficients for the interaction’ ‘het’ represents the between trial standard deviation; ‘aux’ represents the arm-level standard deviations.

Table S3. Characteristics of the included studies with individual participant data (parts 1 to 6)

Author Year	Countries	Subjects included*, demographics, and clinical features	Patients' characteristics	Protocol inclusion criteria	Study type Blinding	Treatment arms	Follow-up (weeks)
Bateman 2014	USA, Argentina, Australia, Germany, Japan, Mexico, Philippines, Poland, Romania, Russian Federation, Ukraine	N = 213<br>mean age (SD) = 14.1 (1.7)<br>Females – N (%) = 82 (38)<br>Not Hispanic or Latino - N (%) = 141 (66)<br>Eczema – N (%) = NA<br>Eosinophilia – N (%) = 75 (38)<br>BL-severity (mild) – N (%) = 104 (49)	Patients ≥12 years of age with persistent asthma using ICS alone (the doses in Table 1 look low, medium, and high) or ICS+LABA.	Subjects must be using an approved dose of an ICS (as per specific prescribing information) for at least 12 weeks preceding Visit 1 and at a stable dose for at least 4 weeks preceding Visit 1. In addition, subjects may be using a combination product with an ICS (as per specific prescribing information) or an ICS plus a LABA for at least 12 weeks preceding Visit 1 and at a stable dose for at least 4 weeks preceding Visit 1.	parallel groups double-blind	fluticasone furoate/vilanterol 100/25 mcg OD (DPI)<br>fluticasone furoate 100 mcg OD (DPI)	≥24–78<br>mean days (SD)[3]: 378.7 (43.1)
Bernstein 2015	USA, Russia, Argentina, Ukraine, Romania, Chile, Germany, Poland, Mexico, Netherlands, Sweden	N = 42<br>mean age (SD) = 14.6 (1.8)<br>Females – N (%) = 15 (36)<br>Not Hispanic or Latino - N (%) = 23 (55)<br>Eczema – N (%) = NA<br>Eosinophilia – N (%) = 18 (44)<br>BL-severity (mild) – N (%) = 0 (0)	Patients ≥12 years of age with moderate to severe, persistent asthma using ICS or ICS/LABA.	Subjects are eligible if they have received ICS for at least 12 weeks prior to Visit 1 and their treatment during the 4 weeks immediately prior to Visit 1.	parallel groups double-blind	fluticasone furoate/vilanterol 200/25 mcg OD (DPI)<br>fluticasone furoate/vilanterol 100/25 mcg OD (DPI)<br>fluticasone furoate 100 mcg OD (DPI)	12<br>mean days (SD)[3]: 87.2 (13.8)
Bleecker 2012	USA, Canada, Estonia, Germany, Greece, Korea, Mexico, Philippines, Poland, Romania, Russian Federation, Slovakia, South Africa	N = 69<br>mean age (SD) = 14.1 (1.6)<br>Females – N (%) = 28 (41)<br>Not Hispanic or Latino - N (%) = 60 (87)<br>Eczema – N (%) = 42 (61)<br>Eosinophilia – N (%) = 35 (52)<br>BL-severity (mild) – N (%) = 29 (42)	Patients ≥12 years of age with persistent asthma and symptomatic on ICS.	Subjects must have been using an ICS for at least 8 weeks prior to visit 1 and maintained on a stable dose of inhaled corticosteroids for four weeks prior to visit 1	parallel groups double-blind	fluticasone propionate 250 mcg BID (Diskus/Accuhaler)<br>fluticasone furoate 100 mcg OD (DPI)<br>fluticasone furoate 200 mcg OD (DPI)<br>fluticasone furoate 300 mcg OD (DPI)<br>fluticasone furoate 400 mcg OD (DPI)<br>placebo	8<br>mean days (SD)[3]: 52.2 (20.2)
Bleecker 2014	USA, Germany, Japan, Poland, Romania, Ukraine	N = 61<br>mean age (SD) = 14.4 (1.6)<br>Females – N (%) = 24 (39)<br>Not Hispanic or Latino - N (%) = 44 (72)<br>Eczema – N (%) = NA<br>Eosinophilia – N (%) = 14 (23)<br>BL-severity (mild) – N (%) = 17 (28)	Patients with persistent asthma aged 12 years and older (Child, Adult, Older Adult).	All patients must be using an ICS with or without LABA for at least 12 weeks before visit 1.	parallel groups double-blind	fluticasone furoate/vilanterol 100/25 OD (DPI)<br>fluticasone furoate 100 OD (DPI)<br>placebo	12<br>mean days (SD)[3]: 86.6 (25.3)
Carroll 2010	UK	N = 39<br>mean age (SD) = 10.6 (2.8)<br>Females – N (%) = 15 (38)<br>Not Hispanic or Latino - N (%) = 39 (100)<br>Eczema – N (%) = NA<br>Eosinophilia – N (%) = NA<br>BL-severity (mild) – N (%) = 30 (81)	Age 7-18 years (effective range: 7-15). Asthmatic children on 400 mcg/day BDP equivalent.	This study contains 37 participants under 18, although the inclusion criteria allowed the inclusion until 18. All participants were using ICS alone at entry. We included all participants from the dataset provided (39 subjects of whom two withdrew at week four). One of these was withdrawn because of an asthma exacerbation considered as an AE, and the other patient does not have contributing data.	Parallel groups double-blind	fluticasone 100 mcg BD<br>salmeterol/fluticasone 50/100 mcg BD	8<br>mean days (SD)[3]: 56.0 (0.0)
de Blic 2009	Belgium, Denmark, France, Italy, Latvia, Lithuania, Netherlands, Norway, Poland, Russian Federation, Spain, Sweden	N = 303<br>mean age (SD) = 8.0 (2.0)<br>Females – N (%) = 108 (36)<br>Not Hispanic or Latino - N (%) = 292 (96)<br>Eczema – N (%) = 265 (88)<br>Eosinophilia – N (%) = NA<br>BL-severity (mild) – N (%) = 243 (80)	Patients are asthmatic children aged 4 to 11 years not controlled by ICS alone at medium dose.	Patients were receiving beclomethasone HFA or budesonide or fluticasone at least three months prior to visit 1.	parallel groups double-blind	fluticasone propionate/salmeterol 100/50 mcg BID<br>fluticasone propionate 200 mcg BID	12<br>mean days (SD)[3]: 85.0 (7.7)

Author<br>Year	Countries	Subjects included*, demographics, and clinical features	Patients' characteristics	Protocol inclusion criteria	Study type<br>Blinding	Treatment arms	Follow-up (weeks)
Fitzpatrick 2016	USA	N = 60[1]<br>mean age (SD) = 3.0 (1.0)<br>Females – N (%) = 23 (38)<br>Not Hispanic or Latino - N (%) = 52 (87)<br>Eczema – N (%) = 34 (57)<br>Eosinophilia – N (%) = 14 (27)<br>BL-severity (mild) – N (%) = NA	Preschool children 12-59 months of age who meet criteria for treatment with long-term, Step 2 asthma controller therapy.	1) ICS- and LTRA-naïve children treated only with intermittent SABA who require step-up therapy.<br>2) Children on current step 2 therapy who are treated with daily ICS, daily LTRA, or intermittent ICS or LTRA.<br>Thus, the inclusion criteria for this study differ somewhat according to prior ICS and LTRA exposure.	Crossover<br>double-blind	fluticasone propionate HFA – 186 mcg/day<br>montelukast – 4 mg<br>as-needed ICS (FP HFA – 88 mcg) + SABA	P1: 16<br>P2: 16<br>P3: 16<br><br>mean days (SD)[3]:<br>109.9 (17.3)
Gappa 2009	Germany	N = 262<br>mean age (SD) = NA<br>Females – N (%) = 81 (31)<br>Not Hispanic or Latino - N (%) = 262 (100)<br>Eczema – N (%) = NA<br>Eosinophilia – N (%) = NA<br>BL-severity (mild) – N (%) = 192 (76)	Patients are children and adolescents 4 to 16 years of age with documented history of persisting seasonal or perennial bronchial asthma.	Patients must have been pretreated with an inhaled corticosteroid at a dosage of 200-400 µg BDP equivalents / day during the last 4 weeks.	Parallel groups<br>double-blind	fluticasone propionate/salmeterol 100/50 mcg BID (Diskus)<br>fluticasone propionate 200 mcg BID (Diskus)	8<br><br>mean days (SD)[3]:<br>56.7 (3.9)
Lemanske 2010	USA	N = 31<br>mean age (SD) = 10.6 (3.7)<br>Females – N (%) = 8 (26)<br>Not Hispanic or Latino - N (%) = 17 (55)<br>Eczema – N (%) = 7 (23)<br>Eosinophilia – N (%) = 14 (45)<br>BL-severity (mild) – N (%) = 27 (87)	Patients aged 6 to 17 with a lack of acceptable asthma control during run-in period.	Children enrolled into BADGER can be characterized as falling into one of three groups:<br>• Step-neutral – currently receiving an ICS dose = 200 ug/day fluticasone equivalent<br>• Step-up – naïve to controller therapy or receiving an ICS dose < 200 ug/day fluticasone equivalent or non-ICS controller therapy (e.g., montelukast, theophylline or cromolyn), and needing step-up therapy<br>• Step-down – currently receiving controller therapy considered by the NAEPP guidelines to be a step above 1x ICS (e.g. 2x ICS or combination therapy of 1x ICS + LABA, montelukast, theophylline or cromolyn)	crossover<br>double-blind	2x ICS: DPI 250 mcg fluticasone + DPI 250 mcg fluticasone + placebo<br>1x ICS + LTRA: DPI 100 mcg fluticasone + DPI 100 mcg fluticasone + montelukast<br>1x ICS + LABA: DPI 100 mcg fluticasone/50 mcg salmeterol + DPI 100 mcg fluticasone/50 mcg salmeterol + placebo	P1: 16<br>P2: 16<br>P3: 16<br><br>mean days (SD)[3]:<br>106.4 (17.4)
Li 2010	USA, Australia, Canada, Chile, Costa Rica, Germany, Latvia, Lithuania, Mexico, Peru, Poland, Russian Federation, Spain	N = 350<br>mean age (SD) = 7.6 (2.1)<br>Females – N (%) = 137 (39)<br>Not Hispanic or Latino - N (%) = 207 (59)<br>Eczema – N (%) = NA<br>Eosinophilia – N (%) = 191 (56)<br>BL-severity (mild) – N (%) = 195 (71)	Patients are children aged 4 to 11 years with asthma requiring pharmacotherapy for at least two months. Patients were using ICS at a consistent dose (low-medium doses) and SABA.	ICS doses:<br>beclomethasone (CFC): 84-100 to 336-400<br>beclomethasone (HFA): 84-100 to 160-200<br>FP (powder): 100 to 200<br>FP (CFC or HFA): 88-100 to 176-200<br>BUD (powder): 200 to 400<br>BUD repulse: 500	parallel groups<br>double-blind	fluticasone propionate/salmeterol 100/50 mcg BID (HFA)<br>fluticasone propionate 100 mcg BID (HFA)	12<br><br>mean days (SD)[3]:<br>80.5 (19.3)
Lötvall 2014a1 §	USA, Germany, Peru, Poland, Ukraine	N = 20<br>mean age (SD) = 14.3 (1.9)<br>Females – N (%) = 8 (40)<br>Not Hispanic or Latino - N (%) = 6 (30)<br>Eczema – N (%) = NA<br>Eosinophilia – N (%) = NA<br>BL-severity (mild) – N (%) = 5 (25)	Patients ≥12 years of age with persistent asthma using a low, medium, or high dose of ICS at visit 1.	All subjects must be using an ICS for at least 12 weeks prior to visit 1. Subjects must be taking a stable dose of ICS (e.g., FP 200-1000 mcg twice daily or equivalent) for at least 4 weeks prior to visit 1. Subjects will be stratified at randomization according to whether they are on low, medium or high dose ICS at visit 1.	parallel groups<br>double-blind	vilanterol 25mcg OD (DPI)<br>salmeterol 50 mcg BID (DPI)<br>placebo<br><br>All patients were additionally using their baseline ICS dose.	12<br><br>mean days (SD)[3]:<br>91.0 (18.0)
Lötvall 2014a2 §		N = 26<br>mean age (SD) = 14.1 (1.6)<br>Females – N (%) = 15 (58)<br>Not Hispanic or Latino - N (%) = 13 (50)<br>Eczema – N (%) = NA<br>Eosinophilia – N (%) = NA<br>BL-severity (mild) – N (%) = 4 (16)					12<br><br>mean days (SD)[3]:<br>95.3 (8.1)

Author Year	Countries	Subjects included*, demographics, and clinical features	Patients' characteristics	Protocol inclusion criteria	Study type Blinding	Treatment arms	Follow-up (weeks)
Lötvall 2014b	USA, Belgium, Germany, Poland, Romania	N = 46<br>mean age (SD) = 13.9 (1.7)<br>Females – N (%) = 20 (43)<br>Not Hispanic or Latino - N (%) = 44 (96)<br>Eczema – N (%) = NA<br>Eosinophilia – N (%) = 14 (31)<br>BL-severity (mild) – N (%) = 16 (36)	Patients ≥12 years of age with persistent asthma taking a stable dose of ICS.	All subjects must be taking a stable dose of ICS for at least 4 weeks prior to Visit 1.	parallel groups<br>double-blind	fluticasone furoate 100 mcg OD (DPI)<br>fluticasone propionate 250 mcg BID (Diskus/Accuhaler)<br>placebo	24<br>mean days (SD)[3]: 163.4 (31.9)
Martin 2020	USA, Canada	N = 11<br>mean age (SD) = 13.7 (2.1)<br>Females – N (%) = 4 (36)<br>Not Hispanic or Latino - N (%) = 11 (100)<br>Eczema – N (%) = NA<br>Eosinophilia – N (%) = NA<br>BL-severity (mild) – N (%) = 11 (100)	Patients aged 12 to 50 years taking low or moderate dose ICS for 12 weeks before visit 1.	Patients with intermittent asthma, seasonal asthma, or exercise-induced bronchoconstriction only were NOT eligible.	crossover<br>double-blind	FF/VI 100/25 mcg QD via Ellipta + Placebo BD via Diskus<br>FP 250 mcg BD via Diskus + Placebo QD via Ellipta	P1: 2<br>washout: 2<br>P2: 2<br>mean days (SD)[3]: 14.4 (1.0)
Murray 2010	New Zealand, UK	N = 13<br>mean age (SD) = 7.7 (2.1)<br>Females – N (%) = 9 (69)<br>Not Hispanic or Latino - N (%) = 13 (100)<br>Eczema – N (%) = 13 (100)<br>Eosinophilia – N (%) = NA<br>BL-severity (mild) – N (%) = NA	Patients aged 4 to 11 years with asthma diagnosed by physicians.	Receiving a total daily dose of 200-800mcg/day BDP or equivalent for at least 4 weeks prior to the start of the run-in period, and in physicians' opinion be sufficiently stable to receive FP 200mcg/day during the 2-week run-in period.	parallel groups<br>double-blind	fluticasone propionate 100 mcg bd BID + fluticasone propionate 100 mcg BID (ACTIVE/ACTIVE)<br>fluticasone propionate/salmeterol 100/50 mcg BID + placebo (ACTIVE/PLACEBO)	6<br>mean days (SD)[3]: 42.5 (0.9)
Murray 2011	USA	N = 230<br>mean age (SD) = 11.5 (3.4)<br>Females – N (%) = 99 (43)<br>Not Hispanic or Latino - N (%) = 202 (88)<br>Eczema – N (%) = NA<br>Eosinophilia – N (%) = NA<br>BL-severity (mild) – N (%) = 157 (68)	Patients are children aged 4 to 17 years with persistent asthma on ICS alone (low-medium doses) and SABA.	Each subject must have been treated for their asthma with one of the following inhaled corticosteroids at the specified daily dosing range for at least 4 weeks prior to Visit 1 and with no other inhaled long acting bronchodilators for at least 2 weeks prior to Screening.<br>Beclomethasone: 84-336 (4-11 y); 168-504 (12-17 y)<br>FP: 88-220 (4-11 y); 88-264 (12-17 y)<br>Budesonide: 200-400 (4-11 y); 200-600 (12-17 y)<br>Not of interest: QVAR, triamcinolone, flunisolide	parallel groups<br>double-blind	fluticasone propionate/salmeterol 100/50 mcg BID (Diskus)<br>fluticasone propionate 100 mcg BID (Diskus)	4<br>mean days (SD)[3]: 28.1 (3.6)
O'Byrne 2014	USA, Germany, Japan, Poland, Romania, Russian Federation	N = 10<br>mean age (SD) = 15.8 (1.4)<br>Females – N (%) = 2 (20)<br>Not Hispanic or Latino - N (%) = 10 (100)<br>Eczema – N (%) = NA<br>Eosinophilia – N (%) = 2 (22)<br>BL-severity (mild) – N (%) = 1 (10)	Patients ≥12 years of age with persistent asthma using ICS alone (FP 500 mcg twice daily or equivalent) or ICS+LABA.	All patients must be using an ICS with or without LABA for at least 12 weeks before visit 1.	parallel groups<br>double-blind	fluticasone furoate/vilanterol 200/25 mcg OD (DPI)<br>fluticasone furoate 200 mcg OD (DPI)<br>fluticasone propionate 500 mcg BID (Diskus/Accuhaler)<br>placebo	24<br>mean days (SD)[3]: 174.4 (4.8)
Oliver 2016a	USA, Argentina, Chile, Georgia, Germany, Japan, Mexico, Peru, Philippines, Poland, Puerto Rico, Slovakia, South Africa, Ukraine	N = 456<br>mean age (SD) = 7.9 (1.8)<br>Females – N (%) = 180 (39)<br>Not Hispanic or Latino - N (%) = 129 (28)<br>Eczema – N (%) = NA<br>Eosinophilia – N (%) = 175 (41)<br>BL-severity (mild) – N (%) = 173 (45)	Patients aged 5-11 with a history of symptoms consistent with asthma diagnosis for at least 6 months prior to Visit 1. Asthma on a background of inhaled corticosteroid therapy.	Subjects with persistent uncontrolled asthma must been receiving stable asthma therapy for at least 4 weeks prior to screening: SABA + ICS (total daily dose FP 250 mcg or equivalent).	parallel groups<br>double-blind	placebo OD + FP 100 BID<br>vilanterol 6.25 mcg OD + FP 100 BID<br>vilanterol 12.5 mcg OD + FP 100 BID<br>vilanterol 25 mcg OD + FP 100 BID	5<br>mean days (SD)[3]: 32.8 (7.2)
Oliver 2016b	USA, Bulgaria, Georgia, Germany, Japan, Latvia, Mexico, Peru, Philippines, Poland, Puerto Rico, Russian Federation, South Africa, Sweden, Ukraine	N = 318<br>mean age (SD) = 8.1 (1.9)<br>Females – N (%) = 119 (37)<br>Not Hispanic or Latino - N (%) = 165 (52)<br>Eczema – N (%) = NA<br>Eosinophilia – N (%) = 96 (34)<br>BL-severity (mild) – N (%) = 150 (47)	Patients aged 5-11 with a history of symptoms consistent with asthma diagnosis for at least 6 months prior to Visit 1.	Subjects with persistent uncontrolled asthma must been receiving stable asthma therapy for at least 4 weeks prior to screening: SABA alone, SABA+leukotriene, or SABA+ low-dose ICS.	parallel groups<br>double-blind	placebo<br>FP 100 mcg Diskus<br>FF 25 mcg NDPI<br>FF 50 mcg NDPI<br>FF 100 mcg NDPI	13<br>mean days (SD)[3]: 75.4 (27.3)

Author Year	Countries	Subjects included*, demographics, and clinical features	Patients' characteristics	Protocol inclusion criteria	Study type Blinding	Treatment arms	Follow-up (weeks)
Pearlman 2009	USA	N = 248<br>mean age (SD) = 11.1 (3.4)<br>Females – N (%) = 99 (40)<br>Not Hispanic or Latino - N (%) = 228 (92)<br>Eczema – N (%) = NA<br>Eosinophilia – N (%) = NA<br>BL-severity (mild) – N (%) = 167 (67)	Patients are children aged 4 to 17 years with persistent asthma using ICS (low-medium doses) and SABA.	Each subject must have been treated for their asthma with inhaled corticosteroids at the specified daily dosing range for at least 4 weeks prior to Visit 1 and with no other inhaled long acting bronchodilators for at least 2 weeks prior to Screening.<br>Beclomethasone: 84-336 (4-11 y); 168-504 (12-17 y)<br>FP: 88-220 (4-11 y); 88-264 (12-17 y)<br>Budesonide: 200-400 (4-11 y); 200-600 (12-17 y)<br>Not of interest: QVAR, triamcinolone, flunisolide	parallel groups double-blind	fluticasone propionate/salmeterol 100/50 mcg BID (Diskus)<br>fluticasone propionate 100 mcg BID (Diskus)	4<br>mean days (SD)[3]: 27.9 (4.3)
Scott 2005[€]	USA, Canada	N = 199<br>mean age (SD) = 8.0 (2.2)<br>Females – N (%) = 73 (37)<br>Not Hispanic or Latino - N (%) = 181 (91)<br>Eczema – N (%) = NA<br>Eosinophilia – N (%) = 99 (51)<br>BL-severity (mild) – N (%) = 70 (43)	Patients are children aged 4 to 11 years with asthma requiring maintenance treatment (ICS or medication other than ICS or SABA alone).	Concurrent anti-asthma therapy.<br>GROUP 1 > Inhaled corticosteroids: subjects must have been using inhaled corticosteroids for at least 3 months prior to Visit 1; and at least one month before Visit 1, must have been on a consistent daily dose of one of the reported table (doses are low-medium).<br>GROUP 2 > Maintenance asthma medication other than inhaled corticosteroids: subjects are eligible if treated with a maintenance asthma medication other than inhaled corticosteroid (e.g., salmeterol, cromolyn or nedocromil, or montelukast) on a regular basis for at least 4 weeks prior to visit 1 OR Short acting beta2 agonists: subjects are eligible if treated with SABA alone for relief of respiratory for at least 4 weeks prior to visit 1 and should not have received an inhaled corticosteroid or maintenance asthma medication other than inhaled corticosteroids for at least 4 weeks prior to visit 1.	parallel groups double-blind	fluticasone propionate/salmeterol 100/50 mcg BID (Diskus)<br>fluticasone propionate 100 mcg BID (Diskus)	12<br>mean days (SD)[3]: 79.0 (17.7)
Sorkness 2007	USA	N = 49<br>mean age (SD) = 9.3 (2.2)<br>Females – N (%) = 15 (31)<br>Not Hispanic or Latino - N (%) = 36 (73)<br>Eczema – N (%) = 30 (61)<br>Eosinophilia – N (%) = 29 (63)<br>BL-severity (mild) – N (%) = 42 (86)	Children ages 6-14 years with mild-moderate persistent asthma defined by symptom criteria and positive methacholine challenge.	Only the naïve group could not use ICS at entry.	parallel groups double-blind	fluticasone propionate (100 mcg BID - Diskus)<br>fluticasone/salmeterol (100 mcg/50 mcg qd - Diskus) + salmeterol (50 mcg qd - Diskus)<br>montelukast (5 mg qd)	48<br>mean days (SD)[3]: 331.6 (32.2)
Stempel 2016a	USA, Argentina, Australia, Austria, Belgium, Bulgaria, Canada, Chile, Colombia, Croatia, Czechia, Germany, Hungary, Italy, Korea, Latvia, Lithuania, Malaysia, Mexico, Peru, Philippines, Poland, Romania, Russian Federation, Serbia, Slovakia, South Africa, Spain, Taiwan, Thailand, Ukraine, UK	N = 1631<br>mean age (SD) = 7.4 (2.2)<br>Females – N (%) = 647 (40)<br>Not Hispanic or Latino - N (%) = 1164 (71)<br>Eczema – N (%) = 334 (20)<br>Eosinophilia – N (%) = NA<br>BL-severity (mild) – N (%) = NA	Patients are children aged 4 to 11 years with persistent asthma.	The allowed pre-treatment consisted of ICS alone (different doses) or ICS with other medicines (LABA, LTRA, theophylline) or SABA, LABA, LTRA, theophylline alone.	parallel groups double-blind	fluticasone propionate - salmeterol combination 100/50<br>fluticasone propionate - salmeterol combination 250/50<br>fluticasone propionate 100<br>fluticasone propionate 250	26<br>mean days (SD)[3]: 168.1 (45.8)

Author Year	Countries	Subjects included*, demographics, and clinical features	Patients' characteristics	Protocol inclusion criteria	Study type Blinding	Treatment arms	Follow-up (weeks)
Stempel 2016b	USA, Argentina, Australia, Austria, Belgium, Bulgaria, Canada, Chile, Colombia, Croatia, Czechia, Denmark, Germany, Hungary, Indonesia, Italy, Korea, Latvia, Lithuania, Malaysia, Mexico, Peru, Philippines, Poland, Romania, Russian Federation, Serbia, Slovakia, South Africa, Spain, Taiwan, Ukraine, UK	N = 222<br>mean age (SD) = 14.2 (1.6)<br>Females – N (%) = 104 (47)<br>Not Hispanic or Latino - N (%) = 156 (70)<br>Eczema – N (%) = 33 (15)<br>Eosinophilia – N (%) = NA<br>BL-severity (mild) – N (%) = NA	Patients are adolescents (12-17) and adults (18+) with persistent asthma.	Patients were stratified based on the entry medicine (ICS alone or ICS+LABA, ICS+LTRA, ICS+theophylline) and ACQ score.	parallel groups<br>double-blind	FP 100 mcg<br>FP+SAL 100/50 mcg<br>FP 250 mcg<br>FP+SAL 250/50 mcg<br>FP 500 mcg<br>FP+SAL 500/50 mcg	26<br>mean days (SD)[3]: 161.8 (51.0)
Thomas 2014	Singapore	N = 33<br>mean age (SD) = 11.1 (3.1)<br>Females – N (%) = 12 (36)<br>Not Hispanic or Latino - N (%) = 33 (100)<br>Eczema – N (%) = 16 (48)<br>Eosinophilia – N (%) = 6 (18)<br>BL-severity (mild) – N (%) = 17 (52)	Children and adolescents aged 6-18 years with uncontrolled or partially controlled asthma on 400 mcg BDP.	Children with uncontrolled or partially controlled asthma, on low-medium dose (400mg BDP [Beclomethasone dipropionate] equivalent) ICS monotherapy.	parallel groups<br>open-label	ICS: 200 mcg of fluticasone twice daily<br>ICS+LABA: 100 mcg of fluticasone plus 50mg of salmeterol (Seretide 50/100 Accuhaler, GlaxoSmithKline) twice daily<br>ICS+LTRA: 100 mcg of fluticasone twice daily plus montelukast (Singulair, MSD) 5 mg (for children 15 years) or 10 mg (for >15 years)	8<br>mean days (SD)[3]: 60.0 (0.0)
Vaessen-Verberne 2010	Netherlands	N = 158<br>mean age (SD) = NA<br>Females – N (%) = 67 (42)<br>Not Hispanic or Latino - N (%) = 158 (100)<br>Eczema – N (%) = NA<br>Eosinophilia – N (%) = NA<br>BL-severity (mild) – N (%) = NA	Children aged 6-16 years with symptomatic asthma.	Subjects who have received BDP, budesonide up to 100-200 mcg bd or fluticasone propionate at a dose of up to 125 mcg bd for at least 4 weeks before the start of the run-in period.	parallel groups<br>double-blind	fluticasone propionate/salmeterol 100/50 mcg BID<br>fluticasone propionate 200 mcg BID	10<br>mean days (SD)[3]: NA
Verberne 1998	Netherlands	N = 177<br>mean age (SD) = 11.2 (2.7)<br>Females – N (%) = 58 (33)<br>Not Hispanic or Latino - N (%) = 177 (100)<br>Eczema – N (%) = NA<br>Eosinophilia – N (%) = NA<br>BL-severity (mild) – N (%) = 119 (67)	Children aged 6 to 16 years with moderate asthma.	A history of stable asthma for at least 1 mo without exacerbations or respiratory tract infections; (6) used inhaled corticosteroids between 200 and 800 mcg daily for at least 3 months before the start of the study.<br>From discussion: During the 6-wk run-in period they were treated with 200 mg beclomethasone twice daily, which is considered a moderate dose in the treatment of childhood asthma (14). Despite this treatment all children were symptomatic and had reversible airway obstruction and airway hyperresponsiveness.	parallel groups<br>double-blind	beclomethasone+ SAL (BDP400+SAL100 mcg)<br>beclomethasone (BDP800)<br>placebo+beclomethasone (BDP400)	54<br>mean days (SD)[3]: 362.8 (61.5)
Wechsler 2019	USA	N = 172<br>mean age (SD) = 9.2 (2.9)<br>Females – N (%) = 77 (45)<br>Not Hispanic or Latino - N (%) = 172 (100)<br>Eczema – N (%) = 98 (70)<br>Eosinophilia – N (%) = 63 (37)<br>BL-severity (mild) – N (%) = 28 (100)	Patients aged 5 or older with at least one Black grandparent.	To enter the run-in, participants must be either: A) inadequately controlled on low-, medium- or high-dose ICS monotherapy, or low- or medium-dose ICS/LABA, or B) well-controlled on low-, medium- or high-dose ICS monotherapy, or low-, medium- or high-dose ICS/LABA (see Study Visits, Screen A, at -10 weeks).	crossover<br>double-blind	**5-11 years**<br>2xICS = fluticasone 100 mcg (Diskus) BID<br>2xICS/LABA = 100/50 mcg (Advair Diskus - FP+SAL) BID<br>5xICS = fluticasone 250 mcg (Diskus) BID<br>5xICS/LABA = 250/50 mcg (Advair Diskus - FP+SAL) BID<br>**12-17 years**<br>2.5xICS = fluticasone 250 mcg (Diskus) BID<br>1xICS/LABA = 100/50 mcg (Advair Diskus - FP+SAL) BID<br>5xICS = fluticasone 500 mcg (Diskus) BID<br>2.5xICS/LABA = 250/50 mcg (Advair Diskus - FP+SAL) BID	P1: 14<br>P2: 14<br>P3: 14<br>P4: 14<br>mean days (SD)[3]: 91.4 (27.1)
Woodcock 2013	USA, Argentina, Chile, Korea, Netherlands, Philippines	N = 32<br>mean age (SD) = 13.8 (1.6)<br>Females – N (%) = 9 (28)<br>Not Hispanic or Latino - N (%) = 19 (59)<br>Eczema – N (%) = NA<br>Eosinophilia – N (%) = 17 (65)<br>BL-severity (mild) – N (%) = 8 (25)	Patients ≥12 years of age with persistent asthma using ICS.	Subjects must have been using an inhaled corticosteroid for at least 12 weeks prior to visit 1 and be maintained on a medium dose (e.g., FP 250 mcg twice daily) for at least 4 weeks prior to Visit 1.	parallel groups<br>double-blind	fluticasone furoate/vilanterol 100/25 mcg OD (DPI)<br>fluticasone propionate/salmeterol 250/50 mcg BID (Diskus/Accuhaler)<br>placebo	24<br>mean days (SD)[3]: 164.5 (29.9)

Author Year	Countries	Subjects included*, demographics, and clinical features	Patients' characteristics	Protocol inclusion criteria	Study type Blinding	Treatment arms	Follow-up (weeks)
Woodcock 2014	USA, Argentina, Chile, France, Mexico, Russian Federation	N = 13<br>mean age (SD) = 14.7 (1.4)<br>Females – N (%) = 5 (38)<br>Not Hispanic or Latino - N (%) = 10 (77)<br>Eczema – N (%) = NA<br>Eosinophilia – N (%) = 5 (71)<br>BL-severity (mild) – N (%) = 5 (42)	Patients ≥12 years of age with persistent asthma with a stable dose, and regimen of ICS.	All subjects must be on stable dose, and regimen of ICS for at least 4 weeks prior to Visit 1.	parallel groups<br>double-blind	fluticasone furoate 100 mcg OD (DPI)<br>fluticasone furoate 200 mcg OD (DPI)	24<br>mean days (SD)[3]: 174.5 (14.9)

*<18 and on ICS alone at randomization or at screening visit if not available
[1] as-needed group was not considered
[€] no publication; only two no longer working links of congress abstracts
[3] follow up of included participants
§ split into two sub-studies because of randomization bias due to the treatment dose categorization based on age class with GINA
ICS = inhaled corticosteroids; LABA = long-acting beta-agonists; LTRA = leukotriene receptor antagonist; BDP = beclomethasone dipropionate; FP = fluticasone propionate; FF = fluticasone furoate; BUD = budesonide; MF = mometasone furoate; SAL = salmeterol; SABA = short-acting beta-agonist
BD/BID = twice a day; OD/QD = once a day; DPI = dry powder inhaler; HFA = hydrofluoroalkane propellant
NA = not available; BL-severity = baseline asthma severity

NOTES: All children using ICS+LABA or other medicines/medicine combinations different from ICS alone at the screening visit were excluded. That was possible because we had sufficient information, from the individual participant data and the appropriate documentation supplied by the data providers (protocol, code of variables, statistical analysis plan, etc.). Conversely, that was not possible for the studies listed in Table S5 without IPD.

Table S4. Characteristics of the included studies with aggregate data (parts 1 to 4)

Study	Countries	Patients included, demographics, clinical features	Patient Characteristics	Study type Blinding	Follow up (weeks)	Interventions (participants)
Akpinarli 1999	Turkey	N = 32<br>mean age (SD) = 10.3 (13.1)<br>Females – N (%) = 17 (53)<br>Not Hispanic or Latino – N (%) = NA<br>Eczema – N (%) = 21 (65.6)<br>Eosinophilia – N (%) = NA<br>BL-severity (mild) – N (%) = NA	sex: 15 M and 17 F<br>mean age: 10.25 - SE age: 2.31 (SD = 13.07)<br>eczema: ICS+LABA = 11; ICS + placebo = 10<br>asthma severity (FEV1 % predicted):<br>ICS+LABA = 79; ICS + placebo = 80	parallel groups double-blind	6	ICS + formoterol (16)<br>ICS + placebo (16)<br><br>ICS: 400-800 mcg day (no medicine specified)
Berger 2006	USA	N = 296<br>mean age (SD) = 8.6 (1.8)<br>Females – N (%) = 109 (37)<br>Not Hispanic or Latino – N (%) = 228 (77)<br>Eczema – N (%) = NA<br>Eosinophilia – N (%) = NA<br>BL-severity (mild) – N (%) = NA	sex:<br>100 mcg F=41; M=57;<br>200 mcg F=32; M=67;<br>placebo F=36; M=63<br>mean age:<br>100 mcg = 9.0 (SD = 1.8);<br>200 mcg = 8.7 (SD = 1.8);<br>placebo = 8.2 (SD = 1.9)<br>ethnicity:<br>100 mcg: White=56; Black=16; Hispanic=22; Asian=1; Native American=1; Other=2<br>200 mcg: White=63; Black=11; Hispanic=22; Asian=1; Native American=2; Other=0<br>placebo: White=60; Black=12; Hispanic=24; Asian=0; Native American=0; Other=3<br>asthma severity (FEV1 % predicted):<br>100 mcg = 79.2; 200 mcg = 79.7; placebo = 77.3<br>BL_FEV1 (mean): 100 mcg = 1.60; 200 mcg = 1.57; placebo = 1.45<br><br>Baseline ICS use includes a small percentage of triamcinolone and flunisolide.	parallel groups double-blind	12	mometasone furoate DPI 100 mcg (98)<br>mometasone furoate DPI 200 mcg (99)<br>placebo (99)
Bisgaard 2006	Argentina, Brazil, Bulgaria, Canada, China, France, Great Britain, Hungary, Indonesia, Israel, Italy, Malaysia, Mexico, Norway, Philippines, Poland, Romania, Singapore, South Africa, Sweden, Taiwan, Turkey	N = 341<br>mean age (SD) = 8 (NA)<br>Females – N (%) = 104 (30)<br>Not Hispanic or Latino – N (%) = NA<br>Eczema – N (%) = NA<br>Eosinophilia – N (%) = NA<br>BL-severity (mild) – N (%) = NA	sex:<br>BUD M = 70, F = 36;<br>BUD/FORM M = 85, F = 35;<br>SMART M = 85, F = 33<br>mean age: BUD = 8; BUD/FORM = 8; SMART = 8 (no SD)<br>race: BUD white = 90, other = 16; BUD/FORM white = 101, other = 16; SMART white = 100, other= 18<br>asthma severity (FEV1 % predicted): BUD = 76; BUD/FORM = 76; SMART = 76<br>exacerbation: BUD = 28; BUD/FORM = 44; SMART = 17<br>BL_FEV1 (L): BUD = 1.6; BUD/FORM = 1.5; SMART = 1.6<br>FEV1 (L): BUD = 1.76; BUD/FORM = 1.70; SMART = 1.86	parallel groups double-blind	52	BUD 320 mcg qd (fixed dose) (106)<br>BUD/FORM 80/4.5 mcg qd (fixed dose) (117)<br>BUD/FORM 80/4.5 mcg qd maintenance + as needed (SMART) (118)
Buchvald 2003[1]	Denmark	N = 23<br>mean age (SD) = 12 (NA)<br>Females – N (%) = 11 (48)<br>Not Hispanic or Latino – N (%) = NA<br>Eczema – N (%) = 7 (30)<br>Eosinophilia – N (%) = NA<br>BL-severity (mild) – N (%) = NA	sex: M=12; F=11<br>mean age: 12 (no SD)<br>eczema: 7<br>mean asthma severity: 101<br>mean FEV1 (L): BUD+placebo = 2.48; BUD+LTRA = 2.57; BUD+SAL = 2.63 (N=22)<br>mean BL_FEV1 (L): 2.54 (N=22)<br>exacerbation: 0<br><br>Crossover study without the possibility to use the data from the first period only.	crossover double-blind	P1 = NA<br>P2 = NA<br>P3 = NA<br><br>no washout	BUD 400 mcg die + salmeterol 50 mcg BID (23)<br>BUD 400 mcg die + montelukast 5 mg OD (23)<br>BUD 400 mcg die + placebo (23)

Study	Countries	Patients included, demographics, clinical features	Patient Characteristics	Study type Blinding	Follow up (weeks)	Interventions (participants)
Everden 2004[2]	UK, Republic of Ireland	N = 155<br>mean age (SD) = 11.8 (2.9)<br>Females – N (%) = 67 (43)<br>Not Hispanic or Latino – N (%) = NA<br>Eczema – N (%) = NA<br>Eosinophilia – N (%) = NA<br>BL-severity (mild) – N (%) = NA	sex: ICS+FORM M = 50, F = 29; ICS+SAL M = 38, F = 38<br>mean age: ICS+FORM = 11.7 (SD = 3.0); ICS+SAL = 11.8 (SD = 2.8)<br>exacerbation (mean episodes): ICS+FORM = 8; ICS+SAL = 12<br>asthma aggravation (AEs): ICS+FORM = 8; ICS+SAL = 10	parallel groups<br>open-label	12	ICS+formoterol (79)<br>ICS+salmeterol (76)<br><br>The ICS dose is unknown.
Heuck 2000	Denmark	N = 24<br>mean age (SD) = 9.5 (NA)<br>Females – N (%) = 10 (42)<br>Not Hispanic or Latino – N (%) = NA<br>Eczema – N (%) = NA<br>Eosinophilia – N (%) = NA<br>BL-severity (mild) – N (%) = NA	mean age: 9.5 (3 patients more) (no SD)<br>sex: M = 14; F = 13 (3 patients more)<br>exacerbation: BUD+placebo = 2; BUD+FORM = 0	crossover<br>double-blind	P1 = 6<br>P2 = 6	budesonide+formoterol 200/24 mcg die DPI (14)<br>budesonide DPI (400 mcg) + placebo die (10)
Jat 2006	India	N = 63<br>mean age (SD) = 9.8 (2.6)<br>Females – N (%) = 18 (29)<br>Not Hispanic or Latino – N (%) = NA<br>Eczema – N (%) = NA<br>Eosinophilia – N (%) = NA<br>BL-severity (mild) – N (%) = NA	sex: ICS+LTRA M = 21, F = 9; ICS M = 24, F = 9<br>mean age: ICS+LTRA = 10.13 (SD = 2.67); ICS = 9.39 (SD = 2.46)<br>asthma severity (FEV1 % predicted): ICS+LABA = 64.17; ICS = 63.36<br>exacerbation: ICS+LTRA = 10; ICS = 3 (first exacerbation)	parallel groups<br>blinded	12	A: budesonide (200 mcg) + montelukast (5 mg) die (30)<br>B: budesonide (400 mcg) die (33)
Kondo 2006	Japan	N = 75<br>mean age (SD) = 9.1 (2.3)<br>Females – N (%) = 31 (41)<br>Not Hispanic or Latino – N (%) = NA<br>Eczema – N (%) = NA<br>Eosinophilia – N (%) = 46 (61)<br>BL-severity (mild) – N (%) = 42 (56)	sex: montelukast M = 21, F = 18; theophylline M = 23, F = 13<br>mean age: montelukast = 9.4 (SD = 2.4); theophylline = 8.8 (SD = 2.2)<br>asthma severity:<br>montelukast − mild = 24, moderate = 12, severe = 3<br>theophylline − mild = 18, moderate = 16, severe = 2<br>phenotype:<br>montelukast − non-eosinophilic = 12, eosinophilic = 27<br>theophylline − non-eosinophilic = 17, eosinophilic = 19<br><br>exacerbation: montelukast = 1; theophylline = 1 (status asthmaticus and asthma aggravation)<br>Data are available for the PP population only (75 of 79 ITT) - randomized: 84.	parallel groups<br>open-label	4	ICS (CFC-BDP: 100-400 mcg or FP: 100-200 mcg) + montelukast 5 mg die (39)<br>ICS (CFC-BDP: 100-400 mcg or FP: 100-200 mcg) + theophylline 10—16 mg/kg/day or 200—400 mg/day (36)
Lenney 2013 (MASCOT)	UK	N = 63<br>mean age (SD) = 10 (21)<br>Females – N (%) = 23 (37)<br>Not Hispanic or Latino – N (%) = NA<br>Eczema – N (%) = NA<br>Eosinophilia – N (%) = NA<br>BL-severity (mild) – N (%) = NA	sex: ICS − M = 17, F = 2; ICS+LABA − M = 13, F = 10; ICS+LTRA − M = 10, F = 11<br>mean age: ICS = 10.37 (SD=19); ICS+LABA = 10.46 (SD=23); ICS+LTRA = 10.33 (SD=21)<br>asthma severity (FEV1 % predicted): ICS = 88.29; ICS+LABA = 79.79; ICS+LTRA = 86.47<br>BL_FEV1 (L): ICS = 1.98; ICS+LABA = 1.83; ICS+LTRA = 1.82<br><br>exacerbation (any): ICS = 4/19; ICS+LABA = 7/23; ICS+LTRA = 3/21 (Tot: 14/63)<br>exacerbation (OC): ICS = 4/18; ICS+LABA = 3/17; ICS+LTRA = 3/19 (Tot: 10/54) (24 weeks)	parallel groups<br>double-blind	48	FP 200 mcg die (19)<br>FP 200 mcg +SAL 100 mcg die (23)<br>FP 200 mcg +montelukast 5 mg die (21)
Malone 2005	USA, Canada	N = 203<br>mean age (SD) = 8.1 (NA)<br>Females – N (%) = 73 (36)<br>Not Hispanic or Latino – N (%) = NA<br>Eczema – N (%) = NA<br>Eosinophilia – N (%) = NA<br>BL-severity (mild) – N (%) = NA	sex: FP − M = 59, F = 41; FP+SAL − M = 68, F = 32;<br>mean age: FP = 8.1; FP+SAL = 8.0 (no SD)<br>race:<br>FP − White = 72, Black = 16, other = 12;<br>FP+SAL − White = 67, Black = 23, other = 10;<br>asthma severity (FEV1 % predicted): FP ≥ 80%; FP+SAL > 80%<br><br>exacerbation: FP = 8; FP+SAL = 3	parallel groups<br>double-blind	12	FP 200 mcg die (102)<br>FP+SAL 200/100 mcg die (101)

Study	Countries	Patients included, demographics, clinical features	Patient Characteristics	Study type Blinding	Follow up (weeks)	Interventions (participants)
Morice 2008	UK	N = 622<br>mean age (SD) = 8 (NA)<br>Females – N (%) = 212 (34)<br>Not Hispanic or Latino – N (%) = NA<br>Eczema – N (%) = NA<br>Eosinophilia – N (%) = NA<br>BL-severity (mild) – N (%) = NA	sex:<br>BUD – M = 137, F = 70;<br>BUD+FORM DPI – M = 141, F = 71;<br>BUD+FORM pMDI – M = 132, F = 71<br>mean age: BUD = 9; BUD+FORM DPI = 8; BUD+FORM pMDI = 8 (no SD)<br>asthma severity (FEV1% predicted): BUD = 87; BUD+FORM DPI = 89; BUD+FORM pMDI = 89<br><br>The mean change of FEV1 (L) is in a graph.<br><br>exacerbation: BUD = 13, BUD+FORM DPI = 7, BUD+FORM pMDI = 7 (asthma aggravated)	parallel groups double-blind	12	budesonide pMDI 400 mcg die (207)<br>budesonide+formoterol DPI 320/18 mcg die (212)<br>budesonide+formoterol pMDI 320/18 mcg die (203)
Russell 1995	UK	N = 206<br>mean age (SD) = 10.2 (2.7)<br>Females – N (%) = 82 (40)<br>Not Hispanic or Latino – N (%) = NA<br>Eczema – N (%) = NA<br>Eosinophilia – N (%) = NA<br>BL-severity (mild) – N (%) = NA	sex: ICS+LABA – M = 59, F = 40; ICS – M = 65, F = 42<br>mean age: ICS+LABA = 10.2 (SD = 2.7); ICS = 10.3 (SD = 2.7)<br><br>exacerbation (asthma-related adverse events): ICS+LABA = 10; ICS = 13	parallel groups double-blind	12	ICS (beclomethasone or budesonide) + salmeterol 50 mcg BID (99)<br>ICS (beclomethasone or budesonide) + placebo (107)<br><br>ICS dose from 400 to 2,400 mcg die; the average dose was 750 mcg
Shapiro 2001	USA	N = 274<br>mean age (SD) = 12.1 (2.8)<br>Females – N (%) = 96 (35)<br>Not Hispanic or Latino – N (%) = NA<br>Eczema – N (%) = NA<br>Eosinophilia – N (%) = NA<br>BL-severity (mild) – N (%) = NA	sex: BUD 200 – M = 55, F = 35; BUD 400 – M = 66, F = 27; placebo – M = 57, F = 34<br>mean age:<br>BUD 200 = 12.1 (SD = 2.8); BUD 400 = 12.1 (SD = 2.8); placebo = 12.1 (SD = 2.8)<br>race:<br>BUD 200 – Caucasian = 75; African American = 10; Asian = 4; Other = 1<br>BUD 400 – Caucasian = 85; African American = 6; Asian = 0; Other = 2<br>placebo – Caucasian = 83; African American = 6; Asian = 2; Other = 0<br>BL_FEV1 (L): BUD 200 = 2.1; BUD 400 = 2.1; placebo = 2.1<br><br>exacerbation (aggravated asthma): BUD 200 = 9; BUD 400 = 8; placebo = 10<br>Some patients used triamcinolone (N=107) and flunisolide (N=23) at entry.	parallel groups double-blind	12	BUD 200 mcg die Turbuhaler (90)<br>BUD 400 mcg die Turbuhaler (93)<br>placebo (91)
Simons 2001[1]	Argentina, Australia, Austria, Brazil, Canada, France, Germany, Greece, Norway, Portugal, Sweden, The Netherlands, Russia, Turkey	N = 279<br>mean age (SD) = 10.4 (2.2)<br>Females – N (%) = 92 (33)<br>Not Hispanic or Latino – N (%) = 17 (6)<br>Eczema – N (%) = NA<br>Eosinophilia – N (%) = NA<br>BL-severity (mild) – N (%) = NA	mean age: 10.4 (SD = 2.2)<br>sex: F = 92; M = 187<br>ethnicity: 83% were white, 10% were Asian, 6% were Hispanic, and 1% were members of other ethnic groups.<br>exacerbation (asthma worsening - AEs): BUD = 35/270; BUD+LTRA = 32/277<br>Some patients used triamcinolone and flunisolide at entry. First period data not available.	crossover double-blind	P1: 4<br>P2: 4<br>P3: 4<br><br>no washout	BUD 400 mcg die (270)<br>BUD 400 mcg die + montelukast 5 mg OD (277)
Strauch 2003	Germany	N = 25<br>mean age (SD) = 10 (NA)<br>Females – N (%) = 9 (36)<br>Not Hispanic or Latino – N (%) = NA<br>Eczema – N (%) = NA<br>Eosinophilia – N (%) = NA<br>BL-severity (mild) – N (%) = NA	sex: 16 M; 9 F<br>age (IPD): table 1 (no indication of the treatment group)<br>asthma severity (FEV1 % predicted): table 1 (IPD) (no indication of the treatment group); table 2 (median)<br>overall QoL (median, 95%CI) (PAQLQ; cores are expressed as the mean score per item): placebo – 7.0 (5.0 –7.0); montelukast – 7.0 (6.0 –7.0)	parallel groups double-blind	4	ICS (400-800 mcg BUD die) + montelukast 5 mg<br>ICS (400-800 mcg BUD die) + placebo
Tal 2002	Czech Republic, Belgium, Hungary, Israel, South Africa, Spain, UK	N = 286<br>mean age (SD) = 11 (NA)<br>Females – N (%) = 109 (38)<br>Not Hispanic or Latino – N (%) = NA<br>Eczema – N (%) = NA<br>Eosinophilia – N (%) = NA<br>BL-severity (mild) – N (%) = NA	sex: ICS+LABA – M = 90, F = 58; ICS – M = 87, F = 51<br>mean age: ICS+LABA = 11; ICS = 11 (no SD)<br>asthma severity: ICS+LABA = 74; ICS = 76<br>mean FEV1 (L): ICS+LABA = 2.01; ICS = 1.91 (no SD)<br><br>exacerbation (asthma aggravated): ICS+LABA = 8; ICS = 4;	parallel groups double-blind	12	budesonide/formoterol 320/18 mcg die (148)<br>budesonide 400 mcg die (138)

Study	Countries	Patients included, demographics, clinical features	Patient Characteristics	Study type Blinding	Follow up (weeks)	Interventions (participants)
Vermeulen 2007[2]	Hungary, Poland, Serbia/Montenegro, South Africa, Spain	N = 403<br>mean age (SD) = NA<br>Females – N (%) = 131 (33)<br>Not Hispanic or Latino – N (%) = NA<br>Eczema – N (%) = NA<br>Eosinophilia – N (%) = NA<br>BL-severity (mild) – N (%) = NA	sex: CIC – M = 192, F = 80; ICS – M = 80, F = 51<br>age: no mean, only the median<br>asthma severity: CIC = 73.2; ICS = 73.1<br>BL FEV1 (mL): CIC = 2310 (2.31 L) (N=270); ICS = 2310 (2.31 L) (N=130)<br>FEV1 (mL): CIC = 2815 (2.82 L) (N=270); ICS = 2846 (2.85 L) (N=130)<br><br>exacerbation: CIC = 7; ICS = 2	parallel groups double-blind	12	ciclesonide (320 mcg OD) (272)<br>budesonide (800 mcg OD) (31)<br><br>randomization 2 (CIC):1 (BUD)
Visitsunthorn 2011	Thailand	N = 29<br>mean age (SD) = 9 (1)<br>Females – N (%) = 6 (21)<br>Not Hispanic or Latino – N (%) = NA<br>Eczema – N (%) = 29 (100)<br>Eosinophilia – N (%) = NA<br>BL-severity (mild) – N (%) = 25 (86)	sex: ICS+placebo – M = 13, F = 2; ICS+LTRA – M = 10, F = 4<br>age: ICS+placebo = 9.1 (SD = 1.1); ICS+LTRA = 8.9 (SD = 0.9)<br>eczema: all patients<br>asthma severity: ICS+placebo – mild = 14, moderate = 1; ICS+LTRA – mild = 11, moderate = 3<br>phenotype: ICS+placebo = 566.34 (eosinophilic); ICS+LTRA = 706.87 (cells)(eosinophilic)<br>FEV1 (L): ICS+placebo = 1.38; ICS+LTRA = 1.43<br>BL FEV1 (L): ICS+placebo = 1.42; ICS+LTRA = 1.31	crossover double-blind	P1: 6<br>washout: 2<br>P2: 6	ICS+placebo (ICS unknown dose) (15)<br>ICS+montelukast (14)
Zimmerman 2004	Canada	N = 302<br>mean age (SD) = 8.7 (NA)<br>Females – N (%) = 114 (38)<br>Not Hispanic or Latino – N (%) = NA<br>Eczema – N (%) = NA<br>Eosinophilia – N (%) = NA<br>BL-severity (mild) – N (%) = NA	sex:<br>ICS → M = 65, F =36;<br>ICS+LABA 4.5 mcg → M = 65, F = 41;<br>ICS+LABA 9 mcg → M = 58, F = 37<br>mean age: ICS = 9; ICS+LABA 4.5 mcg = 8; ICS+LABA 9 mcg = 9 (no SD)<br>asthma severity: ICS = 77.2; ICS+LABA 4.5 mcg = 78.3; ICS+LABA 9 mcg = 77.5<br>BL FEV1 (L): ICS = 1.49; ICS+LABA 4.5 mcg = 1.53; ICS+LABA 9 mcg = 1.50<br>FEV1 (L): ICS = 1.61; ICS+LABA 4.5 mcg = 1.71; ICS+LABA 9 mcg = 1.68<br><br>exacerbation: ICS = 11; ICS+LABA 4.5 mcg = 5; ICS+LABA 9 mcg = 6 (asthma aggravated)	parallel groups double-blind	12	ICS + placebo (101)<br>ICS + formoterol 4.5 mcg BID (106)<br>ICS + formoterol 9 mcg BID (95)<br><br>ICS dose is unknown

1 trial could not be included in analyses as aggregate data for the first period were not presented in the publication
2 trial could not be included in analyses as no comparison could be made when treatment groups considered at the treatment class level

Table S5. Eligible studies without individual participant data or aggregate data (parts 1 to 18)

First author (Year)	Sponsor	Study Reference	Reasons for not extracting AgD	Study	Total randomized participants (age range)	Total randomized children／adolescents*	Treatments (number of participants reported)	Outcome(s) reported in the publication (does not imply adequate AgD)
Abbas (2016)	—	Abbas, A.; Maheshwari, M. P.; Siddiqui, Z. A.; Maheshwari, R. R. Role of long acting beta2 agonist salmeterol, in management of mild to moderate asthmatic patients. Pakistan Journal of Medical and Health Sciences 2016;10(4):1112-1115	population of both adults and adolescents	parallel groups	50 (15-65)	not possible to establish	salmeterol 50 mcg and fluticasone propionate 250 mcg twice daily (24)<br>beclomethasone dipropionate 500 mcg twice daily (23)	symptoms
Amar (2017)	MERCK	Amar NJ, Shekar T, Varnell TA, Mehta A, Philip G. Mometasone furoate (MF) improves lung function in pediatric asthma: A double-blind, randomized controlled dose-ranging trial of MF metered-dose inhaler. Pediatr Pulmonol. 2017 Mar;52(3):310-318. doi: 10.1002/ppul.23563. Epub 2016 Oct 14. Erratum in: Pediatr Pulmonol. 2019 May;54(5):655-656.	ICS or ICS+LABA at screening	parallel groups	578 (5-11)	578	mometasone furoate-MDI 50 mcg BID (120)<br>mometasone furoate-MDI 100 mcg BID (113)<br>mometasone furoate-MDI 200 mcg BID (108)<br>mometasone furoate-DPI 100 mcg QD PM (125)<br>placebo (112)	FEV1<br>QoL<br>AEs
Arama (2016) (§)	—	Marina Arama, Tatiana Gorelco, Tatiama Kuleshina (2016). Antileukotriens in management of paediatric asthma: The hormon reducing force. European Respiratory Journal 2016 48: PA1249; DOI: 10.1183/13993003.congress-2016.PA1249	congress abstract with no data	parallel groups	40 (5-15)	40	ICS+montelukast (NA)<br>ICS+placebo (NA)	symptoms<br>FEV1 (spirometry)
Arsovski (2016) (§)	—	Arsovski, Z.; Dokic, D.; Kjaeva, B.; Goseva, Z.; Pejkovska, S.; Arbutina, S.; Janeva, E. (2016). Different therapeutic response to inhaled Fluticasone propionate in smokers and non-smokers with asthma. Allergy, 71, 365-366.	congress abstract with no data	parallel groups	38 (NA)	not possible to establish	fluticasone propionate 250 mcg BID in smokers and non-smokers	asthma control<br>FEV1
Bensch (2002)	Novartis	Bensch G, Berger WE, Blokhin BM, Socolovsky AL, Thomson MH, Till MD, Castellsague J, Della Cioppa G; International Study Group on Foradil Evaluation in Pediatric Asthma. One-year efficacy and safety of inhaled formoterol dry powder in children with persistent asthma. Ann Allergy Asthma Immunol. 2002 Aug;89(2):180-90.	not only ICS alone at screening	parallel groups	518 (5-12)	518	formoterol 12 mcg BID (171)<br>formoterol 24 mcg BID (171)<br>placebo (176)	FEV1<br>AEs
Berger (2010)	AstraZeneca	Berger WE, Leflein JG, Geller DE, Parasuraman B, Miller CJ, O'Brien CD, O'Dowd L. The safety and clinical benefit	LABA too at screening	parallel groups	187 (6-11)	187	budesonide/formoterol pMDI 320/9 mcg BID (124)<br>budesonide DPI 400 µg BID (63)	FEV1<br>AEs

First author (Year)	Sponsor	Study Reference	Reasons for not extracting AgD	Study	Total randomized participants (age range)	Total randomized children ⁄ adolescents*	Treatments (number of participants reported)	Outcome(s) reported in the publication (does not imply adequate AgD)
		of budesonide/formoterol pressurized metered-dose inhaler versus budesonide alone in children. Allergy Asthma Proc. 2010 Jan-Feb;31(1):26-39. doi: 10.2500/aap.2010.31.3301.						QoL<br>symptoms
Berger (2014)	MERCK	Berger WE, Bensch GW, Weinstein SF, Skoner DP, Prenner BM, Shekar T, Nolte H, Teper AA. Bronchodilation with mometasone furoate/formoterol fumarate administered by metered-dose inhaler with and without a spacer in children with persistent asthma. Pediatr Pulmonol. 2014 May;49(5):441-50. doi: 10.1002/ppul.22850. Epub 2013 Sep 9.	ICS or ICS+LABA at screening	crossover	92 (5-11)	92	mometasone furoate/formoterol without spacer 100/10 mcg (23)<br>mometasone furoate/formoterol with spacer 100/10 mcg (23)<br>formoterol-DPI 10 mcg (23)<br>placebo (23)<br>All patients used mometasone furoate Dry Powder Inhaler (DPI) 100 mcg once daily (QD) in the evening (PM) throughout the whole study, including the treatment periods.	
Bernstein (2011)	MERCK	Bernstein DI, Hébert J, Cheema A, Murphy KR, Chérrez-Ojeda I, Matiz-Bueno CE, Kuo WL, Nolte H. Efficacy and onset of action of mometasone furoate/formoterol and fluticasone propionate/salmeterol combination treatment in subjects with persistent asthma. Allergy Asthma Clin Immunol. 2011 Dec 7;7:21. doi: 10.1186/1710-1492-7-21.	population of both adults and children/ adolescents<br>ICS or ICS+LABA at screening	parallel groups	722 (12-82)	not possible to establish	fluticasone propionate/salmeterol DPI 250/50 mcg BID (351)<br>mometasone furoate/formoterol MDI 200/10 mcg BID (371)	exacerbation<br>asthma control<br>QoL<br>symptoms<br>FEV1<br>AEs
Bernstein (2017)	TEVA	David I. Bernstein, Michael Gillespie, Sharon Song & Jonathan Steinfeld (2017). Safety, efficacy, and dose response of fluticasone propionate delivered via the novel MDPI in patients with severe asthma: A randomized, controlled, dose-ranging study, Journal of Asthma, 54:6, 559-569, DOI: 10.1080/02770903.2016.1242137	population of both adults and children/ adolescents<br>ICS or ICS+LABA at screening	parallel groups	640 (12-65+)	9	fluticasone propionate MDPI 50 mcg (107)<br>fluticasone propionate MDPI 100 mcg BID (107)<br>fluticasone propionate MDPI 200 mcg BID (106)<br>fluticasone propionate MDPI 400 mcg BID (107)<br>fluticasone propionate DPI 250 mcg BID (107)<br>placebo MDPI (106)	FEV1<br>AEs
Bernstein (2019) (§)	Unknown	David I. Bernstein — Efficacy Comparison of Mometasone Furoate/Formoterol Versus Fluticasone Propionate/Salmeterol Combination Therapies in Subjects With Persistent Asthma: noninferiority and Onset-of-Action Findings. Breast (Edinburgh, Scotland) 2019;44():S62-	not found	parallel groups	—	—	mometasone furoate/formoterol (NA)<br>fluticasone propionate/salmeterol (NA)	—
Bose (1987)	—	Bose B, Cater JI, Clark RA. A once daily theophylline preparation in prevention of nocturnal symptoms in childhood asthma. Eur J Pediatr. 1987 Sep;146(5):524-7.	other medicine used at screening	crossover	20 (5-16)	20	theophylline (OD) (20)<br>placebo (20)	symptoms<br>AEs

First author (Year)	Sponsor	Study Reference	Reasons for not extracting AgD	Study	Total randomized participants (age range)	Total randomized children ⁄ adolescents*	Treatments (number of participants reported)	Outcome(s) reported in the publication (does not imply adequate AgD)
Botan (2019)	—	Botan, V.; Miranda, M.; Couto, S.; Rocha, E.; Imaculada Muniz-Junqueira, M. Influence of Montelukast on the State of Eosinophil Activation in Asthmatic Children. Breast (Edinburgh, Scotland) 2019;44():S64-2019	different outcomes in the publication; the author confirmed to have the outcomes of interest, but after the first consensus, she no longer replied	parallel groups	83 (2-18)	83	montelukast (NA)<br>placebo (NA)<br>healthy control (NA)	none of interest
Byrnes (2000) (§)	GSK	Byrnes C, Shrewsbury S, Barnes PJ, Bush A. Salmeterol in paediatric asthma. Thorax. 2000 Sep;55(9):780-4.	control group: salbutamol<br>it is not clear if ICS treatment was maintained after the run-in	crossover	45 (5-16)	45	salmeterol 50 µg bd (45)<br>salmeterol 100 µg bd (45)<br>salbutamol 200 µg qds (45)	FEV1<br>AEs
D'Alonzo (1994)	GSK	D'Alonzo GE, Nathan RA, Henochowicz S, Morris RJ, Ratner P, Rennard SI. Salmeterol xinafoate as maintenance therapy compared with albuterol in patients with asthma. JAMA. 1994 May 11;271(18):1412-6.	population of both adults and children/adolescents<br>only 20% used ICS at screening	parallel groups	322 (NA)	not possible to establish	ICS+salmeterol 42 mcg BID (106)<br>ICS+albuterol 180 mcg 4-time day(108)<br>ICS+placebo (108)	exacerbation<br>FEV1<br>AEs
D'Urzo (2005)	MERCK	D'Urzo A, Karpel JP, Busse WW, Boulet LP, Monahan ME, Lutsky B, Staudinger H. Efficacy and safety of mometasone furoate administered once-daily in the evening in patients with persistent asthma dependent on inhaled corticosteroids. Curr Med Res Opin. 2005 Aug;21(8):1281-9.	population of both adults and children/adolescents	parallel groups	400 (12-78)	not possible to establish	mometasone furoate-DPI 200 µg qd PM (78)<br>mometasone furoate-DPI 400 µg qd PM as one inhalation (from a DPI delivering 400 µg/inhalation) (80)<br>mometasone furoate-DPI 400 µg qd PM as two inhalations (from a DPI delivering 200 µg/inhalation) (78)<br>mometasone furoate-DPI 200 µg bid (81)<br>placebo (83)	FEV1<br>symptoms<br>QoL<br>AEs
Emeryk (2016)	Mundi pharma	Emeryk, Andrzej; Klink, Rabih; McIver, Tammy; Dalvi, Prashant (2016). A 12-week open-label, randomized, controlled trial and 24-week extension to assess the efficacy and safety of fluticasone propionate/formoterol in children with asthma. Therapeutic advances in respiratory disease, 10(4), 324-37.	ICS or LABA at screening	parallel groups	211 (4-12)	211 (180 eligible)	FP/FORM 100/10 mcg BID (106)<br>FP/SAL 100/50 mcg BID (105)	FEV1<br>AEs
EudraCT number: 2014-005047-40 (§)	Sanofi	NO PUBLICATION	no publication<br>population of both adults and children/	crossover	122 (12-64)	12	salmeterol/fluticasone propionate 12.5/250 mcg via DPI PulmoJet (122)<br>salmeterol/fluticasone Propionate 50/250 mcg via DPI PulmoJet (122)	FEV1<br>AEs

First author (Year)	Sponsor	Study Reference	Reasons for not extracting AgD	Study	Total randomized participants (age range)	Total randomized children／adolescents*	Treatments (number of participants reported)	Outcome(s) reported in the publication (does not imply adequate AgD)
			adolescents				salmeterol/fluticasone Propionate 50/250 mcg Seretide Diskus (122)	
EudraCT number: 2017-004424-29-NL (PUFFIN)	—	NO PUBLICATION	still recruiting	–	–	–	–	–
Farzan (2017)	—	Farzan, Sherry; Khan, Sundas; Elera, Claudia; Tsang, James; Akerman, Meredith; DeVoti, James (2017). Effectiveness of montelukast in overweight and obese atopic asthmatics. Ann Allergy Asthma Immunol 119, 189-193.	population of both adults and children/ adolescents<br>not possible to use ACT as a binary variable	parallel groups	26 (NA)	23	ICS+montelukast (Overweight/Obese)<br>ICS+placebo (Overweight/Obese)<br>ICS+montelukast (Normal Weight)<br>ICS+placebo (Normal Weight)	asthma control
Fitzgerald (2003) (§)	AstraZeneca	JM FitzGerald, MR Sears, L-P Boulet, AB Becker, et al. Adjustable maintenance dosing with budesonide/formoterol reduces asthma exacerbations compared with traditional fixed dosing: A five-month multicentre Canadian study. Can Respir J 2003;10(8):427-434.	population of both adults and children/ adolescents<br>ICS or ICS+LABA at screening	parallel groups	995 (12-96)	not possible to establish	budesonide/formoterol (adjustable maintenance) (499)<br>budesonide/formoterol (fixed maintenance) (496)	exacerbation<br>hospitalization and health economic parameters<br>AEs
Gelfand (2006)	COVIS PHARMA	Gelfand EW, Georgitis JW, Noonan M, Ruff ME. Once-daily ciclesonide in children: efficacy and safety in asthma. J Pediatr. 2006 Mar;148(3):377-83.	ICS or leukotriene or cromones at screening	parallel groups	1031 (4-11)	1031	ciclesonide 40 mcg OD (252)<br>ciclesonide 80 mcg OD (259)<br>ciclesonide 160 mcg OD (253)<br>placebo mcg OD (254)	FEV1 (not L/s)<br>QoL<br>symptoms<br>AEs
Gustafsson (1993)	—	Gustafsson P, Tsanakas J, Gold M, Primhak R, Radford M, Gillies E. Comparison of the efficacy and safety of inhaled fluticasone propionate 200 micrograms/day with inhaled beclomethasone dipropionate 400 micrograms/day in mild and moderate asthma. Arch Dis Child. 1993 Aug;69(2):206-11.	children/ adolescent until 19<br>other medicines at screening	parallel groups	398 (4-19)	not possible to establish	fluticasone propionate 200 mcg OD (197)<br>beclometasone dipropionate 400 mcg OD (201)	exacerbation<br>FEV1<br>symptoms<br>AEs
Hampel (2017)	TEVA	Hampel FC Jr, Carr W, Gillespie M, Small CJ. (2017). Evaluation of beclomethasone dipropionate (80 and 160 micrograms/day) delivered via a breath-actuated inhaler for persistent asthma. Allergy Asthma Proc., 38(6):419-430. doi: 10.2500/aap.2017.38.4089. Epub 2017 Sep 8.	population of both adults and children/ adolescents<br>ICS and non-ICS therapy at screening	parallel groups	273 (12-65+)	30	beclometasone dipropionate BAI 80 mcg OD (90)<br>beclometasone dipropionate BAI 160 mcg OD (92)<br>placebo BAI (91)	FEV1<br>QoL<br>symptoms<br>AEs
Ikeda (2015) (§)	Kyorin pharmaceutical Co	K. Ikeda. Comparison Of Efficacy Onset And Clinical Benefit Between Formoterol/fluticasone And Salmeterol/fluticasone In Unstable	abstract with no age range<br>ICS or ICS+LABA at screening	parallel groups	21 (NA)	not possible to establish	formoterol/fluticasone combination 636 mcg per day (11)<br>salmeterol/fluticasone combination 620 mcg per day (10)	pulmonary function<br>asthma control

First author (Year)	Sponsor	Study Reference	Reasons for not extracting AgD	Study	Total randomized participants (age range)	Total randomized children ⁄ adolescents*	Treatments (number of participants reported)	Outcome(s) reported in the publication (does not imply adequate AgD)
		Chronic Asthma: An Open-Label, Randomized Study. Am J Respir Crit Care Med 191;2015:A4238						(ACQ)<br>symptoms
Ilowite (2004)	MERCK	Ilowite J, Webb R, Friedman B, Kerwin E, Bird SR, Hustad CM, Edelman JM: Addition of montelukast or salmeterol to fluticasone for protection against asthma attacks: a randomized, double-blind, multicenter study. Ann Allergy Asthma Immunol. 2004, 92 (6): 641-648	population of both adults and children/ adolescents	parallel groups	1473 (14-73)	not possible to establish	fluticasone 220 mcg + montelukast 10 mg OD (743)<br>fluticasone 220 mcg + salmeterol 84 mcg OD (730)	exacerbation (asthma attack)<br>symptoms<br>AEs
Jamaati (2015)	COVIS PHARMA	Hamidreza Jamaati, Majid Malekmohammad, Fanak Fahimi, Arvin Najafi, Seyed Mohammadreza Hashemian (2015). Efficacy of Low-Dose Ciclesonide and Fluticasone Propionate for Mild to Moderate Persistent Asthma. Tanaffos, 14(1): 1-9	population of both adults and children/ adolescents	parallel groups	230 (15-65)	not possible to establish	ciclesonide 80 mcg OD (115)<br>fluticasone propionate 100 mcg BID (115)	FEV1<br>QoL<br>asthma control<br>AEs
Jehan (2014) (§)	—	Jehan, N.; Rehman, M. U.; Zarkoon, M. H. To determine the efficacy of inhaled corticosteroids compared to montelukast in reducing exacerbation in uncontrolled asthma in children 6 months to 5 years. Pakistan Journal of Medical and Health Sciences 2014;8(3):662-666 Pakistan Lahore Medical And Dental College (Tulspura, North Canal Bank, Lahore, Pakistan. E-mail: prof_abdulmajeed@hotmail.com) 2014	recruitment at the emergency room and no indication of previous treatment patients were given ICS and tab Montelukast by lottery method to remove the bias	parallel groups	2400 (6 months-5 years)	2400	ICS 200 mcg die (1200)<br>montelukast 4 or 5 mg die (1200)	exacerbation
Kerwin (2017)	TEVA	E. M. Kerwin, G. Yiu, L. Hickey, C. J. Small. Analysis Of The Relationship Between Handheld And Clinic-Based Spirometry Measurements In A Randomized, Double-Blind, Placebo-Controlled Study Of Beclomethasone Dipropionate Via Breath-Actuated Inhaler For Persistent Asthma. Am J Respir Crit Care Med 2017;195:A3205	population of both adults and children/ adolescents<br>only abstract	parallel groups	425 (12-NA)	not possible to establish	beclomethasone dipropionate (BAI) 40 mcg/inhalation x 4 inhalations twice daily (BID) (320 mcg/day)<br>beclomethasone dipropionate (BAI) 80 mcg/inhalation x 4 inhalations twice daily (BID) (640 mcg/day)<br>beclomethasone dipropionate (MDI) 40 mcg/inhalation x 4 inhalations BID (320 mcg/day)<br>placebo BAI<br>placebo MDI	FEV1

First author (Year)	Sponsor	Study Reference	Reasons for not extracting AgD	Study	Total randomized participants (age range)	Total randomized children ⁄ adolescents*	Treatments (number of participants reported)	Outcome(s) reported in the publication (does not imply adequate AgD)
Knorr (1998)	MERCK	Knorr B, Matz J, Bernstein JA, Nguyen H, Seidenberg BC, Reiss TF, Becker A. Montelukast for chronic asthma in 6- to 14-year-old children: a randomized, double-blind trial. Pediatric Montelukast Study Group. JAMA. 1998 Apr 15;279(15):1181-6. doi: 10.1001/jama.279.15.1181. PMID: 9555757.	only 20-24% of patients used ICS at screening	parallel groups	336 (6-15)	72	montelukast 5 mg OD (201)<br>placebo (135)	FEV1<br>AEs
Knorr (2001)	MERCK	Knorr B, Franchi LM, Bisgaard H, Vermeulen JH, LeSouef P, Santanello N, Michele TM, Reiss TF, Nguyen HH, Bratton DL. Montelukast, a leukotriene receptor antagonist, for the treatment of persistent asthma in children aged 2 to 5 years. Pediatrics. 2001 Sep;108(3):E48. doi: 10.1542/peds.108.3.e48. PMID: 11533366.	up to 50% of patients used inhaled or nebulized corticosteroids or cromolyn at screening and during the study	parallel groups	689 (2-6)	56	montelukast 4 mg (461)<br>placebo (228)	asthma control<br>symptoms<br>QoL<br>AEs
Kunoe (2016) (§)	—	Kunoe, A.; Agertoft, L.; Chawes, B. L.; Bonnelykke, K.; Bisgaard, H.; Pedersen, S. Early intervention with high-dose inhaled corticosteroids for preschool wheezing does not improve lung function at school age. Allergy: European Journal of Allergy and Clinical Immunology 2016;71(Supplement 102):365	poster – no information on the pre-study treatment (perhaps, naïve) *"a trial to investigate if use of high-dose inhaled corticosteroids for preschool wheezing improves lung function at 6 years of age"*	parallel groups	220 (6–35 months)	220	fluticasone propionate 1000 mcg/day pMDI (112)<br>placebo (108)	FEV1
Langton Hewer (1995)	—	Langton Hewer S, Hobbs J, French D, Lenney W. Pilgrim's progress: the effect of salmeterol in older children with chronic severe asthma. Respir Med. 1995 Jul;89(6):435-40.	34.8% of patients used OC and other medicine besides ICS at screening	parallel groups	24 (12-17)	23	ICS (range 50-1000 mcg BID) + salmeterol 100 mcg BID (11)<br>ICS (range 50-1000 mcg BID) + placebo (12)	exacerbation<br>FEV1<br>symptoms<br>AEs
Lin (2015) (IPD supplied)	GSK	Lin J, Kang J, Lee SH, Wang C, Zhou X, Crawford J, Jacques L, Stone S. Fluticasone furoate/vilanterol 200/25 mcg in Asian asthma patients: a randomized trial. Respir Med. 2015 Jan;109(1):44-53. doi:	population of both adults and children/ adolescents<br>all eligible participants	parallel groups	309 (13-79)	0	fluticasone furoate/vilanterol 200/25 mcg OD (155)<br>fluticasone propionate 500 mcg BID (154)	ACT<br>exacerbation<br>FEV1<br>symptoms<br>QoL<br>AEs

First author (Year)	Sponsor	Study Reference	Reasons for not extracting AgD	Study	Total randomized participants (age range)	Total randomized children / adolescents*	Treatments (number of participants reported)	Outcome(s) reported in the publication (does not imply adequate AgD)
		10.1016/j.rmed.2014.10.012. Epub 2014 Oct 31.	were using ICS+LABA at screening					
Lin (2016) (IPD supplied)	GSK	Lin J, Tang H, Chen P, Wang H, Kim MK, Crawford J, Jacques L, Stone S. Efficacy and safety evaluation of once-daily fluticasone furoate/vilanterol in Asian patients with asthma uncontrolled on a low- to mid-strength inhaled corticosteroid or low-dose inhaled corticosteroid/long-acting beta2-agonist. Allergy Asthma Proc. 2016 Jul;37(4):302-10. doi: 10.2500/aap.2016.37.3968.	population of both adults and children/ adolescents only one participant was using ICS alone at screening	parallel groups	307 (14-79)	1	fluticasone furoate/vilanterol 100/25 mcg OD (153)<br>placebo (154)	ACT<br>exacerbation<br>FEV1<br>symptoms<br>QoL<br>AEs
Mallol (2016)	COVIS PHARMA	J. Mallol, V. Aguirrea, A. Gallardoa, E. Corteza, C. Sáncheza, C. Riquelmea, P. Córdovaa, M. Martíneza, A. Galindob. Effect of once-daily generic ciclesonide on exhaled nitric oxide in atopic children with persistent asthma. Allergologia et immunopathologia 2016;44(2):106-12	1) not possible to use ACT as a binary variable; 2) not possible to classify ICS dose based on age for the secondary analysis	parallel groups	60 (7-15)	60	ciclesonide 80 mcg OD (27)<br>ciclesonide 160 mcg OD (29)	ACT<br>AEs
Mansfield (2017)	TEVA	Mansfield L, Yiu G, Sakov A, Liu S, Caracta C. A 6-month safety and efficacy study of fluticasone propionate and fluticasone propionate/salmeterol multidose dry powder inhalers in persistent asthma. Allergy Asthma Proc. 2017 Jul 24;38(4):264-276. doi: 10.2500/aap.2017.38.4061. Epub 2017 May 24.	population of both adults and children/ adolescents ICS or ICS+LABA at screening	parallel groups	674 (12-65+)	73	fluticasone propionate MDPI 100 mcg BID (127)<br>fluticasone propionate HFA 220 mcg BID (42)<br>fluticasone propionate MDPI 200 mcg BID (126)<br>fluticasone propionate HFA 440 mcg BID (41)<br>fluticasone propionate/salmeterol MDPI 100/12.5 mcg BID (120)<br>fluticasone propionate/salmeterol DPI 250/50 mcg BID (41)<br>fluticasone propionate/salmeterol MDPI 200/12.5 mcg BID (133)<br>fluticasone propionate/salmeterol DPI 500/50 mcg BID (44)	FEV1<br>AEs
Maspero (2010)	MERCK	Maspero JF, Nolte H, Chérrez-Ojeda I; P04139 Study Group. Long-term safety of mometasone furoate/formoterol combination for treatment of patients with persistent asthma. J Asthma. 2010 Dec;47(10):1106-15. doi: 10.3109/02770903.2010.514634. Epub 2010 Nov 1. Erratum in: J Asthma. 2011 Feb;48(1):114.	population of both adults and children/ adolescents ICS or ICS+LABA at screening	parallel groups	404 (NA)	not possible to establish	mometasone furoate/formoterol 200/10 mcg (141)<br>fluticasone propionate/salmeterol 250/50 mcg (68)<br>mometasone furoate/formoterol 400/10 mcg (130)<br>fluticasone propionate/salmeterol 500/50 mcg (65)	AEs<br>FEV1<br>symptoms

First author (Year)	Sponsor	Study Reference	Reasons for not extracting AgD	Study	Total randomized participants (age range)	Total randomized children ⁄ adolescents*	Treatments (number of participants reported)	Outcome(s) reported in the publication (does not imply adequate AgD)
McIver (2011)	Mundipharma	McIver, T.; Emeryk, A.; Klink, R.; Schwab, B. (2011). Fluticasone propionate/formoterol fumarate (FLUT/FORM) combination therapy has comparable efficacy to fluticasone propionate/salmeterol xinafoate (FLUT/SAL) in paediatric patients with asthma. European Respiratory Journal, 38, SUPPL. 55.	likely conference abstract – no information on pre-treatment at screening	parallel groups	211 (4-12)	211	fluticasone propionate/formoterol 100/10µg BID (102)<br>fluticasone propionate/salmeterol 100/50µg BID (99)	FEV1
Meltzer (2012)	MERCK	Meltzer EO, Kuna P, Nolte H, Nayak AS, Laforce C; P04073. Study Investigators. Mometasone furoate/formoterol reduces asthma deteriorations and improves lung function. Eur Respir J. 2012 Feb;39(2):279-89. doi: 10.1183/09031936.00020310.	population of both adults and children/ adolescents<br>ICS or ICS+LABA at screening	parallel groups	746	not possible to establish	formoterol 10 mcg MDI BID (188)<br>mometasone furoate 100 mcg MDI BID (188)<br>mometasone furoate/formoterol 100/10 mcg MDI BID (182)<br>placebo (188)	exacerbation (asthma deterioration)<br>ACQ<br>FEV1<br>QoL<br>AEs
Meltzer (2019)	—	Meltzer (2019). Efficacy and Safety of Combined Mometasone Furoate/Formoterol 100/10µg Twice Daily in Subjects with Asthma Inadequately Controlled on Low-Dose Inhaled Corticosteroids. Breast (Edinburgh, Scotland) 2019;44():S63-S64	paper not found	–	–	–	–	–
Miller (2016) (§)	TEVA	David S. Miller, Gloria Yiu, Edward T. Hellriegel, and Jonathan Steinfeld (2016). Dose-ranging study of salmeterol using a novel fluticasone propionate/salmeterol multidose dry powder inhaler in patients with persistent asthma. Proc 37:291–301, 2016; doi: 10.2500/aap.2016.37.3963	population of both adults and children/ adolescents	crossover	72 (12-65+)	3	fluticasone propionate/salmeterol MDPI 100/6.25 mcg (one dose per treatment)<br>fluticasone propionate/salmeterol MDPI 100/12.5 mcg (one dose per treatment)<br>fluticasone propionate/salmeterol MDPI 100/25 mcg (one dose per treatment)<br>fluticasone propionate/salmeterol MDPI 100/50 mcg (one dose per treatment)<br>fluticasone propionate MDPI 100 mcg (one dose per treatment)<br>fluticasone propionate/salmeterol DPI 100/50mcg (one dose per treatment)	FEV1<br>AEs
Murphy (2015)	AstraZeneca	Kevin R. Murphy, Rajiv Dhand, Frank Trudo,Tom Uryniak, Ajay Aggarwal, Goran Eckerwall (2015). Therapeutic equivalence of budesonide/formoterol delivered via breath-actuated inhaler vs pMDI. Respiratory Medicine, 109, 170-179. http://dx.doi.org/10.1016/j.rmed.2014.12.009	population of both adults and children/ adolescents<br>*"Two patients receiving ICS/LABA combination therapy before study screening*	parallel groups	214 (12-75+)	21	BUD/FM BAI 320/9 mcg BID (71)<br>BUD/FM pMDI 320/9 mcg BID (71)<br>BUD pMDI 320 mcg BID (72)	FEV1<br>AEs

First author (Year)	Sponsor	Study Reference	Reasons for not extracting AgD	Study	Total randomized participants (age range)	Total randomized children ⁄ adolescents*	Treatments (number of participants reported)	Outcome(s) reported in the publication (does not imply adequate AgD)
			were not switched to mono-component ICS before run-in but were subsequently included in the study".					
Nathan (2010)	MERCK	Nathan RA, Nolte H, Pearlman DS; P04334 Study Investigators. Twenty-six-week efficacy and safety study of mometasone furoate/formoterol 200/10 microg combination treatment in patients with persistent asthma previously receiving medium-dose inhaled corticosteroids. Allergy Asthma Proc. 2010 Jul-Aug;31(4):269-79. doi: 10.2500/aap.2010.31.3364. Epub 2010 Jul 30.	population of both adults and children/ adolescents<br>ICS or ICS+LABA at screening	parallel groups	781 (NA)	not possible to establish	mometasone furoate/formoterol 200/10 µg BID (191)<br>mometasone furoate 200 µg BID (192)<br>formotero 10 µg BID (202)<br>placebo (196)	exacerbation (asthma deterioration)<br>ACQ<br>FEV1<br>QoL<br>AEs
NCT00392288 or EFC6695	COVIS PHARMA	NO PUBLICATION	no publication<br>ICS or montelukast at screening	parallel groups	501 (4-12)	501	ciclesonide MDI 40 µg BID (166)<br>ciclesonide MDI 80 µg BID (172)<br>placebo (163)	FEV1<br>symptoms
NCT00419952 or D5896C00022	AstraZeneca	NO PUBLICATION	no publication<br>population of both adults and children/ adolescents	parallel groups	742 (NA)	not possible to establish	budesonide+formoterol pMDI 160/4.5 ug x 2 actuations (twice daily) BID (377)<br>budesonide HFA pMDI 160 ug x 2 actuations (twice daily) BID (365)	exacerbation<br>symptoms<br>FEV1<br>AEs
NCT00442117 or P04880	MERCK	NO PUBLICATION	no publication<br>population of both adults and children/ adolescents	parallel groups	180 (NA)	not possible to establish	mometasone furoate DPI 200 mcg, two puffs once daily PM (total of 400 mcg/day) (85)<br>budesonide DPI DPI 200 mcg, two puffs twice daily (total of 800 mcg/day) (87)	FEV1
NCT00442559	MERCK	NO PUBLICATION	no publication<br>unknown pre-treatment	parallel groups	191 (2-14)	191	montelukast 4/5 mg tablet (oral chewable), OD (100)<br>ICS solution, 1-4 puffs daily (91)	symptoms
NCT00651768	AstraZeneca	NO PUBLICATION	no publication<br>population of both adults and children/ adolescents	parallel groups	570 (NA)	not possible to establish	budesonide/formoterol<br>Symbicort pMDI 2 X 160/4.5mcg & budesonide HFA pMDI 4 X 160mcg	exacerbation<br>lung function<br>AEs

First author (Year)	Sponsor	Study Reference	Reasons for not extracting AgD	Study	Total randomized participants (age range)	Total randomized children / adolescents*	Treatments (number of participants reported)	Outcome(s) reported in the publication (does not imply adequate AgD)
NCT01845025 (§)	Novartis	NO PUBLICATION	no publication<br>population of both adults and children/ adolescents<br>*"Use of ICS, LABA, ICS+LABA, LTRAs, leukotriene modifiers, anticholinergic, or theophylline must be discontinued prior to the first dose of investigational treatment*".	parallel groups	820 (NA)	not possible to establish	formoterol 12 mcg + fluticasone propionate 100 mcg/fluticasone propionate 250 mcg/ fluticasone propionate 500 mcg (411)<br>placebo + fluticasone propionate 100 mcg/fluticasone propionate 250 mcg/fluticasone propionate 500 mcg (409)	exacerbation<br>ACQ<br>symptoms<br>hospitalization<br>mortality<br>AEs<br>unplanned healthcare utilization
NCT02298205 (§)	Washington University School of Medicine	NO PUBLICATION	no publication<br>ICS or LTRA or ICS+LABA at screening	parallel groups	206 (6-17)	206	Provider-based adjustment: The provider will adjust the dose of Beclomethasone based on the participant's asthma control at their encounter with them<br>Asthma controller medication (Beclomethasone) adjustment strategy: The participant will adjust the dose of Beclomethasone based on symptoms	asthma control<br>exacerbation<br>FEV1<br>QoL
NCT02495168	TEVA	NO PUBLICATION	no publication<br>population of both adults and children/ adolescents	parallel groups	1714 (12-75)	not possible to establish	generic budesonide/formoterol − 2 inhalations BID (80/4.5 mcg) pMDI (501)<br>Symbicort budesonide/formoterol − 2 inhalations BID (80/4.5 mcg) pMDI (514)<br>placebo (126)	FEV1
NCT02577497	University of Virginia	NO PUBLICATION	no publication<br>ICS and/or an anti-leukotriene at screening	crossover	31 (6-17)	31	beclomethasone (31)<br>fluticasone (31)	none of interest
NCT02649478	HIKMA	NO PUBLICATION	no publication<br>population of both adults and children/ adolescents<br>ICS with or without LABA, LTRA, theophylline	parallel groups	1430	not possible to establish	fluticasone / salmeterol 100/50 mcg (NA)<br>Advair Diskus 100/50 mcg (NA)<br>placebo (NA)	FEV1<br>AEs

First author (Year)	Sponsor	Study Reference	Reasons for not extracting AgD	Study	Total randomized participants (age range)	Total randomized children ⁄ adolescents*	Treatments (number of participants reported)	Outcome(s) reported in the publication (does not imply adequate AgD)
NCT02680561 (§)	TEVA	NO PUBLICATION	no publication	crossover	20 (4-11)	20	fluticasone propionate MDPI (20)<br>fluticasone propionate/salmeterol MDPI (20)<br>fluticasone propionate/salmeterol (20)	AEs
NCT02758873	University of Sussex	NO PUBLICATION	no publication<br>ICS with/without second line controller (i.e. LABA/LTRA) at screening	parallel groups	241 (12-18)	not possible to establish	salmeterol (NA)<br>montelukast (NA)<br>standard care (NA)	ACQ<br>QoL
NCT03096327	PharmEvo Pvt Ltd	NO PUBLICATION	no publication<br>population of both adults and children/ adolescents	parallel groups	180 (NA)	not possible to establish	montelukast 4-10 mg (NA)<br>placebo (NA)	QoL<br>AEs
NCT03248128 or 107116A	GSK	NO PUBLICATION	recruiting	parallel groups	870 (5-17)	870	fluticasone furoate/vilanterol 50 or 100/25 mcg DPI (NA)<br>fluticasone furoate 50 or 100 mcg DPI (NA)	exacerbation<br>ACQ<br>FEV1<br>symptoms<br>AEs
NCT03387241	Mundipharma	NO PUBLICATION	no publication / no plan to share IPD<br>population of both adults and children/ adolescents	parallel groups	330 (12-75)	not possible to establish	fluticasone/formoterol<br>fluticasone/ salmeterol	FEV1<br>asthma control (ACQ)<br>exacerbation
NCT03535870	HIKMA	NO PUBLICATION	no publication / no plan to share IPD<br>population of both adults and children/ adolescents<br>ICS with or without LABA/LTM at screening	parallel groups	1556 (12-65)	not possible to establish	fluticasone propionate/salmeterol 100/50 mcg DPI<br>Advair Diskus, 100/ 50 mcg DPI<br>Placebo	FEV1
NCT03676413 (§)	Respirent Pharmaceuticals	NO PUBLICATION	no publication / no plan to share IPD<br>population of	parallel groups	451 (NA)	not possible to establish	fluticasone propionate/salmeterol 100/50 mcg DPI BID<br>ADVAIR DISKUS® 100/50 mcg DPI BID<br>placebo	FEV1<br>AEs

First author (Year)	Sponsor	Study Reference	Reasons for not extracting AgD	Study	Total randomized participants (age range)	Total randomized children ⁄ adolescents*	Treatments (number of participants reported)	Outcome(s) reported in the publication (does not imply adequate AgD)
			both adults and children/ adolescents<br>ICS and LABA at screening					
NCT03756883	TEVA	NO PUBLICATION	no publication / no plan to share IPD<br>population of both adults and children/ adolescents	parallel groups	999 (12-75)	not possible to establish	fluticasone propionate/salmeterol DPI 100/50 mcg (485)<br>ADVAIR DISKUS® 100/50 (fluticasone propionate and salmeterol) DPI (413)<br>placebo (101)	FEV1
NCT03847896	Bond Avillion 2 Development LP	NO PUBLICATION	no publication / no plan to share IPD<br>population of both adults and children/ adolescents<br>ICS+SABA or SABA alone at screening	parallel groups	1001 (NA)	not possible to establish	budesonide/albuterol sulfate metered-dose inhaler 80/180 mcg (NA)<br>budesonide/albuterol sulfate metered-dose inhaler 160/180 mcg (NA)<br>budesonide metered-dose inhaler 160 mcg (NA)<br>albuterol sulfate metered-dose inhaler 180 mcg (NA)<br>placebo (NA)	FEV1<br>ACQ
Nielsen (2000)	AstraZeneca	Nielsen KG, Bisgaard H. The effect of inhaled budesonide on symptoms, lung function, and cold air and methacholine responsiveness in 2- to 5-year-old asthmatic children. Am J Respir Crit Care Med 2000;162:1500–1506.	ICS or other medicines (SABA as needed, LABA, sodio cromoglycate - 4 patients, 11%) at entry	parallel groups	38 (2-5)	34	budesonide (19)<br>placebo (19)	symptoms
Pearlman (2011)	SkyePharma AG	Pearlman, D. S.; La-Force, C.; Kaiser, K. Fluticasone propionate/formoterol fumarate combination therapy has superior efficacy to both fluticasone and formoterol alone European Respiratory Journal 2011;38(SUPPL. 55): European Respiratory Society 2011	population of both adults and children/ adolescents<br>congress abstract, the author is retired	parallel groups	357 (NA)	not possible to establish	fluticasone/formoterol 100/10 mcg BID (in a single inhaler) (NA)<br>fluticasone 100 mcg BID (NA)<br>formoterol 10 mcg BID (NA)	FEV1
Pearlman (2017)	AstraZeneca	David S. Pearlman, Göran Eckerwall, Julie McLaren, Rosa Lamarca, Margareta Puu, Ileen Gilbert, Carin Jorup, Kristina Sandin, Miguel J. Lanz. Efficacy and safety of budesonide/formoterol pMDI vs budesonide pMDI in asthmatic children (6-<12 years). Annals of allergy, asthma & immunology : official publication of the	ICS or ICS+LABA at screening	parallel groups	279 (6-11)	137	budesonide/formoterol pMDI 160/9 mcg BID (92)<br>budesonide/formoterol pMDI 160/4.5 mcg BID (95)<br>budesonide pMDI 160 mcg BID (92)	exacerbation<br>FEV1<br>symptoms<br>QoL<br>AEs

First author (Year)	Sponsor	Study Reference	Reasons for not extracting AgD	Study	Total randomized participants (age range)	Total randomized children ⁄ adolescents*	Treatments (number of participants reported)	Outcome(s) reported in the publication (does not imply adequate AgD)
		American College of Allergy, Asthma, & Immunology 2017;118(4):489-499.e1						
Pearlman (2019)	—	Pearlman, D.; Nathan, R.; Meltzer, E.; Nolte, H.; Weinstein, S. Effect of Mometasone Furoate/Formoterol Combination Therapy on Nocturnal Awakenings in Subjects With Persistent Asthma. Breast (Edinburgh, Scotland) 2019;44():S63-2019	author retired and paper not found	–	–	–	–	–
Peden (1998)	GSK	Peden DB, Berger WE, Noonan MJ, Thomas MR, Hendricks VL, Hamedani AG, Mahajan P, House KW. Inhaled fluticasone propionate delivered by means of two different multidose powder inhalers is effective and safe in a large pediatric population with persistent asthma. J Allergy Clin Immunol. 1998 Jul;102(1):32-8.	ICS or cromolyn or LABA alone at screening	parallel groups	437 (4-11)	437	fluticasone propionate 50 mcg BID Diskus (90)<br>fluticasone propionate 100 mcg BID Diskus (87)<br>fluticasone propionate 50 mcg BID Diskhaler (91)<br>fluticasone propionate 100 mcg BID Diskhaler (83)<br>placebo (86)	FEV1<br>symptoms<br>AEs
Pedersen (2009)	COVIS PHARMA	Pedersen S, Engelstätter R, Weber HJ, Hirsch S, Barkai L, Emeryk A, Weber H, Vermeulen J. Efficacy and safety of ciclesonide once daily and fluticasone propionate twice daily in children with asthma. Pulm Pharmacol Ther. 2009 Jun;22(3):214-20. doi: 10.1016/j.pupt.2008.12.013. Epub 2008 Dec 27.	ICS and non-ICS at screening	parallel groups	744 (6-11)	366	ciclesonide 80 mcg OD (252)<br>ciclesonide 160 mcg OD (242)<br>fluticasone propionate 88 mcg BID (250)	FEV1<br>symptoms<br>QoL<br>AEs
Pedersen (2017)	COVIS PHARMA	Søren E Pedersen, Niyati Prasad, Udo-Michael Goehring, Henrik Andersson, Dirkje S Postma. Control of moderate-to-severe asthma with randomized ciclesonide doses of 160, 320 and 640 mug/day. Journal of Asthma and Allergy 2017;10():35-46	population of both adults and children/ adolescents	parallel groups	367 (12-70)	not possible to establish	ciclesonide 160 mcg/day (120)<br>ciclesonide 320 mcg/day (122)<br>ciclesonide 640 mcg/day (125)	FEV1<br>ACQ<br>AEs
Pertseva (2012)	—	Efficacy and safety of fluticasone/formoterol compared to fluticasone alone in patients with asthma. European Respiratory Journal 2012;40(SUPPL. 56): European Respiratory Society 2012 (CONGRESS)	congress abstract population of both adults and children/ adolescents	parallel groups	438 (NA)	not possible to establish	fluticasone propionate/formoterol 250/10 mcg BID pMDI (146)<br>fluticasone 250/10 mcg BID (146) SkyePharma pMDI<br>fluticasone 250/10 mcg BID (146) GSK pMDI	FEV1
Peters (2016)	AstraZeneca	Stephen P. Peters, Eugene R. Bleecker, Giorgio W. Canonica, Yong B. Park, Ricardo Ramirez, Sally Hollis, Harald Fjallbrant, Carin Jorup, and Ubaldo J. Martin. Serious Asthma Events with	population of both adults and children/	parallel groups	11693 (12-65+)	1268	budesonide–formoterol 80/4.5 mcg BID (1645)<br>budesonide 80 mcg BID (1646)<br>budesonide–formoterol 160/4.5 mcg BID (4201)<br>budesonide 160 mcg BID (4201)	exacerbation<br>ACQ<br>AEs

First author (Year)	Sponsor	Study Reference	Reasons for not extracting AgD	Study	Total randomized participants (age range)	Total randomized children / adolescents*	Treatments (number of participants reported)	Outcome(s) reported in the publication (does not imply adequate AgD)
		Budesonide plus Formoterol vs. Budesonide Alone. The New England journal of medicine 2016;375(9):850-60	adolescents ICS or ICS+LABA at screening					
Petnak (2016)(§)	—	Petnak, T.; Pornsuriysak, P.; Boonsarngsuk, V.; Amornputtisathaporn, N.; Kawamatawong, T. Effect of inhaled mometasone/formoterol vs inhaled fluticasone/salmeterol on peripheral airway function in asthma patients: a randomized open label trial. Chest 2016;150(4):16A-2016	no age range (likely naïve)	parallel groups	50	not possible to establish	mometasone/formoterol (25)<br>fluticasone/salmeterol (25)	none of interest
Philip (2011)	MERCK	Philip G, Villarán C, Shah SR, Vandormael K, Smugar SS, Reiss TF. The efficacy and tolerability of inhaled montelukast plus inhaled mometasone compared with mometasone alone in patients with chronic asthma. J Asthma. 2011 Jun;48(5):495-502. doi: 10.3109/02770903.2011.573042. Epub 2011 May 5.	population of both adults and children/ adolescents not only ICS alone at screening (ICS+LABA and montelukast: 35%)	crossover	134 (15-74)	not possible to establish	montelukast 1 mg + mometasone 220 µg (delivered by separate dry powder inhalers) OD (66 - first period)<br>placebo + mometasone 220 µg OD (68 - first period)	exacerbation<br>asthma control<br>FEV1<br>AEs
Phipatanakul (2003)	MERCK	Phipatanakul W, Greene C, Downes SJ, Cronin B, Eller TJ, Schneider LC, Irani AM. Montelukast improves asthma control in asthmatic children maintained on inhaled corticosteroids. Ann Allergy Asthma Immunol. 2003 Jul;91(1):49-54.	no useful data in the article	two-period parallel groups	36 (6-14)	36	ICS+montelukast (run-in dose/5 mg) (19)<br>ICS+placebo (run-in dose) (17)	none of interest
Płoszczuk (2018)	Mundipharma	Anna Płoszczuk, Miroslava Bosheva, Kay Spooner, Tammy McIver and Sanjeeva Dissanayake (2018). Efficacy and safety of fluticasone propionate/formoterol fumarate in pediatric asthma patients: a randomized controlled trial. Ther Adv Respir Dis, 12: 1–15. DOI: 10.1177/1753466618777924	ICS (uncontrolled asthma) or ICS+LABA (controlled asthma) at screening	parallel groups	512 (5-12)	379	fluticasone propionate/formoterol pMDI 100/10 mcg BID (169)<br>fluticasone propionate pMDI 100 mcg BID (173)<br>fluticasone/salmeterol pMDI 100/50 mcg BID (170)	exacerbation<br>FEV1<br>QoL<br>asthma control<br>AEs
Pohunek (2006)	AstraZeneca	Pohunek P, Kuna P, Jorup C, De Boeck K. Budesonide/formoterol improves lung function compared with budesonide alone in children with asthma. Pediatr Allergy Immunol 2006;17:458–465.	ICS (any brand) or ICS+LABA or LABA at screening	parallel groups	630 (4-11)	630	budesonide/formoterol (Symbicort) 80/4.5 mcg, two inhalations BID (216)<br>budesonide (Pulmicort) 100 mcg, two inhalations BID (213)<br>budesonide, 100 mcg, two inhalations BID (Pulmicort) + formoterol 4.5 mcg, two inhalations BID (Oxis) (201)	FEV1<br>QoL<br>AEs

First author (Year)	Sponsor	Study Reference	Reasons for not extracting AgD	Study	Total randomized participants (age range)	Total randomized children / adolescents*	Treatments (number of participants reported)	Outcome(s) reported in the publication (does not imply adequate AgD)
Pohunek (2014)	Chiesi Farmaceutici	Pohunek, P.; Scuri, M.; Reznichenko, Y.; Varoli, G.; Mokia-Serbina, S.; Baronio, R.; Brzostek, J.; Kaczmarek, J. Bronchodilating effects of extrafine beclometasone dipropionate and formoterol fumarate via pressurized metered dose inhaler in asthmatic children. Pediatric pulmonology 2014;49(SUPPL. 37):S55 Wiley-Liss Inc. 2014	abstract	crossover	56 (5-12)	56	BDP /FF 100/12 mcg (CHF1535)<br>BDP pMDI 100 mcg + FF 12 mcg pMDI	FEV1<br>AEs
Rani (2016)	—	Rani, S.; Rawal, M.; Kumar, S.; Lamba, S. To compare efficacy and safety of fixed drug combination of salmeterol / fluticasone and budesonide / formoterol on the lung functions in childhood patients with moderate persistent asthma. Indian Journal of Public Health Research and Development 2016;7(4):203-207	abstract (no data or enough information)	parallel groups	68 (NA)	68	salmeterol/fluticasone (NA)<br>budesonide/formoterol (NA)	FEV1
Raphael (2018)	TEVA	Raphael G, Yiu G, Sakov A, Liu S, Caracta C. Randomized, double-blind trial evaluating the efficacy and safety of fluticasone propionate and fluticasone propionate/salmeterol delivered via multidose dry powder inhalers in patients with persistent asthma aged 12 years and older. J Asthma. 2018 Jun;55(6):640-650. doi: 10.1080/02770903.2017.1350971.	population of both adults and children/ adolescents<br>ICS or ICS+LABA at screening	parallel groups	625 (12-65+)	86	fluticasone propionate 50 mcg DPI BID (125)<br>fluticasone propionate 100 mcg DPI BID (125)<br>fluticasone propionate/salmeterol 50/12.5 DPI BID (125)<br>fluticasone propionate/salmeterol 100/12.5 DPI BID (125)<br>placebo (125)	exacerbation<br>FEV1<br>QoL<br>AEs
Saeed (2018)	—	Saeed, R.; Mustafa, K.; U. Saqib N. Comparison of montelukast with fluticasone for control of Asthma in children. Medical forum monthly 2018;29(3):25-28	unknown if patients used ICS at screening	parallel groups	780 (4-10)	780	montelukast 5-10 mg OD (390)<br>fluticasone 100 mcg BID (390)	FEV1
Shapiro (1998)	AstraZeneca	Shapiro GG, Bronsky EA, LaForce CF, Mendelson L, Pearlman D, Schwartz RH, Szefler SJ. Dose-related efficacy of budesonide administered via a dry powder inhaler in the treatment of children with moderate to severe persistent asthma. J Pediatr. 1998, 132 (6): 976-982	6-18 years<br>not only ICS on entry<br>triamcinolone is not on our list	parallel groups	404 (6-18)	not possible to establish	budesonide 100 mcg DPI BID (102)<br>budesonide 200 mcg DPI BID (100)<br>budesonide 400 mcg DPI BID (99)<br>placebo (103)	FEV1<br>symptoms<br>AEs
Shatalina (2017)	—	Shatalina, S.; Geppe, N.; Denisova, A.; Denisova, V.; Kolosova, N. Intermittent therapy with budesonide/formoterol in children with moderate asthma. European Respiratory Journal 2017;50(Supplement	congress abstract<br>6-18 years	parallel groups	95 (6-18)	not possible to establish	group 1: budesonide/formoterol in a fixed dose twice a day<br>group 2: budesonide/formoterol once a day and in exacerbation of asthma patient increased budesonide/formoterol to 4 inhalations/day for	FEV1<br>asthma symptoms

First author (Year)	Sponsor	Study Reference	Reasons for not extracting AgD	Study	Total randomized participants (age range)	Total randomized children／adolescents*	Treatments (number of participants reported)	Outcome(s) reported in the publication (does not imply adequate AgD)
		61): Netherlands European Respiratory Society 2017					7-14 days (intermittent therapy)<br>group 3: ICS (100-200µg budesonide/day)	
Sher (2017)	TEVA	Sher LD, Yiu G, Sakov A, Liu S, Caracta CF. Fluticasone propionate and fluticasone propionate/salmeterol multidose dry powder inhalers compared with placebo for persistent asthma. Allergy Asthma Proc. 2017 Sep 21;38(5):343-353. doi: 10.2500/aap.2017.38.4069.	population of both adults and children/ adolescents<br>ICS or ICS+LABA at entry	parallel groups	728 (12-65+)	45	fluticasone propionate 100 mcg MDPI BID (146)<br>fluticasone propionate 200 mcg MDPI BID (146)<br>fluticasone propionate/salmeterol 100/12.5 mcg MDPI BID (145)<br>fluticasone propionate/salmeterol 200/12.5 mcg MDPI BID (146)<br>placebo (145)	FEV1<br>QoL<br>AEs
Skoner (2008)	COVIS PHARMA	Skoner DP, Maspero J, Banerji D; Ciclesonide Pediatric Growth Study Group. Assessment of the long-term safety of inhaled ciclesonide on growth in children with asthma. Pediatrics. 2008 Jan;121(1):e1-14. doi: 10.1542/peds.2006-2206. Epub 2007 Dec 10. PMID: 18070931.	ICS or LTRA or SABA at screening	parallel groups	661 (5.5-9.1)	661	ciclesonide 40 mcg QD (221)<br>ciclesonide 160 mcg QD (219)<br>placebo (221)	FEV1<br>AEs (growth)
Steinfeld (2015)(§)	TEVA	Steinfeld, J.; Yiu, G.; Miller, S. D. Dose-ranging study to evaluate the efficacy and safety of four doses of fluticasone propionate/salmeterol multidose dry powder inhaler (FS MDPI) compared with fluticasone propionate (FP) MDPI and FS DPI in subjects with persistent asthma. Journal of allergy and clinical immunology. 2015;135(2 SUPPL. 1):AB6 2015	conference abstract<br>population of both adults and children/ adolescents<br>single dose	crossover	72 (NA)	not possible to establish	fluticasone/salmeterol MDPI 100/6.25 mcg<br>fluticasone/salmeterol MDPI 100/12.5 mcg<br>fluticasone/salmeterol MDPI 100/25 mcg<br>fluticasone/salmeterol MDPI 100/50 mcg<br>fluticasone propionate MDPI 100 mcg<br>fluticasone/salmeterol DPI 100/50 mcg	FEV1
Strunk (2008) (IPD)	CARE Network	Strunk RC, Bacharier LB, Phillips BR, Szefler SJ, Zeiger RS, Chinchilli VM, Martinez FD, Lemanske RF Jr, Taussig LM, Mauger DT, Morgan WJ, Sorkness CA, Paul IM, Guilbert T, Krawiec M, Covar R, Larsen G; CARE Network. Azithromycin or montelukast as inhaled corticosteroid-sparing agents in moderate-to-severe childhood asthma study. J Allergy Clin Immunol. 2008 Dec;122(6):1138-1144.e4. doi: 10.1016/j.jaci.2008.09.028. Epub 2008 Oct 25. PMID: 18951618; PMCID: PMC2737448.	not enough eligible patients<br>ICS alone (uncontrolled) or ICS+LABA or other (controlled)	parallel groups	55 (6-17)	1	placebo and budesonide (400 mcg as minimum)+ salmeterol (50 mcg) BID (19)<br>montelukast (5 or 10 mg) OD and budesonide (400 mcg as minimum)+ salmeterol (50 mcg) BID (19)	asthma control<br>AEs
Suessmuth (2003)	—	Suessmuth S, Freihorst J, Gappa M. Low-dose theophylline in childhood asthma: a placebo-controlled, double-blind study.	adolescents aged 18	parallel groups	36 (6-18)	36	ICS+theophylline 10 mg/kg bodyweight<br>ICS+placebo	symptoms<br>lung function

First author (Year)	Sponsor	Study Reference	Reasons for not extracting AgD	Study	Total randomized participants (age range)	Total randomized children ⁄ adolescents*	Treatments (number of participants reported)	Outcome(s) reported in the publication (does not imply adequate AgD)
		Pediatr Allergy Immunol. 2003 Oct;14(5):394-400.						
van Adelsberg (2005)	MERCK	van Adelsberg J, Moy J, Wei LX, Tozzi CA, Knorr B, Reiss TF. Safety, tolerability, and exploratory efficacy of montelukast in 6- to 24-month-old patients with asthma. Curr Med Res Opin. 2005 Jun;21(6):971-9.	50% used ICS; other medicine or no medicine used at screening and concomitant use of those during the study	parallel groups	256 (6-24 months)	128	ICS (87/175)+montelukast 4 mg (175)<br>ICS (41/81)+placebo (81)	exacerbation (asthma attack)<br>hospitalization<br>AEs
Vandewalker (2017)	TEVA	Vandewalker, Mark; Hickey, Lisa; Small, Calvin J. Efficacy and safety of beclomethasone dipropionate breath-actuated or metered-dose inhaler in pediatric patients with asthma. Allergy and asthma proceedings 2017;38(5):354-364	ICS or NCS at entry	parallel groups	628 (4-11)	445	beclomethasone dipropionate BAI 80 mcg die (126)<br>beclomethasone dipropionate BAI 160 mcg die (125)<br>beclomethasone dipropionate MDI 80 mcg die (125)<br>beclomethasone dipropionate MDI 160 mcg die (125)<br>placebo (127)	FEV1<br>exacerbation<br>symptoms<br>asthma control<br>AEs
Venugopal (2019)(§)	—	Venugopal, S. Effect of Addition of Single Dose of Oral Montelukast to Standard Therapy in Acute Moderate Asthma in Children 5-12 Years of Age - a Randomised Double Blind Placebo Controlled Trial. American journal of respiratory and critical care medicine 2019;199(): 2019	abstract - no information on previous treatments single dose of montelukast to standard therapy in exacerbation	parallel groups	43 (5-12)	43	standard therapy+single tablet of montelukast (5mg) (29)<br>standard therapy+single tablet of placebo (14)	none of interest
Verini (2007)	—	Verini M, Peroni D, Piacentini G, Nicodemo A, Rossi N, Bodini A, Chiarelli F, Boner A: Comparison of add-on therapy to inhaled fluticasone propionate in children with asthma: residual volume and exhaled nitric oxide as outcome measures. Allergy and asthma proceedings. 2007, 28 (6): 691-694	no data for the first period	crossover	12 (6-13)	12	fluticasone propionate 100 mcg BID + montelukast 5 mg OD (12)<br>fluticasone propionate 100 mcg BID + salmeterol 50 mcg BID (12)	exacerbation (none)<br>AEs (none)
von Berg (1998)	GSK	von Berg A, de Blic J, la Rosa M, Kaad PH, Moorat A. A comparison of regular salmeterol vs 'as required' salbutamol therapy in asthmatic children. Respir Med. 1998 Feb;92(2):292-9.	only 50% of patients used ICS at entry patients were allowed to use ICS, cromoglycate,	parallel groups	426 (5-15)	223	ICS (122/220) + salmeterol 50 mcg BID Diskhaler (220)<br>ICS (101/206) + placebo (206)	exacerbation<br>FEV1<br>symptoms<br>AEs

First author (Year)	Sponsor	Study Reference	Reasons for not extracting AgD	Study	Total randomized participants (age range)	Total randomized children ⁄ adolescents*	Treatments (number of participants reported)	Outcome(s) reported in the publication (does not imply adequate AgD)
			nedocromyl, or ketotifen during the study					
Weinstein (1998)	GSK	Weinstein SF, Pearlman DS, Bronsky EA, Byrne A, Arledge T, Liddle R, Stahl E. Efficacy of salmeterol xinafoate powder in children with chronic persistent asthma. Ann Allergy Asthma Immunol. 1998 Jul;81(1):51-8.	other medicine used at screening patients were allowed to use ICS, cromolyn, nedocromil or immunotherapy during the study	parallel groups	207 (4-11)	118	ICS (no patient number)+salmeterol 50 mcg BID (102)<br>ICS (no patient number)+placebo (105)	FEV1<br>AEs
Weinstein (2010)	MERCK	Weinstein SF, Corren J, Murphy K, Nolte H, White M; Study Investigators of P04431. Twelve-week efficacy and safety study of mometasone furoate/formoterol 200/10 microg and 400/10 microg combination treatments in patients with persistent asthma previously receiving high-dose inhaled corticosteroids. Allergy Asthma Proc. 2010 Jul-Aug;31(4):280-9. doi: 10.2500/aap.2010.31.3381. Epub 2010 Aug 3.	population of both adults and children/ adolescents<br>ICS or ICS+LABA at entry	parallel groups	728 (NA)	not possible to establish	mometasone furoate/formoterol 200/10 mcg BID (233)<br>mometasone furoate/formoterol 400/10 mcg BID (255)<br>mometasone furoate 400 mcg BID (240)	FEV1<br>exacerbation<br>ACQ<br>QoL<br>AEs
Weiss (2010)	MERCK	Weiss KB, Gern JE, Johnston NW, Sears MR, Jones CA, Jia G, Watkins MW, Smugar SS, Edelman JM, Grant EN. The Back to School asthma study: the effect of montelukast on asthma burden when initiated prophylactically at the start of the school year. Ann Allergy Asthma Immunol. 2010 Aug;105(2):174-81. doi: 10.1016/j.anai.2010.04.018. Epub 2010 Jul 1.	only 50% of patients used ICS	parallel groups	1162 (6-14)	597	ICS (314) + montelukast 5 mg (580)<br>ICS (283) + placebo (582)	worsening asthma<br>AEs
Zangrilli (2001)	AstraZeneca	Zangrilli J, Mansfield LE, Uryniak T, O'Brien CD. Efficacy of budesonide/formoterol pressurized metered-dose inhaler versus budesonide pressurized metered-dose inhaler alone in Hispanic adults and adolescents with asthma: a randomized, controlled trial. Ann Allergy Asthma Immunol. 2011 Sep;107(3):258-65.e2. doi: 10.1016/j.anai.2011.05.024. Epub 2011 Jul 14. PMID: 21875546.	population of both adults and children/ adolescents	parallel groups	250 (NA)	not possible to establish	budesonide/formoterol pMDI 160/4.5 µg × 2 inhalations (320/9 µg) twice daily (127)<br>budesonide pMDI 160 µg × 2 inhalations (320 µg) twice daily (123)	exacerbation<br>FEV1<br>symptoms<br>AEs

* Not all reported participants can be eligible for inclusion because it is not possible to establish if all inclusion criteria are met (e.g., pre-study treatment with ICS alone). (§): study that may be not eligible after further assessment

Table S6. Risk of bias for included studies with individual participant data or aggregate data (parts 1 to 5)

Study	Data	Treatment classes	Random sequence generation	Allocation concealment	Blinding of participants and personnel	Blinding of outcome assessment	Incomplete outcome data	Selective reporting	Other bias
Akpinarli 1999	AgD	ICS+LABA<br>ICS High	Unclear	Unclear	Unclear	Low	Unclear	Low	Low
Bateman 2014	IPD	ICS Low<br>ICS+LABA	Low	Low	Low	Low	Low	Low	Low
Berger 2006	AgD	ICS Low<br>placebo	Low	Unclear	Unclear	High[a]	Unclear	Low	Low
Bernstein 2015	IPD	ICS Low<br>ICS+LABA	Low	Low	Low	Low	Low	Low	Low
Bisgaard 2006	AgD	ICS Medium<br>ICS+LABA	Low	Low	Low	Low	Low	Low	Low
Bleecker 2012	IPD	ICS High<br>ICS Low<br>ICS Medium<br>Placebo	Low	Low	Low	Low	Low	Low	Low
Bleecker 2014	IPD	ICS Low<br>ICS+LABA<br>Placebo	Low	Low	Low	Low	Low	Low	Low
Buchvald 2003[1]	AgD	ICS Medium<br>ICS+LABA<br>ICS+LTRA	Low	Unclear	Unclear	Low	Low	Low	Unclear
Carroll 2010	IPD	ICS Low<br>ICS+LABA	Unclear	Unclear	Low	Low	Low	Low	Low
de Blic 2009	IPD	ICS Medium<br>ICS+LABA	Low	Low	Low	Low	Low	Low	Low

Study	Data	Treatment classes	Random sequence generation	Allocation concealment	Blinding of participants and personnel	Blinding of outcome assessment	Incomplete outcome data	Selective reporting	Other bias
Everden 2004	AgD	ICS+LABA (SAL)<br>ICS+LABA (FORM)	Low	High[b]	High[b]	High[b]	Low	Low	Unclear
Fitzpatrick 2016	IPD	ICS Low<br>LTRA	Low	Low	Low	Low	High	Low	High[c]
Gappa 2009	IPD	ICS Medium<br>ICS+LABA	Low	Low	Low	Low	Low	Low	Low
Heuck 2000	AgD	ICS+LABA<br>ICS Medium	Low	Low	Unclear	Low	High[d]	Low	Low
Jat 2006	AgD	ICS+LTRA<br>ICS Medium	Unclear	Unclear	Unclear	Low	High[e]	Low	Low
Kondo 2006	AgD	ICS+LTRA<br>ICS+theophylline	Low	Unclear	High	Low	Low	Unclear	Low
Lemanske 2010	IPD	ICS Medium<br>ICS+LABA<br>ICS+LTRA	Low	Low	Low	Low	Low	Low	High[f]
Lenney 2013	AgD	ICS Low<br>ICS+LABA<br>ICS+LTRA	Low	Low	Low	Low	High	Low	Low
Li 2010	IPD	ICS Low<br>ICS+LABA	Low	Low	Low	Low	Low	Low	Low
Lötvall 2014 a[2]	IPD	ICS Low<br>ICS Medium<br>ICS+LABA	Low	Low	Low	Low	Low	Low	Low
Lötvall 2014 b	IPD	ICS Low<br>ICS Medium<br>Placebo	Low	Low	Low		Low	Low	Low
Malone 2005	AgD	ICS Low<br>ICS+LABA	Low	Low	Low	Low	Low	Low	Low

Study	Data	Treatment classes	Random sequence generation	Allocation concealment	Blinding of participants and personnel	Blinding of outcome assessment	Incomplete outcome data	Selective reporting	Other bias
Martin 2020	IPD	ICS Medium<br>ICS+LABA	Low	Low	Low	Low	Low	Low	High[f]
Morice 2008	AgD	ICS Low<br>ICS+LABA	Low	Unclear	Unclear	Low	Low	Low	Low
Murray 2010	IPD	ICS Medium<br>ICS+LABA	Low	Low	Low	Low	Low	Low	Low
Murray 2011	IPD	ICS Low<br>ICS+LABA	Low	Low	Low	Low	Low	Low	Low
O'Byrne 2014	IPD	ICS High<br>ICS+LABA	Low	Low	Low	Low	Low	Low	Low
Oliver 2016 a	IPD	ICS Low<br>ICS+LABA	Low	Low	Low	Low	Low	Low	Low
Oliver 2016 b	IPD	ICS Low<br>Placebo	Low	Low	Low	Low	Low	Low	Low
Pearlman 2009	IPD	ICS Low<br>ICS+LABA	Low	Low	Low	Low	Low	Low	Low
Russell 1995	AgD	ICS+LABA<br>ICS High	Unclear	Unclear	Unclear	Unclear	Unclear	Low	Unclear
Scott 2005	IPD	ICS Low<br>ICS+LABA	Low	Low	Low	Low	Low	Unclear	High[g]
Shapiro 2001	AgD	ICS Low<br>ICS Medium<br>Placebo	Unclear	Unclear	Low	Low	Unclear	Low	Low
Simons 2001[1]	AgD	ICS Medium<br>ICS+LTRA	Unclear	Unclear	Low	Low	Low	Low	High[c]

Study	Data	Treatment classes	Random sequence generation	Allocation concealment	Blinding of participants and personnel	Blinding of outcome assessment	Incomplete outcome data	Selective reporting	Other bias
Sorkness 2007	IPD	ICS Low<br>ICS+LABA<br>LTRA	Low	Low	Low	Low	Low	Low	Low
Stempel 2016 a	IPD	ICS Medium<br>ICS+LABA	Low	Low	Unclear	Low	Low	Low	Unclear
Stempel 2016 b	IPD	ICS High<br>ICS Low<br>ICS Medium<br>ICS+LABA	Low	Low	Unclear	Low	Low	Low	Unclear
Strauch 2003	AgD	ICS High<br>ICS+LTRA	Unclear	Unclear	Low	Low	Low	Low	Low
Tal 2002	AgD	ICS Low<br>ICS+LABA	Low	Low	Low	Low	Low	Low	Low
Thomas 2014	IPD	ICS Medium<br>ICS+LABA<br>ICS+LTRA	High[h]	High[h]	High[h]	Low	Low	Low	Unclear
Vaessen-Verberne 2010	IPD	ICS Medium<br>ICS+LABA	Low	Low	Unclear	Low	Low	Low	High[g]
Verberne 1998	IPD	ICS High<br>ICS+LABA	Low	Low	Low	Low	High[i]	Low	High[i]
Vermeulen 2007	AgD	ICS Medium (CIC)<br>ICS Medium (BUD)	Low	Low	Unclear	Low	Low	Low	Low
Visitsunthorn 2011	AgD	ICS unknown dose<br>ICS+LTRA	Unclear	Unclear	Unclear	Low	Low	Low	High[f]

Study	Data	Treatment classes	Random sequence generation	Allocation concealment	Blinding of participants and personnel	Blinding of outcome assessment	Incomplete outcome data	Selective reporting	Other bias
Wechsler 2019	IPD	ICS High<br>ICS Low<br>ICS+LABA	Low	Low	Low	Low	Low	Low	High[f]
Woodcock 2013	IPD	ICS Low+LABA<br>ICS Medium+LABA	Low	Low	Low	Low	Low	Low	Low
Woodcock 2014	IPD	ICS High<br>ICS Low	Low	Low	Low	Low	Low	Low	Low
Zimmerman 2004	AgD	ICS Medium<br>ICS+LABA	Unclear	Unclear	Unclear	Low	Low	Low	Unclear

[1] data could not be included in analyses as insufficient data reported for first period of cross-over
[2] Lötvall 2014 a included in analyses as two separate studies
[a] response to therapy was assessed by the physician or a designee by comparing the current level of symptoms with those noted at the baseline visit using a 5-point scale. The method can be affected by subjectivity.
[b] study medication was sourced from commercially available stock and was repackaged and administered according to a computer-generated randomization scheme provided by the sponsor. No further details
[c] cross-over trial with no wash-out period
[d] only 24 of 27 children were included in the analysis (11% of missing outcome data). These three withdrawn children were all in the BUD-placebo group, and two had an exacerbation requiring oral corticosteroids.
[e] 8 (11.3%) of 71 randomized patients were dropped out in the first two weeks and were not included in the analysis. Patients dropped out were 4 for each group, and no reasons were provided.
[f] possible carry-over effect
[g] no peer reviewed publication
[h] no methods reported. No protocol was provided by the author
[i] possible bias as discrepancy identified between data and publication that could not be verified due to age of trial and lack of documentation

TABLE S7 Exacerbation Bayesian random-effects network meta-analysis (OR[a], 95% CrI) with IPD and AgD (Analysis A1: 40 trials, 8168 participants, 649 events)

TRT 1 \ TRT 2	ICS Low	ICS Medium	ICS High	ICS Low + LABA	ICS Medium + LABA	ICS High + LABA	ICS+LTRA	LTRA	ICS + Theophylline	Placebo
**ICS Low**	○	1.28 (0.67–2.44)	1.35 (0.54–3.39)	1.20 (0.73–1.95)	**2.29 (1.11–5.21)**	1.06 (0.41–2.77)	0.80 (0.23–2.75)	0.28 (0.04–1.68)	0.74 (0.01–41.26)	**0.42 (0.18–0.91)**
**ICS Medium**	0.78 (0.41–1.49)	○	1.05 (0.41–2.72)	0.93 (0.53–1.67)	1.79 (0.96–3.74)	0.83 (0.33–2.18)	0.63 (0.19–2.10)	0.21 (0.03–1.45)	0.58 (0.01–30.88)	**0.33 (0.13–0.82)**
**ICS High**	0.74 (0.30–1.84)	0.95 (0.37–2.44)	○	0.89 (0.35–2.18)	1.70 (0.68–4.62)	0.79 (0.36–1.72)	0.59 (0.14–2.53)	0.20 (0.02–1.52)	0.55 (0.01–32.46)	**0.31 (0.09–0.98)**
**ICS Low + LABA**	0.84 (0.51–1.38)	1.07 (0.60–1.90)	1.13 (0.46–2.83)	○	1.92 (0.95–4.31)	0.89 (0.35–2.27)	0.67 (0.20–2.27)	0.23 (0.03–1.51)	0.63 (0.01–35.16)	**0.35 (0.14–0.84)**
**ICS Medium + LABA**	**0.44 (0.19–0.90)**	0.56 (0.27–1.04)	0.59 (0.22–1.46)	0.52 (0.23–1.05)	○	0.46 (0.17–1.17)	0.35 (0.09–1.27)	**0.12 (0.01–0.84)**	0.32 (0.01–18.17)	**0.18 (0.06–0.49)**
**ICS High + LABA**	0.94 (0.36–2.41)	1.21 (0.46–3.03)	1.27 (0.58–2.80)	1.13 (0.44–2.83)	2.16 (0.85–5.87)	○	0.76 (0.18–3.25)	0.26 (0.03–1.99)	0.70 (0.01–40.85)	0.39 (0.12–1.26)
**ICS+LTRA**	1.25 (0.36–4.35)	1.60 (0.48–5.26)	1.68 (0.39–7.17)	1.49 (0.44–4.90)	2.86 (0.79–10.91)	1.32 (0.31–5.58)	○	0.34 (0.03–3.03)	0.93 (0.02–41.26)	0.53 (0.12–2.14)
**LTRA**	3.63 (0.59–24.78)	4.66 (0.69–36.97)	4.90 (0.66–42.95)	4.35 (0.66–32.14)	**8.33 (1.20–69.41)**	3.86 (0.50–34.12)	2.92 (0.33–28.79)	○	2.72 (0.03–230.44)	1.52 (0.21–12.18)
**ICS + Theophylline**	1.35 (0.02–74.44)	1.72 (0.03–95.58)	1.82 (0.03–109.95)	1.60 (0.03–86.49)	3.10 (0.06–181.27)	1.42 (0.02–84.77)	1.07 (0.02–47.94)	0.37 (0.00–29.67)	○	0.57 (0.01–31.82)
**Placebo**	**2.39 (1.09–5.42)**	**3.03 (1.22–7.77)**	**3.22 (1.02–10.70)**	**2.86 (1.19–7.10)**	**5.47 (2.03–17.12)**	2.53 (0.79–8.58)	1.90 (0.47–8.17)	0.66 (0.08–4.71)	1.77 (0.03–100.48)	○

The table compares the effect estimate for an intervention in the row with an intervention in a column (TRT 1 vs. TRT 2).
[a] OR > 1 favors treatment 2 (the probability of having exacerbation was modelled); 95% CrIs that exclude unity are highlighted in bold.
OR: odds ratio; CrI: credibility interval; IPD: individual participant data; AgD: aggregate data; TRT: treatment; ICS: inhaled corticosteroid; LABA: Long-Acting β$_2$-Agonist; LTRA: Leukotriene Receptor Antagonist

Table S8. Bayesian fixed effect network meta-analysis results (IPD and AgD) for exacerbations. ICS grouped with LABA – Analysis B1

TRT 1 \ TRT 2	ICS Low	ICS Medium	ICS High	ICS+LABA	ICS+LTRA	LTRA	ICS+ Theophylline	Placebo
**ICS Low**		1.11<br>(0.75; 1.63)<br>*1.19 (0.46; 3.03)*	1.42<br>(0.84; 2.46)<br>*2.48 (0.90; 7.10)*	1.27<br>(0.90; 1.79)<br>*1.25 (0.87; 1.79)*	0.75<br>(0.30; 1.90)<br>*1.49 (0.32; 8.85) ***	0.28<br>(0.05; 1.17)<br>*0..33 (0.07; 1.23) ***	0.74<br>(0.02; 27.66)	**0.43**<br>**(0.28; 0.66)**<br>***0.41 (0.26; 0.64)***
**ICS Medium**	0.90<br>(0.61; 1.34)<br>*0.84 (0.33; 2.18)*		1.30<br>(0.78; 2.14)<br>*0.52 (0.07; 3.60)*	1.15<br>(0.90; 1.48)<br>*1.19 (0.92; 1.52)*	0.68<br>(0.28; 1.65)<br>***0.22 (0.05;0.76) *****	0.25<br>(0.05; 1.12)	0.68<br>(0.02; 24.53)	**0.39**<br>**(0.22; 0.66)**<br>*0.72 (0.27; 1.90)*
**ICS High**	0.70<br>(0.41; 1.20)<br>*0.40 (0.14; 1.11)*	0.77<br>(0.47; 1.28)<br>*1.92 (0.28; 15.03)*		0.90<br>(0.57; 1.40)<br>*0.96 (0.61; 1.52)*	0.52<br>(0.19; 1.45)	**0.20**<br>**(0.04; 0.92)**	0.52<br>(0.01; 19.69)	**0.30**<br>**(0.15; 0.58)**<br>*Not estimable**
**ICS+LABA**	0.79<br>(0.56; 1.11)<br>*0.80 (0.56; 1.15)*	0.87<br>(0.68; 1.11)<br>*0.84 (0.66; 1.08)*	1.12<br>(0.71; 1.77)<br>*1.04 (0.66; 1.65)*		0.58<br>(0.24; 1.45)<br>*2.46 (0.59; 12.18) ***	**0.22**<br>**(0.04; 0.95)**	0.58<br>(0.02; 21.76)	**0.33**<br>**(0.20; 0.56)**<br>*Not estimable**
**ICS+LTRA**	1.64<br>(0.53; 3.35)<br>*0.67 (0.13; 3.22) ***	1.48<br>(0.61; 3.60)<br>***4.48 (1.30; 21.12) *****	1.92<br>(0.69; 5.16)	1.72<br>(0.69; 4.14)<br>*0.41 (0.07; 1.58) ***		0.37<br>(0.06; 2.08)	1.00<br>(0.03; 32.14)<br>*1.00 (0.08; 12.55) ***	0.57<br>(0.21; 1.54)
**LTRA**	3.60<br>(0.85; 18.36)<br>*3.32 (0.86; 13.30) ***	3.97<br>(0.90; 21.33)	**5.10**<br>**(1.08; 28.50)**	**4.57**<br>**(1.05; 24.29)**	2.69<br>(0.48; 16.78)		2.66<br>(0.05; 135.95)	1.54<br>(0.33; 8.33)
**ICS+ Theophylline**	1.35<br>(0.04; 49.40)	1.48<br>(0.04; 54.60)	1.92<br>(0.05; 72.97)	1.72<br>(0.05; 64.07)	1.00<br>(0.03; 33.45)<br>*1.11 (0.10; 13.60) ***	0.38<br>(0.01; 18.73)		0.57<br>(0.02; 21.76)
**Placebo**	**2.34**<br>**(1.52; 3.63)**<br>***2.46 (1.55; 3.86)***	**2.59**<br>**(1.51; 4.48)**<br>*1.39 (0.53; 3.74)*	**3.35**<br>**(1.72; 6.55)**<br>*Not estimable**	**3.00**<br>**(1.79; 5.05)**<br>*Not estimable**	1.75<br>(0.65; 4.81)	0.65<br>(0.12; 3.00)	1.75<br>(0.05; 66.02)	

The table compares the effect estimate for an intervention in the row with an intervention in a column (TRT 1 vs. TRT 2).
39 studies, 8136 patients, 649 events ─ Reference treatment is: ICS+LABA, DIC: 2296.3, residual deviance: 2254.1 (on 5377 data points).
OR > 1 favours treatment 2 (the probability of having exacerbations was modelled). Results with CrI that exclude the OR value of 1 are highlighted in bold. Direct results from pairwise meta-analyses, where applicable, are in Italic. * Not estimable: zero events in both arms; ** Estimates from Bayesian logistic regression models (Stan) (one study).

Table S9. Sensitivity analysis excluding exacerbation events identified from adverse event data: Bayesian random-effects network meta-analysis results (IPD and AgD) for exacerbations. ICS stratified by dose when combined with LABA – Analysis A1

TRT 1 \ TRT 2	ICS Low	ICS Medium	ICS High	ICS Low + LABA	ICS Medium + LABA	ICS High + LABA	ICS+LTRA	LTRA	ICS + Theophylline	Placebo
**ICS Low**		2.34 (0.96 to 6.36)	1.93 (0.64 to 5.93)	1.34 (0.70 to 2.53)	**4.10 (1.36 to 15.03)**	1.26 (0.4 1to 4.18)	1.11 (0.28 to 4.76)	NA	NA	**0.25 (0.07 to 0.77)**
**ICS Medium**	0.43 (0.16 to 1.04)		0.83 (0.25 to 2.59)	0.58 (0.23 to 1.21)	1.75 (0.69 to 5.05)	0.54 (0.16 to 1.75)	0.47 (0.12 to 1.88)	NA	NA	**0.11 (0.02 to 0.43)**
**ICS High**	0.52 (0.17 to 1.55)	1.21 (0.39 to 4.01)		0.70 (0.23 to 1.97)	2.12 (0.68 to 7.92)	0.66 (0.23 to 1.93)	0.58 (0.11 to 3.03)	NA	NA	**0.13 (0.02 to 0.59)**
**ICS Low + LABA**	0.75 (0.39 to 1.42)	1.73 (0.8 3to 4.26)	1.43 (0.51 to 4.44)		**3.06 (1.11 to 10.80)**	0.94 (0.3 2to 3.03)	0.83 (0.22 to 3.35)	NA	NA	**0.19 (0.05 to 0.68)**
**ICS Medium + LABA**	**0.24 (0.07 to 0.73)**	0.57 (0.20 to 1.45)	0.47 (0.13 to 1.48)	**0.33 (0.09 to 0.90)**		**0.31 (0.08 to 0.98)**	0.27 (0.05 to 1.30)	NA	NA	**0.06 (0.01 to 0.29)**
**ICS High + LABA**	0.79 (0.24 to 2.44)	1.84 (0.57 to 6.17)	1.52 (0.52 to 4.35)	1.06 (0.33 to 3.10)	**3.22 (1.02 to 12.06)**		0.88 (0.17 to 4.81)	NA	NA	**0.20 (0.04 to 0.95)**
**ICS+LTRA**	0.90 (0.21 to 3.56)	2.12 (0.53 to 8.58)	1.73 (0.33 to 9.03)	1.21 (0.30 to 4.53)	3.71 (0.77 to 20.29)	1.14 (0.21 to 6.05)		NA	NA	0.23 (0.03 to 1.34)
**LTRA**	NA	NA	NA	NA	NA	NA	NA		NA	NA
**ICS + Theophylline**	NA	NA	NA	NA	NA	NA	NA	NA		NA
**Placebo**	**3.94 (1.30 to 13.60)**	**9.12 (2.34 to 45.15)**	**7.54 (1.68 to 40.45)**	**5.26 (1.48 to 20.91)**	**15.96 (3.46 to 98.49)**	**4.95 (1.05 to 28.50)**	4.35 (0.75 to 29.08)	NA	NA	

The table compares the effect estimate for an intervention in the row with an intervention in a column (TRT 1 vs. TRT 2).
OR (95% CrI) (29 studies, 6005 participants, 519 events). Reference treatment: ICS Low ─ DIC: 2152.5; Residual deviance: 2113 (on 5020 data points). OR > 1 favours treatment 2 (the probability of having exacerbation was modelled). Results with CrI that exclude the OR value of 1 are highlighted in bold. All available data included (IPD and AgD wherever available); TRT 1 = treatment 1; TRT 2 = treatment 2; OR = odds ratio; CrI = credibility interval; DIC = deviance information criterion; ICS = inhaled corticosteroid; LABA = long-acting beta-agonist; LTRA = leukotriene receptor antagonist; NA = not available

Table S10. Sensitivity analysis excluding exacerbation events identified from adverse event data: Bayesian fixed effect network meta-analysis results (IPD and AgD) for the exacerbation outcome. ICS grouped when combined with LABA – Analysis B1

TRT 1 \ TRT 2	ICS Low	ICS Medium	ICS High	ICS+LABA	ICS+LTRA	LTRA	ICS+ Theophylline	Placebo
**ICS Low**		1.36 (0.83 to 2.23)	1.73 (0.90 to 3.32)	1.39 (0.90 to 2.16)	0.83 (0.32 to 2.18)	NA	NA	**0.32 (0.19 to 0.53)**
**ICS Medium**	0.73 (0.45 to 1.21)		1.27 (0.70 to 2.32)	1.02 (0.79 to 1.32)	0.61 (0.24 to 1.51)	NA	NA	**0.24 (0.12 to 0.48)**
**ICS High**	0.58 (0.30 to 1.11)	0.79 (0.43 to 1.42)		0.80 (0.46 to 1.38)	0.48 (0.17 to 1.35)	NA	NA	**0.19 (0.08 to 0.42)**
**ICS+LABA**	0.72 (0.46 to 1.11)	0.98 (0.76 to 1.27)	1.25 (0.73 to 2.16)		0.59 (0.24 to 1.48)	NA	NA	**0.23 (0.12 to 0.44)**
**ICS+LTRA**	1.21 (0.46 to 3.13)	1.63 (0.66 to 4.14)	2.10 (0.74 to 6.05)	1.68 (0.68to 4.18)		NA	NA	0.39 (0.13 to 1.15)
**LTRA**	NA	NA	NA	NA	NA		NA	NA
**ICS+Theophylline**	NA	NA	NA	NA	NA	NA		NA
**Placebo**	**3.10 (1.88 to 5.16)**	**4.18 (2.10 to 8.50)**	**5.37 (2.36 to 12.18)**	**4.31 (2.25 to 8.33)**	2.56 (0.87 to 7.61)	NA	NA	

The table compares the effect estimate for an intervention in the row with an intervention in a column (TRT 1 vs. TRT 2).
28 studies, 5973 patients, 519 events ─ Reference treatment is: ICS+LABA, DIC: 2160.7; Residual deviance: 2132.2 (on 4988 data points). OR > 1 favors treatment 2 (the probability of having exacerbation was modelled).
Results with CrI that exclude the OR value of 1 are highlighted in bold.
All available data included (IPD and AgD wherever available); TRT 1 = treatment 1; TRT 2 = treatment 2; OR = odds ratio; CrI = credibility interval; DIC = deviance information criterion.
ICS = inhaled corticosteroid; LABA = long-acting beta-agonist; LTRA = leukotriene receptor antagonist; NA = not available

Table S11. Sensitivity analysis to explore data availability bias: Bayesian fixed effect network meta-analysis results for exacerbations. ICS stratified by dose when combined with LABA (IPD trials only, i.e., excluding trials with AgD only) – Analysis A1

TRT 1 \ TRT2	ICS Low	ICS Medium	ICS High	ICS Low + LABA	ICS Medium + LABA	ICS High + LABA	ICS+ LTRA	LTRA	ICS + Theophylline	Placebo
**ICS Low**		1.82 (0.87 to 3.78)	1.67 (0.76 to 3.63)	1.32 (0.79 to 2.20)	**2.32 (1.08 to 4.90)**	1.04 (0.47 to 2.29)	NA	0.28 (0.06 to 1.21)	NA	**0.12 (0.02 to 0.59)**
**ICS Medium**	0.55 (0.26 to 1.15)		0.91 (0.44 to 1.93)	0.73 (0.39 to 1.35)	1.27 (0.90 to 1.77)	0.57 (0.27 to 1.22)	NA	**0.15 (0.03 to 0.79)**	NA	**0.07 (0.01 to 0.38)**
**ICS High**	0.60 (0.28 to 1.31)	1.09 (0.52 to 2.29)		0.79 (0.38 to 1.65)	1.39 (0.67 to 2.92)	0.63 (0.34 to 1.16)	NA	**0.17 (0.03 to 0.88)**	NA	**0.07 (0.01 to 0.42)**
**ICS Low + LABA**	0.76 (0.45 to 1.26)	1.38 (0.74 to 2.53)	1.26 (0.61 to 2.61)		1.75 (0.91 to 3.32)	0.79 (0.37 to 1.65)	NA	**0.21 (0.04 to 0.98)**	NA	**0.09 (0.01 to 0.49)**
**ICS Medium + LABA**	**0.43 (0.20 to 0.92)**	0.79 (0.57 to 1.11)	0.72 (0.34 to 1.49)	0.57 (0.30 to 1.09)		**0.45 (0.21 to 0.96)**	NA	**0.12 (0.02 to 0.64)**	NA	**0.05 (0.01 to 0.30)**
**ICS High + LABA**	0.96 (0.44 to 2.12)	1.75 (0.82 to 3.74)	1.60 (0.86 to 2.97)	1.27 (0.61 to 2.69)	**2.23 (1.04 to 4.71)**		NA	0.27 (0.04 to 1.42)	NA	**0.11 (0.02 to 0.68)**
**ICS+LTRA**	NA	NA	NA	NA	NA	NA		NA	NA	NA
**LTRA**	3.60 (0.83 to 18.17)	**6.55 (1.26 to 39.25)**	**5.99 (1.14 to 36.23)**	**4.81 (1.02 to 26.05)**	**8.33 (1.55 to 50.40)**	3.74 (0.70 to 22.65)	NA		NA	0.43 (0.04 to 4.22)
**ICS + Theophylline**	NA	NA	NA	NA	NA	NA	NA	NA		NA
**Placebo**	**8.41 (1.70 to 52.98)**	**15.33 (2.66 to 109.95)**	**14.01 (2.39 to 100.48)**	**11.13 (2.05 to 75.94)**	**19.49 (3.35 to 141.17)**	**8.76 (1.48 to 62.18)**	NA	2.34 (0.24 to 23.57)	NA	

The table compares the effect estimate for an intervention in the row with an intervention in a column (TRT 1 vs. TRT 2).
OR (95% CrI) (27 studies, 5381 patients, 328 events); Reference treatment: ICS Low ─ DIC: 2242.3; Residual deviance: 2212.7 (on 5381 data points). OR > 1 favours treatment 2 (the probability of having exacerbation was modelled). Results with CrI that exclude the OR value of 1 are highlighted in bold. TRT 1 = treatment 1; TRT 2 = treatment 2; OR = odds ratio; CrI = credibility interval; DIC = deviance information criterion; ICS = inhaled corticosteroid; LABA = long-acting beta-agonist; LTRA = leukotriene receptor antagonist; NA = not available

Table S12. Sensitivity analysis to explore data availability bias: Bayesian fixed effect network meta-analysis results for the exacerbation outcome (including ICS grouped when combined with LABA). IPD trials only (i.e., excluding trials with AgD only) – Analysis B1

TRT 1 \ TRT 2	ICS Low	ICS Medium	ICS High	ICS+LABA	ICS+LTRA	LTRA	ICS+ Theophylline	Placebo
**ICS Low**		1.09<br>(0.61 to 1.93)	1.54<br>(0.79 to 3.03)	1.23<br>(0.75 to 1.99)	NA	0.28<br>(0.05 to 1.17)	NA	**0.12<br>(0.02 to 0.59)**
**ICS Medium**	0.91<br>(0.52 to 1.63)		1.40<br>(0.76 to 2.59)	1.13<br>(0.84 to 1.52)	NA	0.25<br>(0.05 to 1.21)	NA	**0.11<br>(0.02 to 0.57)**
**ICS High**	0.65<br>(0.33 to 1.27)	0.71<br>(0.39 to 1.31)		0.80<br>(0.47 to 1.36)	NA	**0.18<br>(0.03 to 0.90)**	NA	**0.08<br>(0.01 to 0.44)**
**ICS+LABA**	0.81<br>(0.50 to 1.34)	0.89<br>(0.66 to 1.20)	1.25<br>(0.73 to 2.14)		NA	0.23<br>(0.04 to 1.03)	NA	**0.09<br>(0.01 to 0.50)**
**ICS+LTRA**	NA	NA	NA	NA		NA	NA	NA
**LTRA**	3.60<br>(0.85 to 18.36)	3.97<br>(0.83 to 22.20)	**5.53<br>(1.11 to 31.50)**	4.44<br>(0.97 to 24.05)	NA		NA	0.42<br>(0.04 to 4.18)
**ICS+Theophylline**	NA	NA	NA	NA	NA	NA		NA
**Placebo**	**8.58<br>(1.68 to 52.46)**	**9.39<br>(1.75 to 60.95)**	**13.20<br>(2.29 to 88.23)**	**10.59<br>(1.99 to 67.36)**	NA	2.36<br>(0.24 to 23.57)	NA	

The table compares the effect estimate for an intervention in the row with an intervention in a column (TRT 1 vs. TRT 2).
OR (95% CrI) (26 studies, 5349 participants, 328 events). Reference treatment: ICS Low ─ DIC: 2243.4; Residual deviance: 2215.5 (on 5349 data points)
OR > 1 favours treatment 2 (the probability of having exacerbation was modelled). Results with CrI that exclude the OR value of 1 are highlighted in bold.
All available data included (IPD and AgD wherever available) ─ IPD = Individual Participant Data; AgD = Aggregate Data; TRT 1 = treatment 1; TRT 2 = treatment 2; OR = odds ratio; CrI = credibility interval; DIC = deviance information criterion; ICS = inhaled corticosteroid; LABA = long-acting beta-agonist; LTRA = leukotriene receptor antagonist; NA = not available

TABLE S13 Asthma Control Bayesian fixed effect network meta-analysis (OR[a], 95% CrI) with IPD (Analysis A2: 16 trials, 3027 participants, 2453 events)

TRT 1 \ TRT 2	ICS Low	ICS Medium	ICS High	ICS Low + LABA	ICS Medium + LABA	ICS High + LABA	ICS+LTRA	LTRA	Placebo
ICS Low	○	0.94 (0.50–1.73)	1.32 (0.70–2.46)	0.86 (0.62–1.21)	0.90 (0.49–1.67)	0.68 (0.34–1.31)	0.82 (0.13–4.71)	4.31 (0.90–21.54)	1.42 (0.78–2.56)
ICS Medium	1.06 (0.58–1.99)	○	1.42 (0.73–2.72)	0.92 (0.50–1.68)	0.96 (0.73–1.27)	0.72 (0.35–1.43)	0.87 (0.14–4.95)	4.57 (0.87–25.28)	1.52 (0.66–3.42)
ICS High	0.76 (0.41–1.43)	0.70 (0.37–1.36)	○	0.65 (0.35–1.22)	0.68 (0.35–1.30)	0.51 (0.25–1.03)	0.62 (0.09–3.74)	3.25 (0.61–18.17)	1.07 (0.46–2.48)
ICS Low + LABA	1.16 (0.83–1.62)	1.08 (0.59–1.99)	1.54 (0.82–2.86)	○	1.04 (0.57–1.92)	0.78 (0.39–1.51)	0.95 (0.15–5.31)	5.00 (1.04–25.53)	1.65 (0.86–3.16)
ICS Medium + LABA	1.12 (0.60–2.05)	1.04 (0.79–1.38)	1.48 (0.77–2.83)	0.96 (0.52–1.75)	○	0.75 (0.36–1.49)	0.90 (0.14–5.21)	4.76 (0.91–26.05)	1.58 (0.69–3.60)
ICS High + LABA	1.48 (0.76–2.94)	1.39 (0.70–2.86)	1.97 (0.97–4.01)	1.28 (0.66–2.53)	1.34 (0.67–2.75)	○	1.21 (0.18–7.46)	6.36 (1.17–35.87)	2.12 (0.87–5.16)
ICS+LTRA	1.22 (0.21–7.61)	1.15 (0.20–7.10)	1.62 (0.27–10.59)	1.05 (0.19–6.69)	1.11 (0.19–6.96)	0.83 (0.13–5.53)	○	5.26 (0.52–60.34)	1.75 (0.28–11.82)
LTRA	0.23 (0.05–1.11)	0.22 (0.04–1.15)	0.31 (0.06–1.63)	0.20 (0.04–0.96)	0.21 (0.04–1.09)	0.16 (0.03–0.85)	0.19 (0.02–1.93)	○	0.33 (0.06–1.75)
Placebo	0.70 (0.39–1.28)	0.66 (0.29–1.51)	0.93 (0.40–2.16)	0.61 (0.32–1.16)	0.63 (0.28–1.45)	0.47 (0.19–1.15)	0.57 (0.08–3.53)	3.00 (0.57–16.61)	○

The table compares the effect estimate for an intervention in the row with an intervention in a column (TRT 1 vs. TRT 2).
[a] OR > 1 favours treatment 1 (the probability of having good/total asthma control was modelled); 95% CrIs that exclude unity are highlighted in bold.
OR: odds ratio; CrI: credibility interval; IPD: individual participant data; TRT: treatment; ICS: inhaled corticosteroid; LABA: Long-Acting β_2-Agonist; LTRA: Leukotriene Receptor Antagonist

Table S14. Bayesian fixed effect network meta-analysis (IPD only) for asthma control. ICS grouped when combined with LABA – Analysis B2

TRT 1 \ TRT 2	ICS Low	ICS Medium	ICS High	ICS+LABA	ICS+LTRA	LTRA	ICS + Theophylline	Placebo
**ICS Low**		0.90<br>(0.59 to 1.36)<br>*0.54 (0.18 to 1.54)*	1.36<br>(0.76 to 2.44)<br>*0.80 (0.37 to 1.73)*	0.85<br>(0.62 to 1.17)<br>*0.90 (0.64 to 1.26)*	0.81<br>(0.14 to 4.76)	4.35<br>(0.93 to 21.98)<br>*3.32 (0.73 to 18.17) ***	NA	1.42<br>(0.77 to 2.56)<br>*1.16 (0.59 to 2.20)*
**ICS Medium**	1.12<br>(0.73 to 1.68)<br>*1.86 (0.65 to 5.42)*		1.51<br>(0.84 to 2.69)<br>*2.23 (0.88 to 5.53) ***	0.94<br>(0.72 to 1.25)<br>*0.91 (0.69 to 1.22)*	0.90<br>(0.15 to 5.10)<br>*Not estimable (*)*	4.85<br>(1.00 to 25.28)	NA	1.58<br>(0.79 to 3.13)<br>*0.67 (0.12 to 4.01) ***
**ICS High**	0.73<br>(0.41 to 1.31)<br>*1.25 (0.58 to 2.72)*	0.66<br>(0.37 to 1.19)<br>*0.45 (0.18 to 1.16) ***		0.63<br>(0.37 to 1.07)<br>***0.53 (0.30 to 0.96)***	0.59<br>(0.09 to 3.63)	3.19<br>(0.62 to 17.99)	NA	1.04<br>(0.46 to 2.36)
**ICS+LABA**	1.17<br>(0.85 to 1.62)<br>*1.12 (0.79 to 1.55)*	1.06<br>(0.80 to 1.39)<br>*1.09 (0.82 to 1.45)*	1.60<br>(0.93 to 2.72)<br>***1.88 (1.04 to 3.39)***		0.95<br>(0.16 to 5.37)<br>*0.43 (0.06 to 2.56)*	**5.16**<br>**(1.08 to 26.58)**<br>*4.48 (0.70 to 53.52) ***	NA	1.67<br>(0.88 to 3.22)<br>***9.97 (2.01 to 59.15) *****
**ICS+LTRA**	1.23<br>(0.21 to 7.39)	1.12<br>(0.20 to 6.62)<br>*Not estimable*	1.68<br>(0.28 to 10.80)	1.05<br>(0.19 to 6.23)<br>*2.34 (0.39 to 15.49)*		5.42<br>(0.52 to 60.95)	NA	1.75<br>(0.28 to 11.36)
**LTRA**	0.23<br>(0.05 to 1.07)<br>*0.27 (0.06 to 1.27) ***	0.21<br>(0.04 to 1.00)	0.31<br>(0.10 to 1.62)	**0.19**<br>**(0.04 to 0.92)**<br>*0.22 (0.02 to 1.54) ***	0.18<br>(0.02 to 1.93)		NA	0.33<br>(0.06 to 1.68)
**ICS + Theophylline**	NA	NA	NA	NA	NA	NA		NA
**Placebo**	0.70<br>(0.39 to 1.30)<br>*0.86 (0.45 to 1.68)*	0.63<br>(0.32 to 1.26)<br>*1.35 (0.23 to 8.08) ***	0.96<br>(0.42 to 2.18)	0.60<br>(0.31 to 1.14)<br>***0.11 (0.02 to 0.50) *****	0.57<br>(0.09 to 3.60)	3.06<br>(0.59 to 17.46)	NA	

The table compares the effect estimate for an intervention in the row with an intervention in a column (TRT 1 vs. TRT 2).
15 studies, 2998 patients, 2433 events. Reference treatment: ICS+LABA ─ DIC: 2822.5; Residual deviance: 2801.3 (on 2998 data points))
OR > 1 favors treatment 1 (the probability of having good/total asthma control was modelled). Direct results from pairwise meta-analyses, where applicable, are in Italic. Results with CrI that exclude the OR value of 1 are highlighted in bold. ICS = inhaled corticosteroid; LABA = long-acting beta-agonist; LTRA = leukotriene receptor antagonist; OR = odds ratio; CrI = credibility interval; DIC = deviance information criterion; NA: not available; ** Estimates from Bayesian logistic regression models (Stan) (one study).

Table S15. Bayesian random-effects network meta-analysis (IPD only) for asthma control (individual compounds) – Analysis C2

TRT 1 \ TRT 2	FF	FF + VI	FP	FP + Montelukast	FP + SAL	FP + VI	Montelukast	Placebo
**FF**		0.51 (0.16 to 1.26)	1.63 (0.53 to 5.00)	1.58 (0.13 to 18.36)	1.73 (0.50 to 7.32)	1.68 (0.22 to 12.81)	8.17 (0.78 to 94.63)	1.54 (0.50 to 4.57)
**FF + VI**	1.97 (0.79 to 6.42)		3.25 (0.97 to 12.55)	3.13 (0.26 to 43.82)	3.46 (0.93 to 18.54)	3.32 (0.45 to 31.82)	**16.28 (1.52 to 212.72)**	3.03 (0.88 to 13.20)
**FP**	0.61 (0.20 to 1.90)	0.31 (0.08 to 1.03)		0.96 (0.10 to 9.03)	1.06 (0.50 to 2.91)	1.02 (0.19 to 5.58)	5.00 (0.61 to 44.70)	0.93 (0.25 to 3.35)
**FP + Montelukast**	0.63 (0.05 to 7.46)	0.32 (0.02 to 3.78)	1.04 (0.11 to 9.97)		1.11 (0.13 to 10.59)	1.06 (0.06 to 16.61)	5.21 (0.25 to 108.85)	0.97 (0.08 to 12.68)
**FP + SAL**	0.58 (0.14 to 2.01)	0.29 (0.05 to 1.07)	0.94 (0.34 to 2.01)	0.90 (0.09 to 7.77)		0.96 (0.12 to 5.70)	4.71 (0.51 to 40.45)	0.88 (0.17 to 3.56)
**FP + VI**	0.59 (0.08 to 4.62)	0.30 (0.03 to 2.20)	0.98 (0.18 to 5.31)	0.94 (0.06 to 15.80)	1.04 (0.18 to 8.00)		4.90 (0.36 to 75.19)	0.91 (0.11 to 7.54)
**Montelukast**	0.12 (0.01 to 1.28)	**0.06 (0.00 to 0.66)**	0.20 (0.02 to 1.63)	0.19 (0.01 to 3.97)	0.21 (0.02 to 1.95)	0.20 (0.01 to 2.80)		0.19 (0.01 to 2.16)
**Placebo**	0.65 (0.22 to 2.01)	0.33 (0.08 to 1.14)	1.07 (0.30 to 3.94)	1.03 (0.08 to 13.20)	1.14 (0.28 to 5.75)	1.09 (0.13 to 9.30)	5.37 (0.46 to 70.11)	

The table compares the effect estimate for an intervention in the row with an intervention in a column (TRT 1 vs. TRT 2).
OR (95% CrI) (15 studies, 3014 participants, 2447 events) Reference treatment: FP ─ DIC: 2836.9; Residual deviance: 2808.4 (on 3014 data points)
OR > 1 favours treatment 1 (the probability of having good/total asthma control was modelled).
All available data included (only IPD) – IPD = Individual Participant Data available. Results with CrI that exclude the OR value of 1 are highlighted in bold.
FF = fluticasone furoate; VI = vilanterol; FP = fluticasone propionate; TRT 1 = treatment 1; TRT 2 = treatment 2; OR = odds ratio, CrI = credibility interval; DIC = deviance information criterion;
NA = not available.

TABLE S16 FEV_1 Bayesian fixed effect network meta-analysis (MD[a], 95% CrI) with IPD and AgD (Analysis A3: 23 trials, 2518 participants)

TRT 1 / TRT 2	ICS Low	ICS Medium	ICS High	ICS unknown dose	ICS Low + LABA	ICS Medium + LABA	ICS High + LABA	ICS+LTRA	LTRA	Placebo
**ICS Low**	○	-0.02 (-0.13 to 0.09)	-0.16 (-0.46 to 0.15)	0.27 (-0.95 to 1.52)	-0.02 (-0.10 to 0.05)	**-0.71 (-1.06 to -0.35)**	0.29 (-0.05 to 0.64)	0.23 (-0.56 to1.04)	-0.15 (-0.63 to 0.33)	**0.15 (0.04 to 0.27)**
**ICS Medium**	0.02 (-0.09 to 0.13)	○	-0.14 (-0.45 to 0.16)	0.29 (-0.93 to 1.53)	-0.01 (-0.10 to 0.09)	**-0.69 (-1.05 to -0.33)**	0.30 (-0.04 to 0.66)	0.25 (-0.55 to 1.05)	-0.13 (-0.63 to 0.36)	**0.17 (0.01 to 0.33)**
**ICS High**	0.16 (-0.15 to 0.46)	0.14 (-0.16 to 0.45)	○	0.44 (-0.83 to 1.72)	0.14 (-0.17 to 0.43)	**-0.54 (-0.81 to -0.24)**	**0.45 (0.25 to 0.64)**	0.39 (-0.46 to 1.25)	0.02 (-0.55 to 0.58)	0.32 (-0.01 to 0.63)
**ICS unknown dose**	-0.27 (-1.52 to 0.95)	-0.29 (-1.53 to 0.93)	-0.44 (-1.72 to 0.83)	○	-0.30 (-1.54 to 0.92)	-0.98 (-2.27 to 0.30)	0.01 (-1.27 to 1.28)	-0.05 (-1.01 to 0.91)	-0.42 (-1.75 to 0.90)	-0.12 (-1.37 to 1.11)
**ICS Low + LABA**	0.02 (-0.05 to 0.10)	0.01 (-0.09 to 0.10)	-0.14 (-0.43 to 0.17)	0.30 (-0.92 to 1.54)	○	**-0.68 (-1.04 to -0.33)**	0.31 (-0.03 to 0.66)	0.25 (-0.54 to 1.06)	-0.12 (-0.61 to 0.36)	**0.18 (0.04 to 0.31)**
**ICS Medium + LABA**	**0.71 (0.35 to 1.06)**	**0.69 (0.33 to 1.05)**	**0.54 (0.24 to 0.81)**	0.98 (-0.30 to 2.27)	**0.68 (0.33 to 1.04)**	○	**0.99 (0.67 to 1.27)**	**0.94 (0.07 to 1.82)**	0.56 (-0.04 to 1.15)	**0.86 (0.49 to 1.24)**
**ICS High + LABA**	-0.29 (-0.64 to 0.05)	-0.30 (-0.66 to 0.04)	**-0.45 (-0.64 to -0.25)**	-0.01 (-1.28 to 1.27)	-0.31 (-0.66 to 0.03)	**-0.99 (-1.27 to -0.67)**	○	-0.06 (-0.92 to 0.81)	-0.43 (-1.02 to 0.15)	-0.13 (-0.50 to 0.22)
**ICS+LTRA**	-0.23 (-1.04 to 0.56)	-0.25 (-1.05 to 0.55)	-0.39 (-1.25 to 0.46)	0.05 (-0.91 to 1.01)	-0.25 (-1.06 to 0.54)	**-0.94 (-1.82 to -0.07)**	0.06 (-0.81 to 0.92)	○	-0.38 (-1.31 to 0.55)	-0.07 (-0.90 to 0.72)
**LTRA**	0.15 (-0.33 to 0.63)	0.13 (-0.36 to 0.63)	-0.02 (-0.58 to 0.55)	0.42 (-0.90 to 1.75)	0.12 (-0.36 to 0.61)	-0.56 (-1.15 to 0.04)	0.43 (-0.15 to 1.02)	0.38 (-0.55 to 1.31)	○	0.30 (-0.19 to 0.80)
**Placebo**	**-0.15 (-0.27 to -0.04)**	**-0.17 (-0.33 to -0.01)**	-0.32 (-0.63 to 0.01)	0.12 (-1.11 to 1.37)	**-0.18 (-0.31 to -0.04)**	**-0.86 (-1.24 to -0.49)**	0.13 (-0.22 to 0.50)	0.07 (-0.72 to 0.90)	-0.30 (-0.80 to 0.19)	○

The table compares the effect estimate for an intervention in the row with an intervention in a column (TRT 1 vs. TRT 2).
[a] MD > 0 favours treatment 1; MD < 0 favours treatment 2. 95% CrIs that exclude the MD value of 0 are highlighted in bold.
FEV_1 (L): forced expiratory volume in 1 second; MD: mean difference; CrI: credibility interval; IPD: individual participant data; AgD: aggregate data; TRT: treatment; ICS: inhaled corticosteroid; LABA: Long-Acting β_2-Agonist; LTRA: Leukotriene Receptor Antagonist

Table S17. Bayesian random-effects network meta-analysis (IPD and AgD) for FEV_1. ICS grouped when combined with LABA – Analysis B3

TRT 1 \ TRT 2	ICS Low	ICS Medium	ICS High	ICS unknown dose	ICS+LABA	ICS+LTRA	LTRA	ICS + Theophylline	Placebo
**ICS Low**		0.00<br>(-0.14 to 0.14)<br>*-0.06 (-1.64 to 1.47)*	-0.15<br>(-0.37 to 0.07)<br>*-0.38 (-2.77 to 2.08)*	0.30<br>(-0.97 to 1.60)	-0.02<br>(-0.11 to 0.08)<br>*0.00 (-0.12 to 0.17)*	0.24<br>(-0.58 to 1.09)	-0.15<br>(-0.63 to 0.35)<br>*-0.10 (-0.56 to 0.41) ***	NA	**0.16**<br>**(0.01 to 0.30)**<br>*0.15 (-0.17 to 0.46)*
**ICS Medium**	0.00<br>(-0.14 to 0.14)<br>*0.06 (-1.47 to 1.64)*		-0.15<br>(-0.38 to 0.09)<br>*-0.20 (-0.64 to 2.28) ***	0.30<br>(-0.96 to 1.59)	-0.02<br>(-0.13 to 0.10)<br>*0.01 (-0.30 to 0.38)*	0.24<br>(-0.57 to 1.08)<br>*0.76 (-0.17 to 1.69) ***	-0.14<br>(-0.65 to 0.36)	NA	0.16<br>(-0.04 to 0.35)<br>*0.12 (-1.03 to 1.29)*
**ICS High**	0.15<br>(-0.07 to 0.37)<br>*0.38 (-2.08 to 2.77)*	0.15<br>(-0.09 to 0.38)<br>*0.20 (-0.28 to 0.63) ***		0.45<br>(-0.83 to 1.76)	0.13<br>(-0.08 to 0.35)<br>*-0.28 (-3.22 to 2.48)*	0.39<br>(-0.43 to 1.26)	0.01<br>(-0.53 to 0.54)	NA	**0.31**<br>**(0.05 to 0.57)**<br>*0.40 (-0.14 to 0.96) ***
**ICS unknown dose**	-0.30<br>(-1.60 to 0.97)	-0.30<br>(-1.59 to 0.96)	-0.45<br>(-1.76 to 0.83)		-0.32<br>(-1.61 to 0.95)	-0.05<br>(-1.02 to 0.91)<br>*not calculated*	-0.44<br>(-1.81 to 0.91)	NA	-0.14<br>(-1.44 to 1.13)
**ICS+LABA**	0.02<br>(-0.08 to 0.11)<br>*0.00 (-0.17 to 0.12)*	0.02<br>(-0.10 to 0.13)<br>*0.01 (-0.38 to 0.30)*	-0.13<br>(-0.35 to 0.08)<br>*0.28 (-2.48 to 3.22)*	0.32<br>(-0.95 to 1.61)		0.26<br>(-0.55 to 1.10)<br>*-0.02 (-0.76 to 0.77) ***	-0.13<br>(-0.61 to 0.36)<br>*-0.20 (-0.74 to 0.34) ***	NA	0.18<br>(0.00 to 0.34)<br>*0.20 (-0.29 to 0.76) ***
**ICS+LTRA**	-0.24<br>(-1.09 to 0.58)	-0.24<br>(-1.08 to 0.57)<br>*-0.78 (-1.64 to 0.14) ***	-0.39<br>(-1.26 to 0.43)	0.05<br>(-0.91 to 1.02)<br>*not calculated*	-0.26<br>(-1.10 to 0.55)<br>*0.02 (-0.72 to 0.77) ***		-0.39<br>(-1.37 to 0.56)	NA	-0.09<br>(-0.94 to 0.73)
**LTRA**	0.15<br>(-0.35 to 0.63)<br>*0.10 (-0.40 to 0.53) ***	0.14<br>(-0.36 to 0.65)	-0.01<br>(-0.54 to 0.53)	0.44<br>(-0.91 to 1.81)	0.13<br>(-0.36 to 0.61)<br>*0.20 (-0.3 to 0.73) ***	0.39<br>(-0.56 to 1.37)		NA	0.30<br>(-0.21 to 0.81)
**ICS + Theophylline**	NA	NA	NA	NA	NA	NA	NA		NA
**Placebo**	**-0.16**<br>**(-0.30 to -0.01)**<br>*-0.15 (-0.46 to 0.17)*	-0.16<br>(-0.35 to 0.04)<br>*-0.12 (-1.29 to 1.03)*	**-0.31**<br>**(-0.57 to -0.05)**<br>*-0.40 (-0.92 to 0.12) ***	0.14<br>(-1.13 to 1.44)	-0.18<br>(-0.34 to 0.00)<br>*-0.20 (-0.75 to 0.27) ***	0.09<br>(-0.73 to 0.94)	-0.30<br>(-0.81 to 0.21)	NA	

The table compares the effect estimate for an intervention in the row with an intervention in a column (TRT 1 vs. TRT 2).
MD (95% CrI) from NMA with direct results from pairwise meta-analyses in Italics; 22 studies, 2486 patients; Reference treatment: ICS+LABA; DIC; 1768.4, Residual deviance: 2129.2 (on 2175 data points)
* MD > 0 favours treatment 1; MD < 0 favours treatment 2. Results with CrI that excludes the MD value of 0 are highlighted in bold. ** Estimates from Bayesian linear regression models (Stan).
TRT 1 = treatment 1; TRT 2 = treatment 2; FEV_1 = forced expiratory volume in 1 second; ICS = inhaled corticosteroid; LABA = long-acting beta-agonist; LTRA = leukotriene receptor antagonist; MD = mean difference; CrI = credibility interval; DIC = deviance information criterion; NA: not available.

Table S18. Bayesian fixed effect network meta-analysis (IPD only) for FEV_1 (individual compounds) – Analysis C3

TRT 1 \ TRT 2	FF	FF + VI	FP	FP + Montelukast	FP + SAL	FP + VI	Montelukast	Placebo
**FF**		-0.05 (-0.22 to 0.12)	0.07 (-0.05 to 0.19)	0.31 (-0.49 to 1.16)	0.05 (-0.09 to 0.20)	0.05 (-0.11 to 0.21)	-0.08 (-0.57 to 0.41)	**0.18 (0.05 to 0.30)**
**FF + VI**	0.05 (-0.12 to 0.22)		0.12 (-0.08 to 0.32)	0.37 (-0.44 to 1.23)	0.10 (-0.11 to 0.32)	0.10 (-0.12 to 0.33)	-0.02 (-0.54 to 0.49)	**0.23 (0.03 to 0.43)**
**FP**	-0.07 (-0.19 to 0.05)	-0.12 (-0.19 to 0.08)		0.25 (-0.55 to 1.08)	-0.02 (-0.09 to 0.06)	-0.02 (-0.12 to 0.09)	-0.14 (-0.62 to 0.33)	0.11 (-0.04 to 0.26)
**FP + Montelukast**	-0.31 (-1.16 to 0.49)	-0.37 (-1.23 to 0.44)	-0.25 (-1.08 to 0.55)		-0.26 (-1.10 to 0.53)	-0.26 (-1.10 to 0.53)	-0.39 (-1.36 to 0.55)	-0.14 (-0.99 to 0.66)
**FP + SAL**	-0.05 (-0.20 to 0.09)	-0.10 (-0.32 to 0.11)	0.02 (-0.06 to 0.09)	0.26 (-0.53 to 1.10)		0.00 (-0.13 to 0.13)	-0.13 (-0.61 to 0.35)	0.12 (-0.05 to 0.29)
**FP + VI**	-0.05 (-0.21 to 0.11)	-0.10 (-0.33 to 0.12)	0.02 (-0.09 to 0.12)	0.26 (-0.53 to 1.10)	0.00 (-0.13 to 0.13)		-0.13 (-0.62 to 0.36)	0.12 (-0.06 to 0.31)
**Montelukast**	0.08 (-0.41 to 0.57)	0.02 (-0.49 to 0.54)	0.14 (-0.33 to 0.62)	0.39 (-0.55 to 1.36)	0.13 (-0.35 to 0.61)	0.13 (-0.36 to 0.62)		0.25 (-0.25 to 0.75)
**Placebo**	**-0.18 (-0.30 to -0.05)**	**-0.23 (-0.43 to -0.03)**	-0.11 (-0.26 to 0.04)	0.14 (-0.66 to 0.99)	-0.12 (-0.29 to 0.05)	-0.12 (-0.31 to 0.06)	-0.25 (-0.75 to 0.25)	

The table compares the effect estimate for an intervention in the row with an intervention in a column (TRT 1 vs. TRT 2).
MD (95% CrI) (17 studies, 1984 participants). Reference treatment: FP ─ DIC: 1087.7; Residual deviance: 1943.1 (on 1984 data points)
MD > 0 favours treatment 1; MD < 0 favours treatment 2. Results with CrI that excludes the MD value of 0 are highlighted in bold.
IPD = Individual Participant Data available; FEV_1 = forced expiratory volume in 1 second; FF = fluticasone furoate; VI = vilanterol; FP = fluticasone propionate; TRT 1 = treatment 1; TRT 2 = treatment 2; MD = mean difference; CrI = credibility interval; DIC = deviance information criterion.

Table S19. Direct pairwise comparisons of treatment classes (IPD and AgD) for quality of life outcome

Direct comparison TRT 1 vs TRT 2	Data[a]	Author Year (participants on each treatment)	Studies (N)	Participants (N)	QoL Tool	Total score at the last visit (average score) TRT 1 vs TRT 2 Mean (SD)	Bayesian meta-analysis			
							Fixed-effect model MD (95% CrI)	DIC	Random effects model MD (95% CrI)	DIC
ICS+LABA vs ICS Low	IPD<br>AgD	Lenney 2013 (15 vs 10) (*)<br>Murray 2011 (86 vs 87) (*)<br>Pearlman 2009 (91 vs 79) (*)<br>Wechsler 2019 (51 vs 22)	4	243 vs 198	PAQLQ	5.4 (1.6) vs 6.3 (0.9)<br>5.9 (0.8) vs 5.9 (0.8)<br>5.8 (0.9) vs 5.8 (0.9)<br>6.2 (0.9) vs 5.7 (1.2)	0.01 (-0.17; 0.19)	431.1	0.06 (-0.53; 0.68)	433.1
ICS+LABA vs ICS Medium	IPD	Lemanske 2010 (8 vs 6) (*)<br>Thomas 2014 (11 vs 11) (*)	2	19 vs 17	PAQLQ	5.8 (1.0) vs 5.3 (1.4)<br>5.4 (1.1) vs 6.4 (0.6)	**-0.91 (-1.53; -0.29)**	37.6	-0.89 (-2.27; 0.50)	38.3
ICS+LTRA vs ICS Medium	IPD	Lemanske 2010 (13 vs 6)<br>Thomas 2014 (11 vs 11)	2	24 vs 17	PAQLQ	6.2 (1.1) vs 6.6 (0.3)<br>6.1 (0.9) vs 6.4 (0.6)	-0.35 (-0.85; 0.18)	42.5	-0.35 (-1.68; 0.95)	43.2
ICS+LTRA vs ICS+LABA	IPD<br>AgD	Lemanske 2010 (13 vs 8)<br>Lenney 2013 (12 vs 15) (*)<br>Thomas 2014(11 vs 11) (*)	3	36 vs 34	PAQLQ	6.2 (1.1) vs 5.8 (1.0)<br>6.3 (0.9) vs 5.4 (1.6)<br>6.1 (0.9) vs 5.4 (1.1)	0.59 (-0.11; 1.30)	46.7	0.60 (-0.56; 1.76)	47.6
ICS Low vs ICS High	IPD	Wechsler 2019 (22 vs 22)	1	22 vs 22	PAQLQ	5.7 (1.2) vs 6.3 (0.9)	Bayesian linear regression model (Stan): -0.61 (-1.23; 0.03)			
ICS+LABA vs ICS High	IPD	Wechsler 2019 (51 vs 22)	1	51 vs 22	PAQLQ	6.2 (0.9) vs 6.3 (0.9)	Bayesian linear regression model (Stan): -0.13 (-0.58; 0.32)			
ICS Low vs ICS+LTRA	AgD	Lenney 2013 (10 vs 12) (*)	1	10 vs 12	PAQLQ	6.3 (0.9) vs 6.3 (0.9)	Bayesian linear regression model (Stan): not estimable**			
ICS+LABA vs ICS Low	IPD	Bernstein 2015 (24 vs 16)<br>Bleecker 2014 (13 vs 14)	2	37 vs 30	AQLQ	5.5 (1.1) vs 5.4 (1.1)<br>6.3 (0.7) vs 5.9 (0.6)	0.31 (-0.15; 0.75)	14.4	0.27 (-1.10; 1.62)	16
ICS+LABA vs ICS High	IPD	O'Byrne 2014 (3 vs 5) (§)<br>Wechsler 2019 (21 vs 10)	2	24 vs 15	AQLQ	6.1 (0.3) vs 5.6 (1.5)<br>6.1 (0.8) vs 6.5 (0.5)	-0.17 (-0.50; 0.17)	113.3	-0.03 (-1.57; 1.72)	114.2
placebo vs ICS Low	IPD	Bleecker 2014 (21 vs 14)<br>Lötvall 2014 b (14 vs 15)	2	35 vs 29	AQLQ	5.5 (0.9) vs 5.9 (0.6)<br>5.9 (0.7) vs 6.2 (0.6)	-0.32 (-0.66; 0.03)	59.7	-0.29 (-1.45; 1.03)	60.4
ICS Medium vs ICS Low	IPD	Lötvall 2014 b (10 vs 15)	1	10 vs 15	AQLQ	5.6 (1.3) vs 6.2 (0.6)	Bayesian linear regression model (Stan): -0.55 (-1.33; 0.23)			
placebo vs ICS Medium	IPD	Lötvall 2014 b (14 vs 10)	1	14 vs 10	AQLQ	5.9 (0.7) vs 5.6 (1.3)	Bayesian linear regression model (Stan): 0.31 (-0.50; 1.16)			
placebo vs ICS+LABA	IPD	Bleecker 2014 (21 vs 13)	1	21 vs 13	AQLQ	5.5 (0.9) vs 6.3 (0.7)	Bayesian linear regression model (Stan): **-0.81 (-1.39; -0.27)**			

MD > 0 favors TRT 1; MD < 0 favors TRT 2
[a]All data available were used (IPD and AgD where possible); IPD = individual participant data; AgD = aggregate data
(*) ICS Low+LABA
(§) ICS High+LABA
** Same mean and SD in both arms (constant)
TRT = treatment; QoL = quality of life; SD = standard deviation; MD = mean difference; CrI = credibility interval; DIC = deviance information criterion; NA = not available; ICS = inhaled corticosteroids; LABA = long-acting beta-agonist; LTRA = leukotriene receptor antagonist; AQLQ = asthma quality of life questionnaire; PAQLQ = paediatric asthma quality of life questionnaire.

Table S20. Hospital admissions

Author Year	Data	Treatment class	Compounds	No. of patients	Was the patient hospitalized due to an asthma attack? No. (%)
Bateman 2014	IPD	ICS Low	FF	102	0
		ICS+LABA	FF+VI	111	3 (2.7%)
De Blic 2009	IPD	ICS Medium	FP	153	0
		ICS+LABA	FP+SAL	150	1 (0.7%)
Stempel 2016 a	IPD	ICS Medium	FP	813	4 (0.5%)
		ICS+LABA	FP+SAL	818	5 (0.6%)
Stempel 2016 b	IPD	ICS High	FP	40	0
		ICS Low	FP	15	0
		ICS Medium	FP	50	0
		ICS+LABA	FP+SAL	117	2 (1.7%)
Wechsler 2019	IPD	ICS High	FP	45	1 (2.2%)
		ICS Low	FP	33	0
		ICS+LABA	FP+SAL	93	1 (1.1%)

IPD: individual participant data; LABA: long-acting beta$_2$-agonist; FF: fluticasone furoate; VI: vilanterol; FP: fluticasone propionate; SAL: salmeterol.

Network meta-regression to explore effect modifiers

We compared the DIC between network meta-regression (NMR) models with and without interaction terms and found no overall evidence of interactions in any of the models. However, for some models there were non-zero interaction regression coefficients, which are described further below. The lack of consistent robust statistical evidence and clinical rationale to support these suggested effects, along with issues of small numbers of patients in some analyses suggests that these results should be viewed very cautiously, they are potentially spurious and should not be over-interpreted. Further research would be needed to explore these effects in more detail, and we note that recommendations regarding the treatment and care of patients would not differ according to any of the studied covariates.

Exacerbation

We did not detect any "treatment by covariate" interaction for age (24 trials, 4929 participants), sex (26 trials, 5349 participants), eczema (8 trials, 2469 participants), and eosinophilia (13 trials, 1898 participants), based on interpretation of the 95% CrI of the interaction regression coefficient and comparison of DIC for models with and without interactions (eTable 18). For the covariates ethnicity (27 trials, 5645 participants) and baseline severity (21 trials, 2916 participants), the DIC comparison did not suggest evidence for an interaction, and the fixed effect model without interactions was the most appropriate model overall. However, the 95% CrI of the interaction regression coefficients (difference in the log odds ratio for levels of the covariate) excludes zero for some comparisons:
(1) *ethnicity*: ICS Medium (OR, -1.25; 95% CrI, -2.47 to -0.18), ICS+LABA (OR, -1.09; 95% CrI, -2.27 to -0.06), and placebo (OR, -2.70; 95% CrI, -5.19 to -0.24) against ICS Low;
(2) *baseline severity*: ICS Medium (OR, 2.11; 95% CrI, 0.32 to 3.89) against ICS Low;
suggesting possible interaction effects (Table S22). The corresponding subgroup level effects have 95% credibility intervals that overlap across subgroup levels for ethnicity and baseline severity (Tables S23, S24). Furthermore, the 95% credibility intervals mostly include the null effect (unity) apart from comparisons with placebo and LTRA for ethnicity with results that are consistent in clinical interpretation with main effect analyses (Table S7). The NMR for baseline severity suggests an advantage to ICS Low over ICS Medium for severe asthma (OR, 0.04; 95% CrI, 0.00 to 0.68) but this is based on sparse data (Table S22) and isn't supported by clinical rationale. Overall, we do not consider that the network meta-regression analyses provide sufficiently robust, conclusive evidence of interaction effects to justify any deviation from the main network meta-analysis results (Table S7).

Asthma control

The network meta-regression analyses for asthma control did not identify any effect modifiers based on interpretation of the 95% CrI of the estimated interaction regression coefficients and comparison of DIC for models with and without interactions (Tables S25, S26) for all covariates considered: age (15 trials, 2998 participants), sex (15 trials, 2998 participants), ethnicity (15 trials, 2998 participants), eczema (6 trials, 1968 participants), eosinophilia (12 trials, 1192 participants), and baseline severity (13 trials, 1074 participants). No AgD were available.

FEV_1

The network meta-regression analyses for FEV_1 did not identify "treatment by covariate" interactions based on the 95% CrI and comparison of DIC for models with and without interactions for covariates age (19 trials, 1689 participants), ethnicity (19 trials, 1908 participants), and eczema (5 trials, 455 participants) (Table S27). For the covariate "*sex*" (20 trials, 1937 participants), although the comparison of DIC of different models did not suggest an interaction (random-effects without interactions is the most appropriate model), the 95% CrI for the "treatment by sex" interaction regression coefficient (difference in the MD for females compared to the MD for males) excludes the zero null effect for LTRA vs ICS+LABA (Table S28), and corresponding subgroup level effects suggest benefit for LTRA for females (Table S29). However, we do not consider these results to be sufficiently robust to claim a conclusive interaction as the NMR included only 3 females on LTRA, and the overall comparison of DIC did not support a model with interactions. Similarly, for the covariate "*eosinophilia*" (11 trials, 1024 participants), the comparison of DIC of different models did not suggest an interaction (fixed effect without interactions is the most appropriate model), but the 95% CrI for the "treatment by eosinophilia" interaction regression coefficient excludes the zero-null effect for ICS+LABA vs ICS Low (Table S28). However, the 95% credibility intervals for corresponding subgroup level MDs overlap between subgroup levels for all comparisons (Table S30); therefore, we conclude that there is insufficient evidence to suggest an interaction between treatment and "*eosinophilia*".

Table S21. Model comparison assessments from network meta-analysis models including interactions for the outcome exacerbation

Interaction	Model	Number of trials (number of participants)	Number of data points	Residual deviance	Effective number of parameters (Pd)	Deviance information Criterion (DIC)	Between trial standard deviation
**Treatment by *age***	**Fixed-effect without interactions**	24 (4,929)	4929	2052.7	27.4	2080.0	-
	**Fixed-effect with interactions**	24 (4,929)	4929	2052.0	33.1	2085.1	-
	**Random-effects with interactions**	24 (4,929)	4929	2049.1	36.4	2085.5	0.47 (0.02, 1.37)
**Treatment by *sex***	**Fixed-effect without interactions**	26 (5,349)	5349	2216.2	29.5	2245.7	-
	**Fixed-effect with interactions**	26 (5,349)	5349	2216.7	34.7	2251.5	-
	**Random-effects with interactions**	26 (5,349)	5349	2215.1	38.0	2253.1	0.34 (0.01, 1.01)
**Treatment by *ethnicity***	**Fixed-effect without interactions**	27 (5,645)	5351	2215.8	30.3	2246.1	-
	**Fixed-effect with interactions**	27 (5,645)	5351	2210.3	34.8	2245.0	-
	**Random-effects with interactions**	27 (5,645)	5351	2209.7	37.3	2246.9	0.22 (0.01, 0.85)
**Treatment by *eczema***	**Fixed-effect without interactions**	8 (2,469)	2439	1312.4	12.3	1324.7	-
	**Fixed-effect with interactions**	8 (2,469)	2439	1313.9	16.7	1330.6	-
	**Random-effects with interactions**	8 (2,469)	2439	1313.4	18.5	1331.9	0.69 (0.02, 2.44)
**Treatment by *eosinophilia***	**Fixed-effect without interactions**	13 (1,898)	1898	600.3	15.9	616.1	-
	**Fixed-effect with interactions**	13 (1,898)	1898	601.8	20.3	622.1	-
	**Random-effects with interactions**	13 (1,898)	1898	596.0	23.6	619.7	1.04 (0.09, 3.17)
**Treatment by *baseline severity*** *(based on FEV_1)*	**Fixed-effect without interactions**	21 (2,916)	2916	741.7	22.1	763.8	-
	**Fixed-effect with interactions**	21 (2,916)	2916	740.2	25.4	765.7	-
	**Random-effects with interactions**	21 (2,916)	2916	736.0	29.8	765.9	0.87 (0.04, 3.07)

Table S22. Parameter estimates (Posterior mean [95% CrI]) from NMR models including interactions for the outcome exacerbation

Interaction	Comparison	Fixed-effect with interactions		Random-effects with interactions	
		Log OR at the mean covariate value (95% CrI)	Regression coefficient treatment by covariate interaction (95% CrI)	Log OR at the mean covariate value (95% CrI)	Regression coefficient treatment by covariate interaction (95% CrI)
**Treatment by *age* (24 trials, 4929 participants)**	ICS High vs ICS Low	-0.33 (-1.05 to 0.39)	0.02 (-0.16 to 0.19)	-0.31 (-1.33 to 0.74)	0.00 (-0.19 to 0.19)
	ICS Medium vs ICS Low	-0.19 (-0.81 to 0.42)	0.11 (-0.04 to 0.26)	-0.29 (-1.35 to 0.66)	0.11 (-0.04 to 0.27)
	ICS+LABA vs ICS Low	-0.28 (-0.78 to 0.22)	0.09 (-0.04 to 0.21)	-0.23 (-0.86 to 0.47)	0.07 (-0.08 to 0.21)
	LTRA vs ICS Low	-2.74 (-9.05 to 2.74)	-0.65 (-1.60 to 0.19)	-2.83 (-9.25 to 2.89)	-0.66 (-1.60 to 0.19)
	placebo vs ICS Low	**2.41 (0.65 to 4.44)**	0.20 (-0.23 to 0.67)	**2.28 (0.18 to 4.52)**	0.21 (-0.22 to 0.69)
**Treatment by *sex* (26 trials, 5349 participants)**	ICS High vs ICS+LABA	-0.23 (-0.78 to 0.30)	0.27 (-0.56 to 1.11)	-0.26 (-1.03 to 0.47)	0.28 (-0.56 to 1.12)
	ICS Low vs ICS+LABA	0.24 (-0.26 to 0.72)	-0.02 (-0.80 to 0.75)	0.22 (-0.40 to 0.80)	-0.03 (-0.80 to 0.76)
	ICS Medium vs ICS+LABA	0.12 (-0.18 to 0.42)	-0.28 (-0.85 to 0.28)	0.13 (-0.45 to 0.73)	-0.28 (-0.84 to 0.27)
	LTRA vs ICS+LABA	1.53 (-0.03 to 3.27)	0.94 (-0.84 to 2.76)	1.51 (-0.34 to 3.44)	0.95 (-0.84 to 2.80)
	placebo vs ICS+LABA	**2.33 (0.35 to 4.49)**	-1.80 (-5.21 to 0.56)	**2.28 (0.18 to 4.56)**	-1.78 (-5.06 to 0.55)
**Treatment by *ethnicity* (27 trials, 5645 participants)**	ICS High vs ICS Low	-0.52 (-1.51 to 0.32)	-0.55 (-2.97 to 2.65)	-0.54 (-1.66 to 0.41)	-0.50 (-2.97 to 2.91)
	ICS Medium vs ICS Low	-0.08 (-0.66 to 0.52)	**-1.25 (-2.47 to -0.18)**	-0.06 (-0.77 to 0.70)	**-1.21 (-2.40 to -0.11)**
	ICS+LABA vs ICS Low	-0.19 (-0.70 to 0.32)	**-1.09 (-2.27 to -0.06)**	-0.18 (-0.75 to 0.39)	-1.03 (-2.20 to 0.04)
	LTRA vs ICS Low	not estimable	not estimable	not estimable	not estimable
	placebo vs ICS Low	**1.19 (0.59 to 1.80)**	**-2.70 (-5.19 to -0.24)**	**1.24 (0.43 to 2.15)**	**-2.61 (-5.14 to -0.06)**
**Treatment by *eczema* (8 trials, 2469 participants)**	ICS High vs ICS Medium	-0.01 (-1.34 to 1.52)	-1.89 (-4.40 to 0.43)	0.00 (-1.88 to 2.02)	-1.88 (-4.46 to 0.45)
	ICS Low vs ICS Medium	0.07 (-1.14 to 1.52)	-1.04 (-3.06 to 0.63)	0.05 (-1.94 to 2.21)	-0.99 (-3.06 to 0.71)
	ICS+LABA vs ICS Medium	-0.04 (-1.20 to 1.37)	-1.29 (-3.30 to 0.37)	0.01 (-1.74 to 1.97)	-1.22 (-3.29 to 0.48)
	ICS+LTRA vs ICS Medium	not estimable	not estimable	not estimable	not estimable
	LTRA vs ICS Medium	1.49 (-0.40 to 3.48)	-0.67 (-3.34 to 2.05)	1.46 (-1.18 to 4.18)	-0.63 (-3.39 to 2.13)
	placebo vs ICS Medium	not estimable	not estimable	not estimable	not estimable
**Treatment by *eosinophilia* (13 trials, 1898 participants)**	ICS High vs ICS Low	-1.20 (-2.72 to 0.02)	-1.38 (-4.73 to 1.18)	-1.67 (-4.91 to 0.57)	-1.38 (-4.66 to 1.11)
	ICS Medium vs ICS Low	not estimable	not estimable	not estimable	not estimable
	ICS+LABA vs ICS Low	-0.40 (-0.98 to 0.16)	-0.28 (-1.31 to 0.75)	-0.44 (-1.94 to 0.98)	-0.25 (-1.31 to 0.79)
	LTRA vs ICS Low	1.12 (-0.45 to 2.86)	0.18 (-2.19 to 2.39)	1.09 (-2.36 to 4.37)	0.19 (-2.22 to 2.41)
	placebo vs ICS Low	**2.15 (0.29 to 4.26)**	1.32 (-0.79 to 3.61)	1.88 (-0.97 to 4.76)	1.37 (-0.78 to 3.69)
**Treatment by *baseline severity* (21 trials, 2916 participants)**	ICS High vs ICS Low	-0.38 (-1.31 to 0.55)	0.71 (-0.39 to 1.85)	-1.24 (-5.13 to 0.71)	0.65 (-0.47 to 1.80)
	ICS Medium vs ICS Low	0.04 (-1.57 to 1.61)	**2.11 (0.32 to 3.89)**	-0.31 (-3.02 to 1.81)	**2.01 (0.16 to 3.89)**
	ICS+LABA vs ICS Low	-0.10 (-0.74 to 0.55)	0.49 (-0.43 to 1.47)	-0.32 (-1.79 to 0.79)	0.39 (-0.59 to 1.40)
	placebo vs ICS Low	**2.40 (0.60 to 4.54)**	0.64 (-1.45 to 2.78)	2.22 (-0.48 to 4.98)	0.61 (-1.44 to 2.73)

Bold indicates that zero is excluded from the credibility interval. Regression coefficient: change in the log OR per unit increase in the covariate value.

Table S23. Odds ratios (95% CrI) from fixed effect NMR with "treatment by ethnicity" interactions for the outcome exacerbation

	TRT 1 \ TRT 2	ICS Medium	ICS High	ICS+LABA	LTRA	Placebo
**Hispanic or Latino (N = 1457)**	**ICS Low N=418**	0.43 (0.13 to 1.21)	1.12 (0.11 to 27.11)	0.54 (0.17 to 1.43)	*Not estimable*	**0.04 (0.01 to 0.28)**
	**ICS Medium N = 258**		2.61 (0.32 to 56.83)	1.26 (0.75 to 2.12)	*Not estimable*	**0.10 (0.01 to 0.62)**
	**ICS High N = 18**			0.48 (0.02 to 3.86)	*Not estimable*	**0.04 (0.00 to 0.61)**
	**ICS+LABA N = 698**				*Not estimable*	**0.08 (0.01 to 0.49)**
	**LTRA N = 3**					*Not estimable*
**Not Hispanic or Latino (N = 4188)**	**ICS Low N = 941**	1.49 (0.80 to 2.72)	1.93 (0.95 to 3.97)	1.60 (0.94 to 2.69)	0.26 (0.05 to 1.09)	0.61 (0.27 to 1.42)
	**ICS Medium N = 1014**		1.30 (0.69 to 2.51)	1.07 (0.75 to 1.52)	**0.17 (0.03 to 0.83)**	0.41 (0.15 to 1.13)
	**ICS High N = 226**			0.83 (0.47 to 1.42)	**0.13 (0.02 to 0.67)**	**0.31 (0.11 to 0.91)**
	**ICS+LABA N = 1824**				**0.16 (0.03 to 0.75)**	0.38 (0.15 to 1.00)
	**LTRA N = 27**					2.36 (0.45 to 15.03)

The table compares the effect estimate for an intervention in the row with an intervention in a column (TRT 1 vs. TRT 2).

OR > 1 favours TRT 2 (all data included, IPD and AgD where possible). 95% CrIs that exclude unity are highlighted in bold
N = number of participants; TRT = treatment; ICS = inhaled corticosteroids; LABA = long-acting beta$_2$-agonists; LTRA = leukotriene receptor antagonists.

Table S24. Odds ratios (95% CrI) from fixed effect NMR with "treatment by baseline severity" interactions for the outcome exacerbation

	TRT 1 \ TRT 2	ICS Medium	ICS High	ICS+LABA	Placebo*
**Mild (N = 1716, 60 events)**	**ICS Low N = 544**	2.64 (0.41 to 20.29)	2.05 (0.75 to 5.64)	1.39 (0.65 to 3.00)	0.12 (0.01 to 1.16)
		**ICS Medium N = 236**	0.78 (0.10 to 5.05)	0.53 (0.08 to 3.10)	**0.05 (0.00 to 0.76)**
			**ICS High N = 98**	0.68 (0.31 to 1.46)	**0.06 (0.01 to 0.64)**
				**ICS+LABA N = 788**	**0.09 (0.01 to 0.88)**
**Moderate (N = 1007, 40 events)**	**ICS Low N = 416**	0.32 (0.06 to 1.62)	1.00 (0.32 to 3.13)	0.85 (0.36 to 1.93)	**0.06 (0.01 to 0.48)**
		**ICS Medium N = 73**	3.16 (0.57 to 16.78)	2.69 (0.61 to 11.47)	0.20 (0.02 to 2.01)
			**ICS High N = 60**	0.85 (0.35 to 2.10)	**0.06 (0.01 to 0.58)**
				**ICS+LABA N = 392**	**0.08 (0.01 to 0.59)**
**Severe (N = 193, 5 events)**	**ICS Low N = 49**	**0.04 (0.00 to 0.68)**	0.49 (0.06 to 3.53)	0.52 (0.10 to 2.44)	0.03 (0.00 to 1.32)
		**ICS Medium N = 6**	12.68 (0.65 to 204.38)	13.60 (0.89 to 152.93)	0.89 (0.02 to 43.82)
			**ICS High N = 5**	1.06 (0.20 to 5.64)	0.07 (0.00 to 2.77)
				**ICS+LABA N = 130**	0.07 (0.00 to 2.27)

The table compares the effect estimate for an intervention in the row with an intervention in a column (TRT 1 vs. TRT 2).

OR > 1 favours TRT 2 (all data included, only IPD). 95% CrIs that exclude unity are highlighted in bold.
N = number of participants; TRT = treatment; ICS = inhaled corticosteroids; LABA = long-acting beta2-agonists;
*****placebo (mild), N = 50; (moderate) N = 66; (severe) N = 3.

Table S25. Model comparison assessments from network meta-analysis models including interactions for the outcome asthma control

Interaction	Model	Number of trials (number of participants)	Number of data points	Residual deviance	Effective number of parameters (Pd)	Deviance information Criterion (DIC)	Between trial standard deviation
**Treatment by *age***	**Random-effects without interactions**	15 (2998)	2998	2797.0	27.8	2824.8	0.43 (0.03,1.02)
	**Fixed-effect with interactions**	15 (2998)	2998	2804.6	29.2	2833.9	-
	**Random-effects with interactions**	15 (2998)	2998	2790.8	36.7	2827.5	0.75 (0.19,1.47)
**Treatment by *sex***	**Fixed-effect without interactions**	15 (2998)	2998	2800.7	22.5	2823.2	-
	**Fixed-effect with interactions**	15 (2998)	2998	2799.2	28	2827.2	-
	**Random-effects with interactions**	15 (2998)	2998	2793.1	33	2826.1	0.44 (0.03,1.06)
**Treatment by *ethnicity***	**Fixed-effect without interactions**	15 (2998)	2998	2802.6	22.7	2825.3	-
	**Fixed-effect with interactions**	15 (2998)	2998	2805.2	28.9	2834.1	-
	**Random-effects with interactions**	15 (2998)	2998	2798.4	34.7	2833.1	0.49 (0.04,1.11)
**Treatment by *eczema***	**Fixed-effect without interactions**	6 (1968)	1968	1607.3	12.3	1619.5	-
	**Fixed-effect with interactions**	6 (1968)	1968	1610.0	17.6	1627.6	-
	**Random-effects with interactions**	6 (1968)	1968	1608.6	17.6	1626.2	0.29(0.01,0.87)
**Treatment by *eosinophilia***	**Fixed-effect without interactions**	12 (1192)	1192	1326.2	19.5	1345.7	-
	**Fixed-effect with interactions**	12 (1192)	1192	1328.7	26.3	1355.0	-
	**Random-effects with interactions**	12 (1192)	1192	1325.1	30	1355.1	0.54 (0.02,1.52)
**Treatment by *Baseline severity (based on FEV_1)***	**Fixed-effect without interactions**	13 (1074)	1074	1187.2	20.5	1207.6	-
	**Fixed-effect with interactions**	13 (1074)	1074	1187.3	25.5	1212.7	-
	**Random-effects with interactions**	13 (1074)	1074	1177.8	30.8	1208.7	1.09 (0.08,2.78)

Table S26. Parameter estimates (Posterior mean [95% CrI]) from NMR models including interactions for the outcome asthma control

**Model**		**Fixed-effect NMA with interactions**		**Random-effects NMA with interactions**	
		**Log odds ratio at the mean covariate value (95% CrI)**	**Regression coefficient for the treatment by covariate interaction (95% CrI)**	**Log odds ratio at the mean covariate value (95% CrI)**	**Regression coefficient for the treatment by covariate interaction (95% CrI)**
**Treatment by *age***	ICS High vs ICS+LABA	-0.56 (-1.27 to 0.17)	0.01 (-0.15 to 0.17)	-0.98 (-2.36 to 0.22)	0.12 (-0.08 to 0.33)
	ICS Low vs ICS+LABA	-0.20 (-0.55 to 0.15)	0.01 (-0.07 to 0.10)	-0.51 (-1.38 to 0.23)	0.04 (-0.07 to 0.16)
	ICS Medium vs ICS+LABA	-0.09 (-0.37 to 0.20)	-0.07 (-0.15 to 0.01)	0.36 (-0.55 to 1.44)	-0.10 (-0.21 to 0.00)
	ICS+LTRA vs ICS+LABA	0.06 (-1.69 to 1.96)	-0.04 (-0.45 to 0.43)	0.19 (-2.06 to 2.59)	-0.04 (-0.45 to 0.43)
	LTRA vs ICS+LABA	-1.57 (-3.21 to 0.08)	-0.15 (-0.70 to 0.36)	-1.83 (-4.16 to 0.35)	-0.14 (-0.68 to 0.35)
	placebo vs ICS+LABA	-0.46 (-1.19 to 0.30)	-0.05 (-0.23 to 0.12)	-0.69 (-2.16 to 0.70)	-0.01 (-0.25 to 0.23)
**Treatment by *sex***	ICS High vs ICS+LABA	-0.43 (-0.98 to 0.15)	-0.08 (-1.05 to 0.86)	-0.45 (-1.27 to 0.37)	-0.04 (-1.00 to 0.92)
	ICS Low vs ICS+LABA	-0.17 (-0.50 to 0.15)	0.48 (-0.03 to 1.00)	-0.30 (-0.90 to 0.19)	0.48 (-0.03 to 0.99)
	ICS Medium vs ICS+LABA	-0.06 (-0.34 to 0.22)	0.14 (-0.34 to 0.63)	0.00 (-0.65 to 0.72)	0.14 (-0.35 to 0.62)
	ICS+LTRA vs ICS+LABA	not estimable	not estimable	not estimable	not estimable
	LTRA vs ICS+LABA	**-2.03 (-3.97 to -0.23)**	-1.85 (-5.50 to 1.16)	**-2.15 (-4.37 to -0.14)**	-1.85 (-5.63 to 1.26)
	placebo vs ICS+LABA	-0.48 (-1.12 to 0.18)	-0.49 (-1.57 to 0.58)	-0.58 (-1.58 to 0.35)	-0.56 (-1.65 to 0.53)
**Treatment by *ethnicity***	ICS High vs ICS+LABA	-0.53 (-1.09 to 0.05)	0.43 (-0.86 to 1.68)	-0.51 (-1.39 to 0.36)	0.22 (-1.12 to 1.53)
	ICS Low vs ICS+LABA	-0.17 (-0.49 to 0.16)	0.07 (-0.44 to 0.57)	-0.32 (-0.96 to 0.21)	0.15 (-0.39 to 0.69)
	ICS Medium vs ICS+LABA	-0.05 (-0.32 to 0.23)	-0.05 (-0.61 to 0.49)	0.05 (-0.66 to 0.84)	-0.03 (-0.60 to 0.52)
	ICS+LTRA vs ICS+LABA	0.49 (-1.51 to 2.92)	1.24 (-1.77 to 4.89)	0.51 (-1.67 to 3.12)	1.23 (-1.75 to 4.75)
	LTRA vs ICS+LABA	-1.49 (-3.21 to 0.25)	-1.00 (-4.45 to 1.82)	-1.59 (-3.63 to 0.41)	-1.00 (-4.56 to 1.79)
	placebo vs ICS+LABA	-0.52 (-1.15 to 0.15)	0.94 (-0.22 to 2.10)	-0.69 (-1.77 to 0.28)	1.17 (-0.12 to 2.54)
**Treatment by *eczema***	ICS High vs ICS+LABA	**-0.82 (-1.45 to -0.18)**	-0.02 (-1.12 to 1.07)	-0.73 (-1.49 to 0.13)	-0.09 (-1.21 to 1.01)
	ICS Low vs ICS+LABA	**-0.91 (-1.76 to -0.04)**	0.52 (-0.73 to 1.74)	-0.79 (-1.69 to 0.18)	0.45 (-0.84 to 1.70)
	ICS Medium vs ICS+LABA	-0.06 (-0.35 to 0.22)	0.50 (-0.16 to 1.18)	0.04 (-0.48 to 0.81)	0.47 (-0.20 to 1.16)
	ICS+LTRA vs ICS+LABA	0.16 (-1.64 to 2.14)	0.02 (-3.06 to 3.58)	0.22 (-1.53 to 2.11)	-0.03 (-2.67 to 2.96)
	LTRA vs ICS+LABA	**-2.28 (-4.07 to -0.53)**	0.73 (-1.72 to 3.29)	**-1.98 (-3.79 to -0.21)**	0.55 (-1.70 to 2.89)
**Treatment by *eosinophilia***	ICS High vs ICS+LABA	0.22 (-0.60 to 1.08)	0.99 (-0.51 to 2.70)	0.11 (-1.30 to 1.35)	0.98 (-0.55 to 2.70)
	ICS Low vs ICS+LABA	-0.05 (-0.39 to 0.31)	0.28 (-0.32 to 0.88)	-0.14 (-0.89 to 0.51)	0.27 (-0.32 to 0.87)
	ICS Medium vs ICS+LABA	1.13 (-0.55 to 3.32)	-1.29 (-4.83 to 1.58)	1.23 (-0.66 to 3.64)	-1.30 (-4.82 to 1.67)
	ICS+LTRA vs ICS+LABA	0.45 (-1.45 to 2.50)	1.32 (-1.69 to 4.85)	0.48 (-1.70 to 2.78)	1.32 (-1.63 to 4.96)
	LTRA vs ICS+LABA	-1.78 (-3.70 to 0.08)	1.28 (-1.39 to 3.96)	-1.88 (-4.23 to 0.35)	1.30 (-1.43 to 4.05)
	placebo vs ICS+LABA	-0.33 (-1.05 to 0.40)	-0.36 (-1.62 to 0.89)	-0.38 (-1.52 to 0.77)	-0.42 (-1.71 to 0.87)
**Treatment by *baseline severity***	ICS High vs ICS+LABA	0.34 (-1.53 to 2.30)	-0.51 (-3.16 to 2.03)	-0.04 (-2.86 to 2.55)	-0.23 (-3.04 to 2.62)
	ICS Low vs ICS+LABA	-0.16 (-0.54 to 0.21)	0.22 (-0.22 to 0.65)	-0.66 (-2.10 to 0.36)	0.19 (-0.26 to 0.66)
	ICS Medium vs ICS+LABA	0.52 (-0.90 to 2.09)	-0.77 (-3.04 to 1.59)	0.48 (-1.54 to 2.76)	-1.17 (-4.01 to 1.43)
	ICS+LTRA vs ICS+LABA	not estimable	not estimable	not estimable	not estimable
	LTRA vs ICS+LABA	**-2.51 (-5.01 to -0.37)**	-1.90 (-5.53 to 1.14)	-2.89 (-6.37 to 0.26)	-1.92 (-5.57 to 1.06)
	placebo vs ICS+LABA	-0.49 (-1.18 to 0.22)	-0.69 (-1.88 to 0.41)	-0.85 (-2.84 to 0.86)	-0.61 (-1.82 to 0.52)

Bold indicates that zero is excluded from the credibility interval. The regression coefficient represents the change in the log odds ratio per unit increase in the covariate value.

Table S27. Model comparison assessments from network meta-analysis models including interactions for the outcome FEV_1

Interaction	Model	Number of trials (number of participants)	Number of data points	Residual deviance	Effective number of parameters (Pd)	Deviance information Criterion (DIC)	Between trial standard deviation
**Treatment by *age***	**Fixed-effect without interactions**	18 (1,657)	1659	1616.8	-2196	-579.2	-
	**Fixed-effect with interactions**	18 (1,657)	1659	1616.2	-2330.5	-714.3	-
	**Random-effects with interactions**	18 (1,657)	1659	1618.3	-2299.9	-681.6	0.05 (0.00, 0.14)
**Treatment by *sex***	**Random-effects without interactions**	20 (1,937)	1910	1864.3	-1193.8	670.6	0.04 (0.00, 0.12)
	**Fixed-effect with interactions**	20 (1,937)	1910	1866.9	-1105.4	761.5	-
	**Random-effects with interactions**	20 (1,937)	1910	1866.3	-1120	746.2	0.04 (0.00, 0.12)
**Treatment by *ethnicity***	**Random-effects without interactions**	19 (1,908)	1908	1865.7	-1205.8	659.8	0.04 (0.00, 0.12)
	**Fixed-effect with interactions**	19 (1,908)	1908	1864.6	-1002.8	861.7	-
	**Random-effects with interactions**	19 (1,908)	1908	1864.9	-1029.6	835.3	0.04 (0.00, 0.12)
**Treatment by *eczema***	**Fixed-effect without interactions**	5 (455)	455	441.1	199.8	640.9	-
	**Fixed-effect with interactions**	5 (455)	455	441.0	205.7	646.7	-
	**Random-effects with interactions**	5 (455)	455	441.9	203.3	645.1	0.08 (0.00, 0.22)
**Treatment by *eosinophilia***	**Fixed-effect without interactions**	11 (1,024)	1024	996.9	121.4	1118.3	-
	**Fixed-effect with interactions**	11 (1,024)	1024	996.2	128.6	1124.8	-
	**Random-effects with interactions**	11 (1,024)	1024	998.8	137.5	1136.3	0.07 (0.00, 0.21)

Table S28. Parameter estimates (Posterior mean [95% CrI]) from NMR models including interactions for the outcome FEV_1

Model		Fixed-effect NMA with interactions		Random-effects NMA with interactions	
		**Log odds ratio at the mean covariate value (95% CrI)**	**Regression coefficient for the treatment by covariate interaction (95% CrI)**	**Log odds ratio at the mean covariate value (95% CrI)**	**Regression coefficient for the treatment by covariate interaction (95% CrI)**
**Treatment by *age***	ICS High vs ICS+LABA	-0.04 (-0.15 to 0.06)	0.02 (0.00 to 0.04)	-0.03 (-0.16 to 0.12)	0.02 (0.00 to 0.04)
	ICS Low vs ICS+LABA	-0.02 (-0.07 to 0.02)	0.00 (-0.02 to 0.01)	-0.02 (-0.09 to 0.06)	0.00 (-0.02 to 0.01)
	ICS Medium vs ICS+LABA	-0.02 (-0.07 to 0.02)	-0.01 (-0.03 to 0.00)	-0.03 (-0.13 to 0.06)	-0.01 (-0.03 to 0.01)
	ICS unknown dose vs ICS+LABA	-0.28 (-5.25 to 4.40)	-0.05 (-8.85 to 8.35)	-0.29 (-3.27 to 2.69)	-0.06 (-5.41 to 5.09)
	ICS+LTRA vs ICS+LABA	**-0.10 (-0.18 to -0.01)**	0.01 (0.00 to 0.03)	-0.10 (-0.24 to 0.05)	0.01 (-0.01 to 0.03)
	LTRA vs ICS+LABA	0.14 (-0.11 to 0.39)	0.04 (-0.05 to 0.13)	0.16 (-0.12 to 0.43)	0.04 (-0.05 to 0.13)
	placebo vs ICS+LABA	**-0.13 (-0.21 to -0.05)**	-0.02 (-0.04 to 0.01)	-0.13 (-0.27 to 0.00)	-0.02 (-0.05 to 0.01)
**Treatment by *sex***	ICS High vs ICS+LABA	0.02 (-0.08 to 0.12)	-0.02 (-0.15 to 0.12)	0.02 (-0.10 to 0.16)	-0.01 (-0.15 to 0.12)
	ICS Low vs ICS+LABA	-0.02 (-0.07 to 0.03)	0.00 (-0.07 to 0.06)	-0.02 (-0.08 to 0.05)	0.00 (-0.06 to 0.07)
	ICS Medium vs ICS+LABA	-0.01 (-0.05 to 0.02)	0.02 (-0.05 to 0.09)	-0.02 (-0.10 to 0.04)	0.02 (-0.05 to 0.09)
	ICS unknown dose vs ICS+LABA	-0.37 (-2.74 to 2.04)	-0.14 (-9.96 to 9.57)	-0.32 (-2.79 to 1.99)	0.12 (-9.26 to 9.60)
	ICS+LTRA vs ICS+LABA	**-0.20 (-0.32 to -0.08)**	-0.08 (-0.33 to 0.16)	**-0.20 (-0.37 to -0.05)**	-0.09 (-0.33 to 0.16)
	LTRA vs ICS+LABA	0.22 (-0.01 to 0.44)	**0.67 (0.23 to 1.11)**	0.23 (-0.01 to 0.48)	**0.68 (0.21 to 1.14)**
	placebo vs ICS+LABA	**-0.12 (-0.21 to -0.03)**	0.04 (-0.11 to 0.18)	**-0.13 (-0.26 to -0.02)**	0.04 (-0.09 to 0.17)
**Treatment by *ethnicity***	ICS High vs ICS+LABA	0.05 (-0.10 to 0.20)	-0.10 (-0.56 to 0.34)	0.05 (-0.11 to 0.22)	-0.08 (-0.52 to 0.36)
	ICS Low vs ICS+LABA	-0.02 (-0.07 to 0.02)	-0.05 (-0.12 to 0.03)	-0.02 (-0.09 to 0.05)	-0.04 (-0.12 to 0.04)
	ICS Medium vs ICS+LABA	0.02 (-0.03 to 0.08)	-0.16 (-0.32 to 0.00)	0.01 (-0.08 to 0.09)	-0.16 (-0.32 to 0.00)
	ICS+LTRA vs ICS+LABA	**-0.18 (-0.30 to -0.07)**	-0.08 (-0.23 to 0.06)	**-0.18 (-0.34 to -0.03)**	-0.07 (-0.21 to 0.07)
	LTRA vs ICS+LABA	0.12 (-0.16 to 0.39)	0.23 (-0.32 to 0.77)	0.13 (-0.15 to 0.40)	0.23 (-0.32 to 0.77)
	placebo vs ICS+LABA	**-0.11 (-0.20 to -0.02)**	0.03 (-0.12 to 0.18)	**-0.13 (-0.27 to -0.01)**	0.04 (-0.11 to 0.19)
**Treatment by *eczema***	ICS High vs ICS Medium	0.14 (-0.15 to 0.44)	-0.01 (-0.37 to 0.35)	0.12 (-0.24 to 0.46)	0.00 (-0.37 to 0.35)
	ICS Low vs ICS Medium	0.08 (-0.14 to 0.28)	-0.03 (-0.27 to 0.21)	0.05 (-0.25 to 0.30)	-0.03 (-0.27 to 0.20)
	ICS+LABA vs ICS Medium	0.00 (-0.04 to 0.05)	0.03 (-0.10 to 0.15)	-0.01 (-0.17 to 0.13)	0.04 (-0.10 to 0.17)
	ICS+LTRA vs ICS Medium	**-0.18 (-0.32 to -0.05)**	-0.03 (-0.20 to 0.13)	-0.19 (-0.42 to 0.04)	-0.02 (-0.19 to 0.14)
	LTRA vs ICS Medium	0.24 (-0.11 to 0.59)	0.12 (-0.40 to 0.63)	0.22 (-0.22 to 0.62)	0.12 (-0.40 to 0.63)
	placebo vs ICS Medium	-0.30 (-0.78 to 0.19)	-0.51 (-1.20 to 0.17)	-0.30 (-0.80 to 0.19)	-0.49 (-1.14 to 0.19)
**Treatment by *eosinophilia***	ICS High vs ICS Low	0.16 (-0.08 to 0.39)	-0.14 (-0.45 to 0.18)	0.15 (-0.14 to 0.42)	-0.14 (-0.44 to 0.17)
	ICS Medium vs ICS Low	0.03 (-0.12 to 0.19)	-0.08 (-0.34 to 0.16)	0.03 (-0.17 to 0.22)	-0.08 (-0.34 to 0.15)
	ICS+LABA vs ICS Low	0.01 (-0.05 to 0.06)	**0.11 (0.03 to 0.19)**	0.00 (-0.12 to 0.10)	**0.10 (0.03 to 0.18)**
	ICS+LTRA vs ICS Low	**-0.15 (-0.28 to -0.01)**	-0.05 (-0.22 to 0.11)	-0.15 (-0.39 to 0.08)	-0.05 (-0.22 to 0.11)
	LTRA vs ICS Low	0.04 (-0.29 to 0.36)	0.26 (-0.32 to 0.81)	0.05 (-0.30 to 0.42)	0.25 (-0.29 to 0.79)
	placebo vs ICS Low	**-0.09 (-0.17 to -0.01)**	-0.03 (-0.18 to 0.13)	-0.11 (-0.28 to 0.01)	-0.03 (-0.18 to 0.12)

Bold indicates that zero is excluded from the credibility interval. The regression coefficient represents the change in the mean difference per unit increase in the covariate value.

Table S29. Mean difference (95% CrI) from random- effects NMR with "treatment by sex" interactions for the outcome FEV_1

	TRT 1 \ TRT 2	ICS Medium	ICS High	ICS+LABA	ICS unknown dose	ICS+LTRA	LTRA	Placebo*
**Females (N = 701)**	**ICS Low N = 195**	-0.01 (-0.11 to 0.11)	-0.03 (-0.20 to 0.13)	-0.02 (-0.09 to 0.06)	0.23 (-7.91 to 8.50)	0.24 (-0.03 to 0.53)	**-0.68 (-1.10 to -0.27)**	0.09 (-0.04 to 0.24)
	**ICS Medium N = 111**		-0.02 (-0.21 to 0.14)	-0.01 (-0.10 to 0.07)	0.24 (-7.87 to 8.53)	0.25 (-0.02 to 0.52)	**-0.67 (-1.10 to -0.24)**	0.10 (-0.05 to 0.26)
	**ICS High N = 45**			0.02 (-0.14 to 0.18)	0.26 (-7.85 to 8.57)	0.28 (-0.03 to 0.59)	**-0.65 (-1.10 to -0.21)**	0.12 (-0.09 to 0.35)
	**ICS+LABA N = 290**				0.25 (-7.87 to 8.55)	0.26 (-0.02 to 0.52)	**-0.66 (-1.09 to -0.24)**	0.11 (-0.03 to 0.26)
	**ICS unknown dose N = 2**					0.01 (-8.22 to 8.13)	-0.91 (-9.09 to 7.35)	-0.14 (-8.40 to 7.99)
	**ICS+LTRA N = 6**						**-0.92 (-1.41 to -0.43)**	-0.15 (-0.45 to 0.16)
	**LTRA N = 3**							**0.77 (0.33 to 1.22)**
**Males (N = 1237)**	**ICS Low N = 311**	0.01 (-0.08 to 0.12)	-0.05 (-0.19 to 0.10)	-0.02 (-0.09 to 0.06)	0.35 (-1.19 to 1.94)	0.16 (0.00 to 0.32)	0.00 (-0.25 to 0.24)	**0.13 (0.02 to 0.27)**
	**ICS Medium N = 213**		-0.06 (-0.22 to 0.08)	-0.03 (-0.11 to 0.04)	0.33 (-1.21 to 1.93)	0.14 (-0.01 to 0.29)	-0.01 (-0.28 to 0.24)	0.12 (-0.02 to 0.27)
	**ICS High N = 102**			0.03 (-0.10 to 0.17)	0.39 (-1.16 to 1.98)	**0.20 (0.01 to 0.41)**	0.05 (-0.23 to 0.33)	**0.18 (0.01 to 0.37)**
	**ICS+LABA N = 499**				0.36 (-1.17 to 1.96)	**0.17 (0.03 to 0.32)**	0.02 (-0.24 to 0.26)	**0.15 (0.03 to 0.29)**
	**ICS unknown dose N = 13**					-0.19 (-1.79 to 1.33)	-0.35 (-1.96 to 1.20)	-0.21 (-1.81 to 1.31)
	**ICS+LTRA N = 23**						-0.15 (-0.45 to 0.13)	-0.02 (-0.20 to 0.17)
	**LTRA N = 11**							0.13 (-0.14 to 0.41)

The table compares the effect estimate for an intervention in the row with an intervention in a column (TRT 1 vs. TRT 2).
MD > 0 favours TRT 1 (all data included, IPD and AgD where possible); 95% CrIs that exclude zero are highlighted in bold; N = number of participants; TRT = treatment; ICS = inhaled corticosteroids; LABA = long-acting beta2-agonists; LTRA = leukotriene receptor antagonists; *Placebo (females), N = 49; (males), N=65.

Table S30. Mean difference (95% CrI) from fixed effect NMR with "treatment by eosinophilia" interactions for the outcome FEV_1

	TRT 1 \ TRT 2	ICS Medium	ICS High	ICS+LABA	ICS+LTRA	LTRA	Placebo*
Eosinophilic (N = 419)	**ICS Low<br>N = 178**	0.02<br>(-0.19 to 0.23)	-0.08<br>(-0.33 to 0.17)	-0.07<br>(-0.14 to 0.00)	**0.18<br>(0.02 to 0.34)**	-0.19<br>(-0.50 to 0.13)	0.10<br>(-0.03 to 0.23)
		**ICS Medium<br>N = 11**	-0.10<br>(-0.40 to 0.20)	-0.08<br>(-0.29 to 0.12)	0.16<br>(-0.06 to 0.39)	-0.20<br>(-0.58 to 0.17)	0.09<br>(-0.15 to 0.33)
			**ICS High<br>N = 21**	0.01<br>(-0.24 to 0.27)	0.26<br>(-0.02 to 0.55)	-0.11<br>(-0.50 to 0.30)	0.19<br>(-0.09 to 0.45)
				**ICS+LABA<br>N = 161**	**0.25<br>(0.09 to 0.40)**	-0.12<br>(-0.44 to 0.20)	**0.17<br>(0.03 to 0.31)**
					**ICS+LTRA<br>N = 7**	**-0.37<br>(-0.72 to -0.02)**	-0.07<br>(-0.27 to 0.12)
						**LTRA<br>N = 10**	0.29<br>(-0.05 to 0.63)
Non-eosinophilic (N = 605)	**ICS Low<br>N = 270**	-0.06<br>(-0.25 to 0.12)	-0.22<br>(-0.52 to 0.09)	0.04<br>(-0.02 to 0.10)	0.13<br>(-0.03 to 0.29)	0.07<br>(-0.43 to 0.57)	0.08<br>(-0.01 to 0.16)
		**ICS Medium<br>N = 18**	-0.16<br>(-0.49 to 0.18)	0.10<br>(-0.08 to 0.29)	0.19<br>(0.00 to 0.39)	0.13<br>(-0.39 to 0.65)	0.14<br>(-0.06 to 0.34)
			**ICS High<br>N = 15**	0.26<br>(-0.05 to 0.56)	**0.35<br>(0.02 to 0.67)**	0.29<br>(-0.29 to 0.87)	0.29<br>(-0.02 to 0.60)
				**ICS+LABA<br>N = 215**	0.09<br>(-0.07 to 0.24)	0.03<br>(-0.46 to 0.52)	0.04<br>(-0.06 to 0.14)
					**ICS+LTRA<br>N = 7**	-0.06<br>(-0.57 to 0.45)	-0.05<br>(-0.23 to 0.12)
						**LTRA<br>N = 4**	0.01<br>(-0.49 to 0.50)

The table compares the effect estimate for an intervention in the row with an intervention in a column (TRT 1 vs. TRT 2).

MD > 0 favours TRT 1 (all data included, only IPD). The estimates not including 0 are in bold. N = number of participants; TRT = treatment; ICS = inhaled corticosteroids; LABA = long-acting $beta_2$-agonists; LTRA = leukotriene receptor antagonists; *Placebo (Eosinophilic), N = 31; (Non-Eosinophilic), N=76.

Figure S1. Secondary flowchart

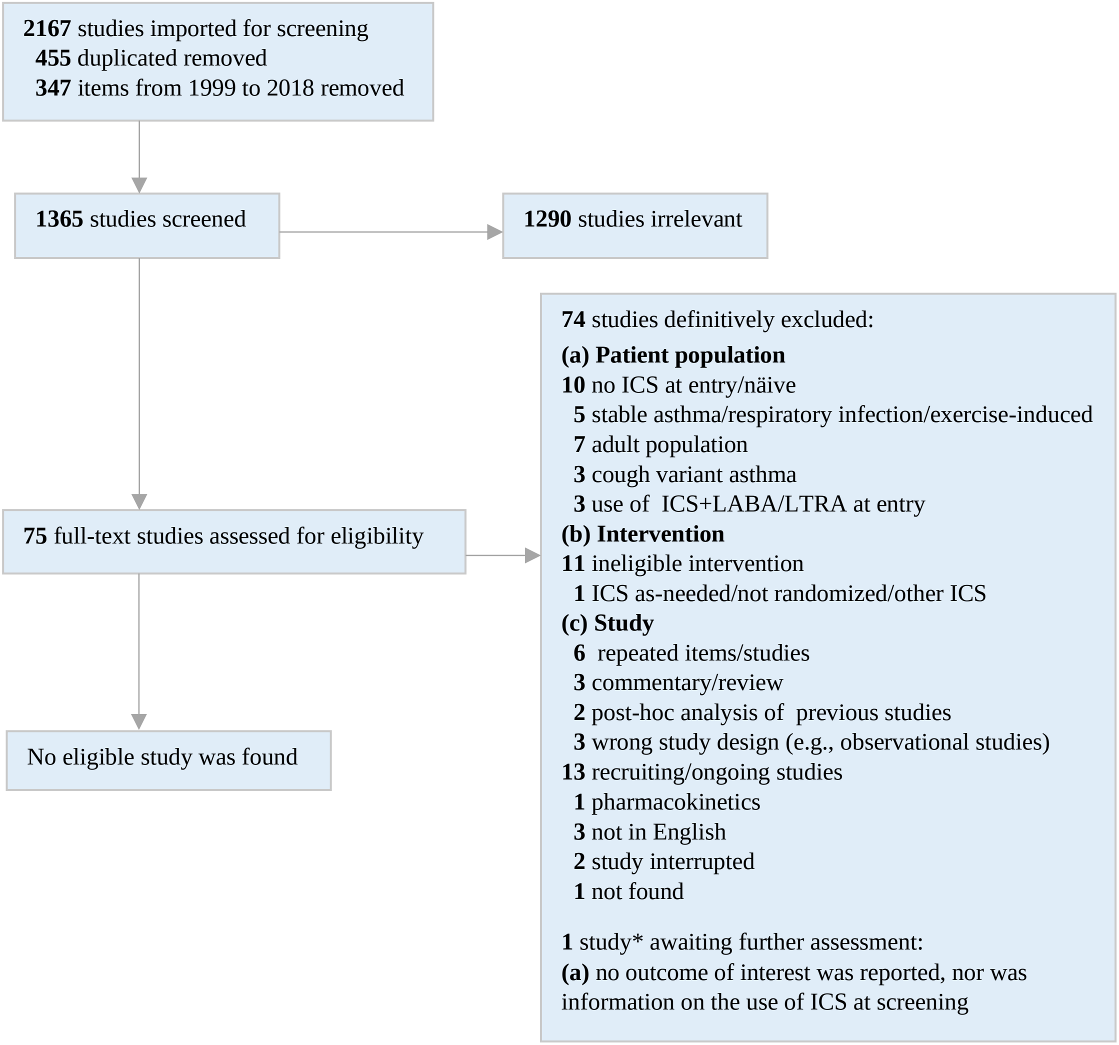

Study search from 10 September 2019 to 5 May 2023 (used to assess the impact on results of any missing studies**).**
*This study does not report any outcome of interest for the network meta-analysis and whether children were using ICS alone at screening.
ICS: inhaled corticosteroid; LABA: Long-Acting β_2-Agonist; LTRA: Leukotriene Receptor Antagonist; IPD: individual participant data; FEV_1: forced expiratory volume in 1 second.

Figure S2A. Comparison-adjusted funnel plots (exacerbation frequentist random-effects network meta-analysis)

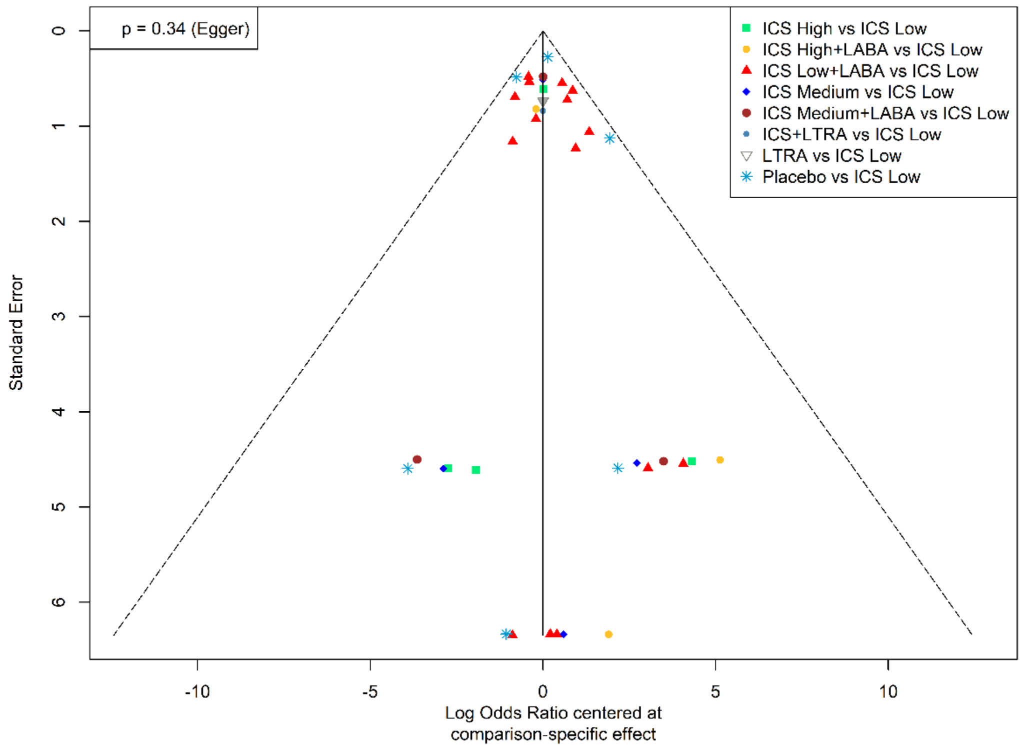

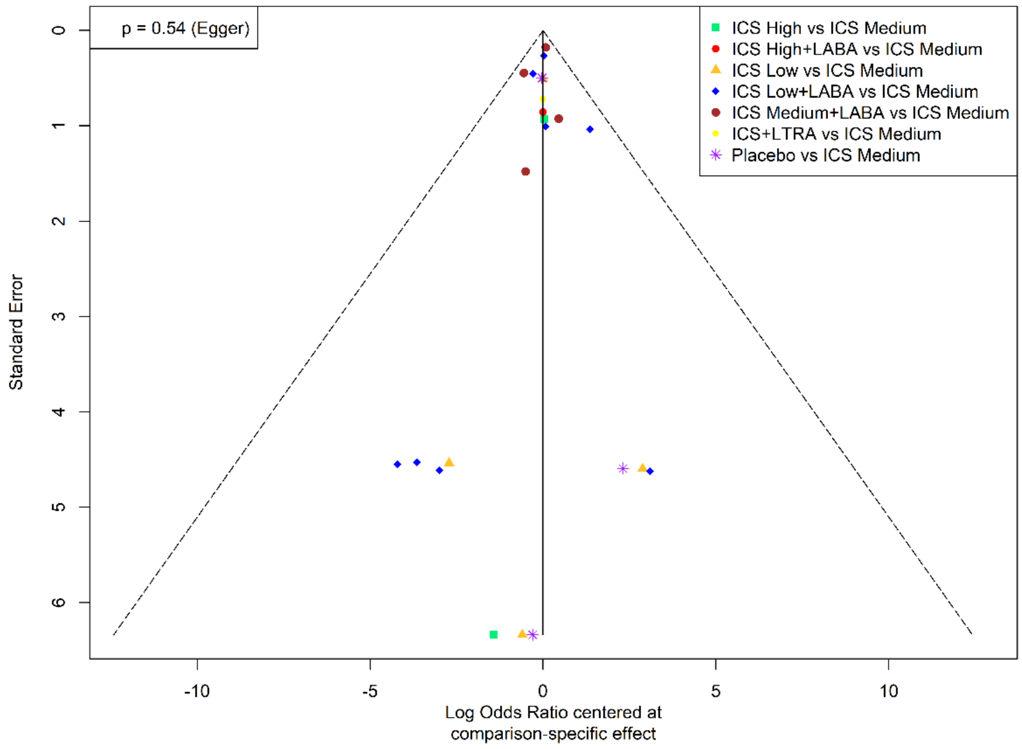

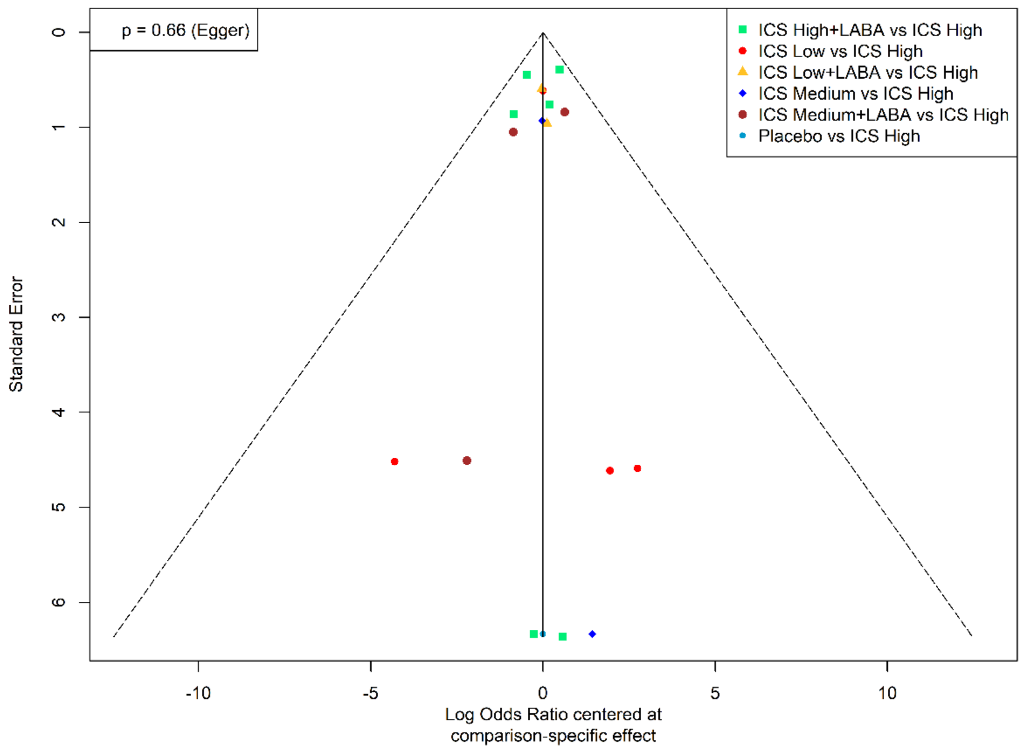
p = 0.66 (Egger)
ICS High+LABA vs ICS High
ICS Low vs ICS High
ICS Low+LABA vs ICS High
ICS Medium vs ICS High
ICS Medium+LABA vs ICS High
Placebo vs ICS High
Standard Error
0
1
2
3
4
5
6
-10
-5
0
5
10
Log Odds Ratio centered at
comparison-specific effect

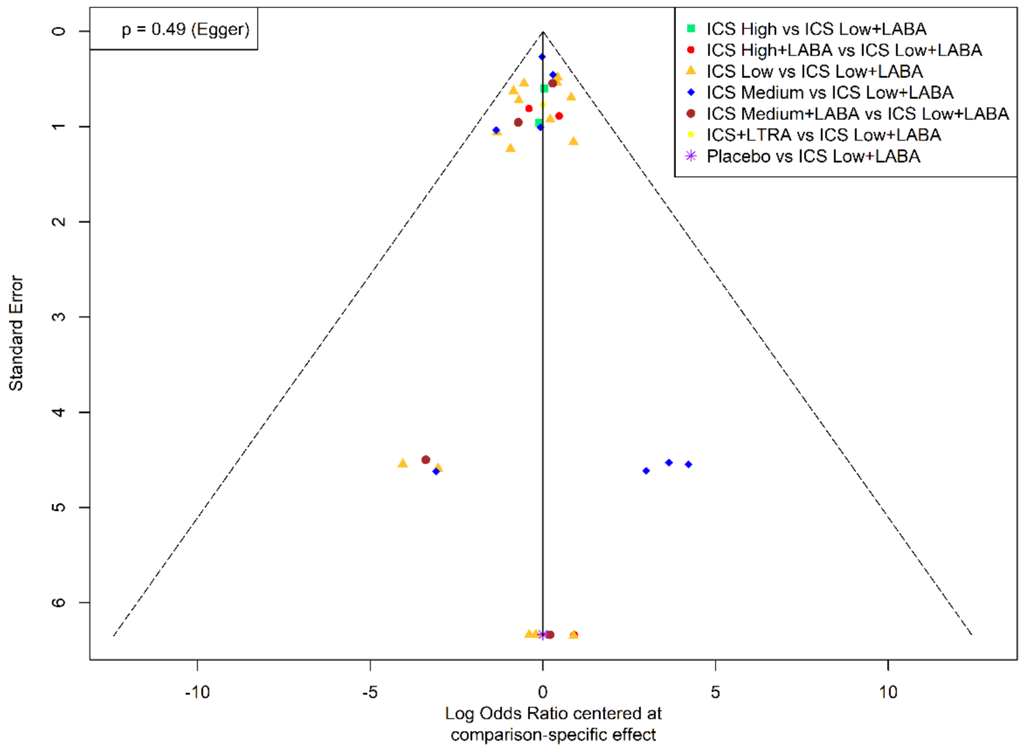
p = 0.49 (Egger)
ICS High vs ICS Low+LABA
ICS High+LABA vs ICS Low+LABA
ICS Low vs ICS Low+LABA
ICS Medium vs ICS Low+LABA
ICS Medium+LABA vs ICS Low+LABA
ICS+LTRA vs ICS Low+LABA
Placebo vs ICS Low+LABA
Standard Error
0
1
2
3
4
5
6
-10
-5
0
5
10
Log Odds Ratio centered at
comparison-specific effect

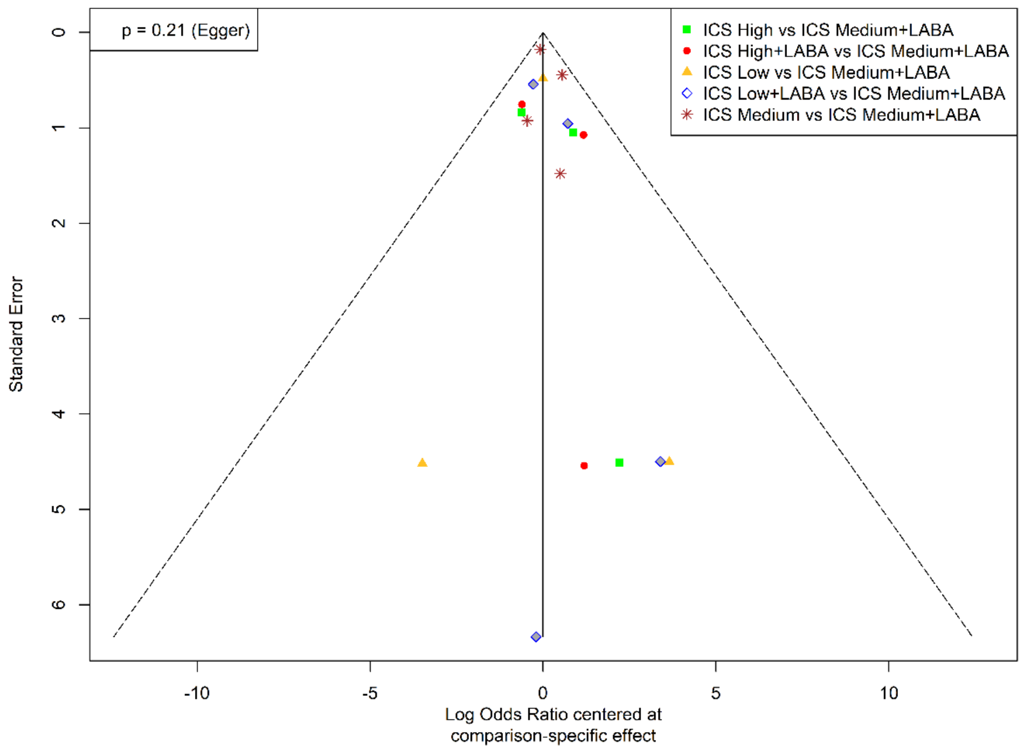
p = 0.21 (Egger)
ICS High vs ICS Medium+LABA
ICS High+LABA vs ICS Medium+LABA
ICS Low vs ICS Medium+LABA
ICS Low+LABA vs ICS Medium+LABA
ICS Medium vs ICS Medium+LABA
Standard Error
0
1
2
3
4
5
6
-10
-5
0
5
10
Log Odds Ratio centered at comparison-specific effect

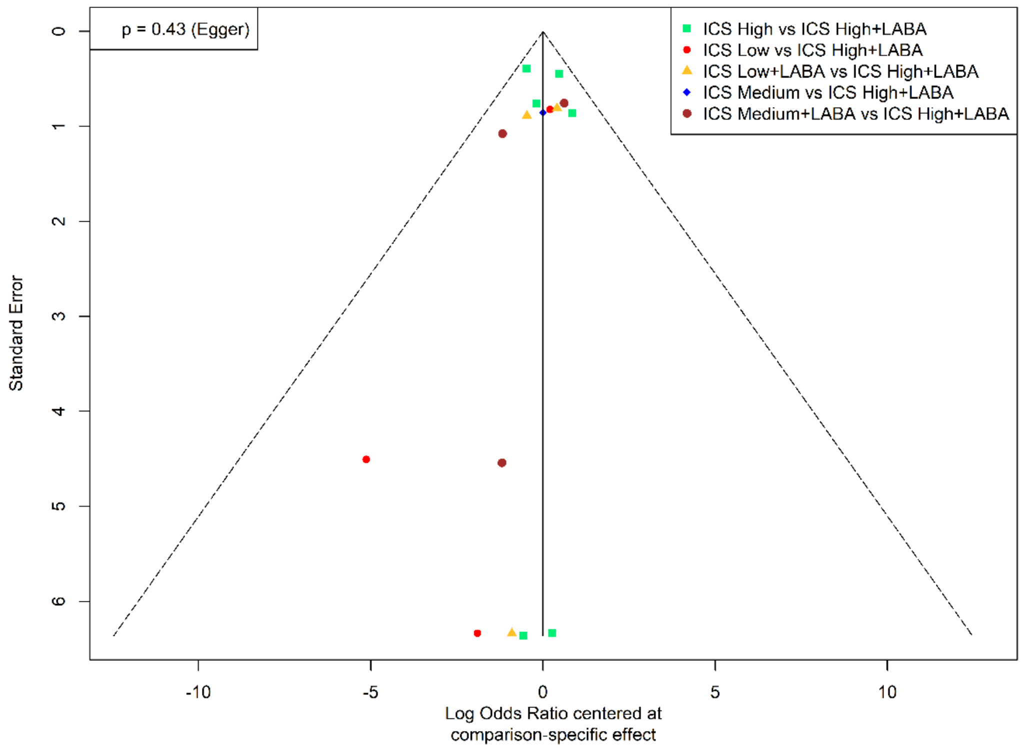
p = 0.43 (Egger)
ICS High vs ICS High+LABA
ICS Low vs ICS High+LABA
ICS Low+LABA vs ICS High+LABA
ICS Medium vs ICS High+LABA
ICS Medium+LABA vs ICS High+LABA
Standard Error
0
1
2
3
4
5
6
-10
-5
0
5
10
Log Odds Ratio centered at comparison-specific effect

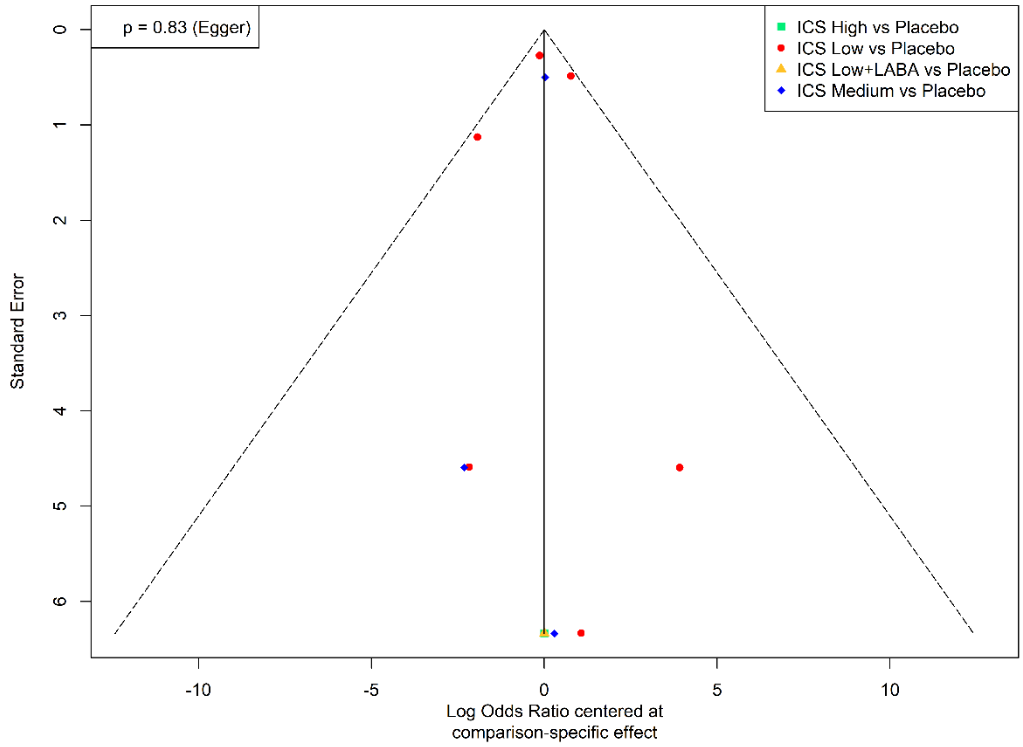

The comparison-adjusted funnel plots appear symmetric, implying the absence of small-study effects in the network. The Egger's test did not show publication bias at the confidence level of 0.05.

There are insufficient direct comparisons to carry out Egger's test for ICS+LTRA, LTRA, and ICS+Theophylline.

Figure S2B. Comparison-adjusted funnel plots (asthma control frequentist fixed effect network meta-analysis)

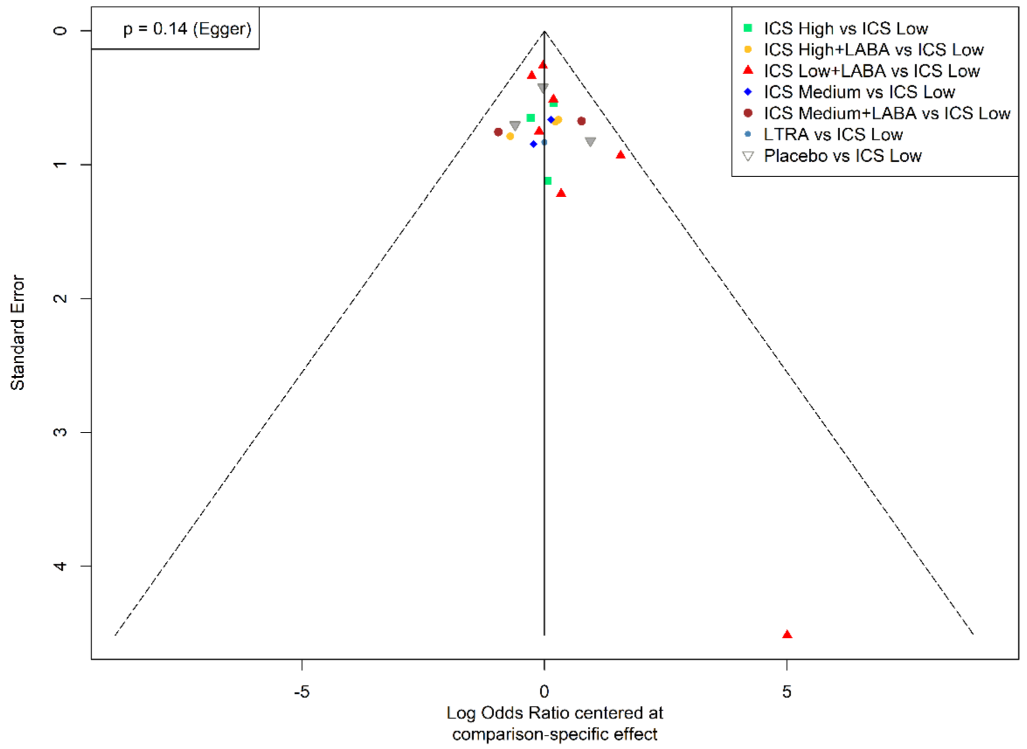

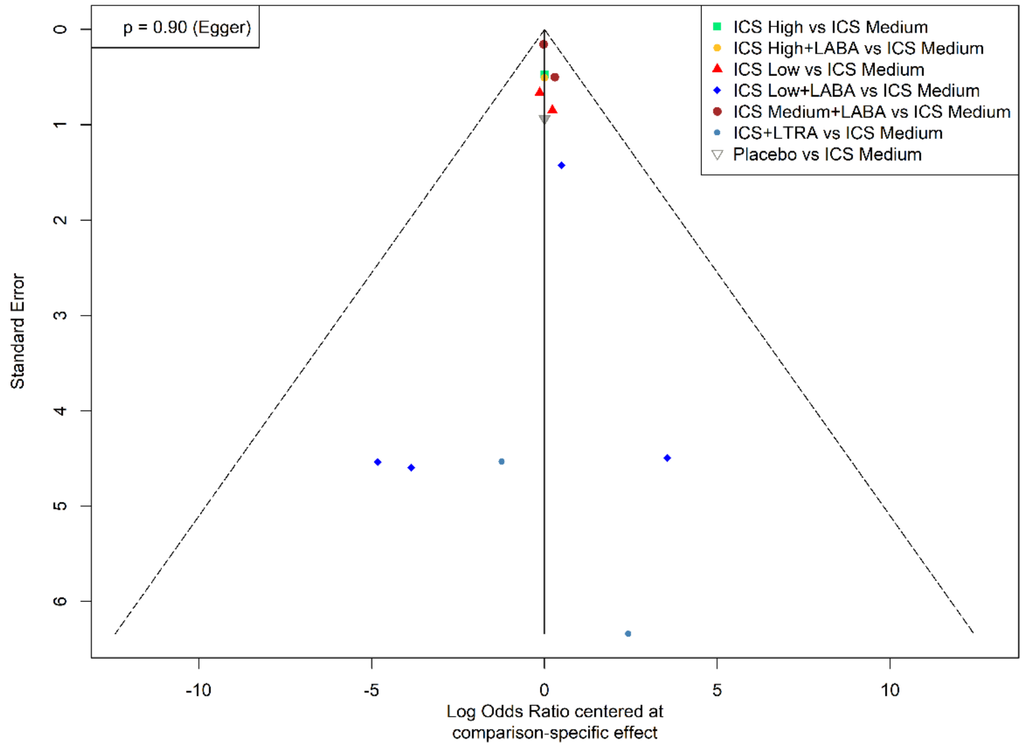

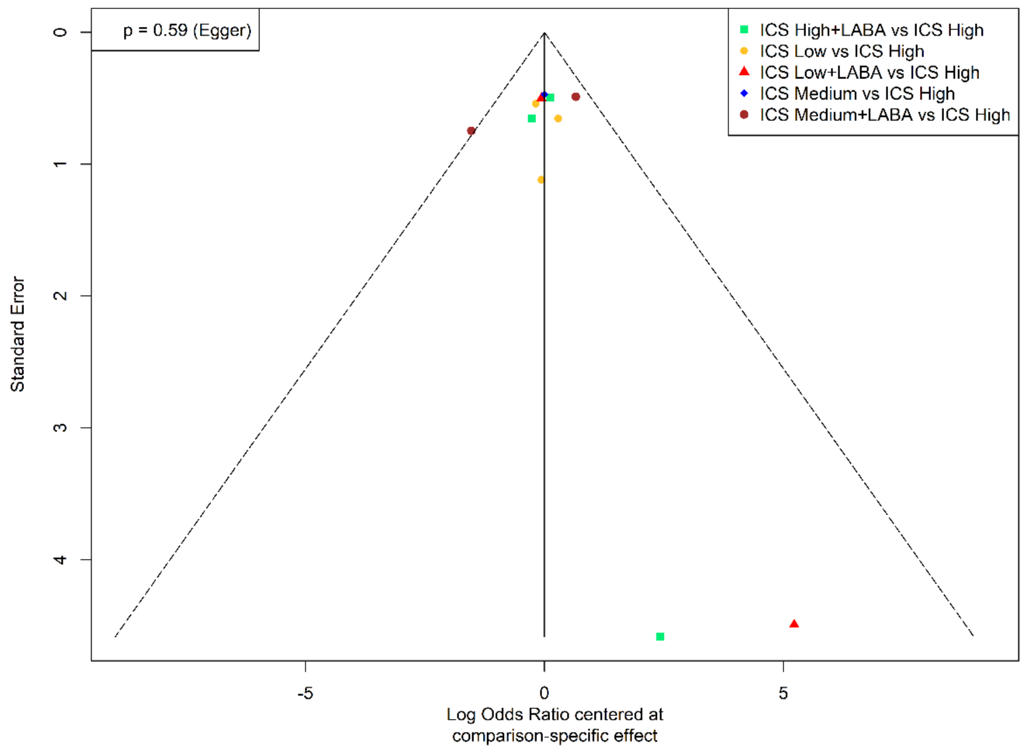
p = 0.59 (Egger)
ICS High+LABA vs ICS High
ICS Low vs ICS High
ICS Low+LABA vs ICS High
ICS Medium vs ICS High
ICS Medium+LABA vs ICS High
Standard Error
0
1
2
3
4
-5
0
5
Log Odds Ratio centered at
comparison-specific effect

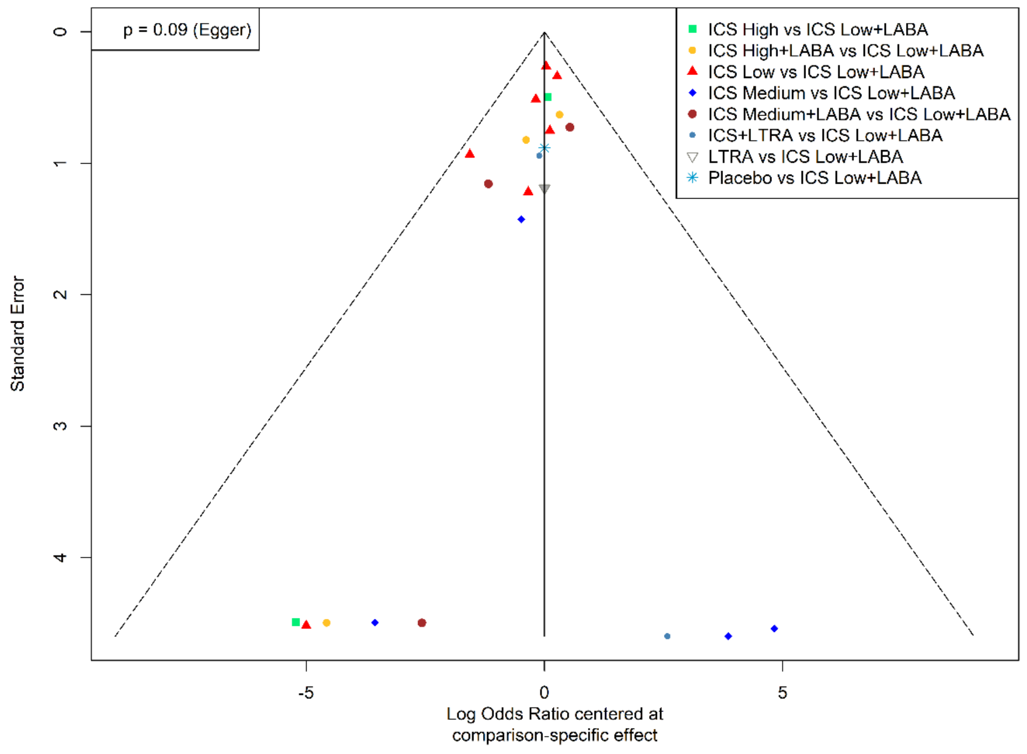
p = 0.09 (Egger)
ICS High vs ICS Low+LABA
ICS High+LABA vs ICS Low+LABA
ICS Low vs ICS Low+LABA
ICS Medium vs ICS Low+LABA
ICS Medium+LABA vs ICS Low+LABA
ICS+LTRA vs ICS Low+LABA
LTRA vs ICS Low+LABA
Placebo vs ICS Low+LABA
Standard Error
0
1
2
3
4
-5
0
5
Log Odds Ratio centered at
comparison-specific effect

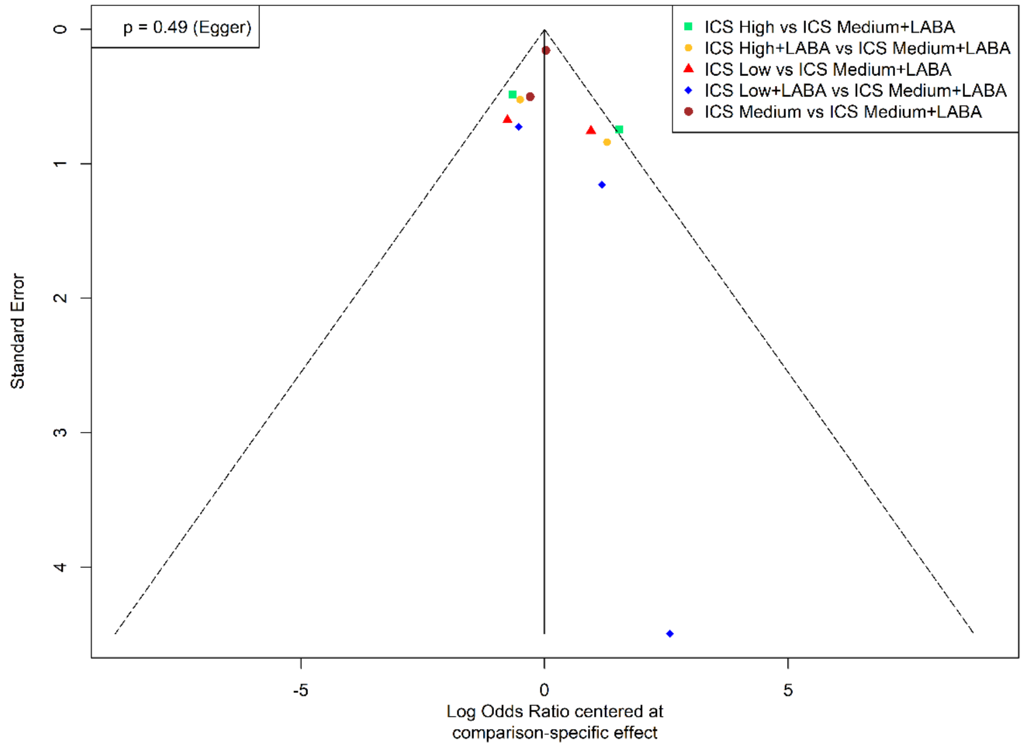

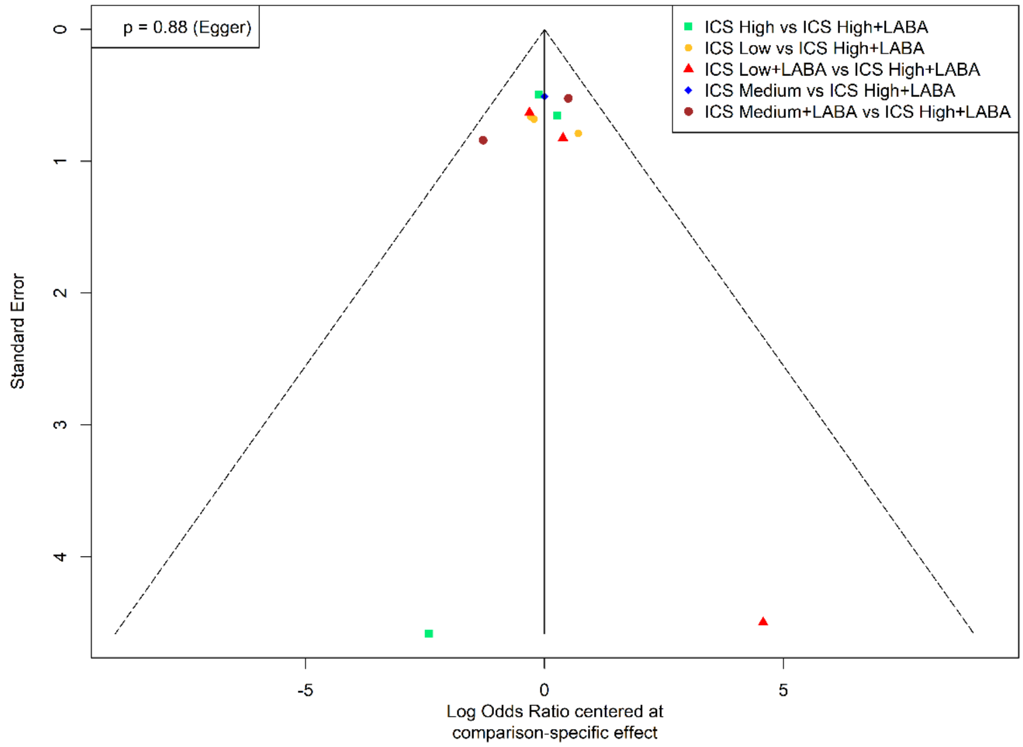

The comparison-adjusted funnel plots appear symmetric, implying the absence of small-study effects in the network. The Egger's test did not show publication bias at the confidence level of 0.05.

There are insufficient direct comparisons to carry out Egger's test for ICS+LTRA, LTRA, and placebo.

Figure S2C. Comparison-adjusted funnel plots (FEV$_1$ frequentist fixed effect network meta-analysis)

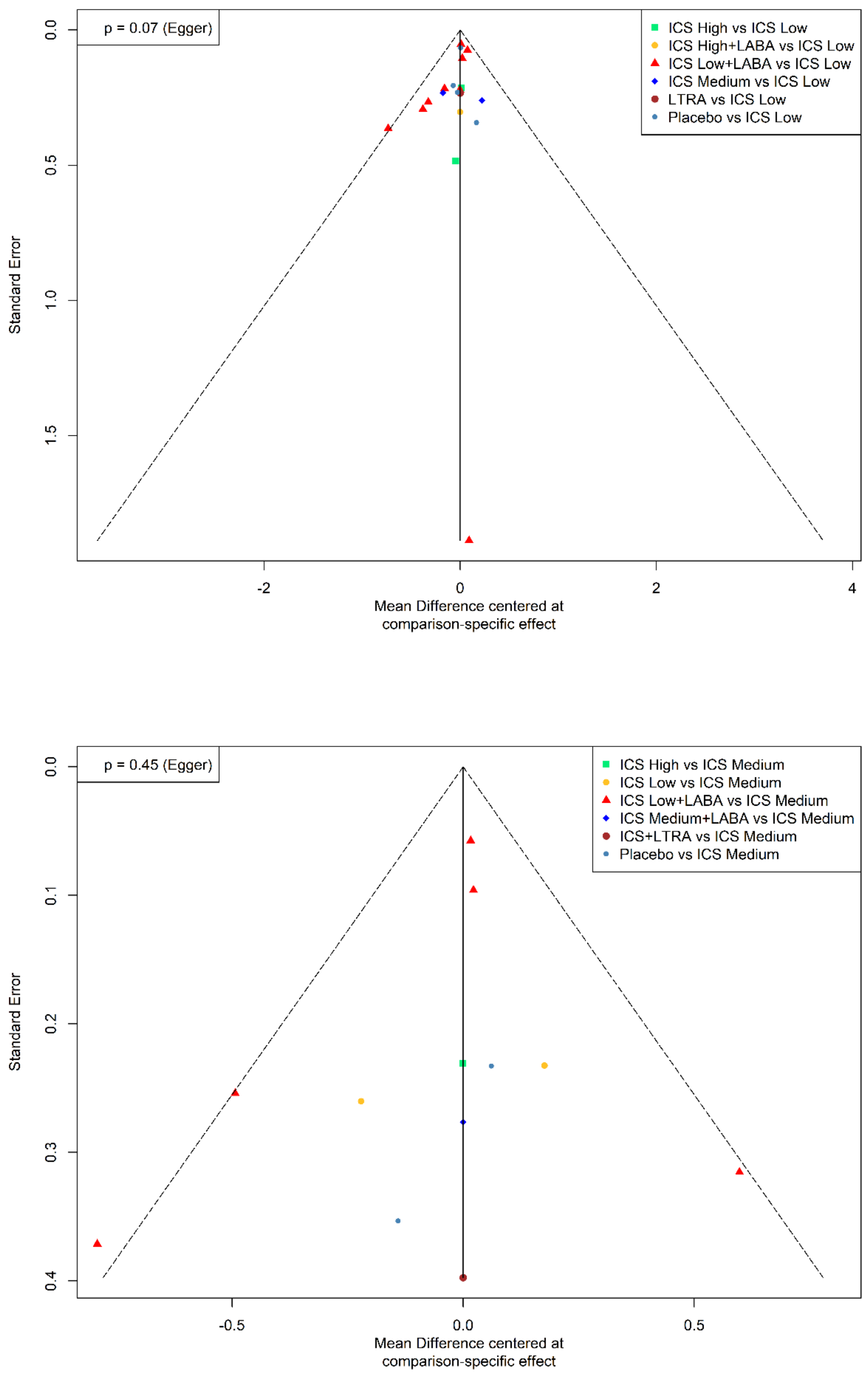

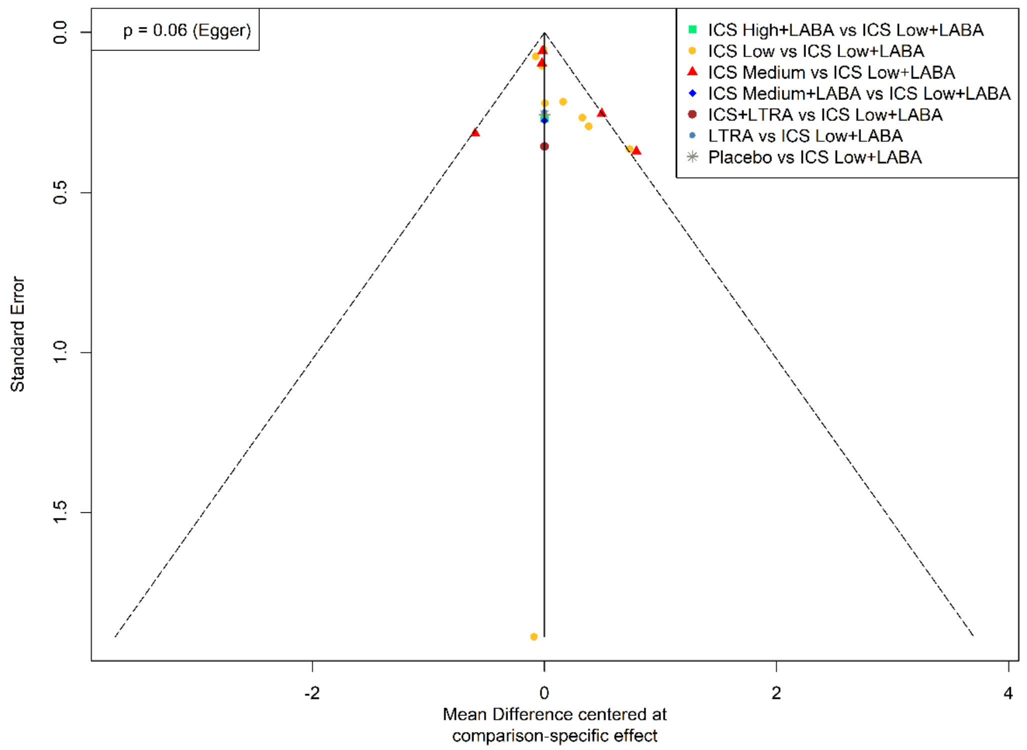

The comparison-adjusted funnel plots appear symmetric, implying the absence of small-study effects in the network. The Egger's test did not show publication bias at the confidence level of 0.05.

There are insufficient direct comparisons to carry out Egger's test for ICS High, ICS Medium+LABA, ICS High+LABA, ICS+LTRA, LTRA, ICS unknown dose, and placebo.

Figure S3. Rankings for the random-effects network meta-analysis (ICS stratified by dose when combined with LABA) for exacerbations – Analysis A1

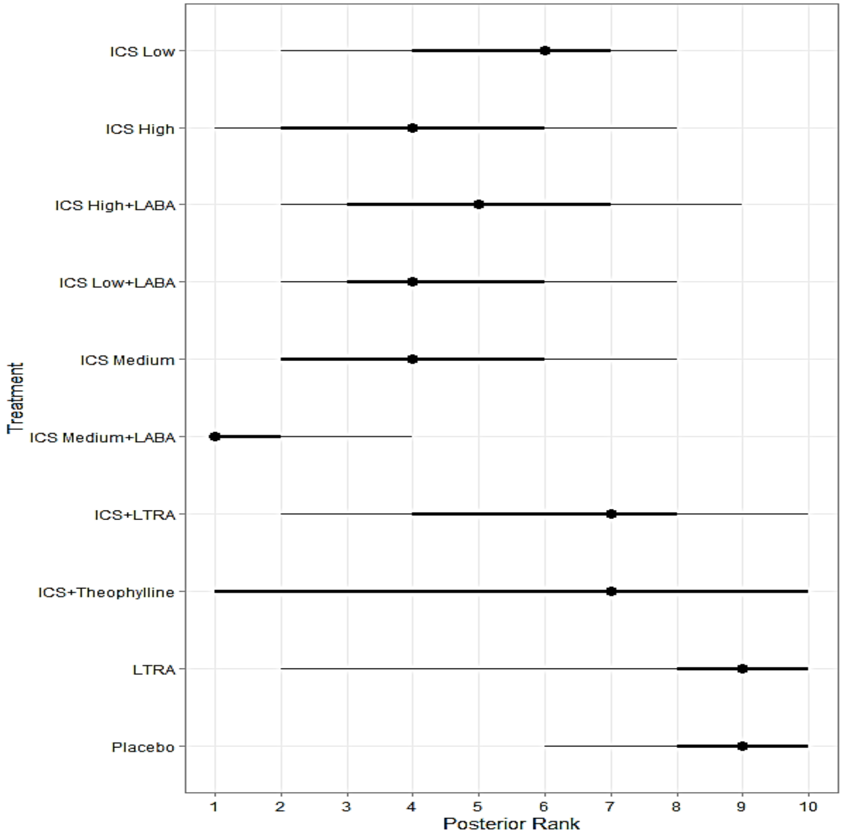

A, Posterior treatment rankings from fitted NMA model. Rank median (point), IQR (bold line), 95% interval (thin line). Lower rank is better.

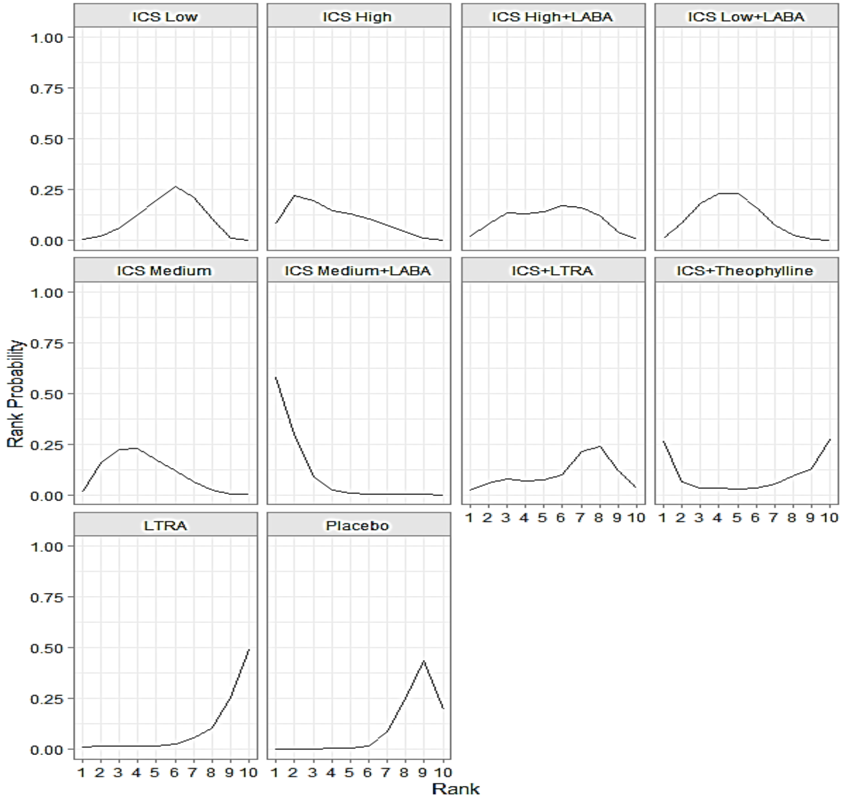

B, Rank probability plots from fitted NMA model.

Figure S4 (parts 1 to 3)**. Exacerbation frequentist random-effects network meta-analysis (OR, 95% Cr) with IPD and AgD (Analysis A1: 40 trials, 8168 participants, 649 events)**

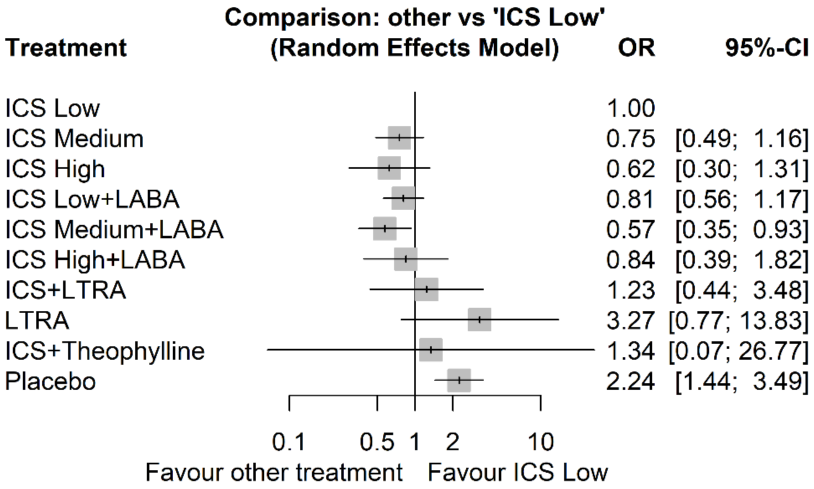

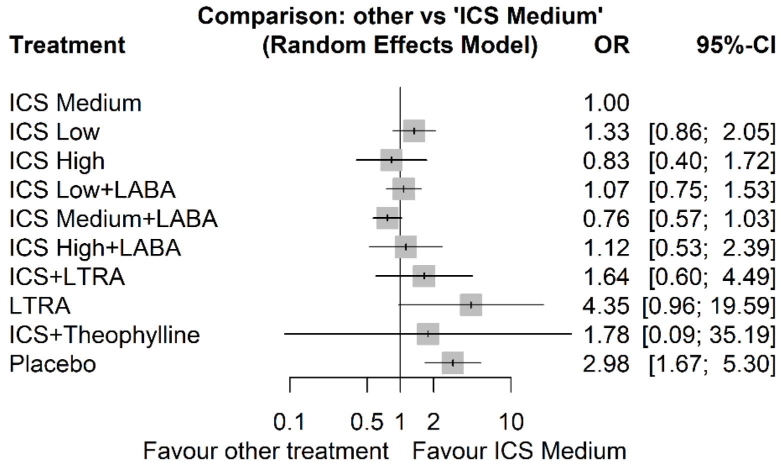

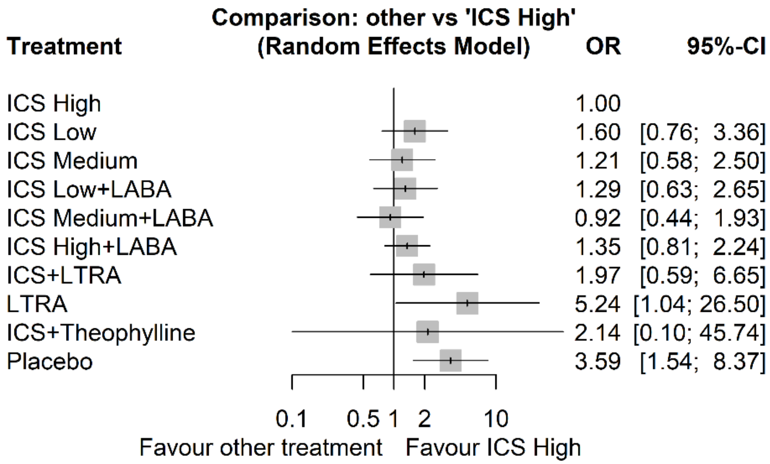

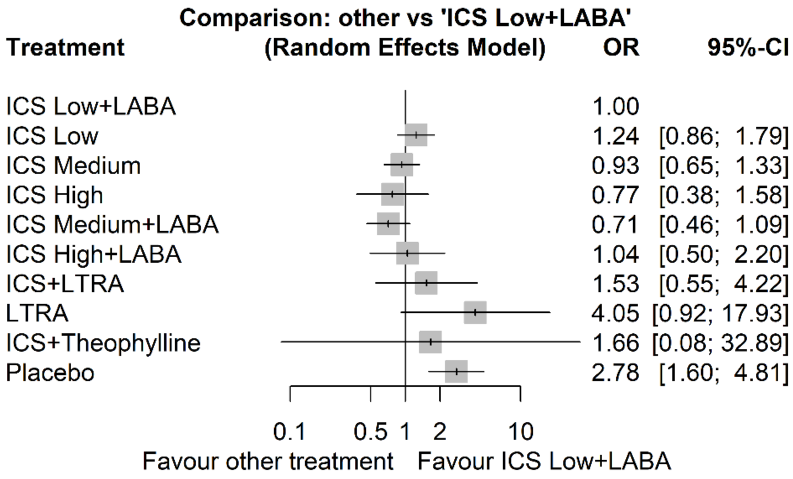

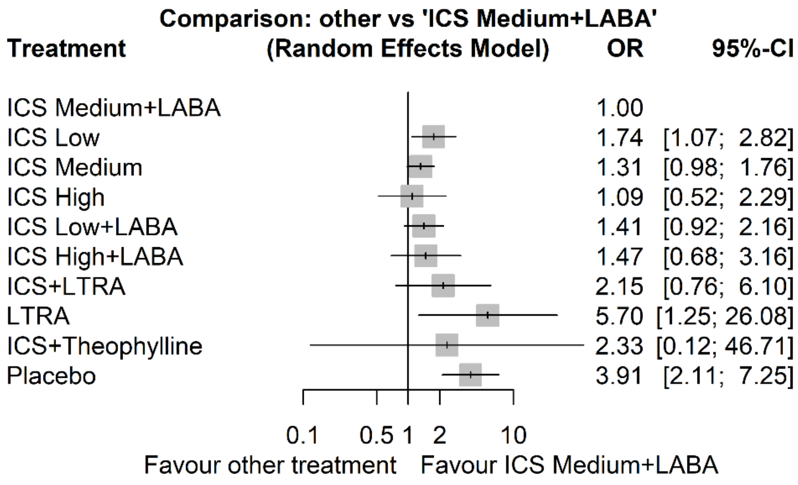
Comparison: other vs 'ICS Medium+LABA'
Treatment
(Random Effects Model)
OR
95%-CI
ICS Medium+LABA 1.00
ICS Low 1.74 [1.07; 2.82]
ICS Medium 1.31 [0.98; 1.76]
ICS High 1.09 [0.52; 2.29]
ICS Low+LABA 1.41 [0.92; 2.16]
ICS High+LABA 1.47 [0.68; 3.16]
ICS+LTRA 2.15 [0.76; 6.10]
LTRA 5.70 [1.25; 26.08]
ICS+Theophylline 2.33 [0.12; 46.71]
Placebo 3.91 [2.11; 7.25]
0.1 0.5 1 2 10
Favour other treatment
Favour ICS Medium+LABA

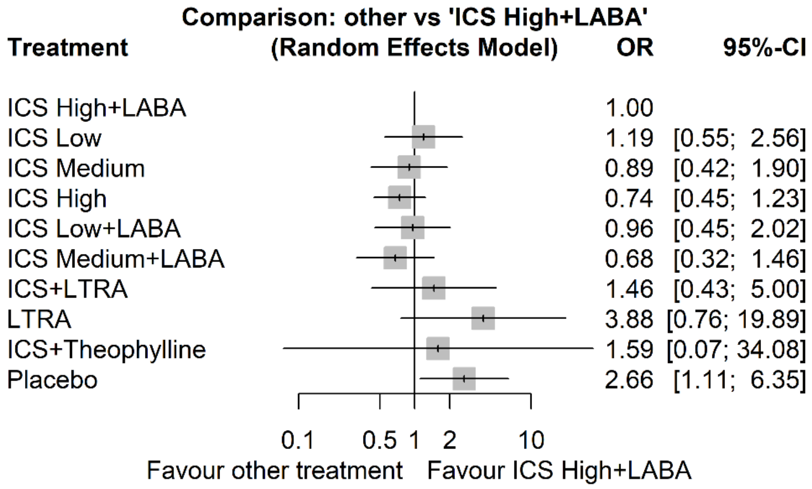
Comparison: other vs 'ICS High+LABA'
Treatment
(Random Effects Model)
OR
95%-CI
ICS High+LABA 1.00
ICS Low 1.19 [0.55; 2.56]
ICS Medium 0.89 [0.42; 1.90]
ICS High 0.74 [0.45; 1.23]
ICS Low+LABA 0.96 [0.45; 2.02]
ICS Medium+LABA 0.68 [0.32; 1.46]
ICS+LTRA 1.46 [0.43; 5.00]
LTRA 3.88 [0.76; 19.89]
ICS+Theophylline 1.59 [0.07; 34.08]
Placebo 2.66 [1.11; 6.35]
0.1 0.5 1 2 10
Favour other treatment
Favour ICS High+LABA

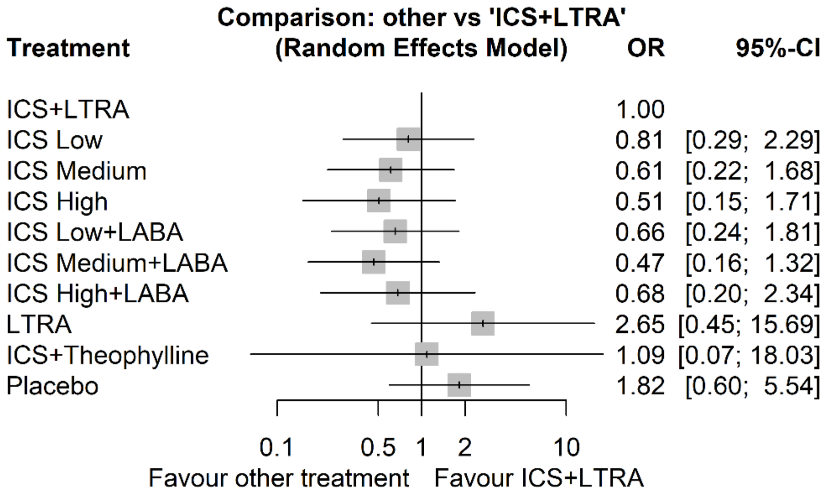
Comparison: other vs 'ICS+LTRA'
Treatment
(Random Effects Model)
OR
95%-CI
ICS+LTRA 1.00
ICS Low 0.81 [0.29; 2.29]
ICS Medium 0.61 [0.22; 1.68]
ICS High 0.51 [0.15; 1.71]
ICS Low+LABA 0.66 [0.24; 1.81]
ICS Medium+LABA 0.47 [0.16; 1.32]
ICS High+LABA 0.68 [0.20; 2.34]
LTRA 2.65 [0.45; 15.69]
ICS+Theophylline 1.09 [0.07; 18.03]
Placebo 1.82 [0.60; 5.54]
0.1 0.5 1 2 10
Favour other treatment
Favour ICS+LTRA

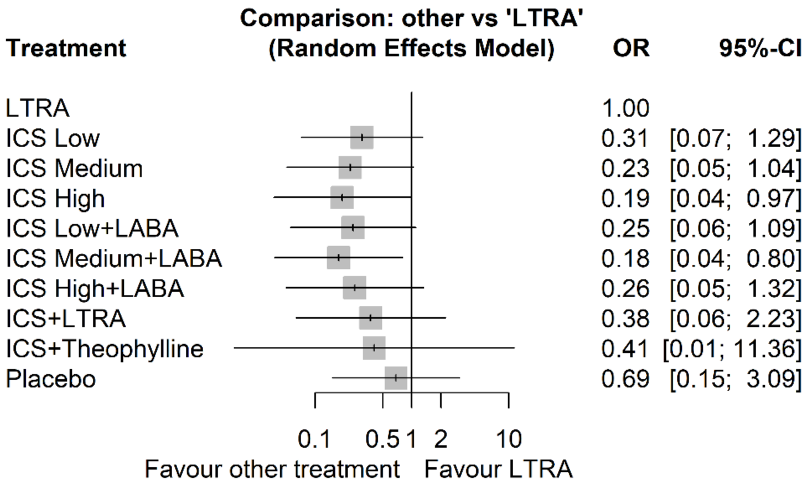
Comparison: other vs 'LTRA'
Treatment
(Random Effects Model)
OR
95%-CI
LTRA 1.00
ICS Low 0.31 [0.07; 1.29]
ICS Medium 0.23 [0.05; 1.04]
ICS High 0.19 [0.04; 0.97]
ICS Low+LABA 0.25 [0.06; 1.09]
ICS Medium+LABA 0.18 [0.04; 0.80]
ICS High+LABA 0.26 [0.05; 1.32]
ICS+LTRA 0.38 [0.06; 2.23]
ICS+Theophylline 0.41 [0.01; 11.36]
Placebo 0.69 [0.15; 3.09]
0.1 0.5 1 2 10
Favour other treatment
Favour LTRA

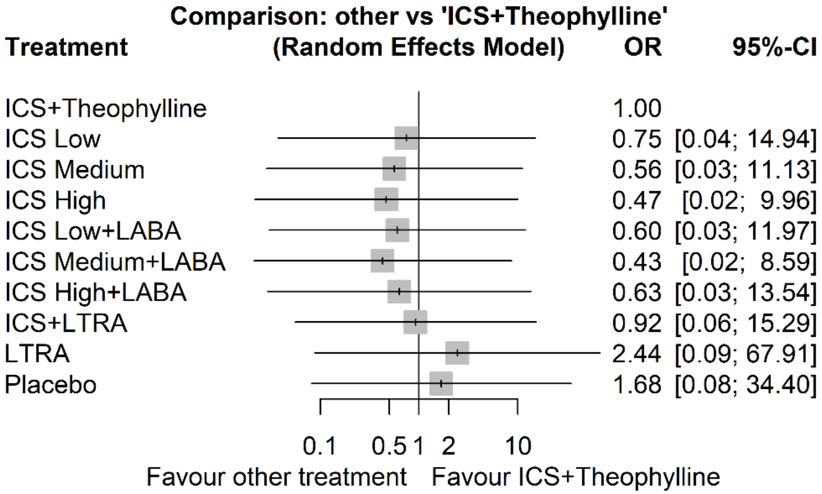

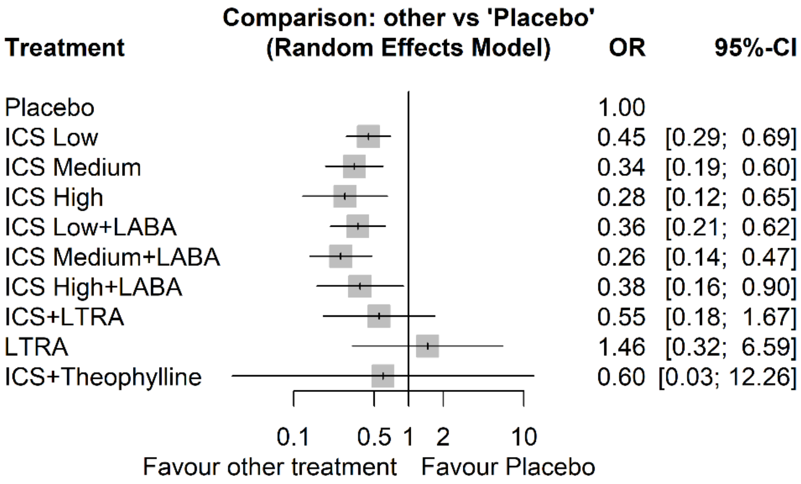

The probability of having exacerbation was modelled.
OR: odds ratio; CI: confidence interval; IPD: individual participant data; AgD: aggregate data; ICS: inhaled corticosteroid; LABA: Long-Acting β2-Agonist; LTRA: Leukotriene Receptor Antagonist.
Quantifying heterogeneity / inconsistency: tau^2 = 0; tau = 0; I^2 = 0% [0.0%; 33.5%]
Tests of heterogeneity (within designs) and inconsistency (between designs):
Total ─ Q = 42.88, d.f. = 47, p-value = 0.6436
Within designs ─ Q = 16.34, d.f. = 22, p-value = 0.7986
Between designs ─ Q = 26.54, d.f. = 25, p-value = 0.3791

Figure S5. Network plot and rankings for the fixed effect network meta-analysis (ICS grouped when combined with LABA) for exacerbations – Analysis B1

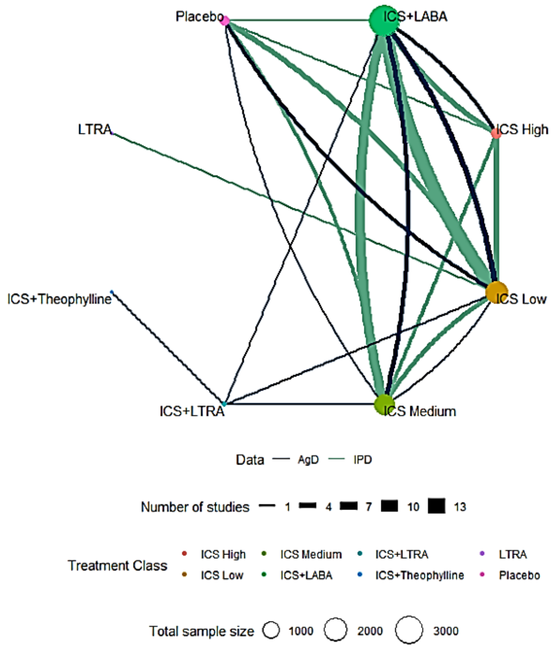

A, Network plot

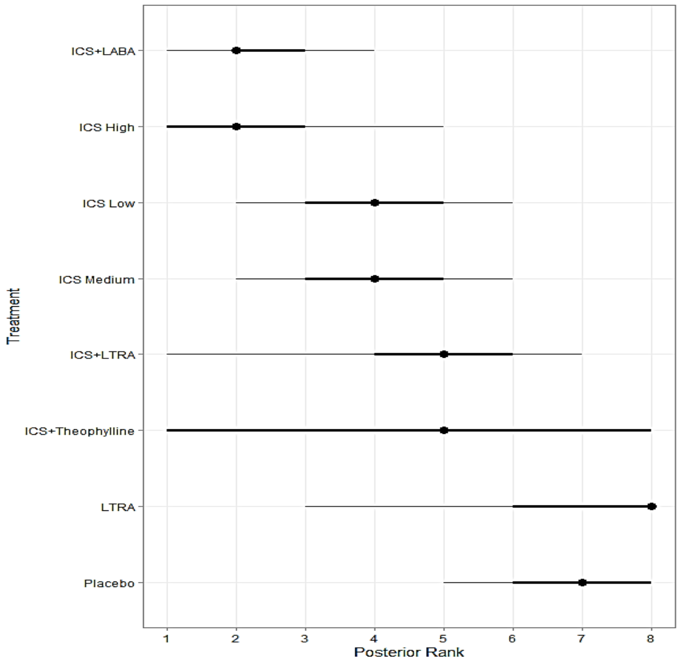

B, Posterior treatment rankings from fitted NMA model. Rank median (point), IQR (bold line), 95% interval (thin line). Lower rank is better.

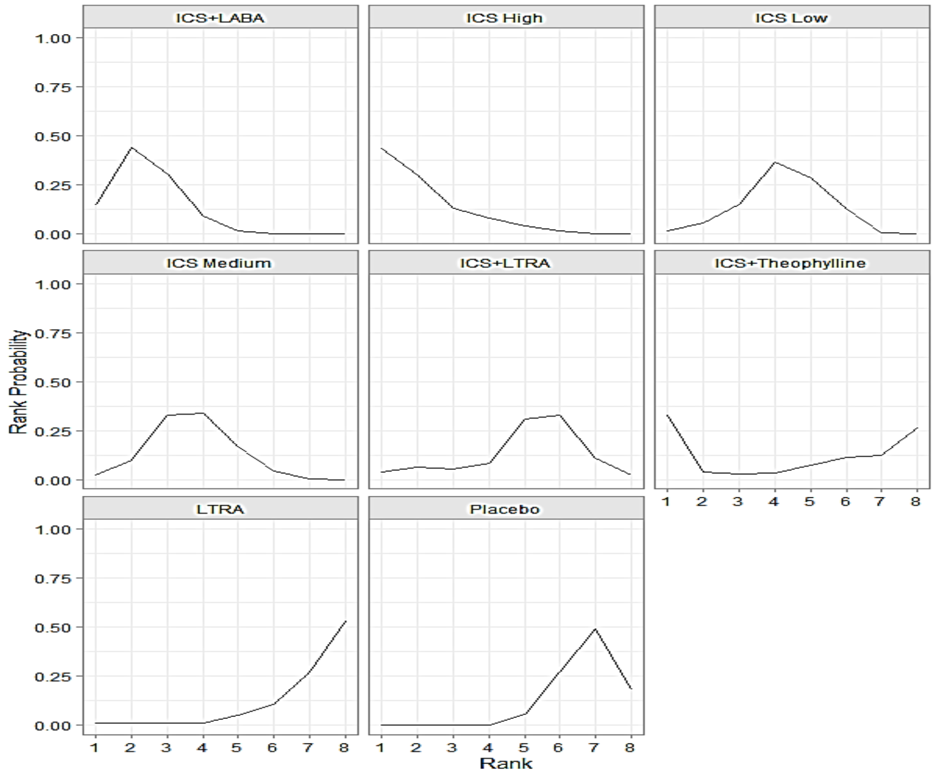

C, Rank probability plots from fitted NMA model.

Figure S6. Network plot and rankings for the fixed effect network meta-analysis (ICS stratified when combined with LABA) for asthma control – Analysis A2

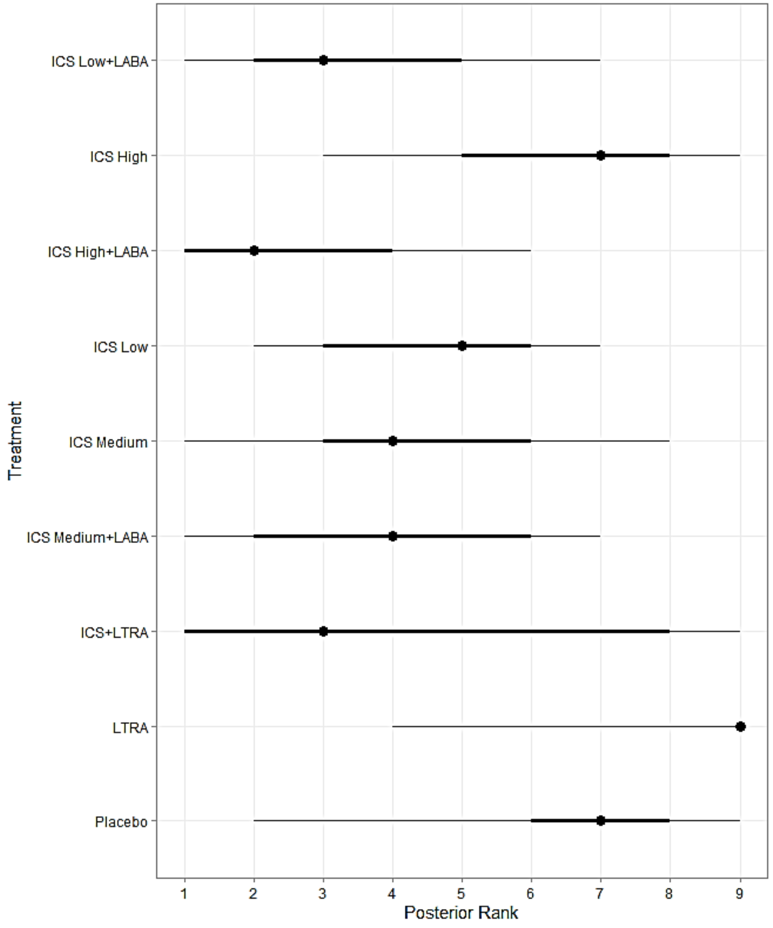

A, Posterior treatment rankings from fitted NMA model. Rank median (point), IQR (bold line), 95% interval (thin line). Lower rank is better.

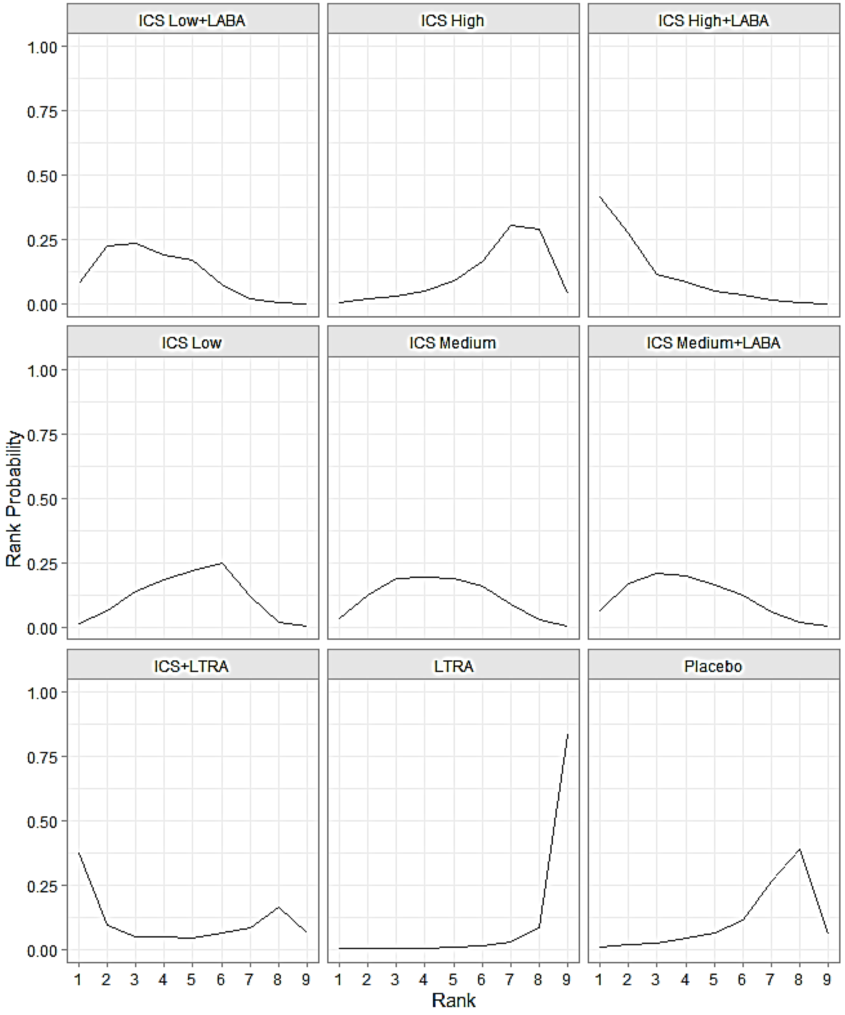

B, Rank probability plots from fitted NMA model.

Figure S7 (parts 1 to 3). Asthma Control frequentist fixed effect network meta-analysis (OR, 95% Cr) with IPD (Analysis A2: 16 trials, 3027 participants, 2453 events)

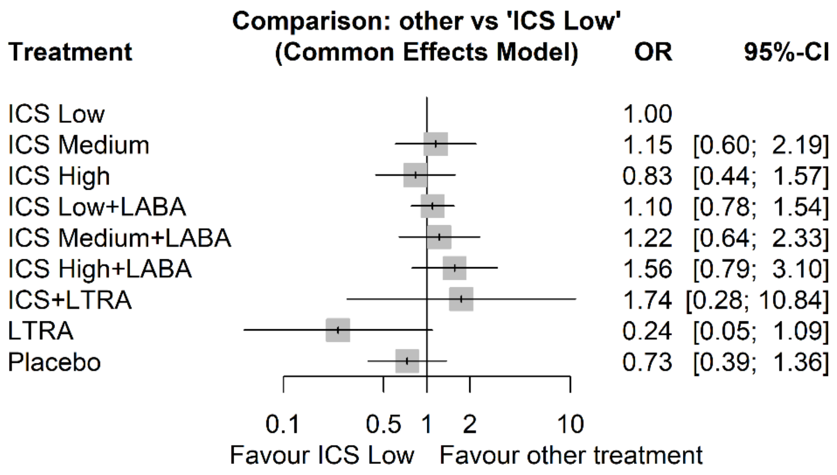

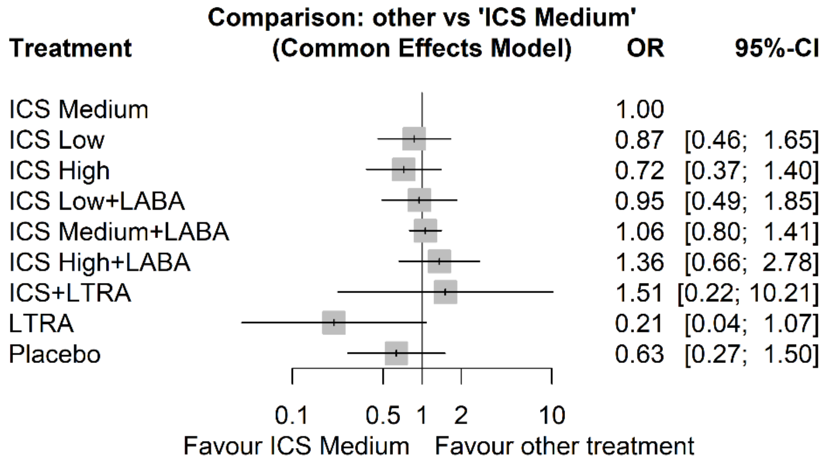

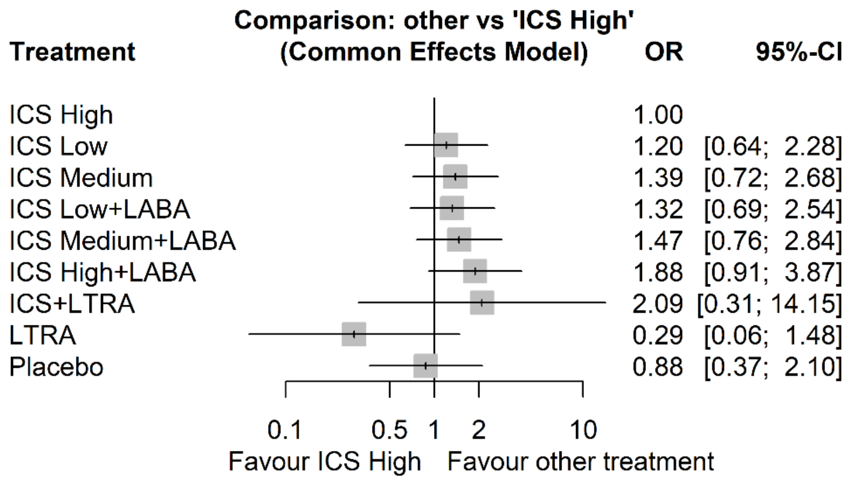

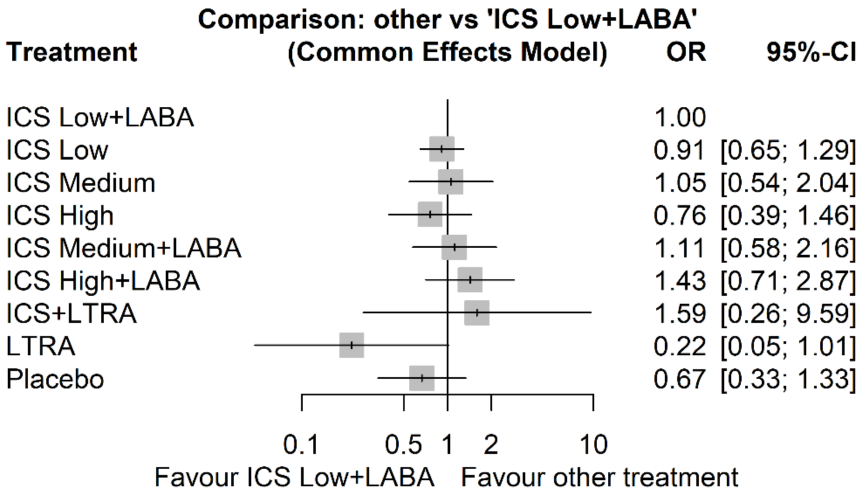

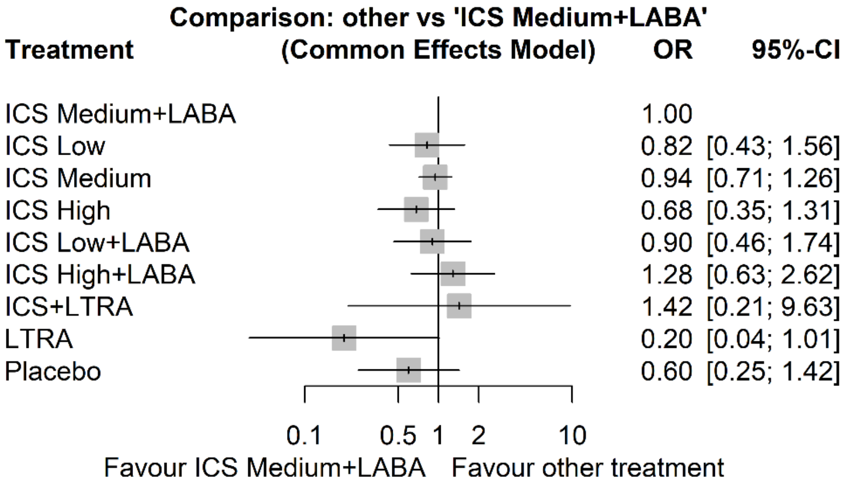
Comparison: other vs 'ICS Medium+LABA'
(Common Effects Model)
Treatment
OR
95%-CI
ICS Medium+LABA 1.00
ICS Low 0.82 [0.43; 1.56]
ICS Medium 0.94 [0.71; 1.26]
ICS High 0.68 [0.35; 1.31]
ICS Low+LABA 0.90 [0.46; 1.74]
ICS High+LABA 1.28 [0.63; 2.62]
ICS+LTRA 1.42 [0.21; 9.63]
LTRA 0.20 [0.04; 1.01]
Placebo 0.60 [0.25; 1.42]
0.1
0.5
1
2
10
Favour ICS Medium+LABA
Favour other treatment

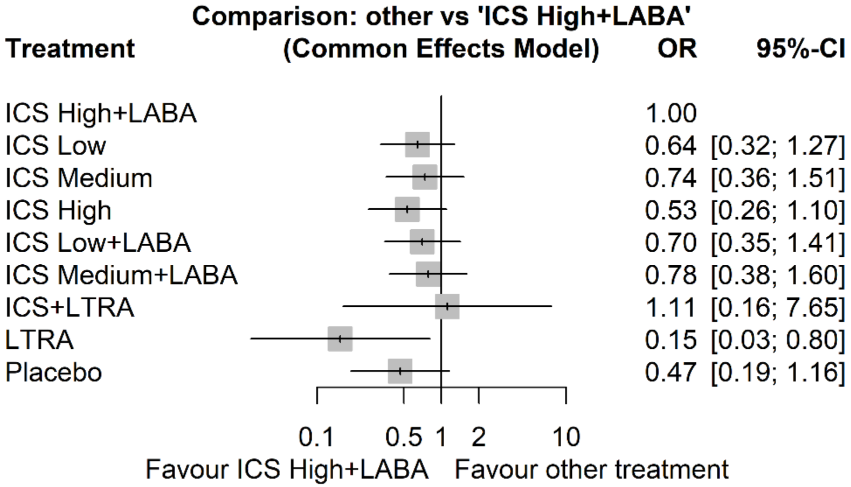
Comparison: other vs 'ICS High+LABA'
(Common Effects Model)
Treatment
OR
95%-CI
ICS High+LABA 1.00
ICS Low 0.64 [0.32; 1.27]
ICS Medium 0.74 [0.36; 1.51]
ICS High 0.53 [0.26; 1.10]
ICS Low+LABA 0.70 [0.35; 1.41]
ICS Medium+LABA 0.78 [0.38; 1.60]
ICS+LTRA 1.11 [0.16; 7.65]
LTRA 0.15 [0.03; 0.80]
Placebo 0.47 [0.19; 1.16]
0.1
0.5
1
2
10
Favour ICS High+LABA
Favour other treatment

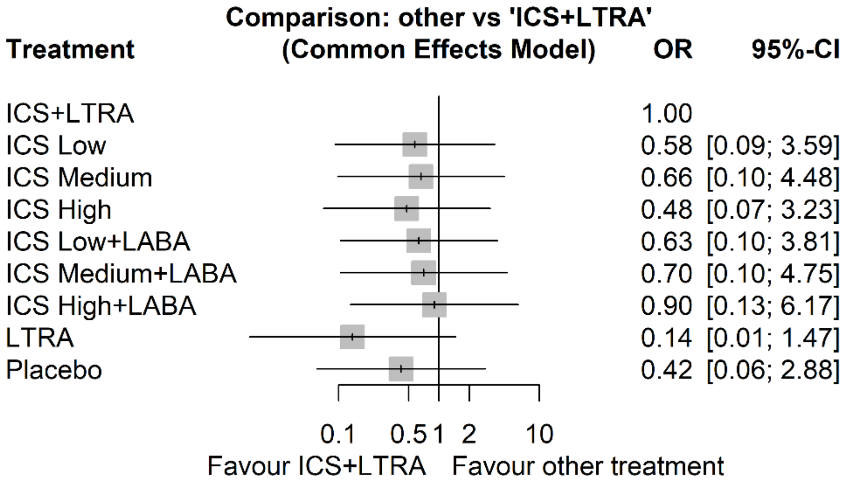
Comparison: other vs 'ICS+LTRA'
(Common Effects Model)
Treatment
OR
95%-CI
ICS+LTRA 1.00
ICS Low 0.58 [0.09; 3.59]
ICS Medium 0.66 [0.10; 4.48]
ICS High 0.48 [0.07; 3.23]
ICS Low+LABA 0.63 [0.10; 3.81]
ICS Medium+LABA 0.70 [0.10; 4.75]
ICS High+LABA 0.90 [0.13; 6.17]
LTRA 0.14 [0.01; 1.47]
Placebo 0.42 [0.06; 2.88]
0.1
0.5
1
2
10
Favour ICS+LTRA
Favour other treatment

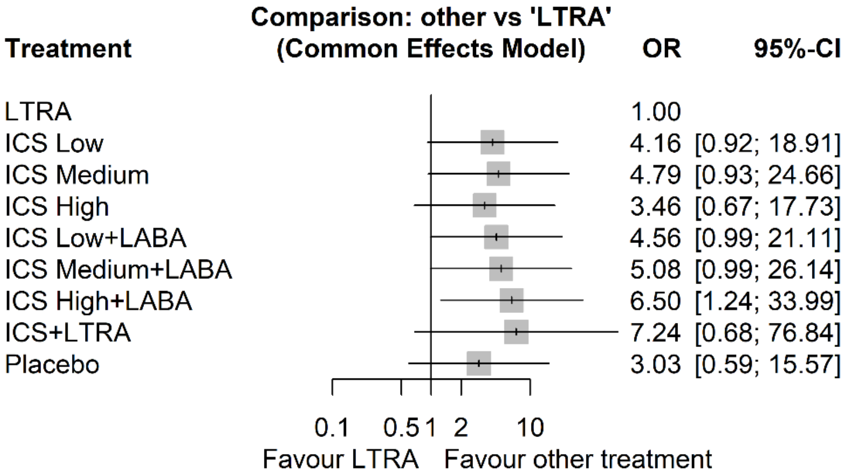
Comparison: other vs 'LTRA'
(Common Effects Model)
Treatment
OR
95%-CI
LTRA 1.00
ICS Low 4.16 [0.92; 18.91]
ICS Medium 4.79 [0.93; 24.66]
ICS High 3.46 [0.67; 17.73]
ICS Low+LABA 4.56 [0.99; 21.11]
ICS Medium+LABA 5.08 [0.99; 26.14]
ICS High+LABA 6.50 [1.24; 33.99]
ICS+LTRA 7.24 [0.68; 76.84]
Placebo 3.03 [0.59; 15.57]
0.1
0.5
1
2
10
Favour LTRA
Favour other treatment

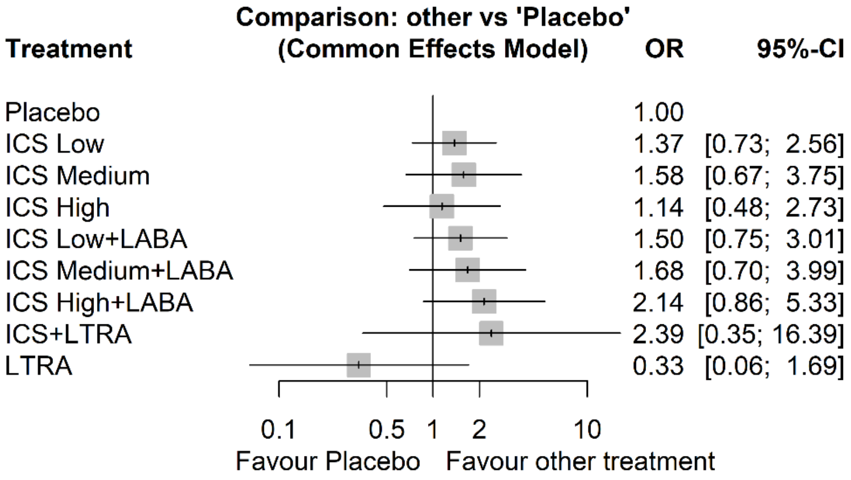

The probability of having good/total asthma control was modelled.
OR: odds ratio; CI: confidence interval; IPD: individual participant data; ICS: inhaled corticosteroid; LABA: Long-Acting β_2-Agonist; LTRA: Leukotriene Receptor Antagonist

Quantifying heterogeneity / inconsistency: tau^2 = 0.0834; tau = 0.2887; I^2 = 16% [0.0%; 49.6%].

Tests of heterogeneity (within designs) and inconsistency (between designs):

Total ─ Q = 25.00, d.f. = 21, p-value = 0.2471
Within designs ─ Q = 0.66, d.f. = 3, p-value = 0.8832
Between designs ─ Q = 24.34, d.f. = 18, p-value = 0.1441

Figure S8. Network plot and rankings for the fixed effect network meta-analysis (ICS grouped when combined with LABA) for asthma control – Analysis B2

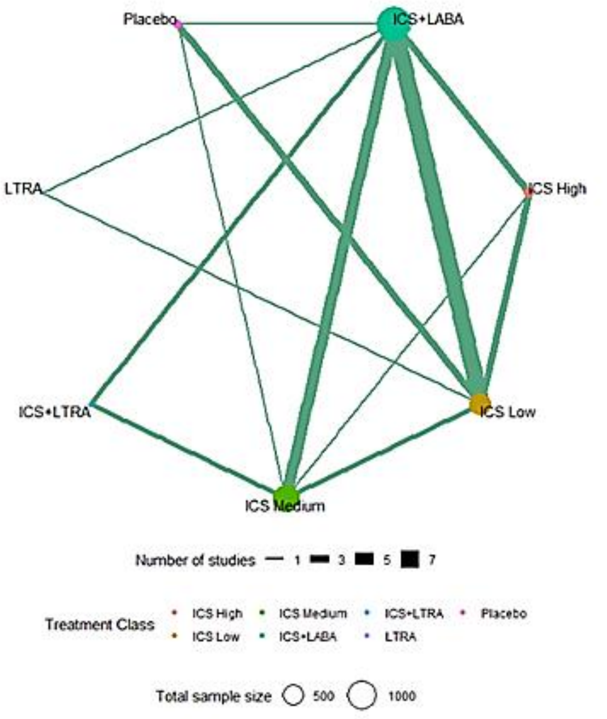

A, Network plot

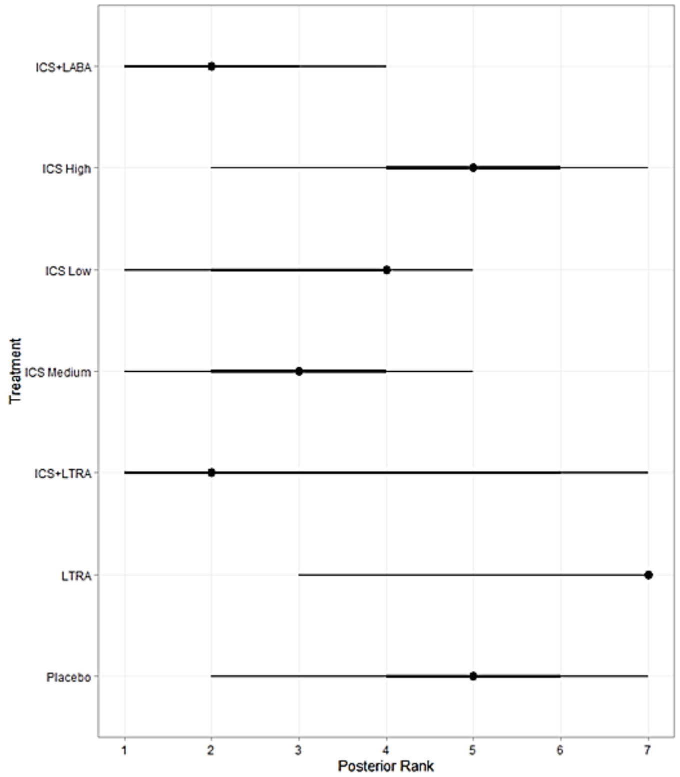

B, Posterior treatment rankings from fitted NMA model. Rank median (point), IQR (bold line), 95% interval (thin line). Lower rank is better.

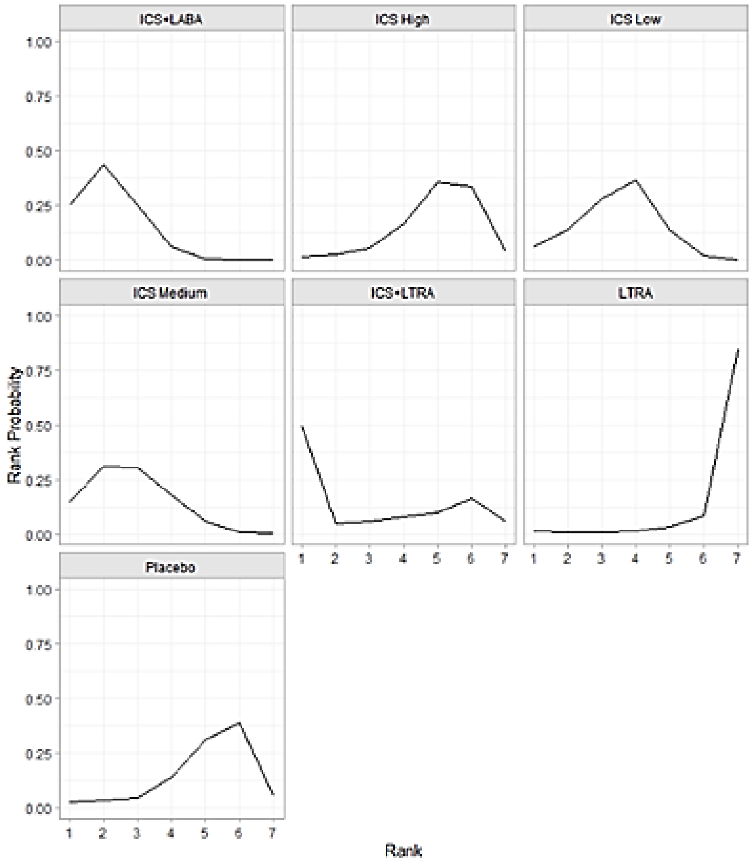

C, Rank probability plots from fitted NMA model.

Figure S9. Network plot and rankings for the random-effects network meta-analysis (individual compounds) for asthma control – Analysis C2

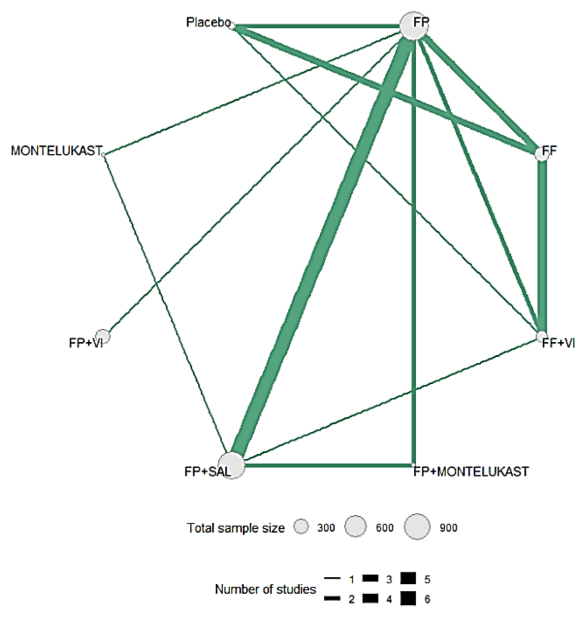

A, Network plot

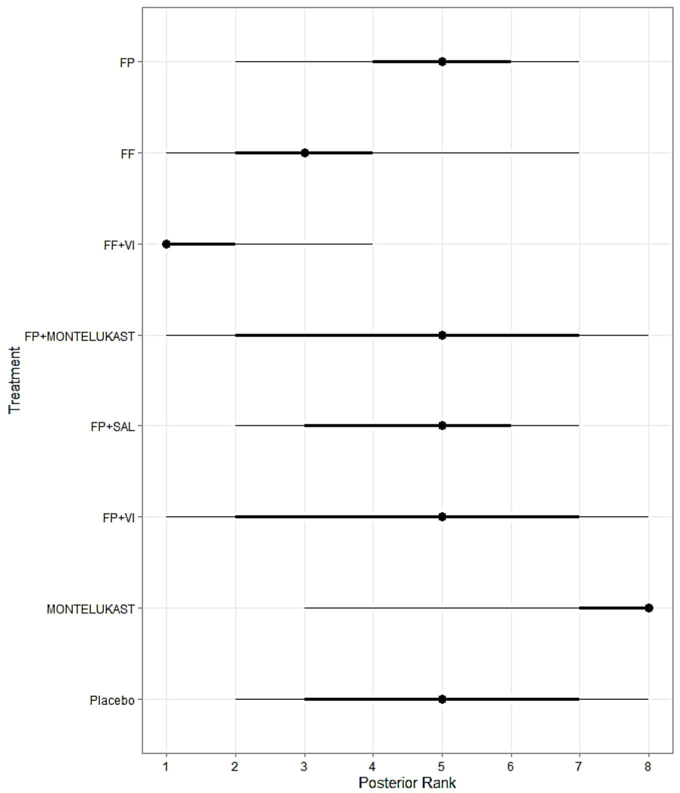

B, Posterior treatment rankings from fitted NMA model. Rank median (point), IQR (bold line), 95% interval (thin line). Lower rank is better.

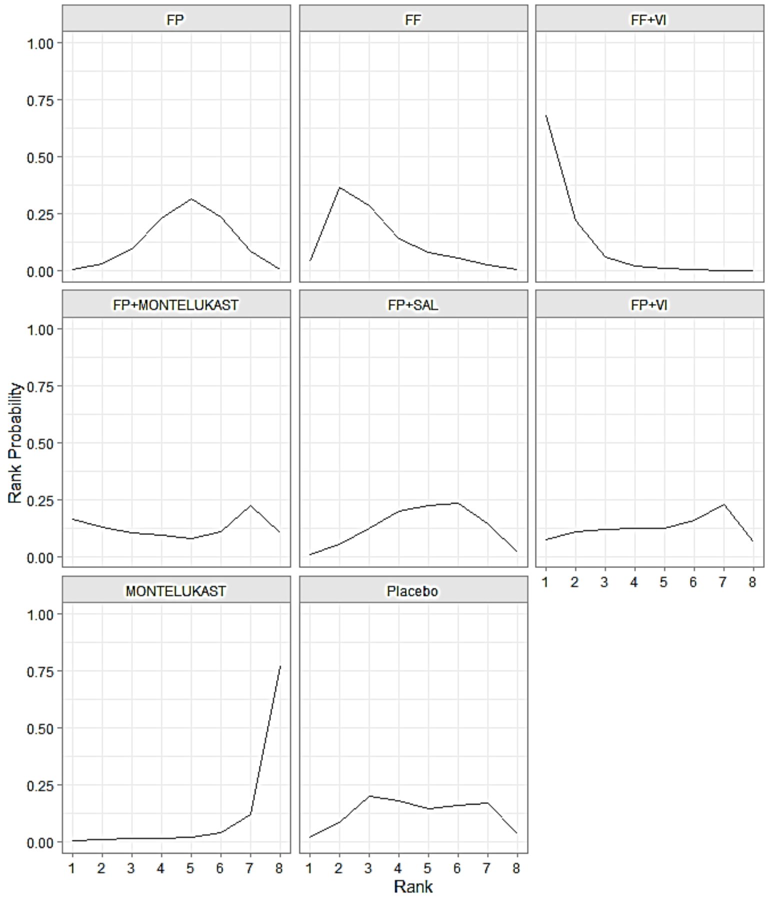

C, Rank probability plots from fitted NMA model.

Figure S10. Network plot and rankings for the fixed effect network meta-analysis (ICS stratified when combined with LABA) for FEV_1 – Analysis A3

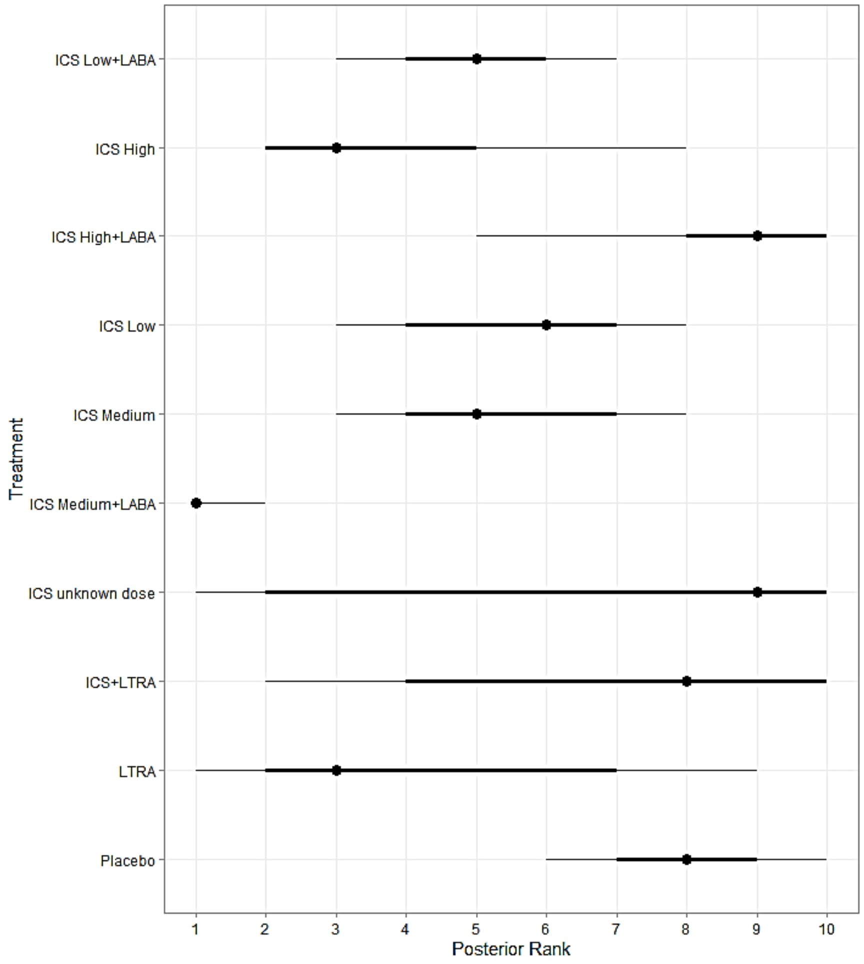

A, Posterior treatment rankings from fitted NMA model. Rank median (point), IQR (bold line), 95% interval (thin line). Lower rank is better.

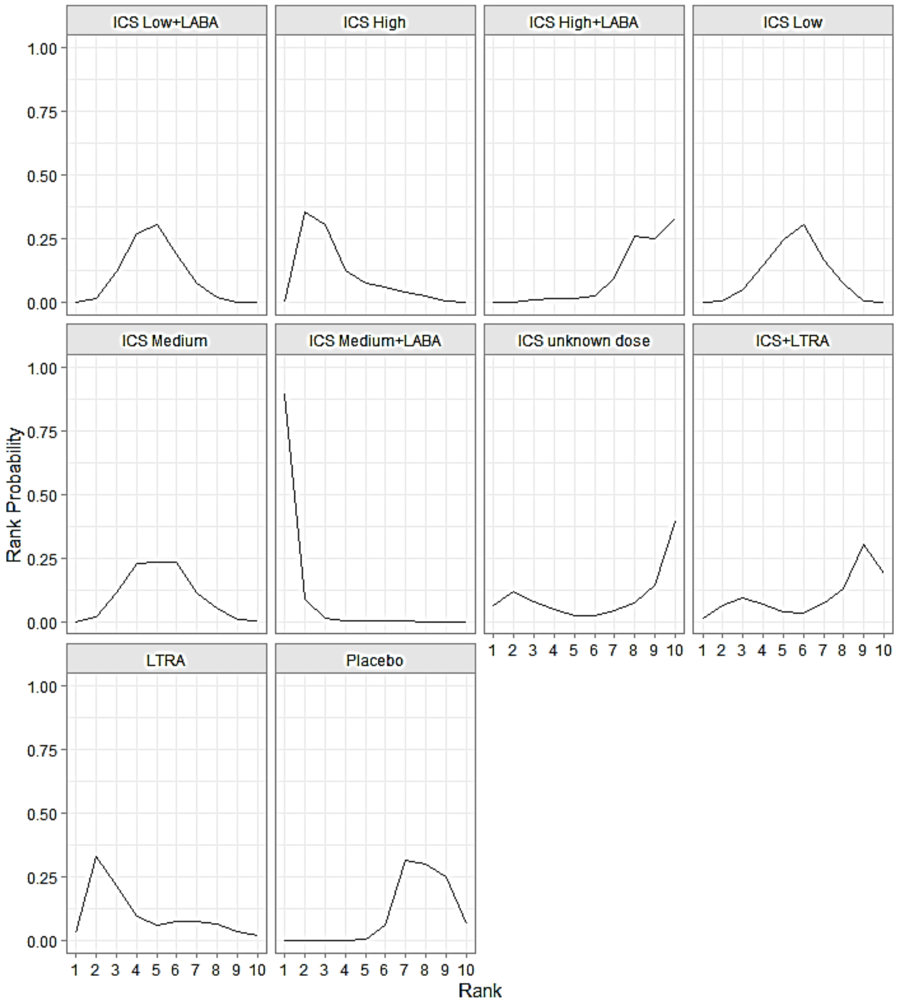

B, Rank probability plots from fitted NMA model.

Figure S11 (parts 1 to 3). FEV$_1$ frequentist fixed effect network meta-analysis (MD, 95% CI) with IPD and AgD (Analysis A3: 23 trials, 2518 participants)

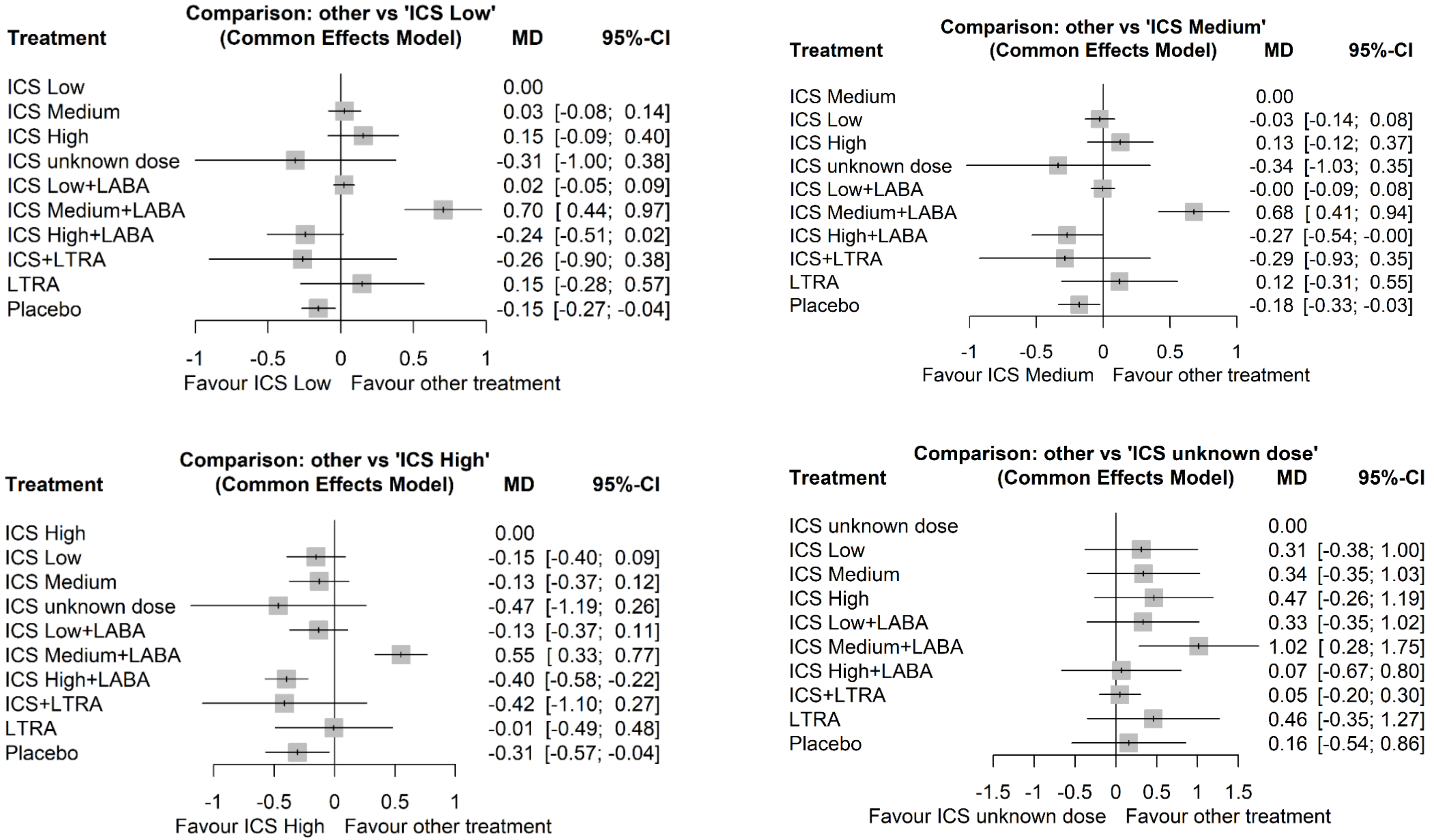

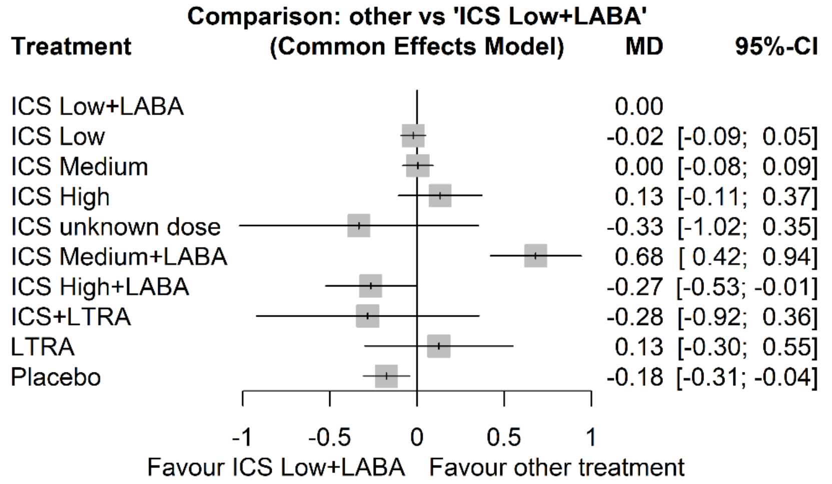
Comparison: other vs 'ICS Low+LABA'
Treatment
(Common Effects Model)
MD
95%-CI
ICS Low+LABA 0.00
ICS Low -0.02 [-0.09; 0.05]
ICS Medium 0.00 [-0.08; 0.09]
ICS High 0.13 [-0.11; 0.37]
ICS unknown dose -0.33 [-1.02; 0.35]
ICS Medium+LABA 0.68 [0.42; 0.94]
ICS High+LABA -0.27 [-0.53; -0.01]
ICS+LTRA -0.28 [-0.92; 0.36]
LTRA 0.13 [-0.30; 0.55]
Placebo -0.18 [-0.31; -0.04]
-1 -0.5 0 0.5 1
Favour ICS Low+LABA
Favour other treatment

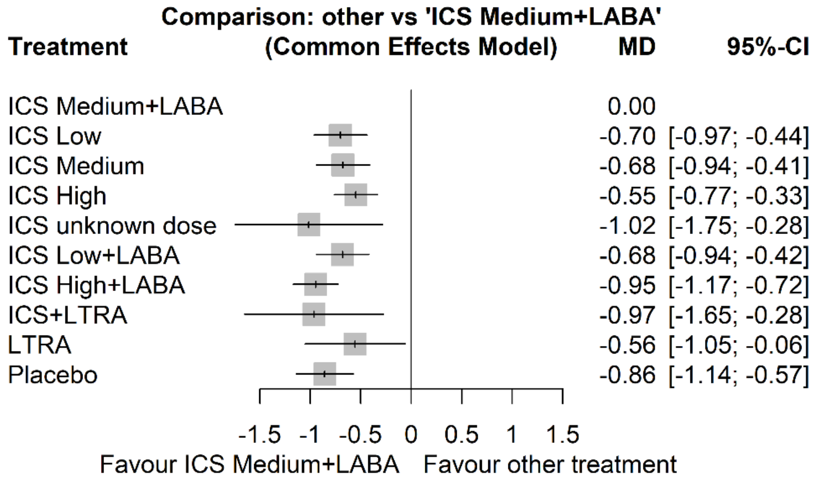
Comparison: other vs 'ICS Medium+LABA'
Treatment
(Common Effects Model)
MD
95%-CI
ICS Medium+LABA 0.00
ICS Low -0.70 [-0.97; -0.44]
ICS Medium -0.68 [-0.94; -0.41]
ICS High -0.55 [-0.77; -0.33]
ICS unknown dose -1.02 [-1.75; -0.28]
ICS Low+LABA -0.68 [-0.94; -0.42]
ICS High+LABA -0.95 [-1.17; -0.72]
ICS+LTRA -0.97 [-1.65; -0.28]
LTRA -0.56 [-1.05; -0.06]
Placebo -0.86 [-1.14; -0.57]
-1.5 -1 -0.5 0 0.5 1 1.5
Favour ICS Medium+LABA
Favour other treatment

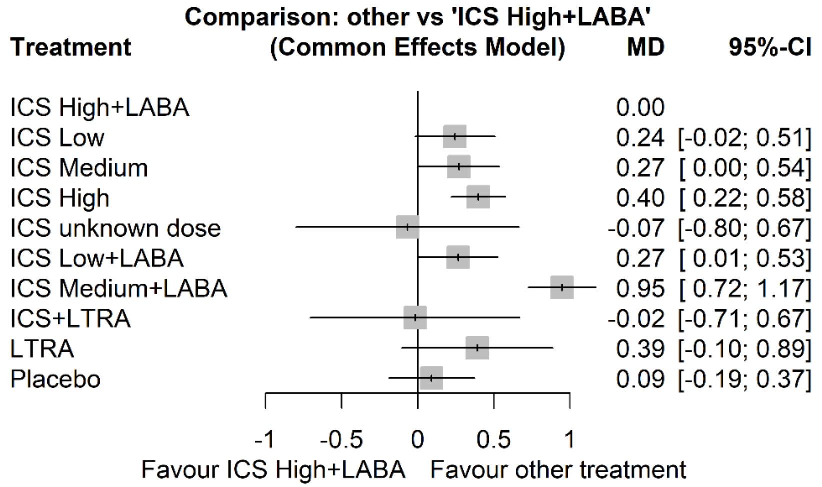
Comparison: other vs 'ICS High+LABA'
Treatment
(Common Effects Model)
MD
95%-CI
ICS High+LABA 0.00
ICS Low 0.24 [-0.02; 0.51]
ICS Medium 0.27 [0.00; 0.54]
ICS High 0.40 [0.22; 0.58]
ICS unknown dose -0.07 [-0.80; 0.67]
ICS Low+LABA 0.27 [0.01; 0.53]
ICS Medium+LABA 0.95 [0.72; 1.17]
ICS+LTRA -0.02 [-0.71; 0.67]
LTRA 0.39 [-0.10; 0.89]
Placebo 0.09 [-0.19; 0.37]
-1 -0.5 0 0.5 1
Favour ICS High+LABA
Favour other treatment

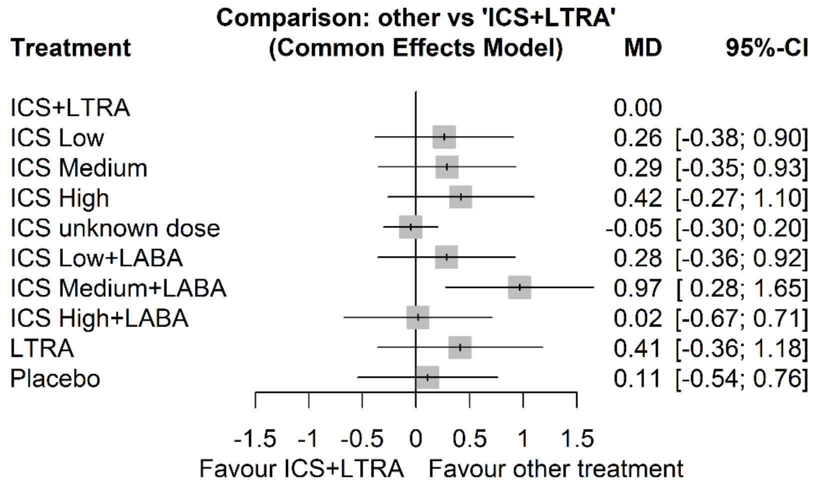
Comparison: other vs 'ICS+LTRA'
Treatment
(Common Effects Model)
MD
95%-CI
ICS+LTRA 0.00
ICS Low 0.26 [-0.38; 0.90]
ICS Medium 0.29 [-0.35; 0.93]
ICS High 0.42 [-0.27; 1.10]
ICS unknown dose -0.05 [-0.30; 0.20]
ICS Low+LABA 0.28 [-0.36; 0.92]
ICS Medium+LABA 0.97 [0.28; 1.65]
ICS High+LABA 0.02 [-0.67; 0.71]
LTRA 0.41 [-0.36; 1.18]
Placebo 0.11 [-0.54; 0.76]
-1.5 -1 -0.5 0 0.5 1 1.5
Favour ICS+LTRA
Favour other treatment

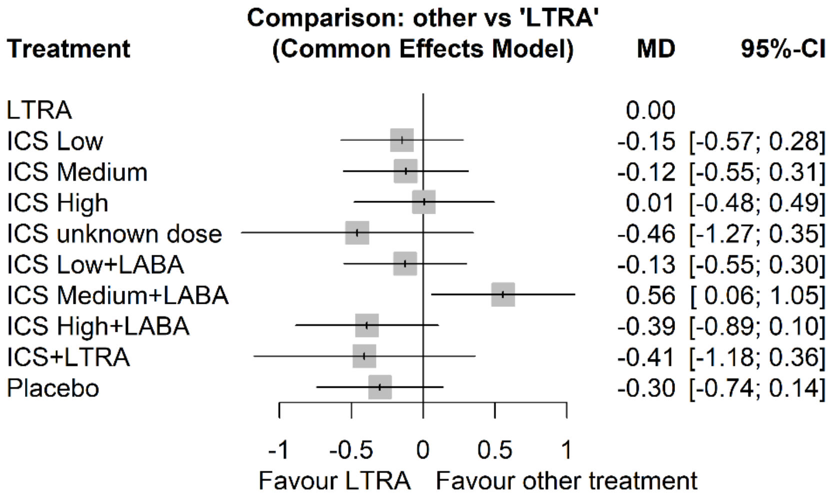

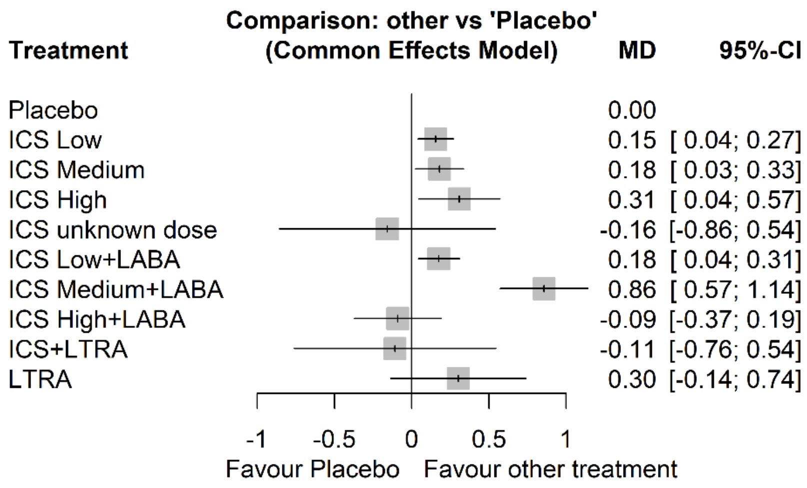

MD: mean difference; CI: confidence interval; IPD: individual participant data; ICS: inhaled corticosteroid; LABA: Long-Acting β2-Agonist; LTRA: Leukotriene Receptor Antagonist

Quantifying heterogeneity / inconsistency: tau-square = 0.0359; tau = 0.1894; I-square = 59.6% [36.1%; 74.4%].

Tests of heterogeneity (within designs) and inconsistency (between designs):
Total — Q = 54.43, d.f. = 22, p-value = 0.0001
Within designs — Q = 14.13, d.f. = 8, p-value = 0.0784
Between designs — Q = 40.29, d.f. = 14, p-value = 0.0002

Figure S12. Network plot and rankings for the random-effects network-meta-analysis (ICS grouped when combined with LABA) for FEV_1 – Analysis B3

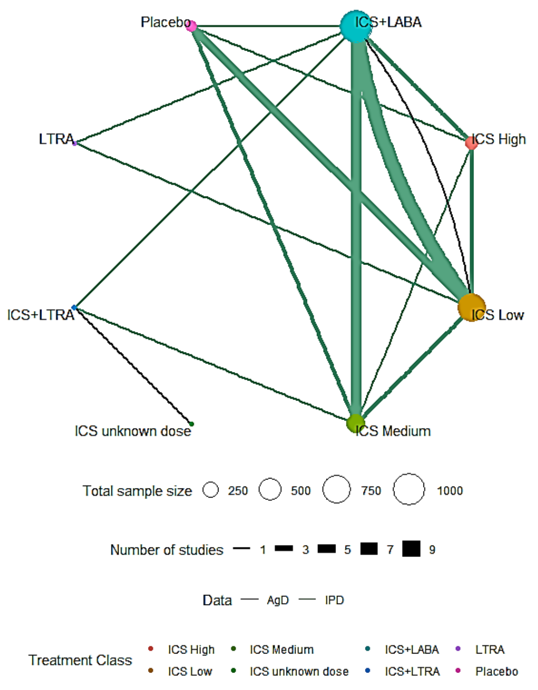

A, Network plot

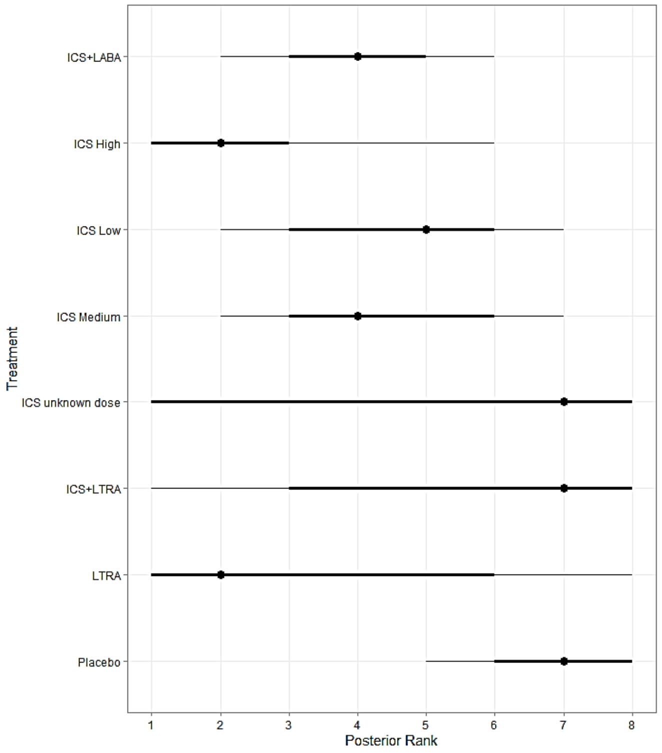

B, Posterior treatment rankings from fitted NMA model. Rank median (point), IQR (bold line), 95% interval (thin line). Lower rank is better.

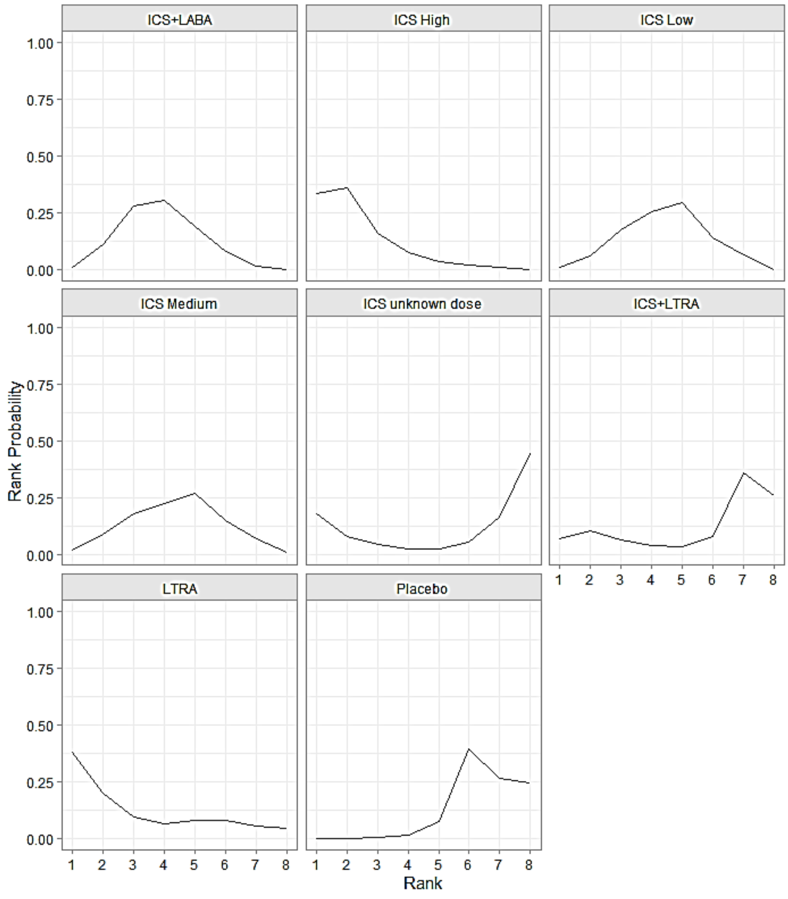

C, Rank probability plots from fitted NMA model.

Figure S13. Network plot and rankings for the fixed effect network meta-analysis (individual compounds) for FEV_1 – Analysis C3

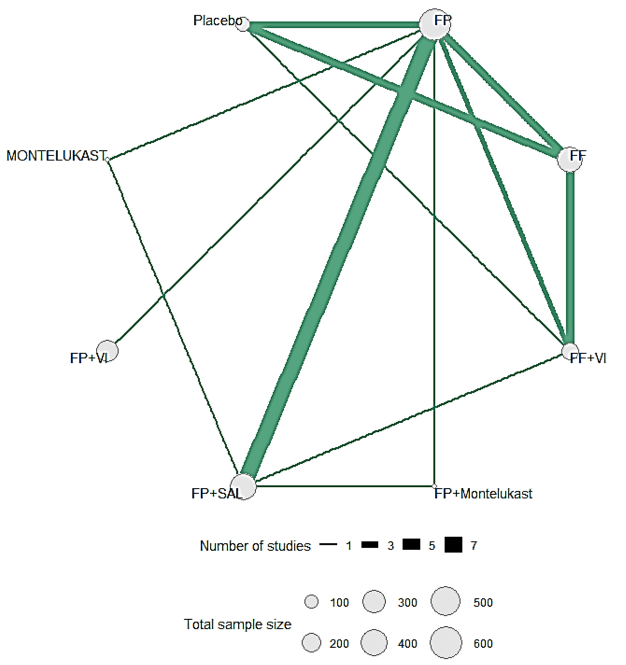

A, Network plot

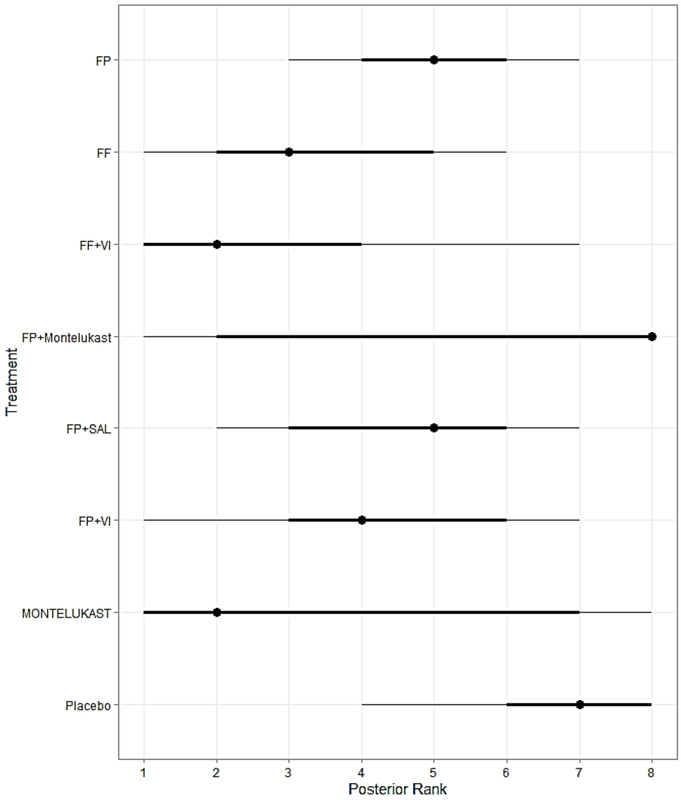

B, Posterior treatment rankings from fitted NMA model. Rank median (point), IQR (bold line), 95% interval (thin line). Lower rank is better.

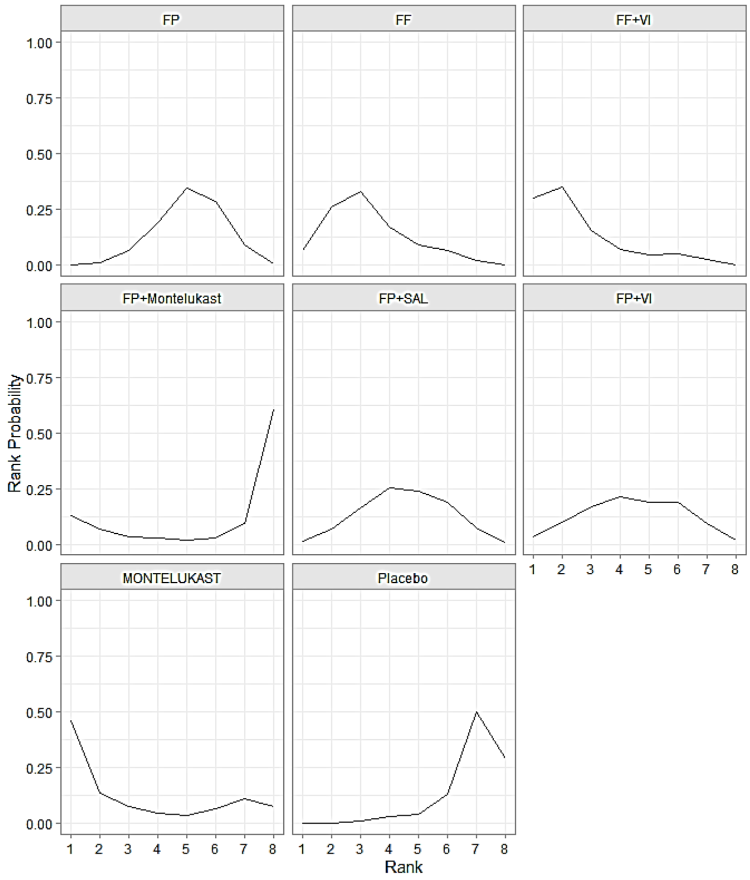

C, Rank probability plots from fitted NMA model.

Figure S14. Oral candidiasis (ICS dose stratified)

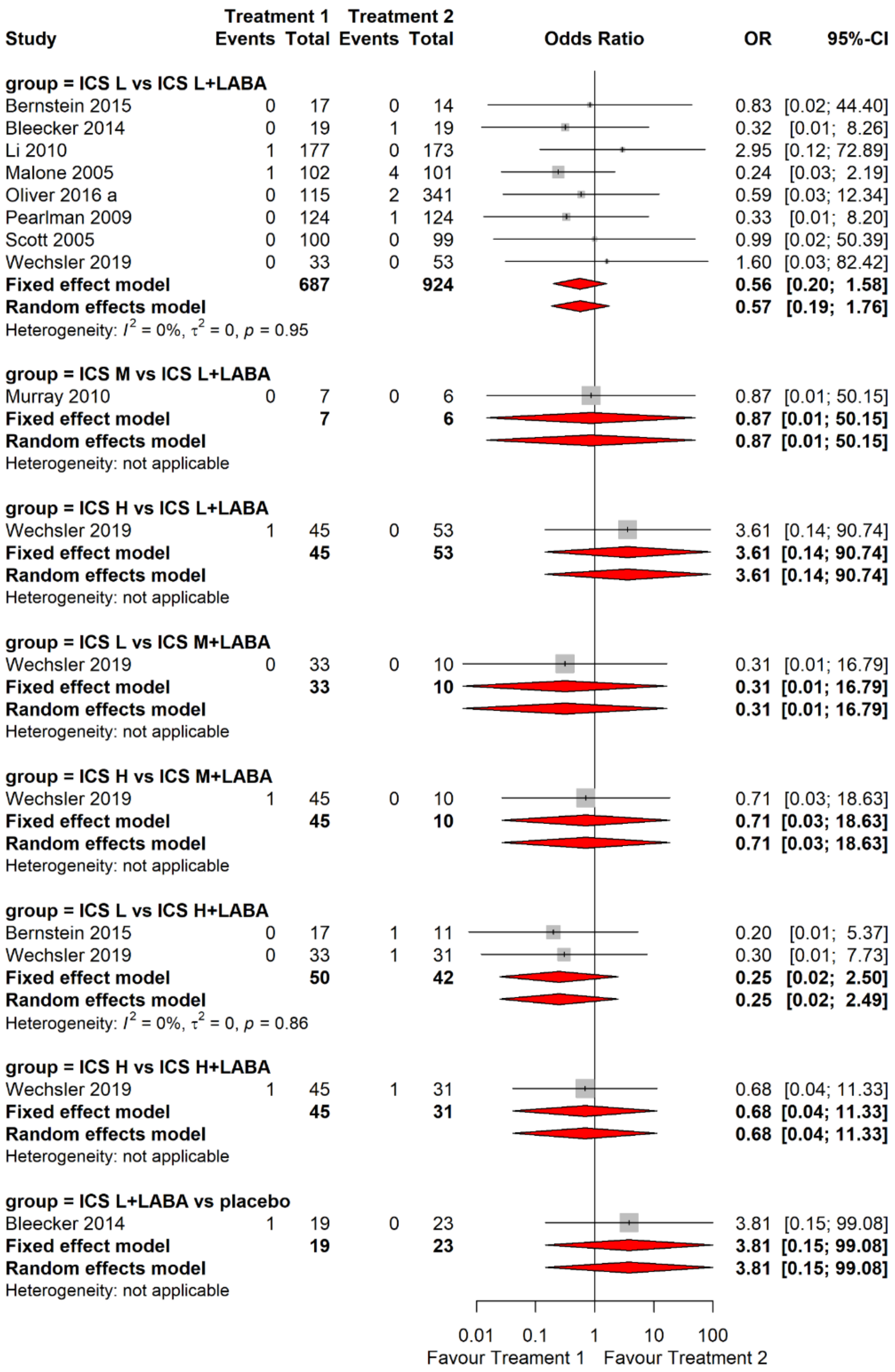

Meta-analyses with a frequentist approach (Mantel-Haenszel) based on all available comparisons. All data included (IPD and AgD where possible).
OR > 1 favours treatment 2. IPD = individual participant data; AgD = aggregate data; ICS = inhaled corticosteroids; LABA = long-acting beta-agonists; LTRA = leukotriene receptor antagonists; L = low dose; M = medium dose; H = high dose; OR = odds ratio; CI = confidence interval.

Figure S15. Oral candidiasis (any ICS dose combined with LABA)

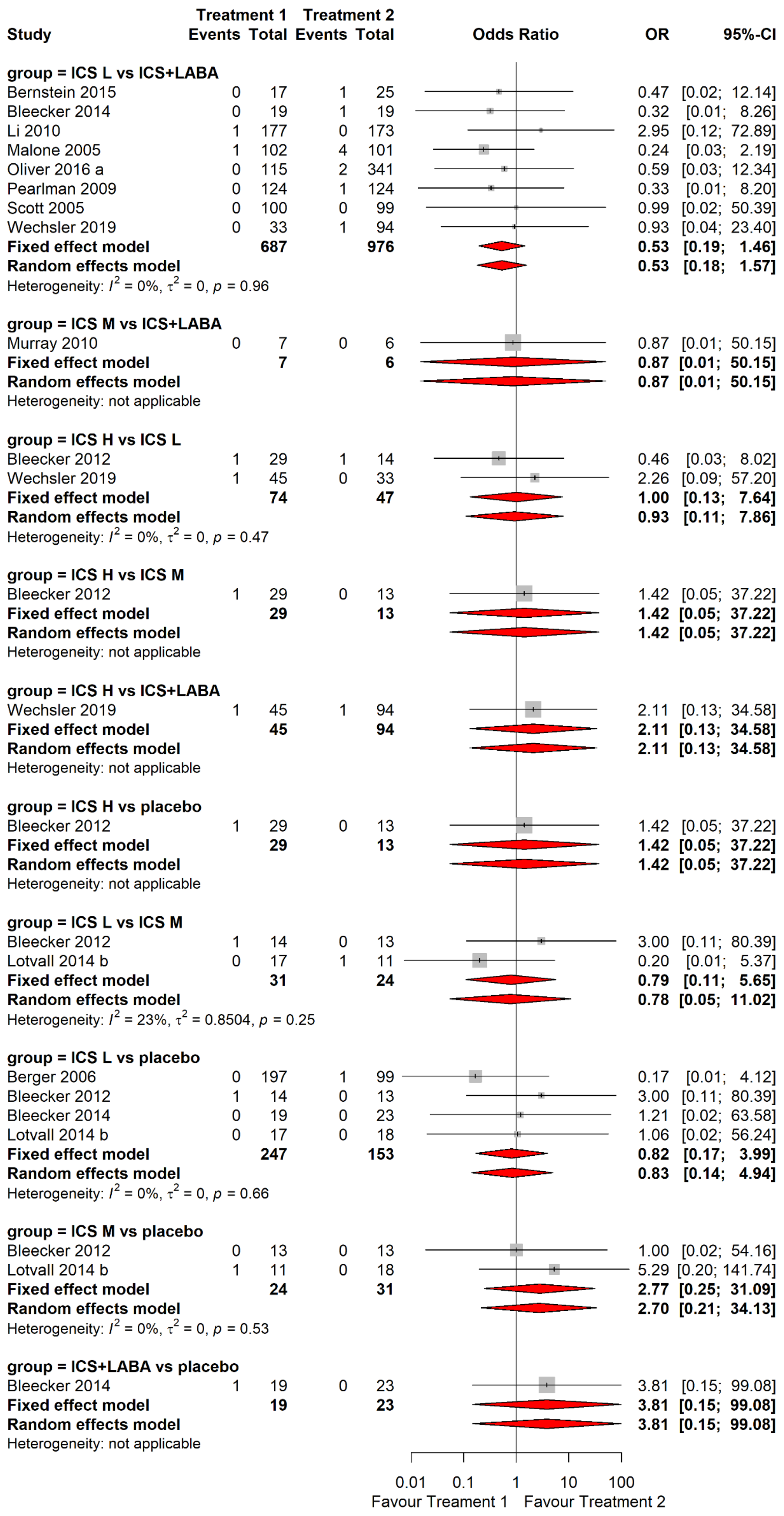

Meta-analyses with a frequentist approach (Mantel-Haenszel) based on all available comparisons. All data included (IPD and AgD where possible). OR > 1 favours treatment 2. IPD = individual participant data; AgD = aggregate data; ICS = inhaled corticosteroids; LABA = long-acting beta-agonists; LTRA = leukotriene receptor antagonists; L = low dose; M = medium dose; H = high dose; OR = odds ratio; CI = confidence interval.

Figure S16. Cardiac disorders (ICS dose grouped)

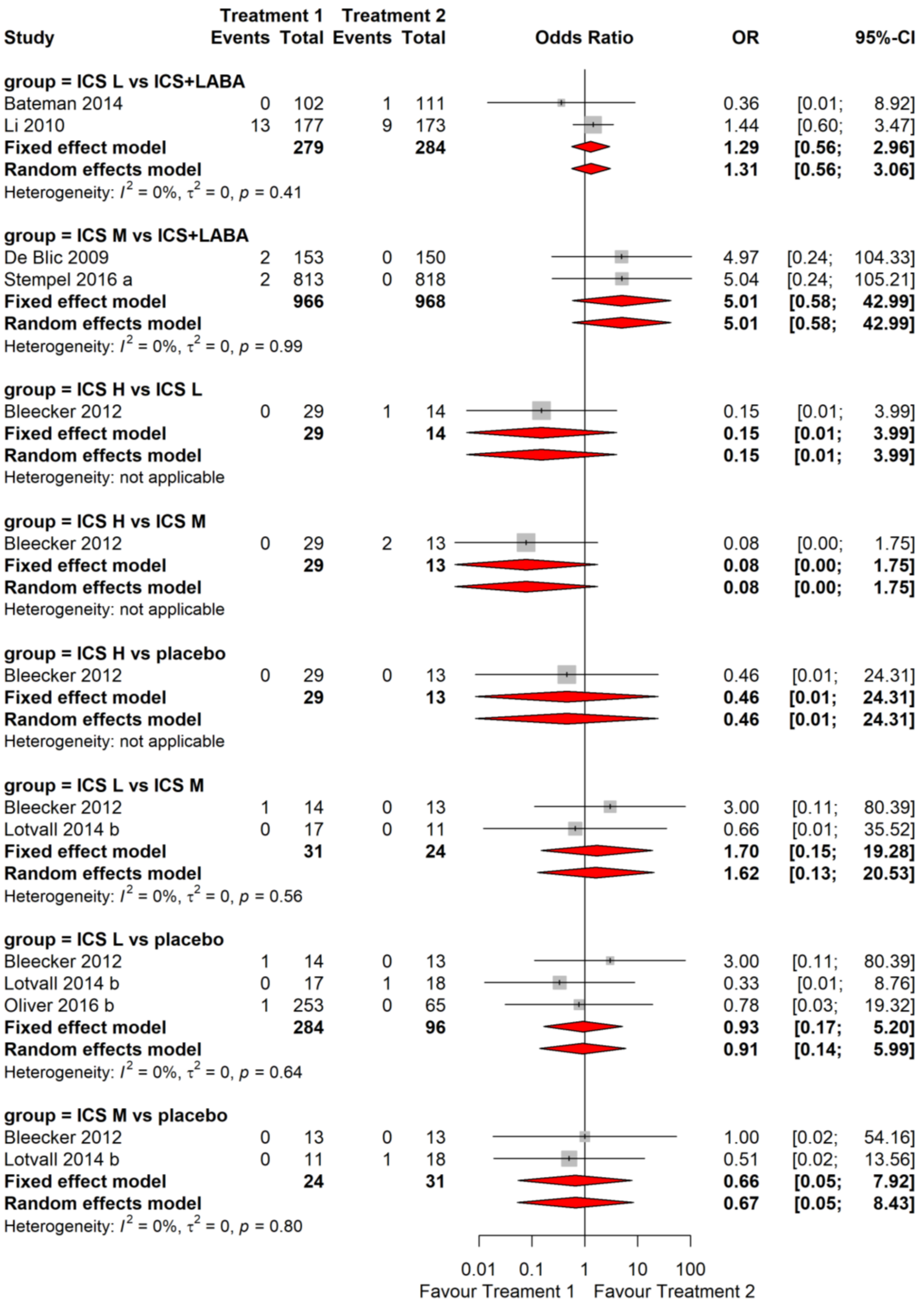

Meta-analysis with a frequentist approach (Mantel-Haenszel) based on all available comparisons. All data included (IPD only).
OR > 1 favours treatment 2. IPD = individual participant data; AgD = aggregate data; ICS = inhaled corticosteroids; LABA = long-acting beta-agonists; LTRA = leukotriene receptor antagonists; L = low dose; M = medium dose; H = high dose; OR = odds ratio; CI = confidence interval

Figure S17. Clinically significant electrocardiogram (ECG) favorable changes (ICS dose grouped)

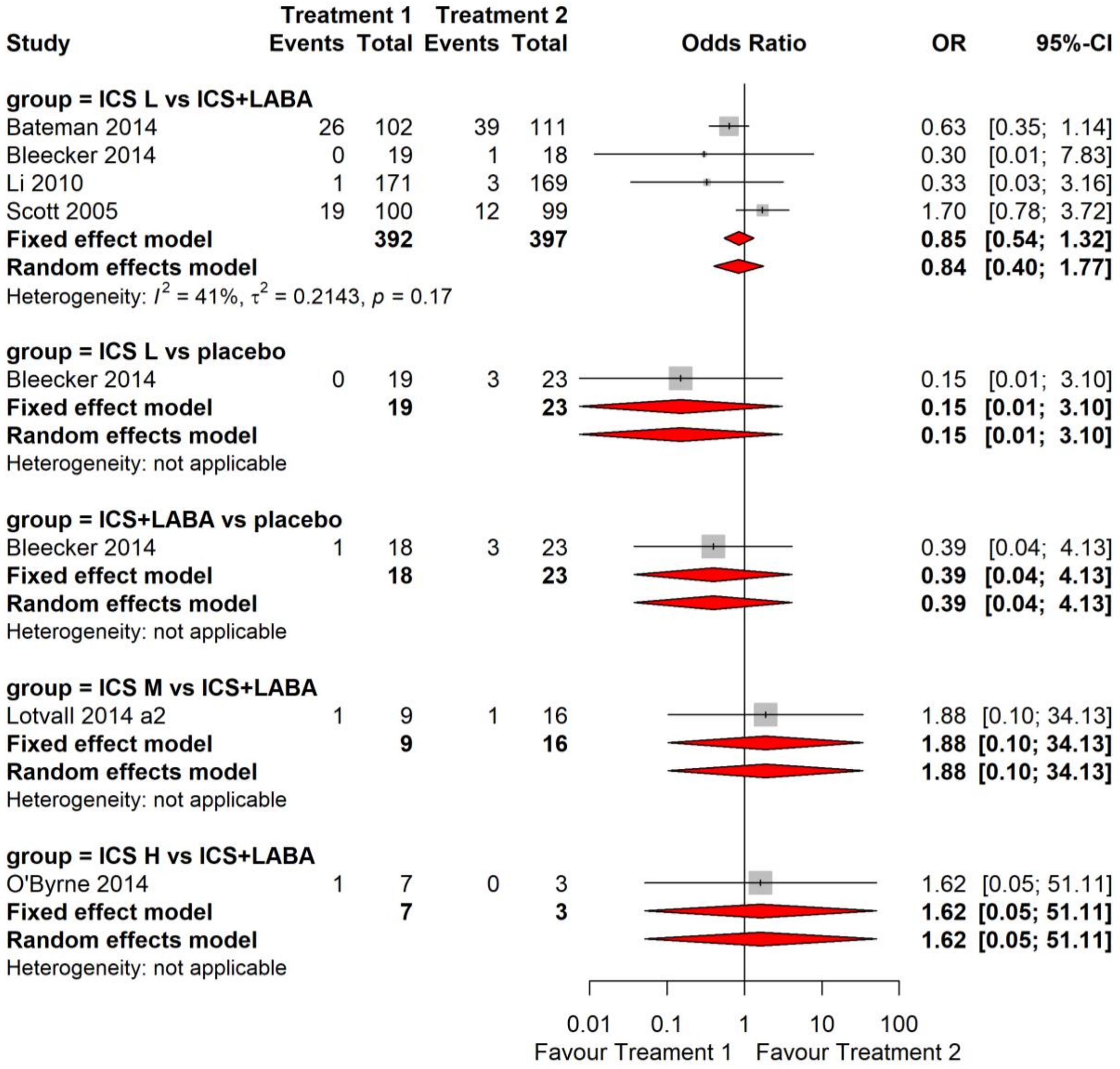

Meta-analysis with a frequentist approach (Mantel-Haenszel) based on all available comparisons. All data included (IPD only). OR > 1 favours treatment 2
IPD = individual participant data; AgD = aggregate data; ICS = inhaled corticosteroids; LABA = long-acting beta-agonists; LTRA = leukotriene receptor antagonists; L = low dose; M = medium dose; H = high dose; OR = odds ratio; CI = confidence interval.

Figure S18. Clinically significant electrocardiogram (ECG) unfavorable changes (ICS dose grouped)

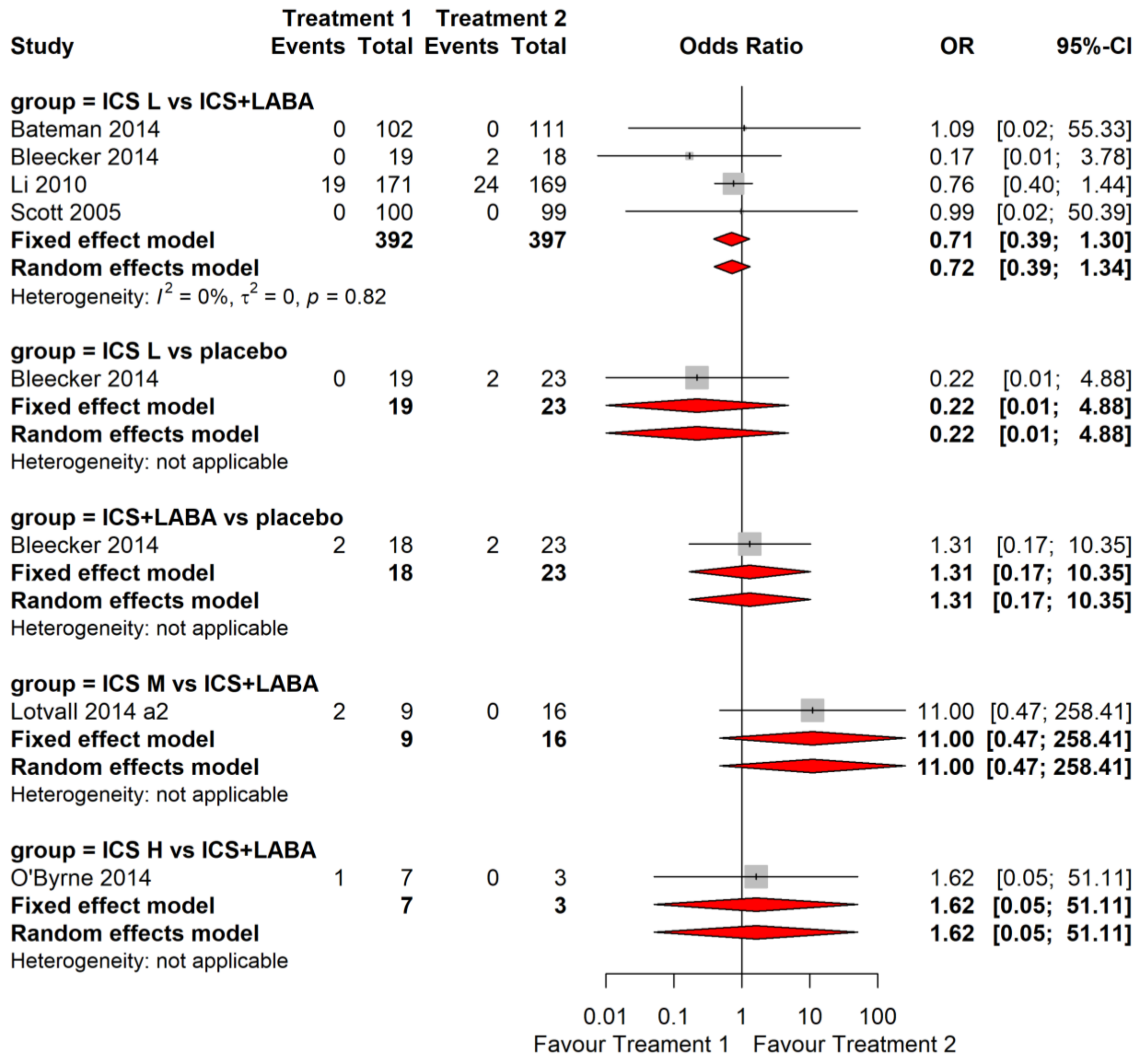

Meta-analysis with a frequentist approach (Mantel-Haenszel) based on all available comparisons. All data included (IPD only). OR > 1 favours treatment 2. IPD = individual participant data; AgD = aggregate data; ICS = inhaled corticosteroids; LABA = long-acting beta-agonists; LTRA = leukotriene receptor antagonists; L = low dose; M = medium dose; H = high dose; OR = odds ratio; CI = confidence interval.

Figure S19. Heart rate (HR) change (last visit vs baseline) (ICS dose grouped)

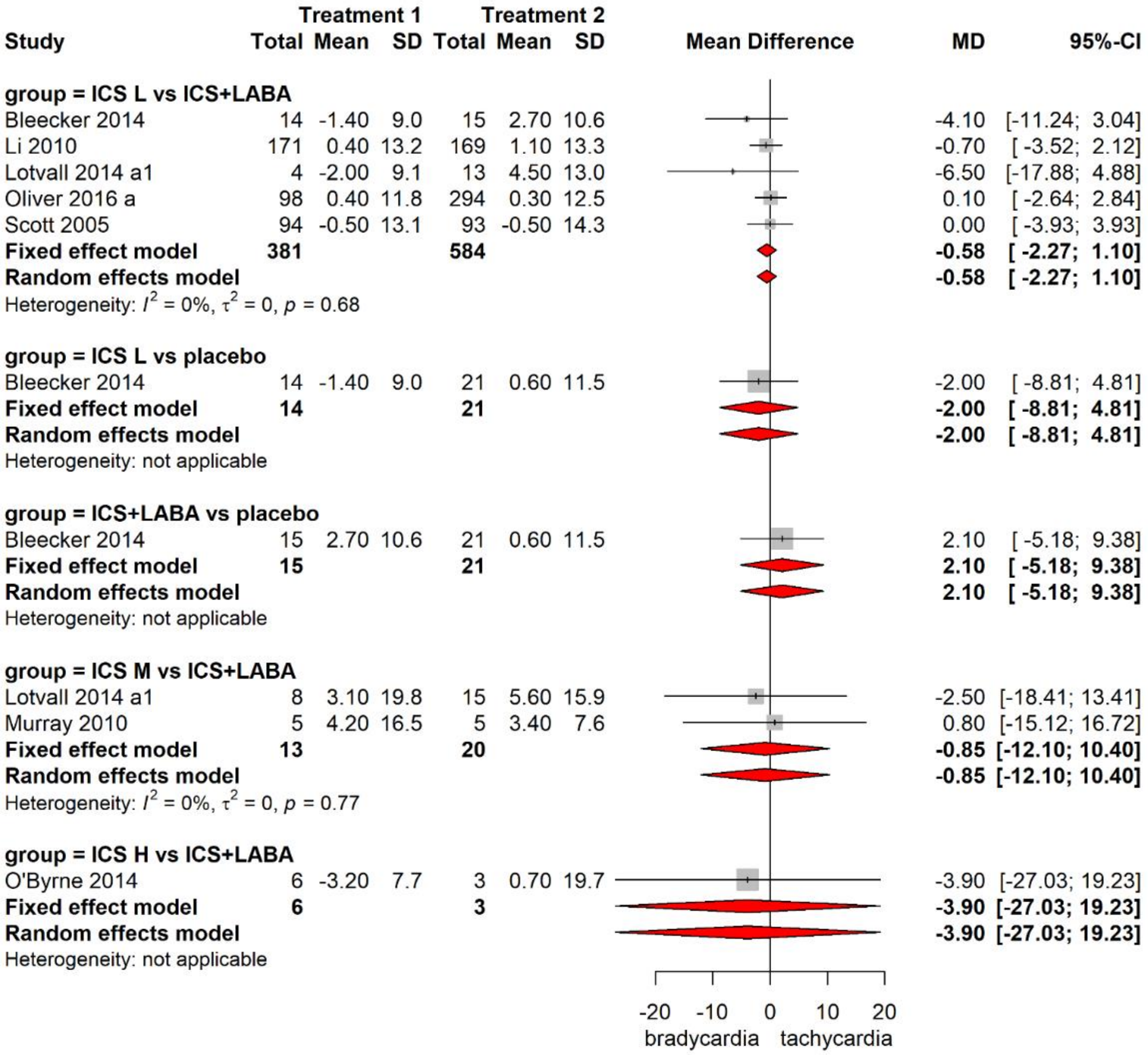

Meta-analysis with a frequentist approach (inverse variance) based on all available comparisons. All data included (IPD only).
When MD > 0, treatment 1 increases HR compared to treatment 2; when MD < 0, treatment 1 decreases HR compared to treatment 2.
IPD = individual participant data; AgD = aggregate data; ICS = inhaled corticosteroids; LABA = long-acting beta-agonists; LTRA = leukotriene receptor antagonists; L = low dose; M = medium dose; H = high dose; MD = mean difference; SD = standard deviation; CI = confidence interval.

Figure S20 (part 1). Infections and infestations (ICS dose grouped)

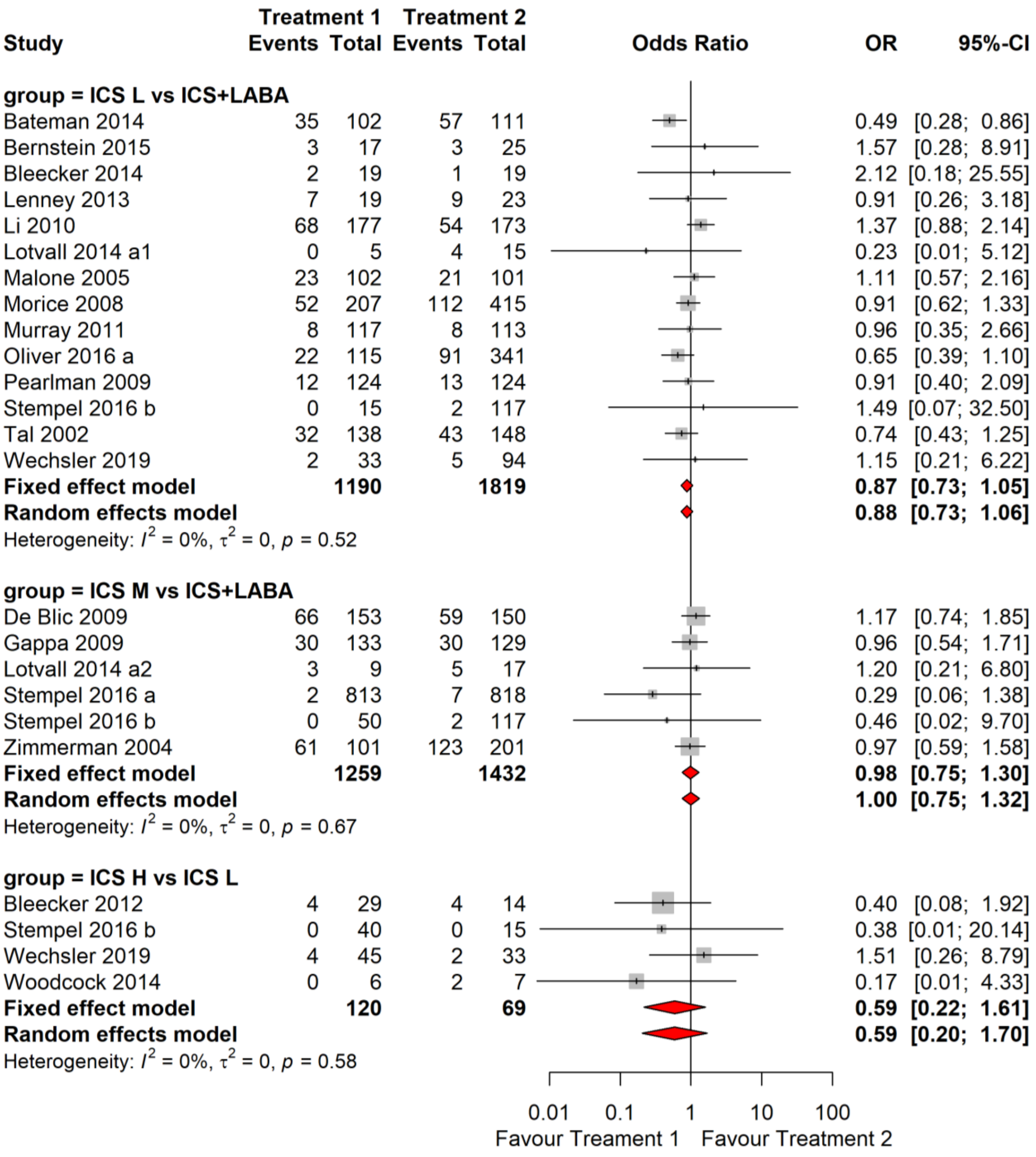

Meta-analysis with a frequentist approach (Mantel-Haenszel) based on all available comparisons. All data included (IPD and AgD where possible). OR > 1 favours treatment 2. IPD = individual participant data; AgD = aggregate data; ICS = inhaled corticosteroids; LABA = long-acting beta-agonists; LTRA = leukotriene receptor antagonists; L = low dose; M = medium dose; H = high dose; OR = odds ratio; CI = confidence interval.

Figure S20 (part 2). Infections and infestations (ICS dose grouped)

Study	Treatment 1 Events	Treatment 1 Total	Treatment 2 Events	Treatment 2 Total	OR	95%-CI
**group = ICS H vs ICS M**						
Bleecker 2012	4	29	1	13	1.92	[0.19; 19.09]
Stempel 2016 b	0	40	0	50	1.25	[0.02; 64.22]
**Fixed effect model**		**69**		**63**	**1.74**	**[0.24; 12.43]**
**Random effects model**					**1.72**	**[0.24; 12.52]**
Heterogeneity: $I^2 = 0\%$, $\tau^2 = 0$, $p = 0.85$						
**group = ICS H vs ICS+LABA**						
Akpinarli 1990	0	16	0	16	1.00	[0.02; 53.46]
O Byrne 2014	2	7	1	3	0.80	[0.04; 14.64]
Russell 1995	7	107	7	99	0.92	[0.31; 2.72]
Stempel 2016 b	0	40	2	117	0.57	[0.03; 12.13]
Wechsler 2019	4	45	5	94	1.74	[0.44; 6.81]
**Fixed effect model**		**215**		**329**	**1.07**	**[0.50; 2.31]**
**Random effects model**					**1.09**	**[0.50; 2.35]**
Heterogeneity: $I^2 = 0\%$, $\tau^2 = 0$, $p = 0.94$						
**group = ICS H vs placebo**						
Bleecker 2012	4	29	3	13	0.53	[0.10; 2.82]
**Fixed effect model**		**29**		**13**	**0.53**	**[0.10; 2.82]**
**Random effects model**					**0.53**	**[0.10; 2.82]**
Heterogeneity: not applicable						
**group = ICS L vs ICS M**						
Bleecker 2012	4	14	1	13	4.80	[0.46; 50.16]
Lotvall 2014 b	6	17	5	11	0.65	[0.14; 3.08]
Shapiro 2001	29	90	34	93	0.82	[0.45; 1.52]
Stempel 2016 b	0	15	0	50	3.26	[0.06; 171.09]
**Fixed effect model**		**136**		**167**	**0.93**	**[0.54; 1.59]**
**Random effects model**					**0.91**	**[0.52; 1.57]**
Heterogeneity: $I^2 = 0\%$, $\tau^2 = 0$, $p = 0.46$						
**group = ICS L vs placebo**						
Berger 2006	43	197	25	99	0.83	[0.47; 1.45]
Bleecker 2012	4	14	3	13	1.33	[0.24; 7.56]
Bleecker 2014	2	19	3	23	0.78	[0.12; 5.26]
Lotvall 2014 b	6	17	5	18	1.42	[0.34; 5.94]
Oliver 2016 b	50	253	12	65	1.09	[0.54; 2.19]
Shapiro 2001	29	90	29	91	1.02	[0.54; 1.90]
**Fixed effect model**		**590**		**309**	**0.98**	**[0.70; 1.37]**
**Random effects model**					**0.98**	**[0.70; 1.37]**
Heterogeneity: $I^2 = 0\%$, $\tau^2 = 0$, $p = 0.97$						

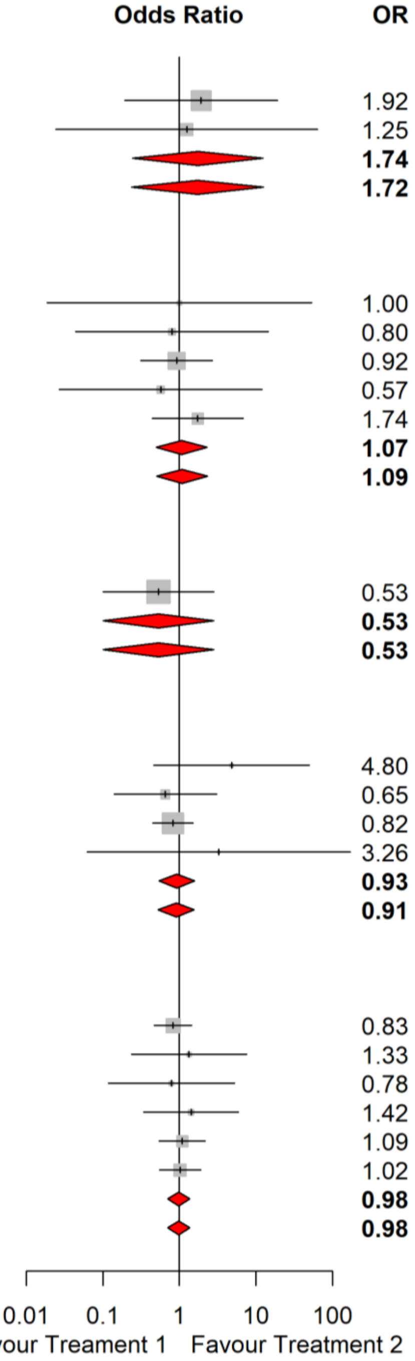

Meta-analysis with a frequentist approach (Mantel-Haenszel) based on all available comparisons. All data included (IPD and AgD where possible).
OR > 1 favours treatment 2. IPD = individual participant data; AgD = aggregate data; ICS = inhaled corticosteroids; LABA = long-acting beta-agonists; LTRA = leukotriene receptor antagonists; L = low dose; M = medium dose; H = high dose; OR = odds ratio; CI = confidence interval.

Figure S20 (part 3). Infections and infestations (ICS dose grouped)

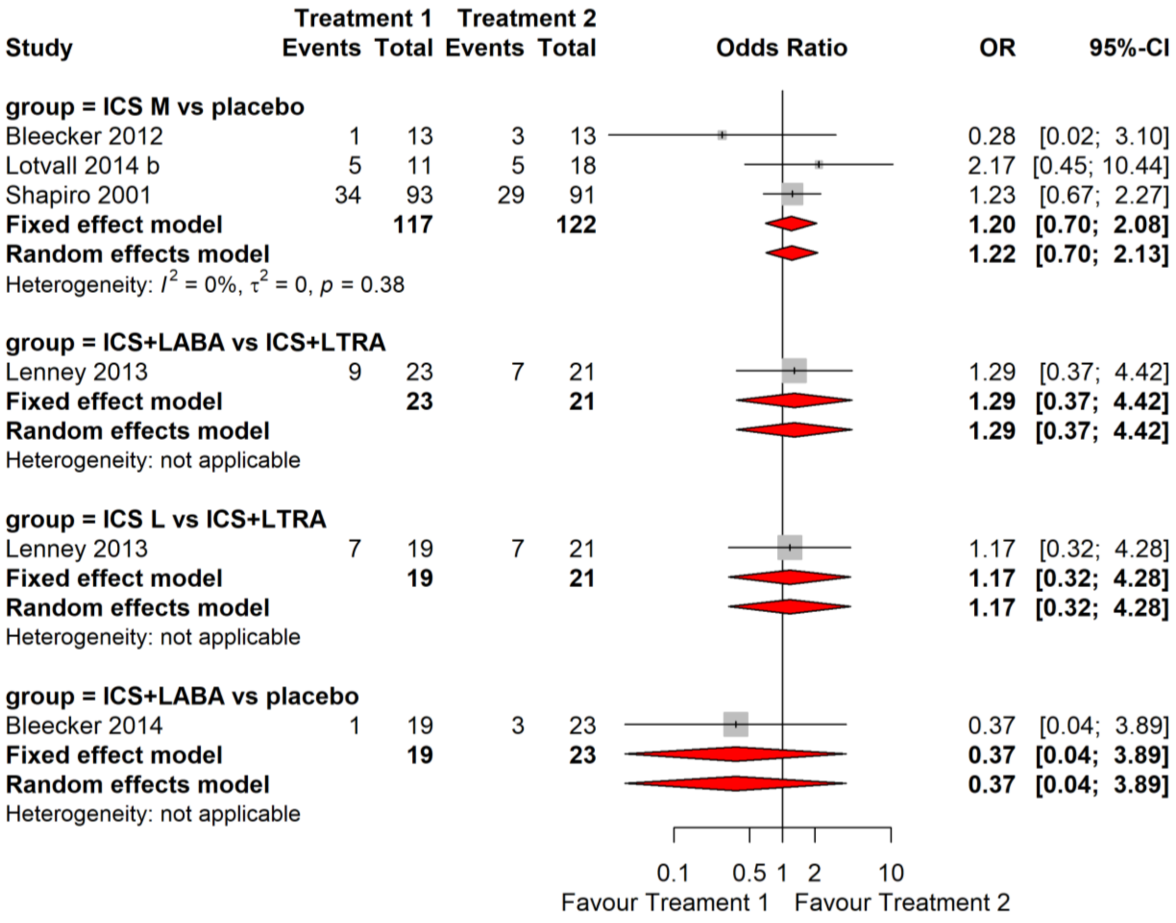

Meta-analysis with a frequentist approach (Mantel-Haenszel) based on all available comparisons. All data included (IPD and AgD where possible).
OR > 1 favours treatment 2. IPD = individual participant data; AgD = aggregate data; ICS = inhaled corticosteroids; LABA = long-acting beta-agonists; LTRA = leukotriene receptor antagonists; L = low dose; M = medium dose; H = high dose; OR = odds ratio; CI = confidence interval.

Figure S21 (part 1). Neurological disorders (ICS dose grouped)

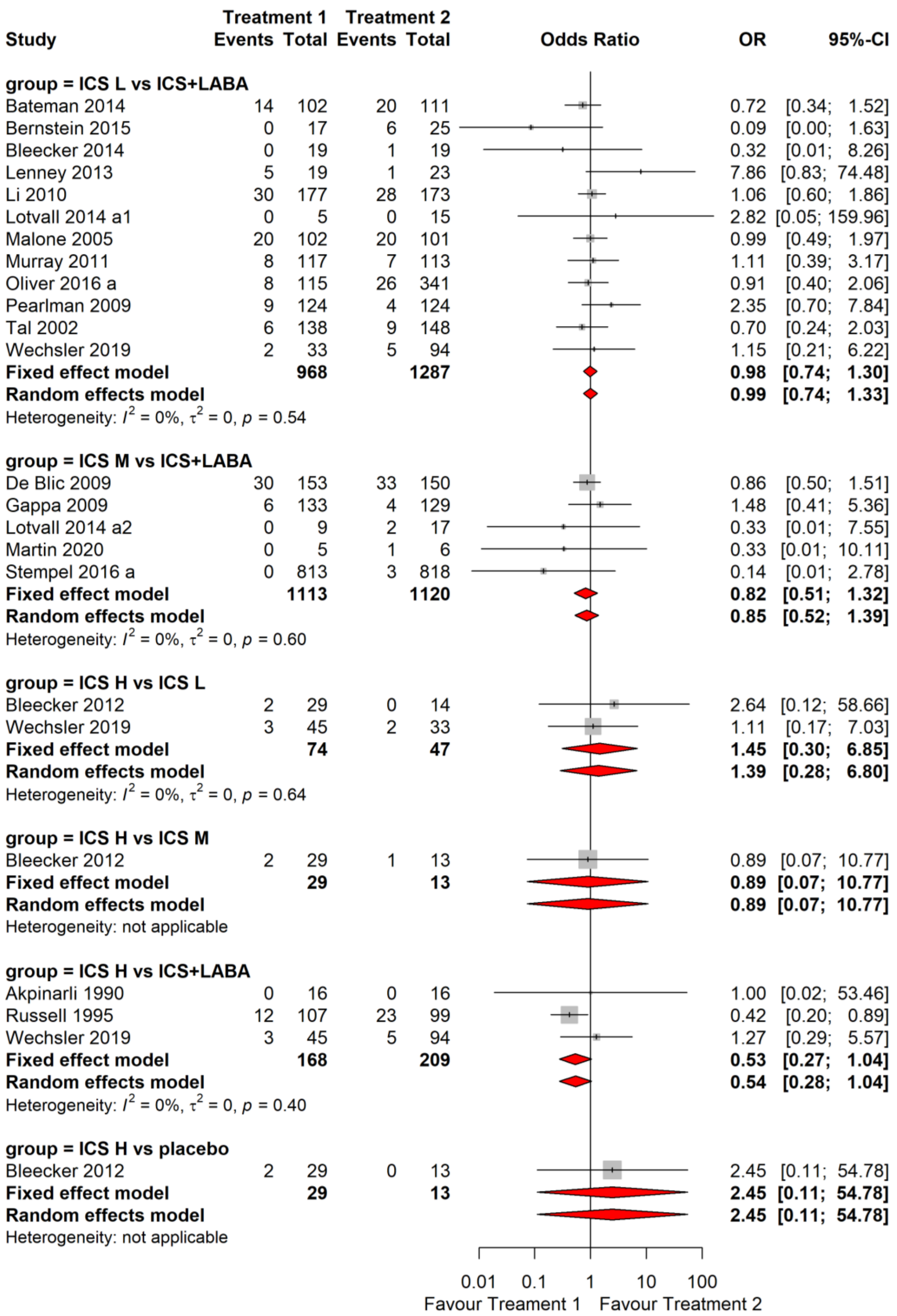

Meta-analysis with a frequentist approach (Mantel-Haenszel) based on all available comparisons. All data included (IPD and AgD where possible). OR > 1 favours treatment 2. IPD = individual participant data; AgD = aggregate data; ICS = inhaled corticosteroids; LABA = long-acting beta-agonists; LTRA = leukotriene receptor antagonists; L = low dose; M = medium dose; H = high dose; OR = odds ratio; CI = confidence interval

Figure S21 (part 2). Neurological disorders (ICS dose grouped)

Study	Treatment 1 Events	Treatment 1 Total	Treatment 2 Events	Treatment 2 Total	OR	95%-CI
**group = ICS L vs ICS M**						
Bleecker 2012	0	14	1	13	0.29	[0.01; 7.70]
Lotvall 2014 b	2	17	2	11	0.60	[0.07; 5.03]
Shapiro 2001	4	90	6	93	0.67	[0.18; 2.47]
**Fixed effect model**		**121**		**117**	**0.59**	**[0.21; 1.69]**
**Random effects model**					**0.60**	**[0.21; 1.72]**
Heterogeneity: $I^2 = 0\%$, $\tau^2 = 0$, $p = 0.89$						
**group = ICS L vs placebo**						
Berger 2006	17	197	14	99	0.57	[0.27; 1.22]
Bleecker 2012	0	14	0	13	0.93	[0.02; 50.30]
Bleecker 2014	0	19	2	23	0.22	[0.01; 4.88]
Lotvall 2014 b	2	17	1	18	2.27	[0.19; 27.58]
Oliver 2016 b	9	253	0	65	5.09	[0.29; 88.60]
Shapiro 2001	4	90	1	91	4.19	[0.46; 38.20]
**Fixed effect model**		**590**		**309**	**0.92**	**[0.51; 1.67]**
**Random effects model**					**1.02**	**[0.41; 2.49]**
Heterogeneity: $I^2 = 17\%$, $\tau^2 = 0.2415$, $p = 0.31$						
**group = ICS M vs placebo**						
Bleecker 2012	1	13	0	13	3.24	[0.12; 87.13]
Lotvall 2014 b	2	11	1	18	3.78	[0.30; 47.56]
Shapiro 2001	6	93	1	91	6.21	[0.73; 52.62]
**Fixed effect model**		**117**		**122**	**4.80**	**[1.12; 20.60]**
**Random effects model**					**4.63**	**[1.07; 19.98]**
Heterogeneity: $I^2 = 0\%$, $\tau^2 = 0$, $p = 0.93$						
**group = ICS L vs ICS+LTRA**						
Lenney 2013	5	19	7	21	0.71	[0.18; 2.80]
**Fixed effect model**		**19**		**21**	**0.71**	**[0.18; 2.80]**
**Random effects model**					**0.71**	**[0.18; 2.80]**
Heterogeneity: not applicable						
**group = ICS+LABA vs ICS+LTRA**						
Lenney 2013	1	23	7	21	0.09	[0.01; 0.82]
**Fixed effect model**		**23**		**21**	**0.09**	**[0.01; 0.82]**
**Random effects model**					**0.09**	**[0.01; 0.82]**
Heterogeneity: not applicable						
**group = ICS+LABA vs placebo**						
Bleecker 2014	1	19	2	23	0.58	[0.05; 6.98]
**Fixed effect model**		**19**		**23**	**0.58**	**[0.05; 6.98]**
**Random effects model**					**0.58**	**[0.05; 6.98]**
Heterogeneity: not applicable						
**group = ICS+LTRA vs ICS+Theophylline**						
Kondo 2006	1	39	0	36	2.84	[0.11; 72.08]
**Fixed effect model**		**39**		**36**	**2.84**	**[0.11; 72.08]**
**Random effects model**					**2.84**	**[0.11; 72.08]**
Heterogeneity: not applicable						

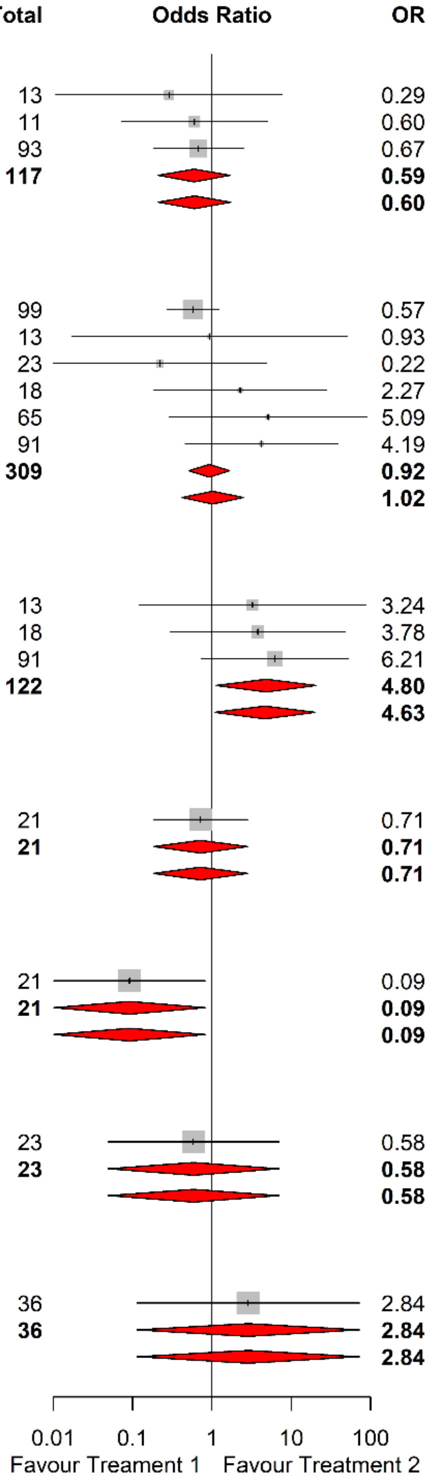

Meta-analysis with a frequentist approach (Mantel-Haenszel) based on all available comparisons. All data included (IPD and AgD where possible). OR > 1 favours treatment 2. IPD = individual participant data; AgD = aggregate data; ICS = inhaled corticosteroids; LABA = long-acting beta-agonists; LTRA = leukotriene receptor antagonists; L = low dose; M = medium dose; H = high dose; OR = odds ratio; CI = confidence interval.

Figure S22. Pneumonia (ICS dose grouped)

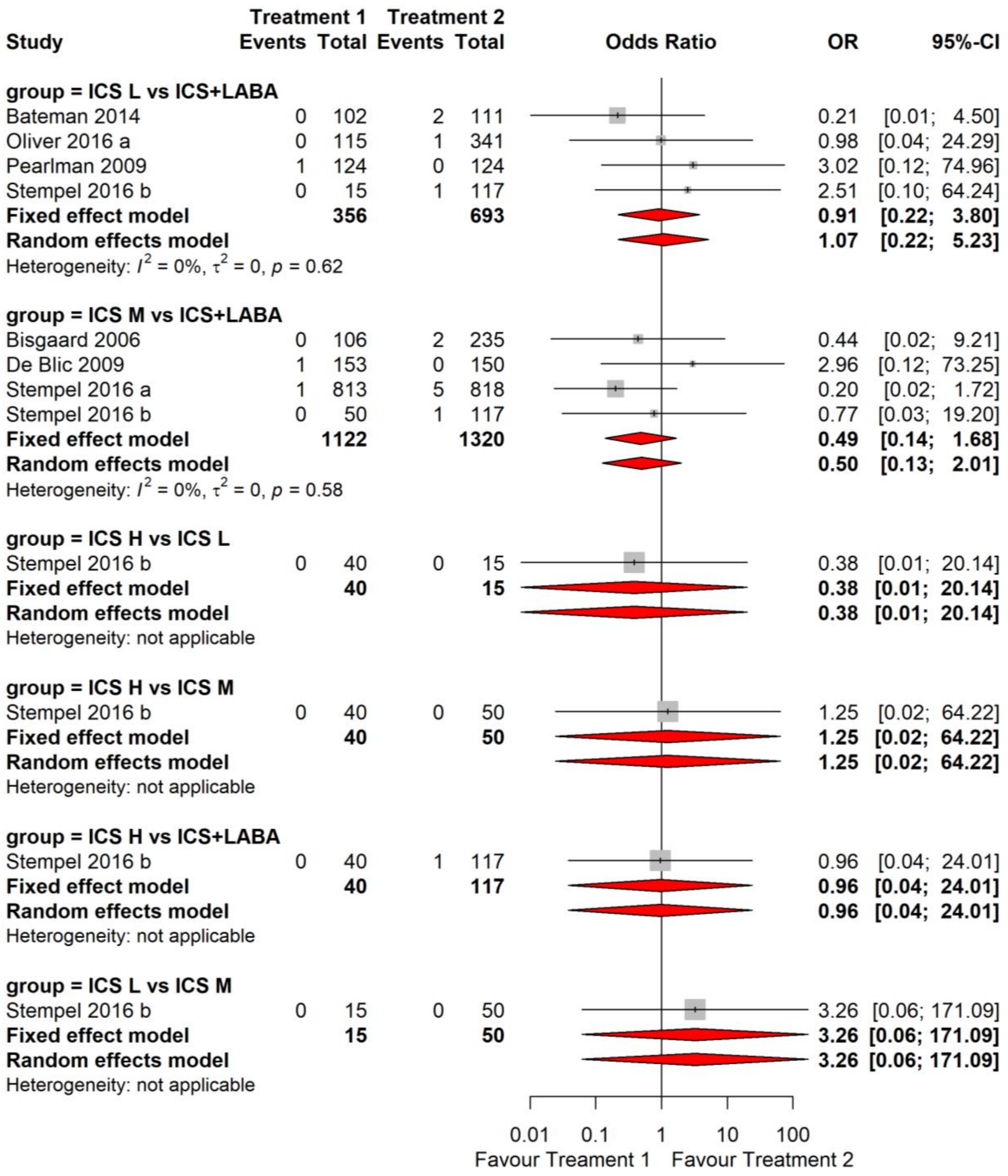

Meta-analysis with a frequentist approach (Mantel-Haenszel) based on all available comparisons. All data included (IPD and AgD where possible).
OR > 1 favours treatment 2. IPD = individual participant data; AgD = aggregate data; ICS = inhaled corticosteroids; LABA = long-acting beta-agonists; LTRA = leukotriene receptor antagonists; L = low dose; M = medium dose; H = high dose; OR = odds ratio; CI = confidence interval.
