# Supplementary material for: Best step-up treatments for children with uncontrolled asthma: a systematic review and network meta-analysis of individual participant data
Source: Eur Respir J. 2023 Dec 21;62(6):2301011. doi: 10.1183/13993003.01011-2023 (PMC10752294; doi:10.1183/13993003.01011-2023)
Supplement: Supplementary file 4 [file ERJ-01011-2023.Shareable.pdf]

EUROPEAN RESPIRATORY JOURNAL
ORIGINAL RESEARCH ARTICLE
S. CIVIDINI ET AL.

SHAREABLE PDF

Best step-up treatments for children with uncontrolled asthma: a systematic review and network meta-analysis of individual participant data

Sofia Cividini, Ian Sinha, Sarah Donegan, Michelle Maden, Katie Rose, Olivia Fulton, Giovanna Culeddu, Dyfrig A. Hughes, Stephen Turner and Catrin Tudur Smith on behalf of the EINSTEIN Collaborative Group

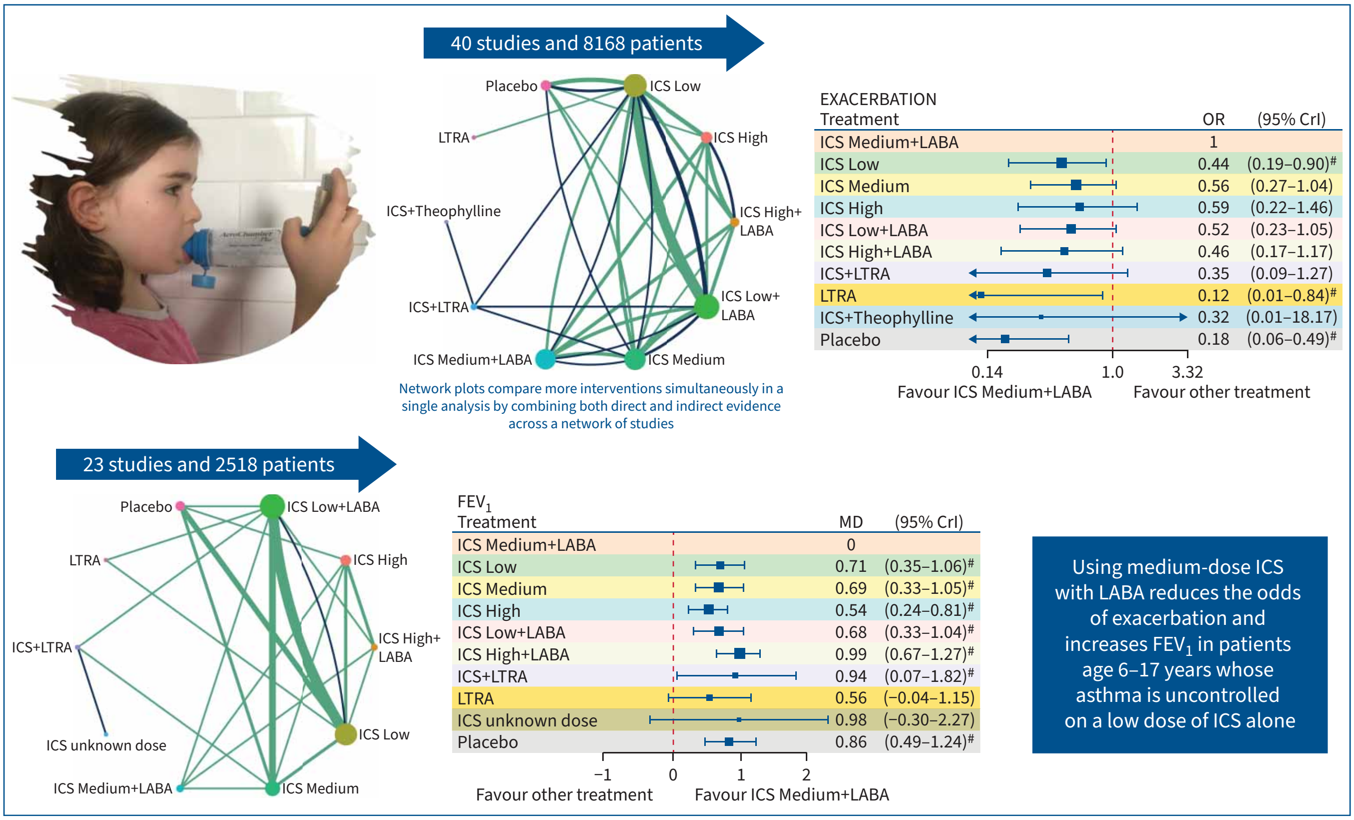

GRAPHICAL ABSTRACT The EstablishINg the best STEp-up treatments for children with uncontrolled asthma despite INhaled corticosteroids (EINSTEIN) study. Photograph provided by the International Primary Care Respiratory Group (IPCRG) under under Creative Commons licence CC BY-NC-SA. ICS: inhaled corticosteroid; LTRA: leukotriene receptor antagonist; LABA: long-acting β_2-agonist; OR: odds ratio; 95% CrI: 95% credibility interval; MD: mean difference; FEV_1: forced expiratory volume in 1 s. $^{\#}$: 95% CrIs that exclude the null value (1 or 0).

SHAREABLE PDF

Best step-up treatments for children with uncontrolled asthma: a systematic review and network meta-analysis of individual participant data

Sofia Cividini [1], Ian Sinha [2], Sarah Donegan [1], Michelle Maden [3], Katie Rose [2], Olivia Fulton [4], Giovanna Culeddu [5], Dyfrig A. Hughes [5], Stephen Turner [6,7] and Catrin Tudur Smith [1] on behalf of the EINSTEIN Collaborative Group

[1]Department of Health Data Science, Institute of Population Health, University of Liverpool, Liverpool, UK. [2]Alder Hey Children's Foundation NHS Trust, Liverpool, UK. [3]Liverpool Reviews and Implementation Group, Institute of Population Health, University of Liverpool, Liverpool, UK. [4]Patient Representative, Liverpool, UK. [5]Centre for Health Economics and Medicines Evaluation, Bangor University, Bangor, UK. [6]Women and Children Division, NHS Grampian, Aberdeen, UK. [7]Institute of Applied Health Sciences, University of Aberdeen, Aberdeen, UK.

Corresponding author: Catrin Tudur Smith (cat1@liverpool.ac.uk)

Check for updates

Shareable abstract (@ERSpublications)
Using medium-dose inhaled corticosteroids (ICS) with long-acting β_2-agonists reduces the odds of exacerbation and increases FEV_1 in patients age 6–17 years whose asthma is uncontrolled on a low dose of ICS alone. https://bit.ly/47buW6o

Cite this article as: Cividini S, Sinha I, Donegan S, *et al.* Best step-up treatments for children with uncontrolled asthma: a systematic review and network meta-analysis of individual participant data. *Eur Respir J* 2023; 62: 2301011 [DOI: 10.1183/13993003.01011-2023].

This extracted version can be shared freely online.

Copyright ©The authors 2023.

This version is distributed under the terms of the Creative Commons Attribution Licence 4.0.

Received: 14 June 2023
Accepted: 25 Oct 2023

Abstract

Background There is uncertainty about the best treatment option for children/adolescents with uncontrolled asthma despite inhaled corticosteroids (ICS) and international guidelines make different recommendations. We evaluated the pharmacological treatments to reduce asthma exacerbations and symptoms in uncontrolled patients age <18 years on ICS.

Methods We searched MEDLINE, Cochrane Database of Systematic Reviews, Cochrane Central Register of Controlled Trials, Embase, Web of Science, National Institute for Health and Care Excellence Technology Appraisals, National Institute for Health and Care Research Health Technology Assessment series, World Health Organization International Clinical Trials Registry, conference abstracts and internal clinical trial registers (1 July 2014 to 5 May 2023) for randomised controlled trials of participants age <18 years with uncontrolled asthma on any ICS dose alone at screening. Studies before July 2014 were retrieved from previous systematic reviews/contact with authors. Patients had to be randomised to any dose of ICS alone or combined with long-acting β_2-agonists (LABA) or combined with leukotriene receptor antagonists (LTRA), LTRA alone, theophylline or placebo. Primary outcomes were exacerbation and asthma control. The interventions evaluated were ICS (low/medium/high dose), ICS+LABA, ICS+LTRA, LTRA alone, theophylline and placebo.

Results Of the 4708 publications identified, 144 trials were eligible. Individual participant data were obtained from 29 trials and aggregate data were obtained from 19 trials. Compared with ICS Low, ICS Medium+LABA was associated with the lowest odds of exacerbation (OR 0.44, 95% credibility interval (95% CrI) 0.19–0.90) and with an increased forced expiratory volume in 1 s (mean difference 0.71, 95% CrI 0.35–1.06). Treatment with LTRA was the least preferred. No apparent differences were found for asthma control.

Conclusions Uncontrolled children/adolescents on low-dose ICS should be recommended a change to medium-dose ICS+LABA to reduce the risk for exacerbation and improve lung function.
